# Pd(II)-catalysed *meta*-C–H functionalizations of benzoic acid derivatives

**DOI:** 10.1038/ncomms10443

**Published:** 2016-01-27

**Authors:** Shangda Li, Lei Cai, Huafang Ji, Long Yang, Gang Li

**Affiliations:** 1 State Key Laboratory of Structural Chemistry, Fujian Institute of Research on the Structure of Matter, Chinese Academy of Sciences, Fujian 350002, China; 2 Key Laboratory of Coal to Ethylene Glycol and Its Related Technology, CAS, Fujian 350002, China

## Abstract

Benzoic acids are highly important structural motifs in drug molecules and natural products. Selective C–H bond functionalization of benzoic acids will provide synthetically useful tools for step-economical organic synthesis. Although direct *ortho*-C–H functionalizations of benzoic acids or their derivatives have been intensely studied, the ability to activate *meta*-C–H bond of benzoic acids or their derivatives in a general manner via transition-metal catalysis has been largely unsuccessful. Although chelation-assisted *meta*-C–H functionalization of electron-rich arenes was reported, chelation-assisted *meta*-C–H activation of electron-poor arenes such as benzoic acid derivatives remains a formidable challenge. Herein, we report a general protocol for *meta*-C–H olefination of benzoic acid derivatives using a nitrile-based sulfonamide template. A broad range of benzoic acid derivatives are *meta*-selectively olefinated using molecular oxygen as the terminal oxidant. The *meta*-C–H acetoxylation, product of which is further transformed at the *meta*-position, is also reported.

## Abstract (web summary)

While directed ortho-metalation is a well-established protocol, meta-functionalisation of aromatic groups is more challenging. Here, the authors report a directed meta-C-H functionalisation of benzoic acid derivatives, using a nitrile-based sulfonamide directing group.

## Graphical abstract

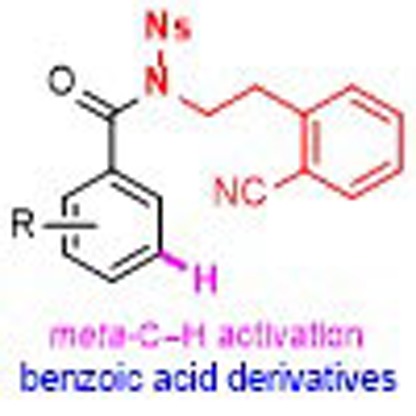

Benzoic acids are highly valuable structural motifs and precursors for synthesizing other organic substances. Direct and selective transformation of the C–H bonds of this class of compounds would be very attractive for developing step-economical organic syntheses (Fig. 1)^1^,^2^,^3^,^4^,^5^,^6^,^7^,^8^,^9^,^10^,^11^,^12^,^13^,^14^. To date, direct *ortho*-C–H functionalizations of benzoic acids or their derivatives have been intensely studied with transition-metal catalysts^1^,^2^,^3^,^4^,^5^,^6^,^7^,^8^,^9^,^10^,^11^,^12^ or by directed *ortho* metalation^13^. Traditionally, the *meta*-position of benzoic acids were functionalized by electrophilic aromatic substitution, which often requires harsh conditions as benzoic acids are generally deactivated towards this reaction^14^. Transition-metal catalysts have been used to functionalize *meta*-C–H bonds of limited benzoic acid derivatives. Hartwig, Yu, Sanford and others have achieved modest to high selectivity because of substrate steric or electronic control^15^,^16^,^17^,^18^,^19^,^20^,^21^,^22^,^23^,^24^,^25^. In prior examples, an excess of the benzoic acid derivative, which was often used as the solvent, was generally required with a Pd catalyst (Fig. 1a)^17^,^18^,^21^. Despite these highly important pioneering studies, the ability to activate the *meta*-C–H bond of benzoic acids or their derivatives in a general manner via transition-metal catalysis has been largely unsuccessful^25^. Therefore, a general approach to *meta*-C–H functionalization of benzoic acids or their derivatives regardless of the substitution patterns is highly desirable to provide synthetic short-cuts.

Controlling the site-selectivity of C–H activation reactions is an outstanding challenge in the development syntheticially useful C–H functinalization methodology^26^,^27^. So far only a limited number of approaches are available for addressing *meta*-C–H functionalizations of arenes^28^,^29^,^30^,^31^,^32^,^33^,^34^,^35^,^36^,^37^,^38^,^39^,^40^,^41^,^42^,^43^,^44^,^45^,^46^,^47^,^48^,^49^,^50^,^51^,^52^,^53^,^54^,^55^,^56^,^57^,^58^,^59^,^60^,^61^,^62^,^63^. These approaches include inherent substrate control via steric and/or electronic factors^28^,^29^,^30^,^31^,^32^,^33^,^34^,^35^, chelating group-assisted Cu(II)-catalysed arylation^36^,^37^, ruthenium(II) complex facilitated *meta*-C–H functionalizations^38^,^39^,^40^,^41^,^42^,^43^, the use of transient norbornene mediator^44^,^45^ and formal *meta*-C–H functionalizations utilizing traceless directing groups^46^,^47^,^48^,^49^,^50^,^51^. Another unique method is the use of nitrile-based templates for *meta*-C–H functionalizations of electron-rich arenes, such as hydrocinnamic acids and phenylacetic acids, which was pioneered by the group of Yu^54^,^55^,^56^,^57^,^58^,^59^,^60^,^61^,^62^,^63^. However, chelation-assisted *meta*-C–H activation of electron-poor arenes such as benzoic acid derivatives remains a formidable challenge, possibly due to the low reactivity of the electron-poor arenes towards palladation in this type of C–H activation.

Herein, we disclose our discovery of a recyclable nitrile-based sulfonamide template that promotes the olefination and acetoxylation of *meta*-C–H bonds of a broad range of benzoic acid derivatives (Fig. 1b). Notably, a protocol is developed that enables the use of environmentally benign molecular oxygen as the terminal oxidant for chelation-assisted *meta*-C–H olefination, which previously required the use of costly silver salt oxidants.

## Results

### Development of *meta*-C–H olefination reaction conditions

As benzoic acids are both electronically and structurally distinct from other electron-rich arenes that undergo template-assisted *meta*-C–H functinalizations, the elaboration of a compatible template-directing group is required to accommodate their unique properties. After investigating several newly designed templates (see Supplementary Table 1 for details), we found amide **1a** bearing a highly electron-withdrawing nosyl group was the most promising substrate. After **1a** was subjected to the similar reaction conditions that were developed by us for *meta*-C–H olefination^64^,^65^,^66^,^67^ of phenylethylamines^63^, much to our delight, excellent yields of desired products were obtained with tiny traces of other olefinated isomers (Table 1, entry 1). To avoid using the costly silver acetate as the oxidant, we continued to optimize the reaction conditions to search for a less costly oxidant. After screening a few inorganic as well as organic oxidants (entries 2–5), we were very surprised to find Cu(OAc)_2_, which might compete with Pd(OAc)_2_ in coordination with the weakly coordinating nitrile group, could be used as an effective oxidant (entry 2). The yield was increased to 72% when the reaction was run under oxygen atmosphere (entry 6)^10^,^68^,^69^. Notably, better results were obtained by increasing the loading of Ac-Gly-OH ligand (entries 7–8), leading to almost full conversion of the substrate with 60 mol% of the ligand (entry 8). By further tuning the loading of Cu(OAc)_2_, the reaction time and temperature (entries 9–13), the best result was achieved in 48 h at 90 °C using oxygen as the terminal oxidant with catalytic amount of Cu(OAc)_2_ (entry 13). It was found that the reaction was almost shut down without adding Cu(OAc)_2_ as co-oxidant (entry 12). The use of oxygen is notable as all the previous chelation-assisted *meta*-C–H olefination reactions required the use of silver salt as the oxidant^54^,^55^,^56^,^57^,^58^,^59^,^60^,^61^,^62^,^63^. Finally, the reaction conditions were carefully tuned to improve the mono versus di-olefination selectivity (see also Supplementary Table 1 for details), although the two products could be easily separated. Pleasingly, the use of Formyl-Gly-OH ligand with an inorganic base would result in good mono versus di-olefination selectivities (entries 14–15)^59^, albeit in lower overall yield. Interestingly, this selectivity was switched when no inorganic base was added (entry 16).

### Substrate scope of *meta*-C–H olefination

**Figure 2:**
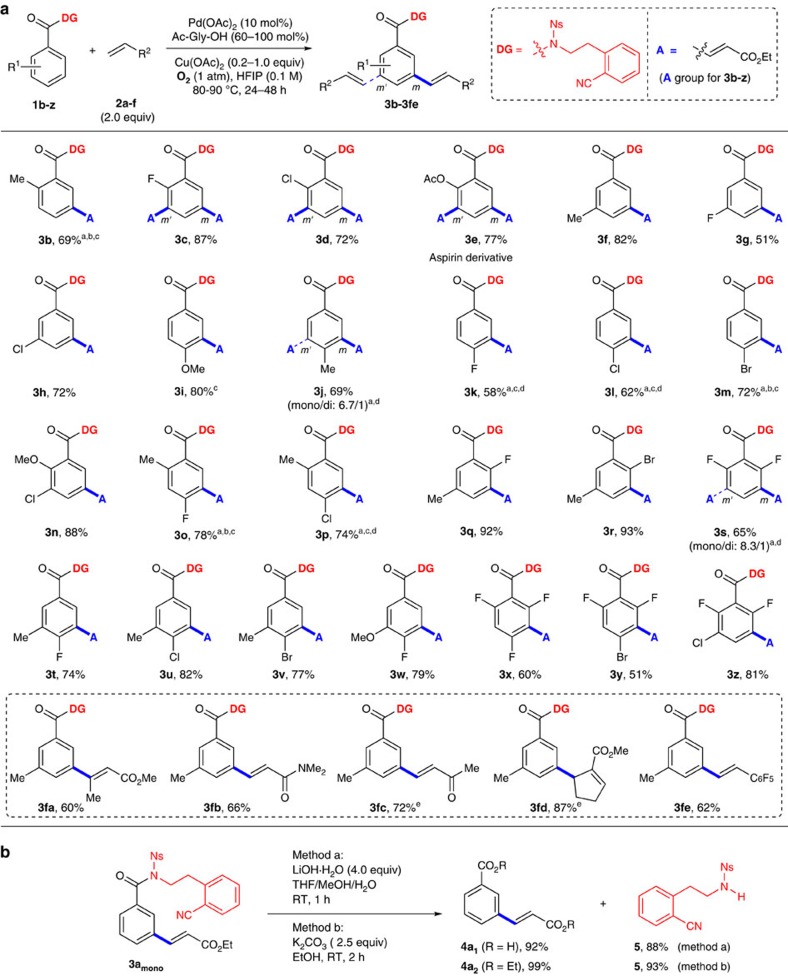
Substrate scope and removal and recycle of the template. (**a**) Substrate scope of *meta*-C–H olefination. Reaction conditions: **1** (0.1–0.2 mmol), **2** (2.0 equiv), Pd(OAc)_2_ (10 mol%), Ac-Gly-OH (60–100 mol%), Cu(OAc)_2_ (0.2–1.0 equiv), O_2_ (1 atm), HFIP (1–2 ml), 80–90 °C, 24–48 h, see the Supplementary Methods for details. Isolated yields are reported. ^a^Formyl-Gly-OH (60 mol%) was used instead of Ac-Gly-OH. ^b^KH_2_PO_4_ (0.5 equiv) was added. ^c^A trace of (*m,m'*)-diolefinated product was observed. ^d^K_2_HPO_4_ (0.5 equiv) was added. ^e^AgOAc (3.0 equiv) was used instead of O_2_/Cu(OAc)_2_. (**b**) Two mild methods available for regenerating auxiliary **5**.

With the optimized conditions in hand, we carried out olefinations on a variety of benzoic acid derivatives, which were easily prepared in one step from benzoic acids using routine conditions (see Supplementary Methods for details). It was found that both electron-donating and electron-withdrawing *ortho*-substituents were well tolerated (Fig. 2a, **3b**–**3e**), and a good yield of mono-olefinated product was obtained with substrate **1b**. Di-olefination occurred predominantly with less hindered amides **1c**–**1e**. Notably, our method provided a direct access to an aspirin derivative (**3e**). The reaction also proceeded smoothly with *meta*-substituted substrates (**3f**–**3h**). *Para*-substituted benzamides with electron-donating methoxy (**3i**) and methyl (**3j**) groups as well as electron-withdrawing fluoro (**3k**), chloro (**3l**) and bromo (**3m**) groups were all suitable substrates, affording mono-olefinated products selectively. Interesting, the bromo group (**3m**) was tolerated in our protocol, which is synthetically useful for further elaborations of the product. It should be noted di-olefination products with high overall yields could also be produced when KH_2_PO_4_ was used as the base for substrates **1j**–**1l** (see Supplementary Methods for details). Importantly, the method was compatible with a range of substrates carrying two substituents (**3n**–**3w**), generally producing high yields of desired products. It is surprising that tri-substituted substrates were also able to afford desired products in moderate to high yields (**3x**–**3z**). Such highly substituted patterns were not observed in all previous transition-metal catalysed *meta*-C–H functionalizations. Desired products were also generated with other electron-deficient olefin-coupling partners (**3fa**–**3fe**), although production of **3fc** and **3fd** required silver acetate as the oxidant since the standard conditions only afforded low yields of these two products. The *meta*-selectivity was generally excellent, although traces of isomers were observed for some substrates. However, it was hard to assign the peaks of isomers that were only traces from the crude ^1^H NMR and thus we did not attempt to calculate the exact ratios of isomers. Finally, the template-directing group could be removed and recycled readily with LiOH or K_2_CO_3_ in high yields as shown in Fig. 2b. Moreover, the auxiliary **5** could be synthesized in multi-gram scale from inexpensive chemicals (see Supplementary Methods for details).

### *Meta*-C–H acetoxylation

**Figure 3:**
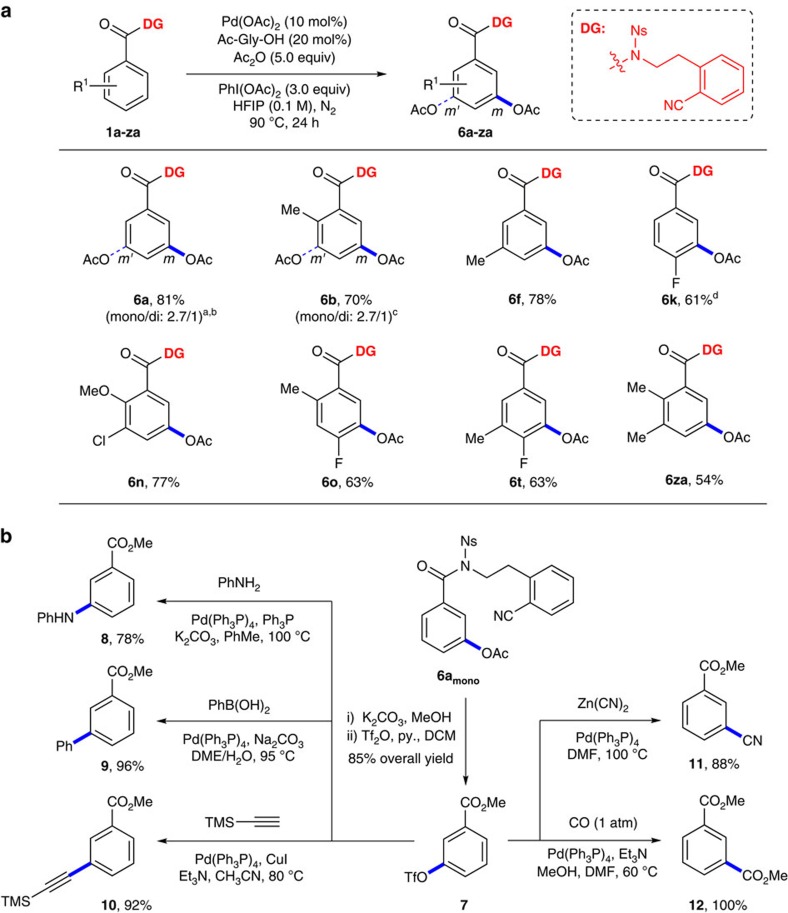
*Meta*-C–H acetoxylation and synthetic elaboration. (**a**) *Meta*-C–H acetoxylation. Reaction conditions: **1** (0.1 mmol), Pd(OAc)_2_ (10 mol%), Ac-Gly-OH (20 mol%), Ac_2_O (5.0 equiv), PhI(OAc)_2_ (3.0 equiv), HFIP (1 ml), N_2_, 90 °C, 24 h. Isolated yields are reported; see the Supplementary Methods for details. ^a^A trace of regioisomer was observed. ^b^The yield was 60% (mono/di: 4.8:1) in 1.3 mmol scale. ^c^6 h. ^d^A trace of (*m,m'*)-di-acetoxylated product was observed. (**b**) Application potential of *meta*-acetoxylation demonstrated with further elaborations.

The versatility of our sulfonamide template with different catalytic cycles was investigated briefly with *meta*-acetoxylation of benzoic acid derivatives using the previously established oxidation conditions (Fig. 3a)^70^. Although the acetoxylation was generally less efficient than olefination, several substrates with different substitution patterns underwent *meta*-acetoxylation successfully to give desired products in moderate to good yields (**6a–6za**). Moreover, 60% combined yield of products was obtained when the reaction was performed in 1.3 mmol scale (**6a**).

### Synthetic elaboration

To demonstrate the utility of our *meta*-C–H functionalizations, we attempted further elaboration of the acetoxylated product (Fig. 3b). Thus, triflate **7** was prepared in high yield from **6a**_**mono**_ by hydrolysis of the template and the acetoxy group in one step, which was followed by triflation of the resulting hydroxyl group. Triflate **7** was then transformed to a range of synthetically useful substances with well-established coupling reactions, namely, amination (**8**), arylation (**9**), alkynylation (**10**), cyanation (**11**) and carbonylation (**12**), greatly expanding the application potential of our method.

### Proposed catalytic cycle

**Figure 4:**
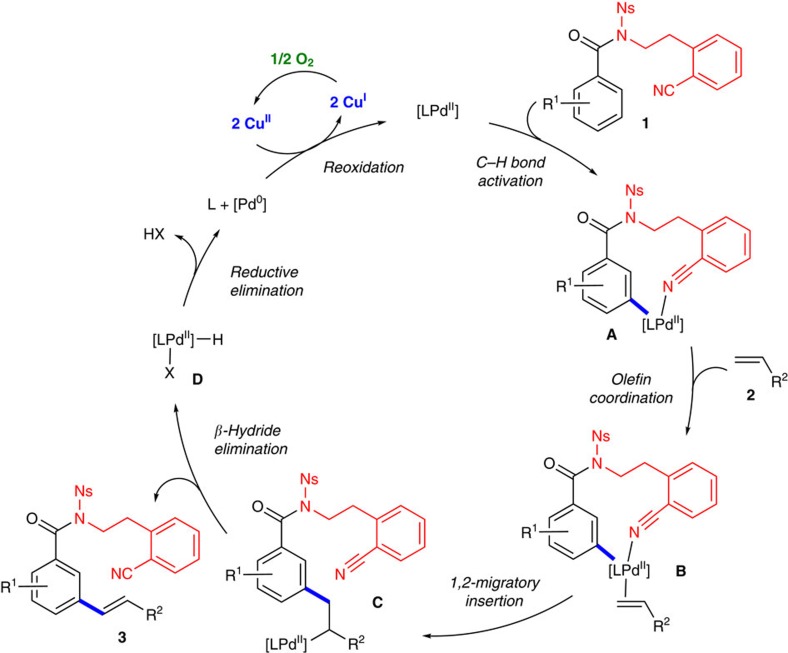
Proposed catalytic cycle of *meta*-C–H olefination. The plausible mechanism involves regeneration of a Pd(II) catalyst with only catalytic amount of Cu(II) species.

On the basis of the above results, a plausible catalytic cycle is proposed for *meta*-C–H olefination (Fig. 4). The complex **A** is generated by coordination of substrate **1** to a Pd(II) species followed by template-directed insertion of Pd(II) into the *meta*-C–H bond of **1**. Following coordination of **A** with olefin **2**, the resulting complex **B** undergoes 1,2-migratory insertion to give intermediate **C**. Product **3** is then afforded by *β*-hydride elimination of **C**. Reductive elimination of hydride **D** produces Pd(0), which is reoxidized to Pd(II) by two equivalents of Cu(II). The resulting Cu(I) is then oxidized by molecular oxygen to re-enter the catalytic cycle.

## Discussion

In summary, a general protocol for *meta*-C–H bond olefination of a broad range of benzoic acid derivatives assisted by a nitrile-based sulfonamide template has been developed. Desired *meta*-functionalized products were obtained regardless of the substitution patterns and steric biases of the substrates. Notably, the challenging tri-substituted substrates were tolerated, which was not observed in previous transition-metal catalysed *meta*-C–H functionalizations^15^,^16^,^17^,^18^,^19^,^20^,^21^,^22^,^23^,^24^,^25^,^26^,^27^,^28^,^29^,^30^,^31^,^32^,^33^,^34^,^35^,^36^,^37^,^38^,^39^,^40^,^41^,^42^,^43^,^44^,^45^,^46^,^47^,^48^,^49^,^50^,^51^,^52^,^53^,^54^,^55^,^56^,^57^,^58^,^59^,^60^,^61^,^62^,^63^. Importantly, the new protocol was compatible with molecular oxygen as the terminal oxidant. Moreover, the sulfonamide template auxiliary could be efficiently removed and recycled under mild conditions. Finally, the versatility of our template was demonstrated with *meta*-C–H acetoxylation, which enabled the access to five synthetically useful major classes of substitents at the *meta*-position of benzoic acid derivatives. It is expected that the protocol disclosed herein will soon inspire the development of more synthetically useful *meta*-C–H transformations for benzoic acid derivatives.

## Methods

### General methods

For ^1^H and ^13^C NMR spectra of compounds in this manuscript and details of the synthetic procedures, see Supplementary Figs 1–102 and Supplementary Methods.

### General procedure for *meta*-C–H olefination

To a 50-ml Schlenk sealed tube (with a Teflon cap) equipped with a magnetic stir bar was charged with amide **1** (0.10 mmol, 1.0 equiv), Pd(OAc)_2_ (2.3 mg, 0.010 mmol, 10 mol%), Ac-Gly-OH (20–100 mol%) and Cu(OAc)_2_ (0.2–1.0 equiv) sequentially. Hexafluoro-2-propanol (HFIP; 1.0 ml) was added to the mixture along the inside wall of the tube, followed by the corresponding alkene **2** (2.0 equiv). The reaction tube was capped, then evacuated briefly under vacuum and charged with O_2_ (1 atm, balloon, × 3). The tube was then submerged into a preheated 80 or 90 °C oil bath. The reaction was stirred for 24–48 h and cooled to room temperature. The crude reaction mixture was diluted with EtOAc (5 ml) and filtered through a short pad of Celite. The sealed tube and Celite pad were washed with an additional 20 ml of EtOAc. The filtrate was concentrated *in vacuo*, and the resulting residue was purified by flash silica gel chromatography or preparative thin layer chromatography using petroleum ether/EtOAc as the eluent. The site selectivity was assigned by NMR analysis of the product or the hydrolysed product. Full experimental details and characterization of new compounds can be found in the Supplementary Methods.

### General procedure for *meta*-C–H acetoxylation

To a 50-ml Schlenk sealed tube (with a Teflon cap) equipped with a magnetic stir bar was charged with amide **1** (0.10 mmol, 1.0 equiv), Pd(OAc)_2_ (2.3 mg, 0.010 mmol, 10 mol%), Ac-Gly-OH (2.4 mg, 0.020 mmol, 20 mol%) and PhI(OAc)_2_ (96.6 mg, 0.30 mmol, 3 equiv). HFIP (1.0 ml) was added to the mixture along the inside wall of the tube, followed by Ac_2_O (47 μl, 5 equiv). The reaction tube was capped, then evacuated briefly under vacuum and charged with N_2_ (1 atm, balloon, × 3). The tube was then submerged into a preheated 90 °C oil bath. The reaction was stirred for 24 h and cooled to room temperature. The crude reaction mixture was diluted with EtOAc (5 ml) and filtered through a short pad of Celite. The sealed tube and Celite pad were washed with an additional 20 ml of EtOAc. The filtrate was concentrated *in vacuo*, and the resulting residue was purified by flash silica gel chromatography using petroleum ether/EtOAc as the eluent. Full experimental details and characterization of new compounds can be found in the Supplementary Methods.

## Additional information

**How to cite this article:** Li, S. *et al*. Pd(II)-catalysed *meta*-C–H functionalizations of benzoic acid derivatives. *Nat. Commun*. 7:10443 doi: 10.1038/ncomms10443 (2016).

## Supplementary Material

Supplementary Information Supplementary Figures 1-102, Supplementary Table 1, Supplementary Methods and Supplementary References

## Figures and Tables

**Figure 1:**
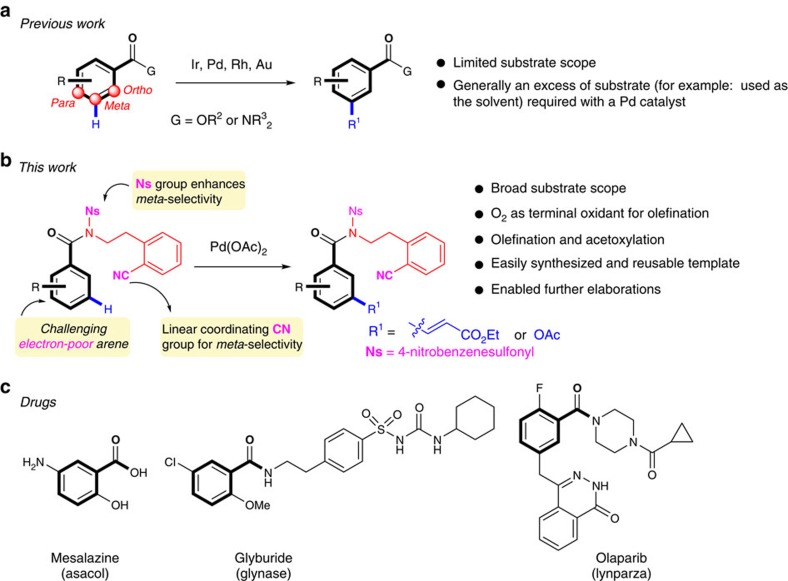
Transition-metal-catalysed *meta*-C–H functionalizations of benzoic acid derivatives. (**a**) Previous reports on *meta*-C–H functionalizations of limited benzoic acid derivatives. (**b**) Our design of template for *meta*-C–H functionalizations of electron-poor benzoic acid derivatives and the highlights of our work. (**c**) Representative drugs of benzoic acid derivatives with *meta*-substituents.

**Table 1:**
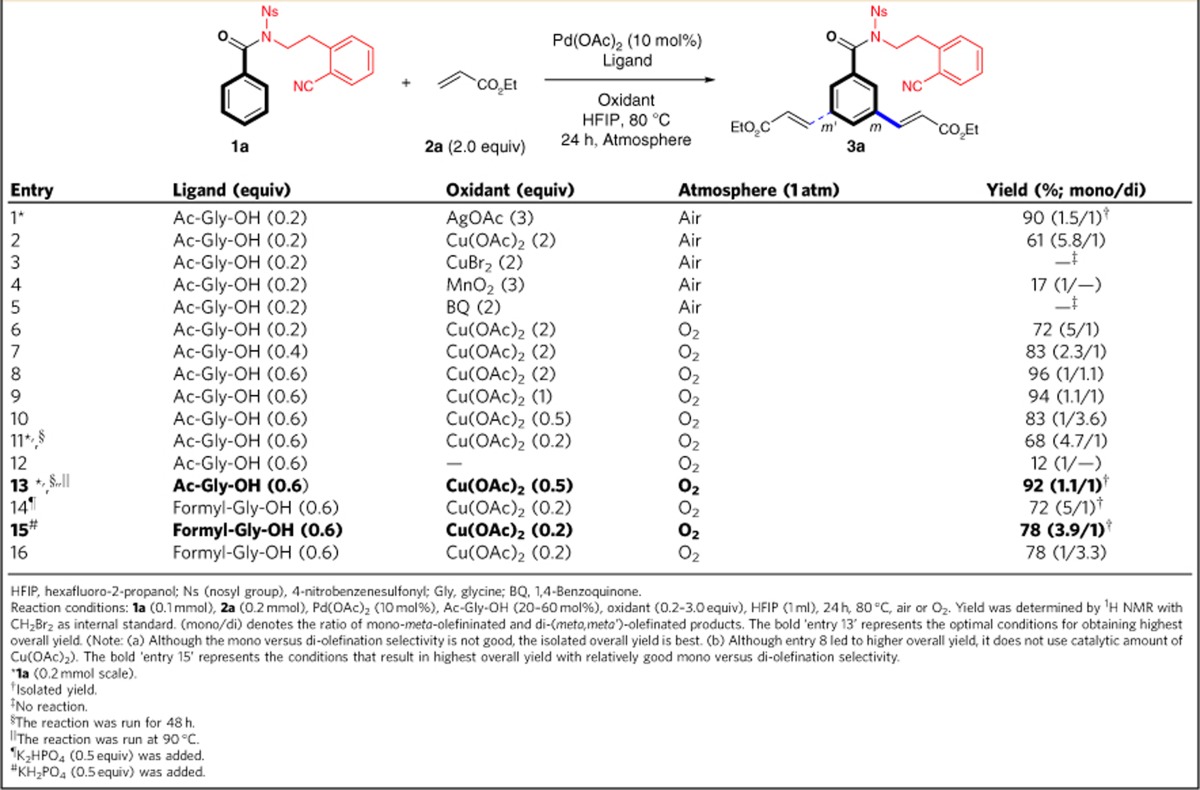
Optimization of reaction conditions.

HFIP, hexafluoro-2-propanol; Ns (nosyl group), 4-nitrobenzenesulfonyl; Gly, glycine; BQ, 1,4-Benzoquinone.

Reaction conditions: **1a** (0.1 mmol), **2a** (0.2 mmol), Pd(OAc)_2_ (10 mol%), Ac-Gly-OH (20–60 mol%), oxidant (0.2–3.0 equiv), HFIP (1 ml), 24 h, 80 °C, air or O_2_. Yield was determined by ^1^H NMR with CH_2_Br_2_ as internal standard. (mono/di) denotes the ratio of mono-*meta*-olefininated and di-(*meta,meta'*)-olefinated products. The bold ‘entry 13' represents the optimal conditions for obtaining highest overall yield. (Note: (a) Although the mono versus di-olefination selectivity is not good, the isolated overall yield is best. (b) Although entry 8 led to higher overall yield, it does not use catalytic amount of Cu(OAc)_2_). The bold ‘entry 15' represents the conditions that result in highest overall yield with relatively good mono versus di-olefination selectivity.

^*^1a (0.2 mmol scale).

^†^Isolated yield.

^‡^No reaction.

^§^The reaction was run for 48 h.

^||^The reaction was run at 90 °C.

^¶^K_2_HPO_4_ (0.5 equiv) was added.

^#^KH_2_PO_4_ (0.5 equiv) was added.
