# Supplementary material for: Pd(II)-catalysed meta-C–H functionalizations of benzoic acid derivatives
Source: Nat Commun. 2016 Jan 27;7:10443. doi: 10.1038/ncomms10443 (PMC4737847; doi:10.1038/ncomms10443)
Supplement: Supplementary Information — Supplementary Figures 1-102, Supplementary Table 1, Supplementary Methods and Supplementary References [file ncomms10443-s1.pdf]

Supplementary Figures

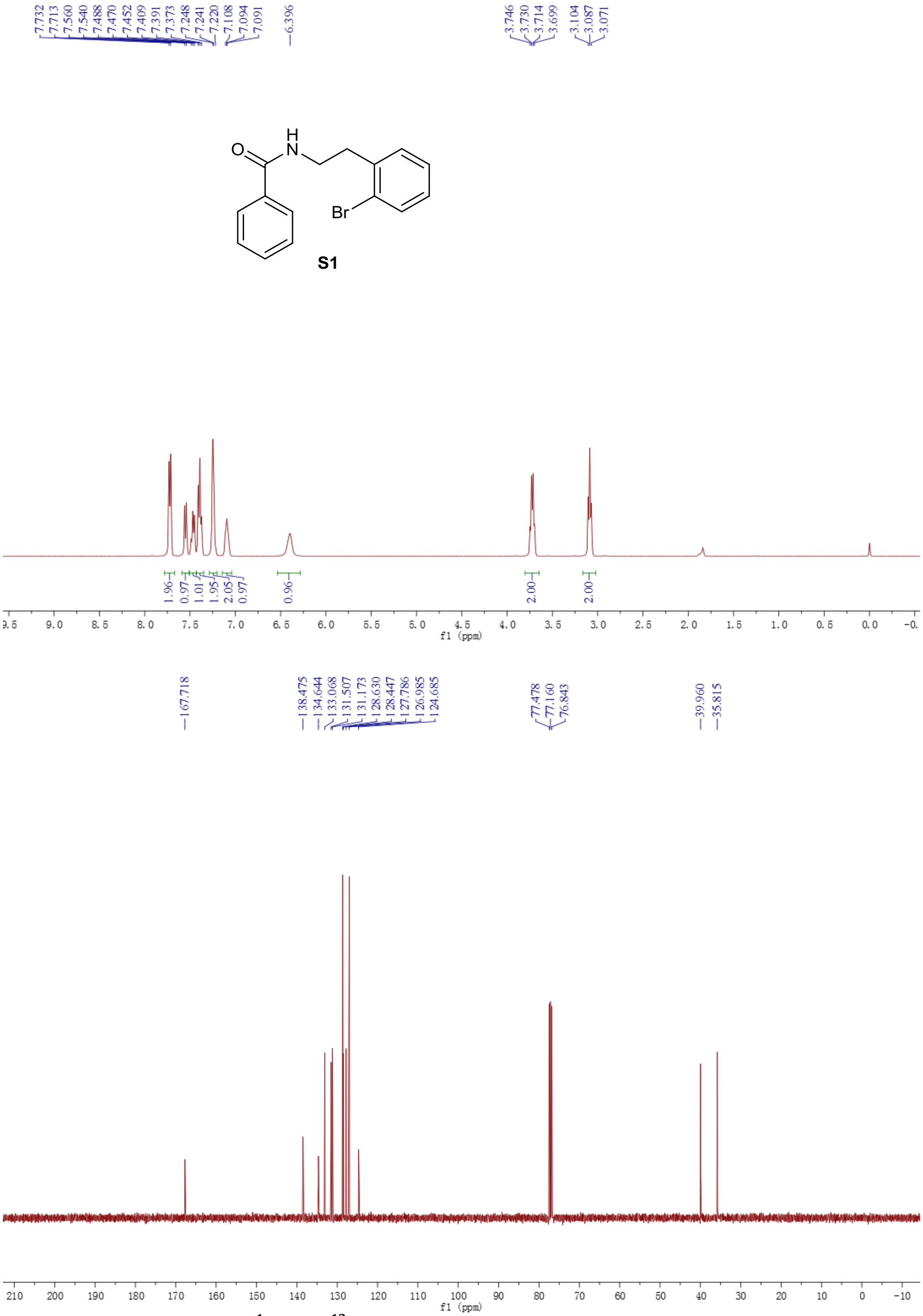

Supplementary Figure 1. ^{1}H and ^{13}C NMR spectra for S1

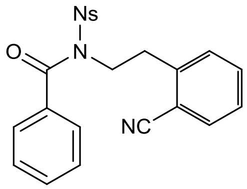

1a

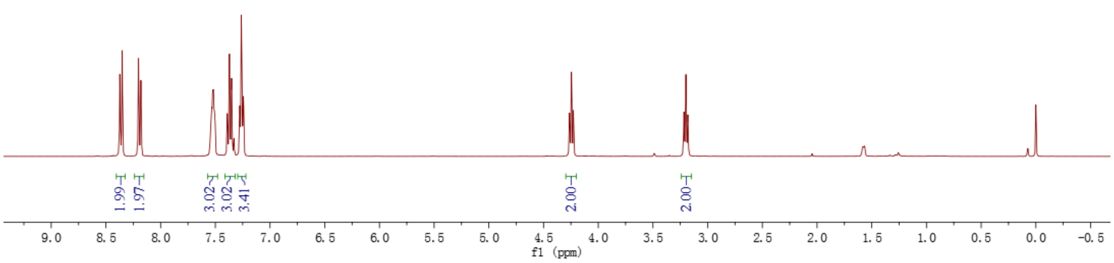

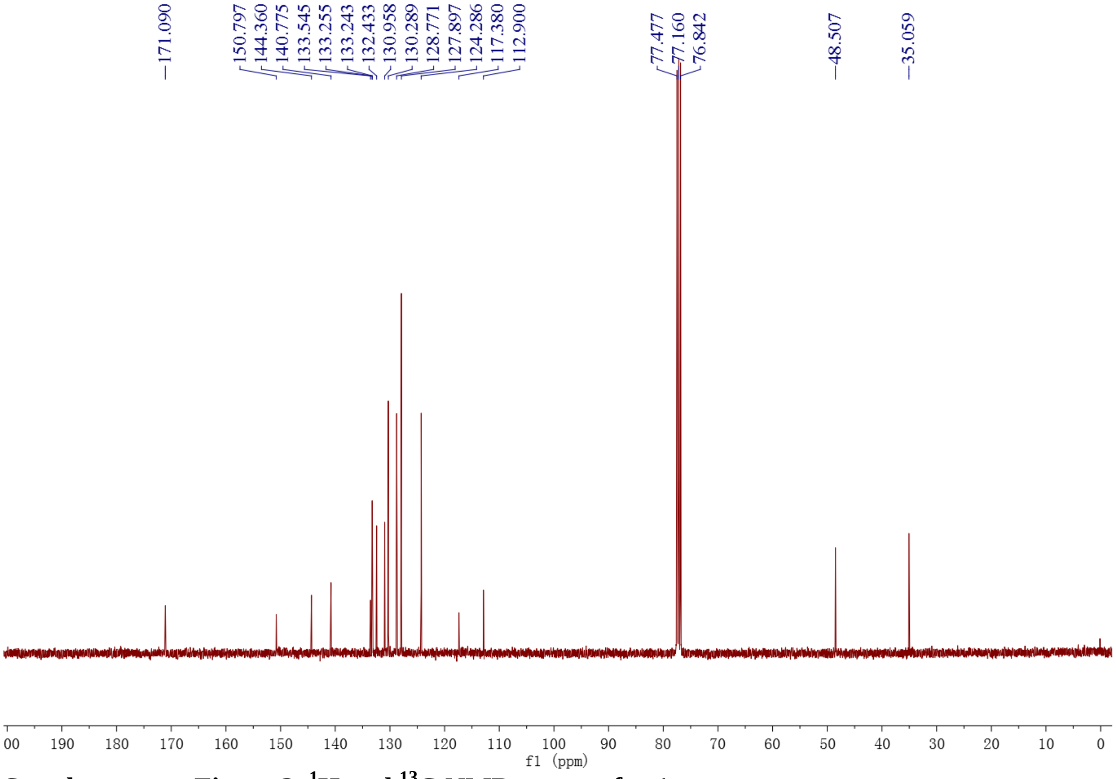

Supplementary Figure 2. ^{1}H and ^{13}C NMR spectra for 1a

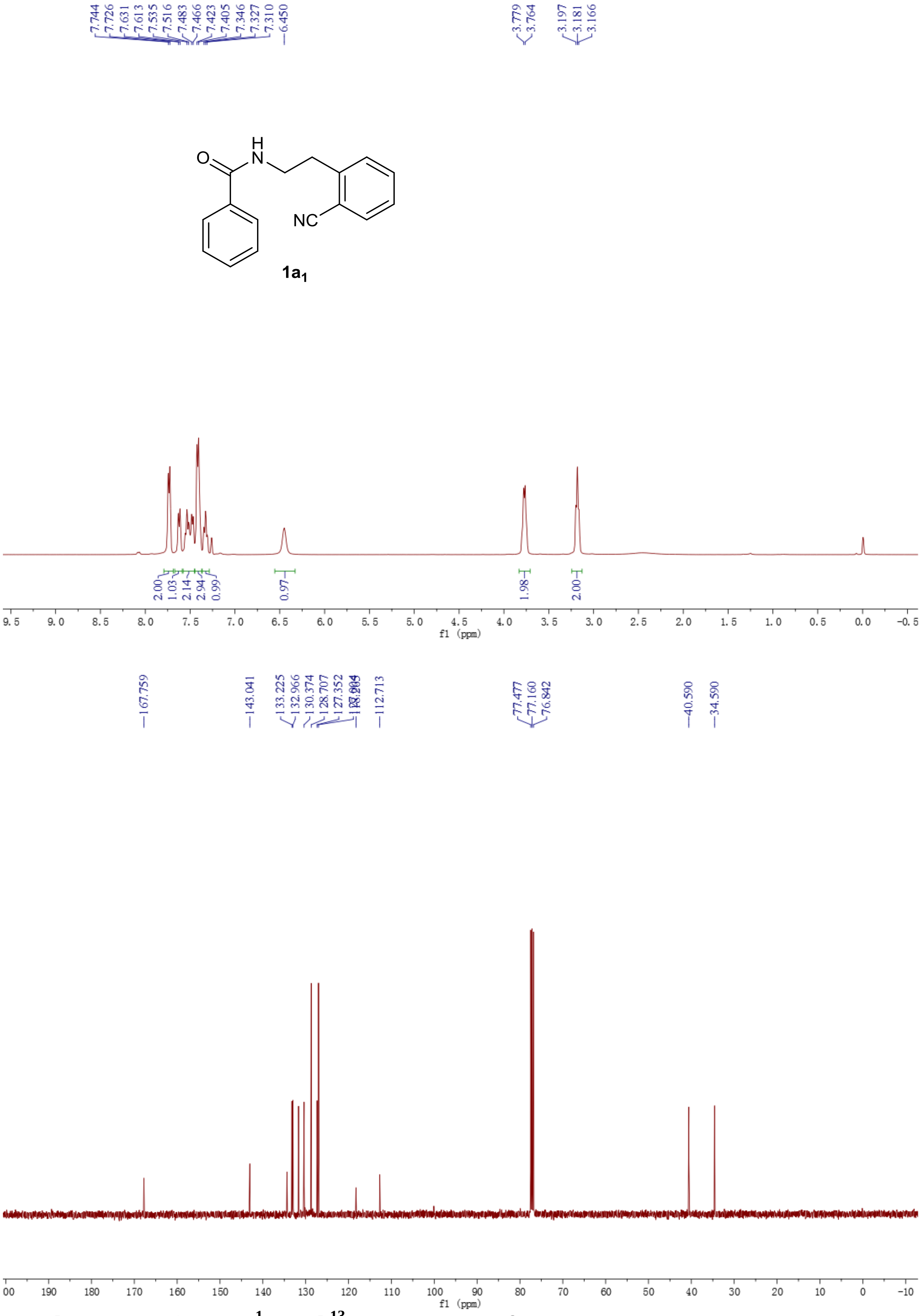

Supplementary Figure 3. ^{1}H and ^{13}C NMR spectra for 1a$_1$

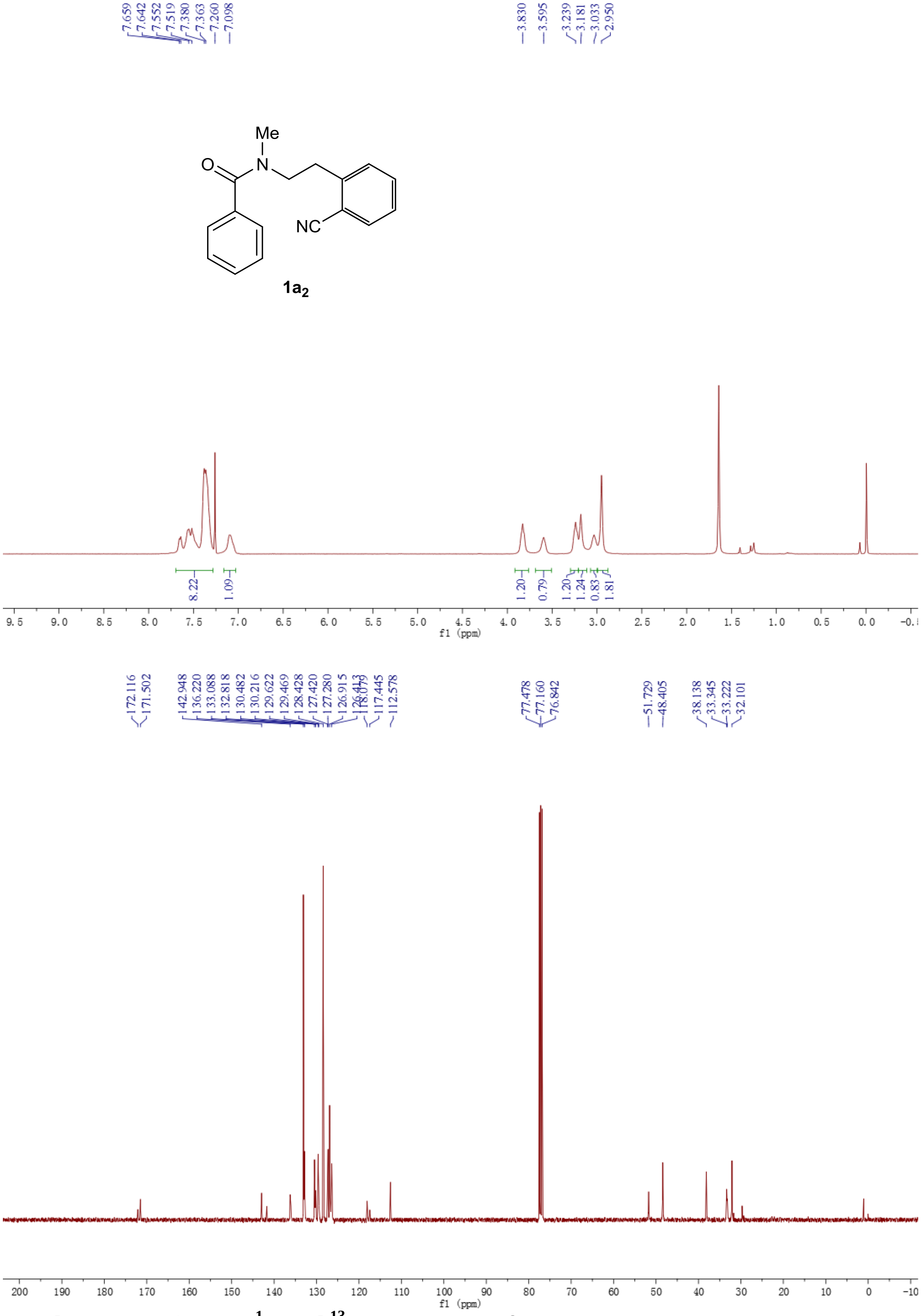

Supplementary Figure 4. ^{1}H and ^{13}C NMR spectra for 1a$_2$

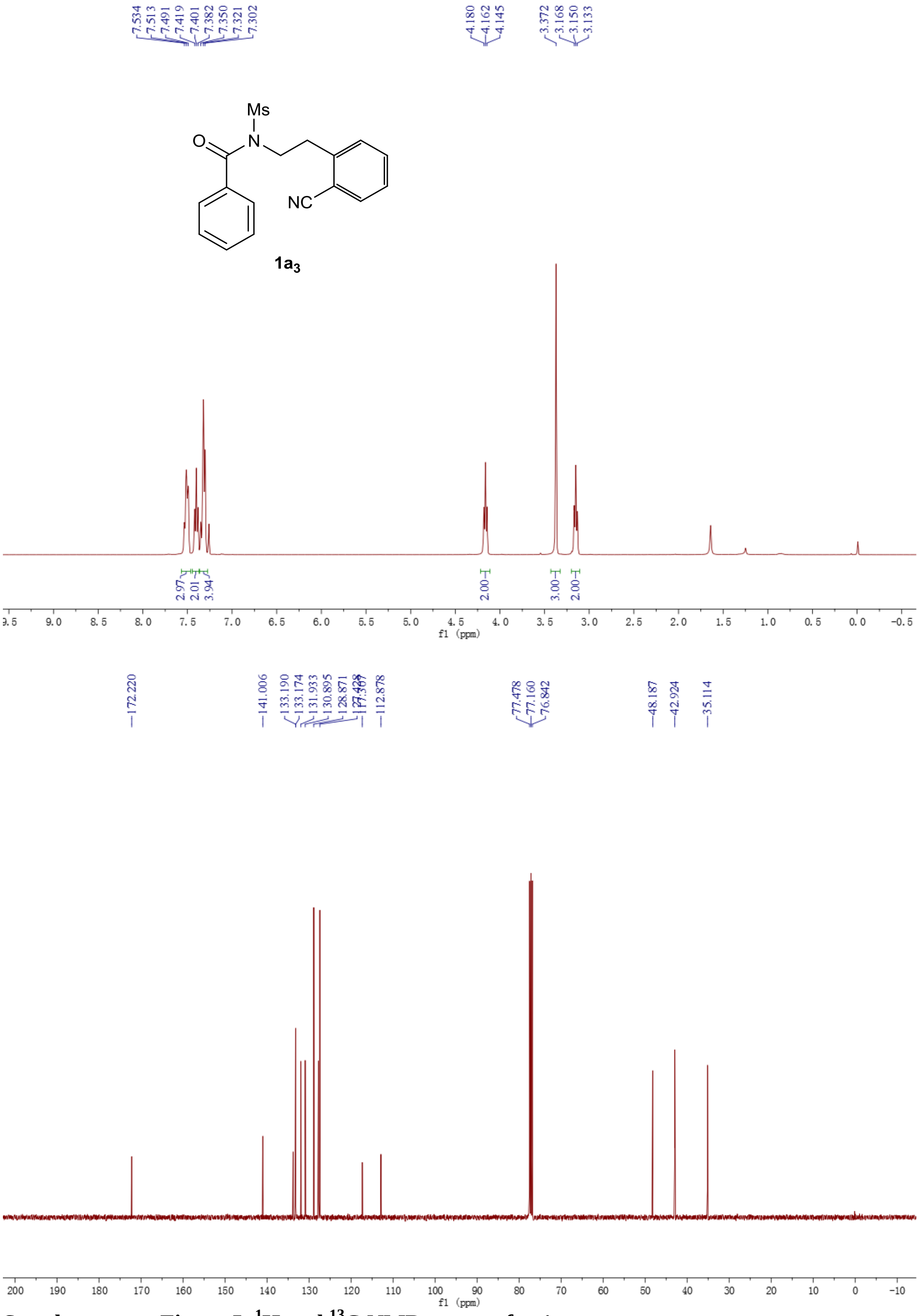

Supplementary Figure 5. ^{1}H and ^{13}C NMR spectra for 1a$_3$

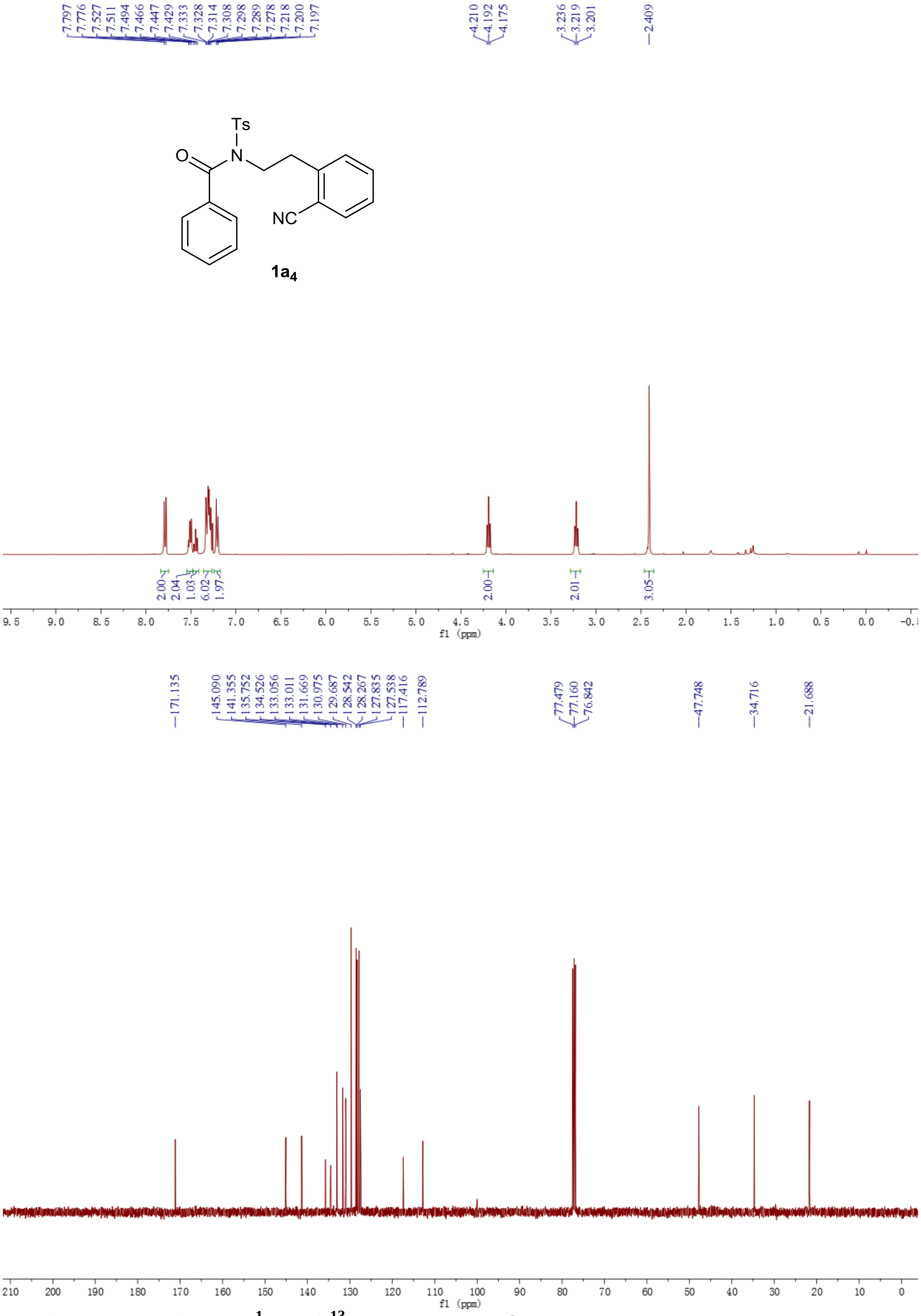

Supplementary Figure 6. ^{1}H and ^{13}C NMR spectra for 1a$_4$

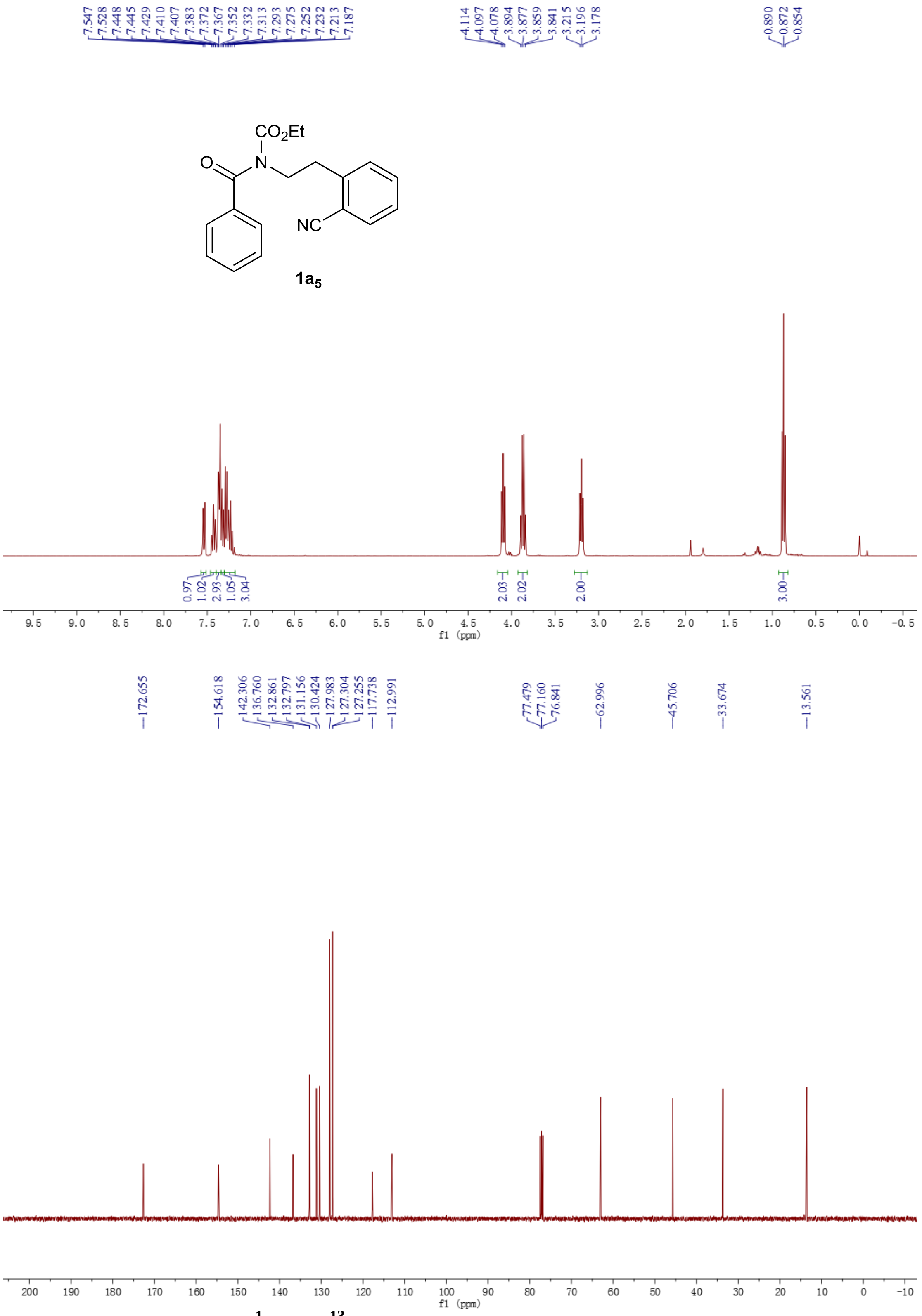

Supplementary Figure 7. ^{1}H and ^{13}C NMR spectra for $1a_5$

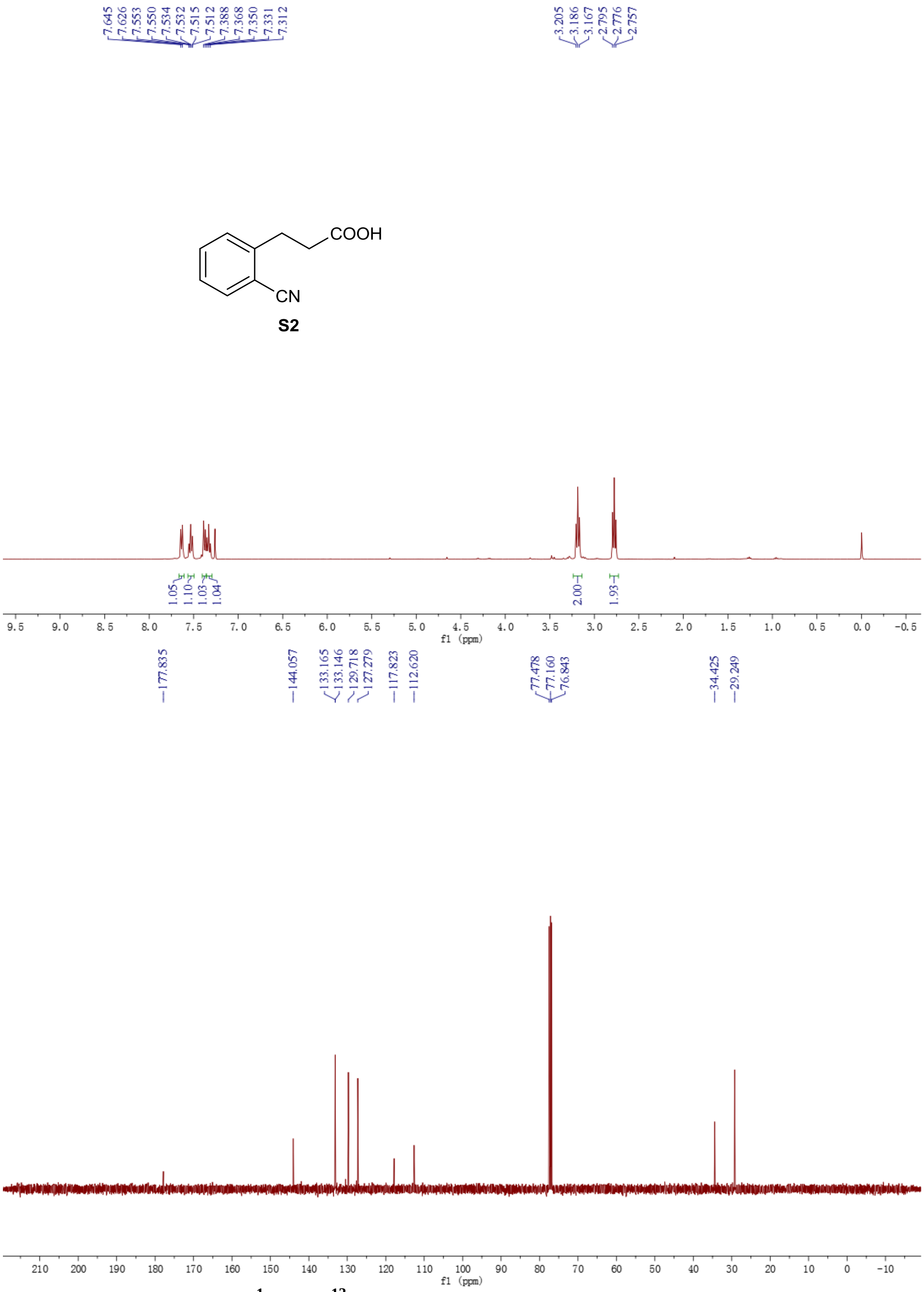

Supplementary Figure 8. ^{1}H and ^{13}C NMR spectra for S2

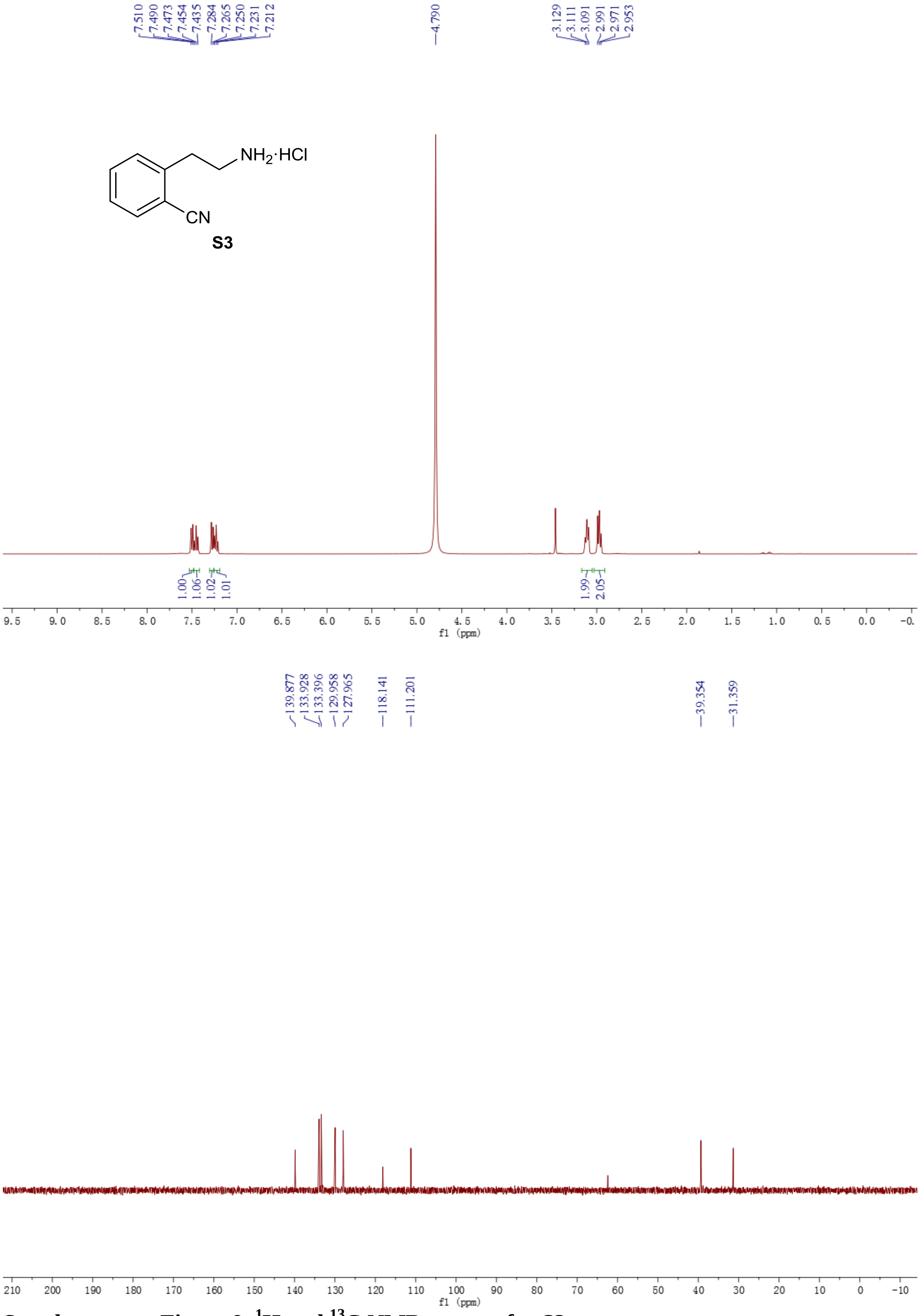

Supplementary Figure 9. ^{1}H and ^{13}C NMR spectra for S3

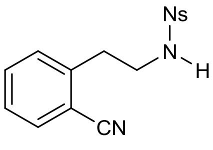

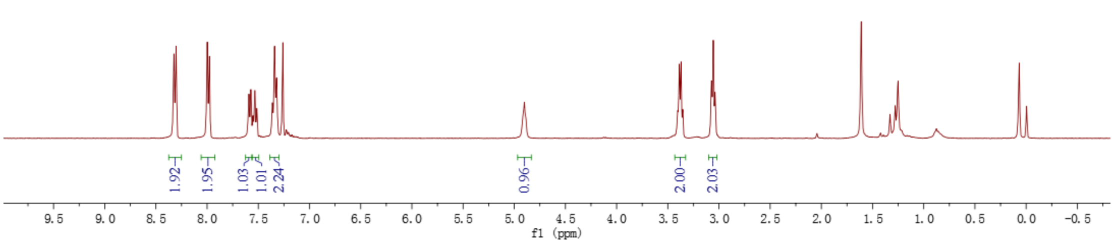

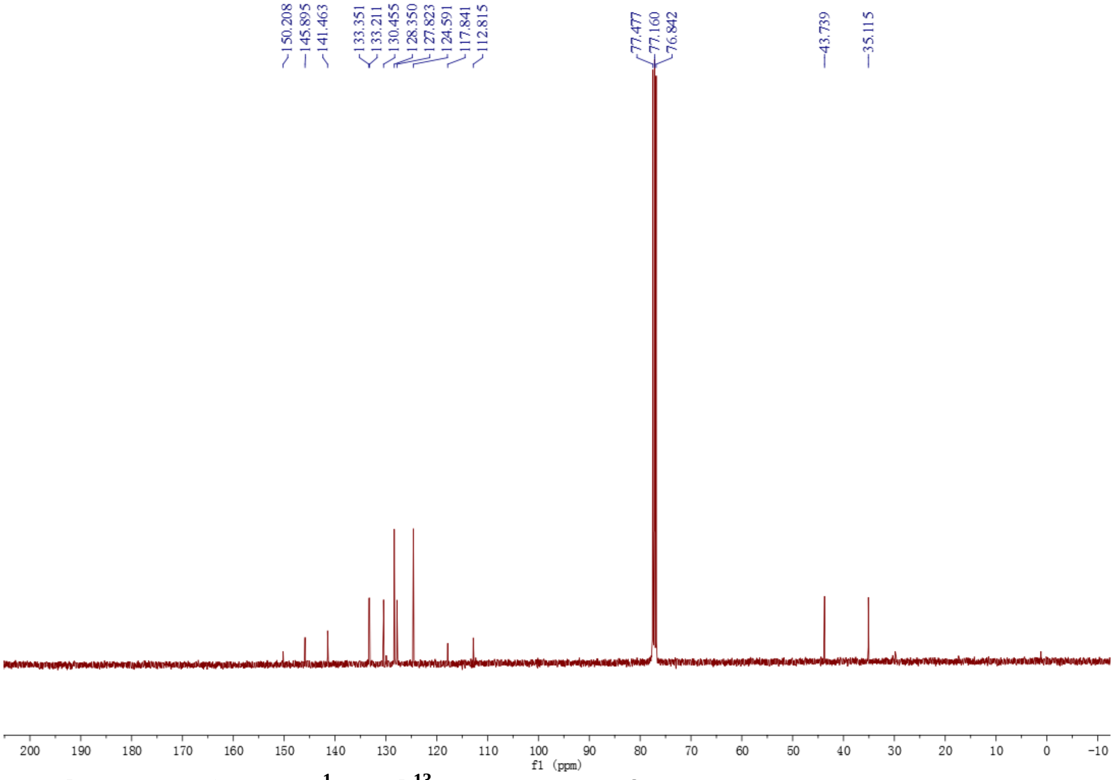

Supplementary Figure 10. ^{1}H and ^{13}C NMR spectra for 5

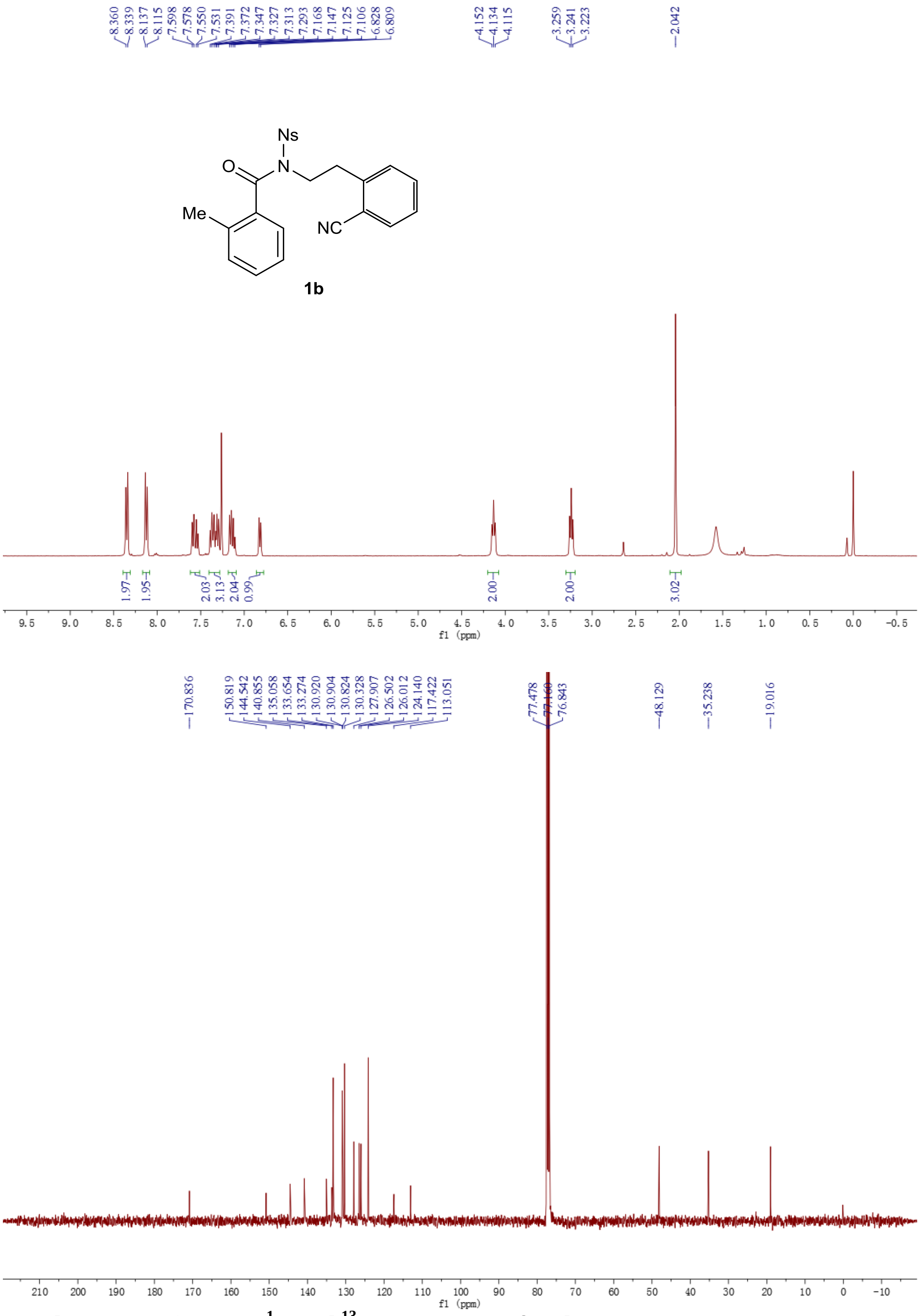

Supplementary Figure 11. ^{1}H **and** ^{13}C **NMR spectra for 1b**

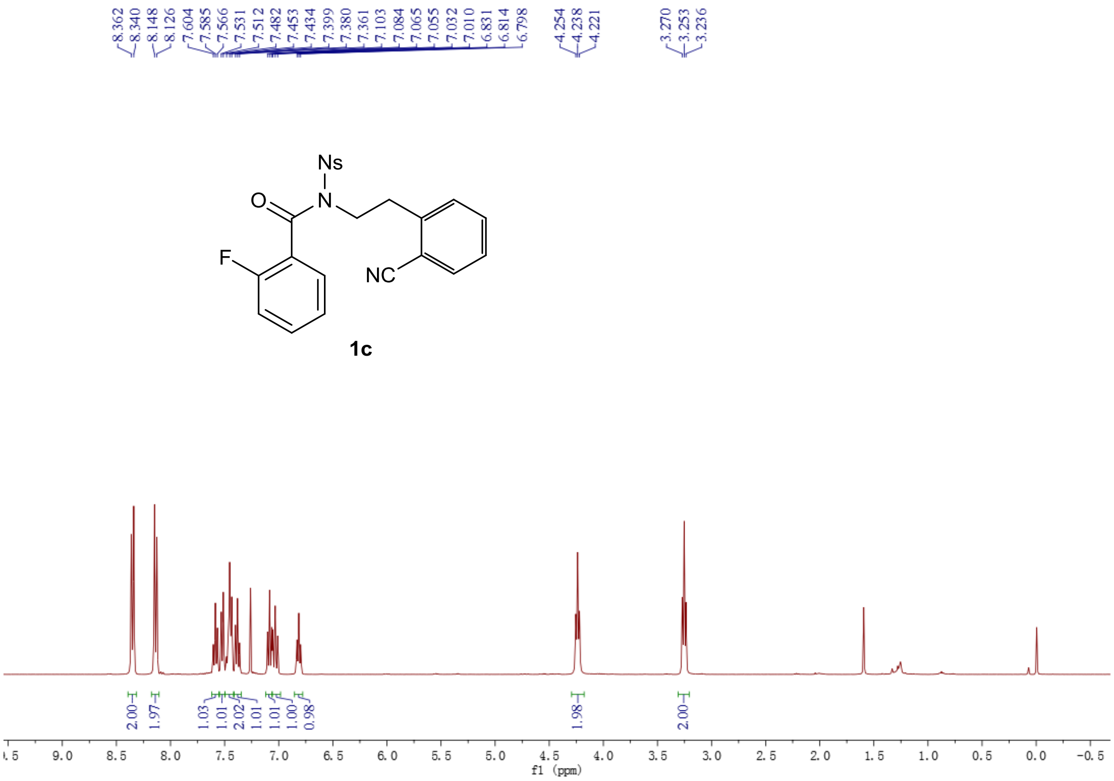

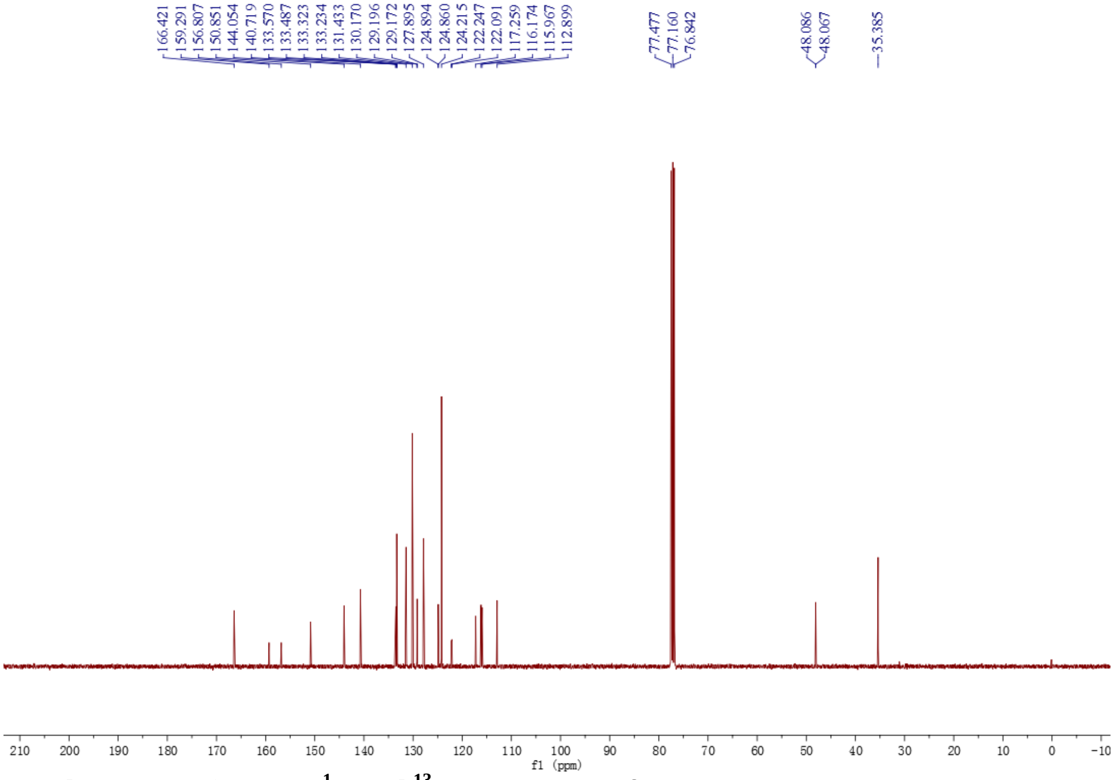

Supplementary Figure 12. ^{1}H and ^{13}C NMR spectra for 1c

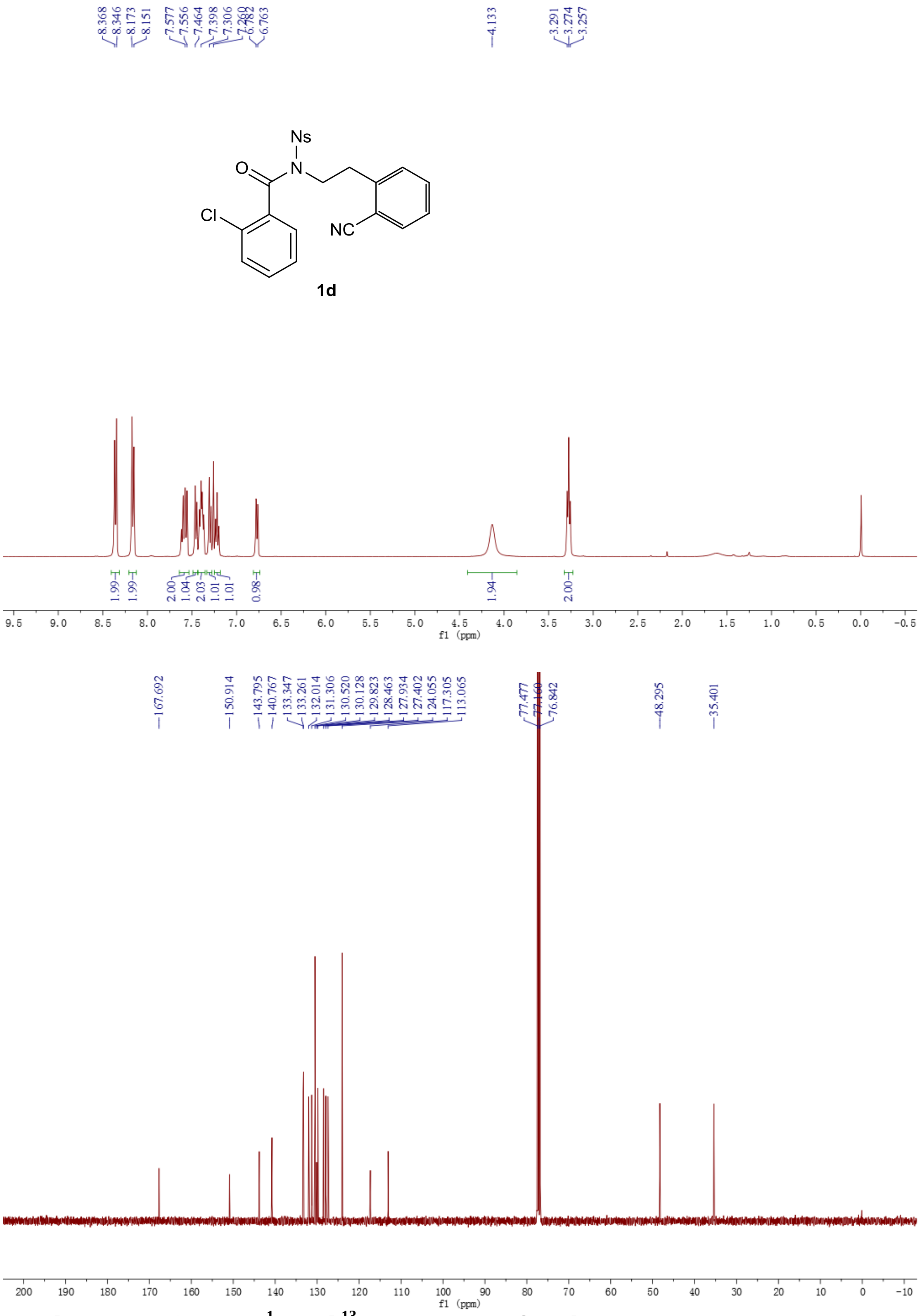

Supplementary Figure 13. ^{1}H and ^{13}C NMR spectra for 1d

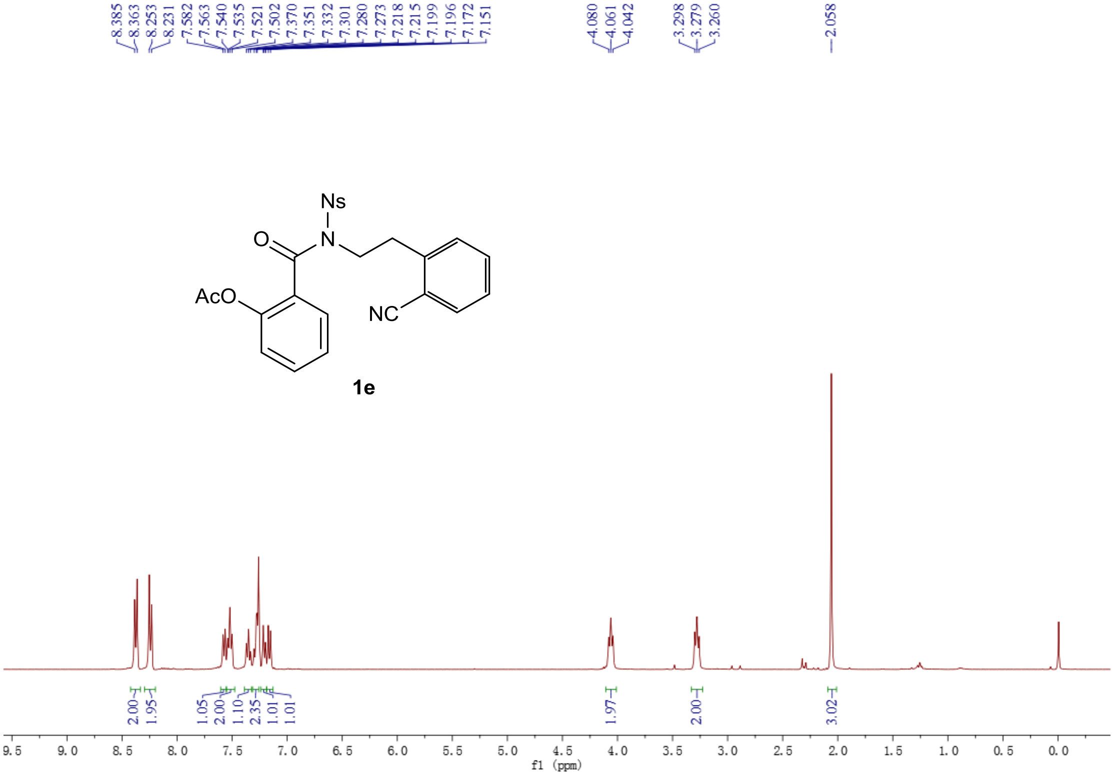

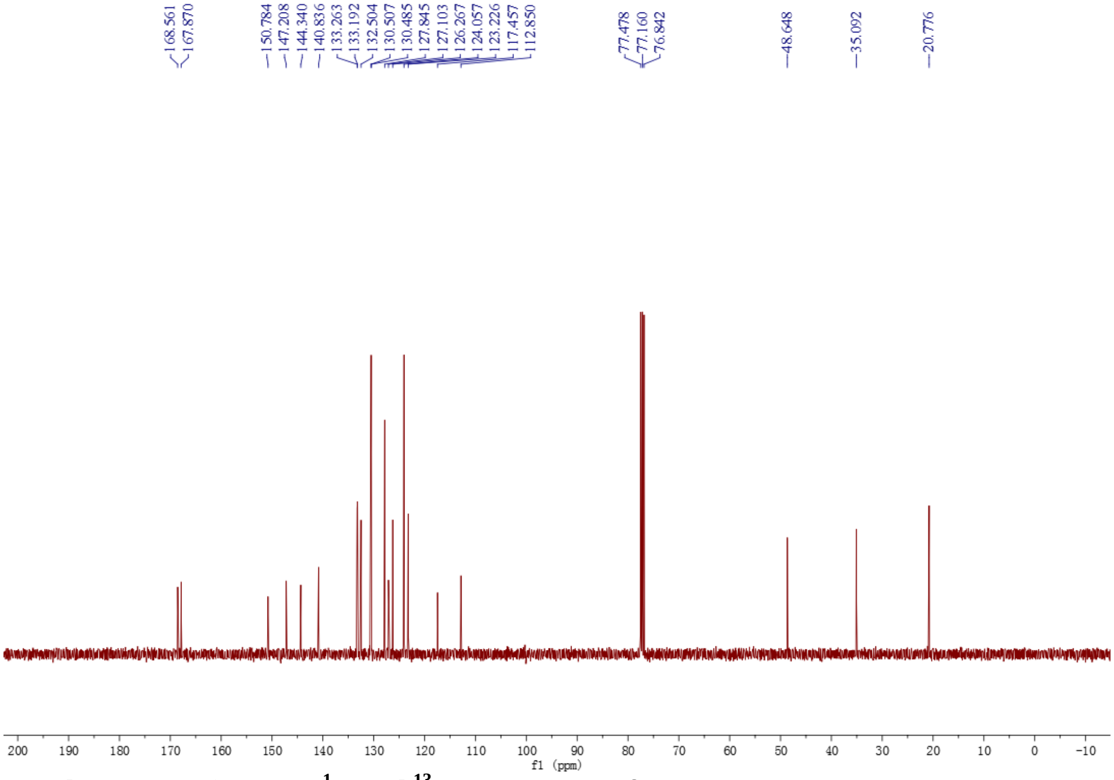

Supplementary Figure 14. ^{1}H and ^{13}C NMR spectra for 1e

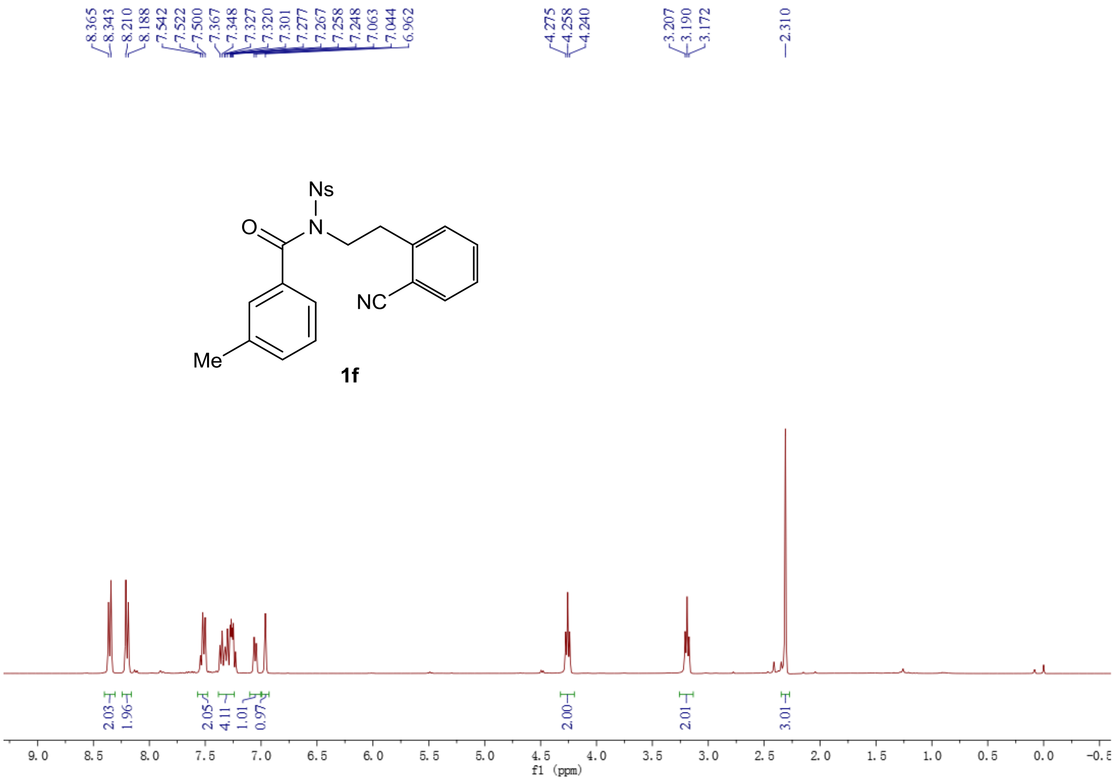

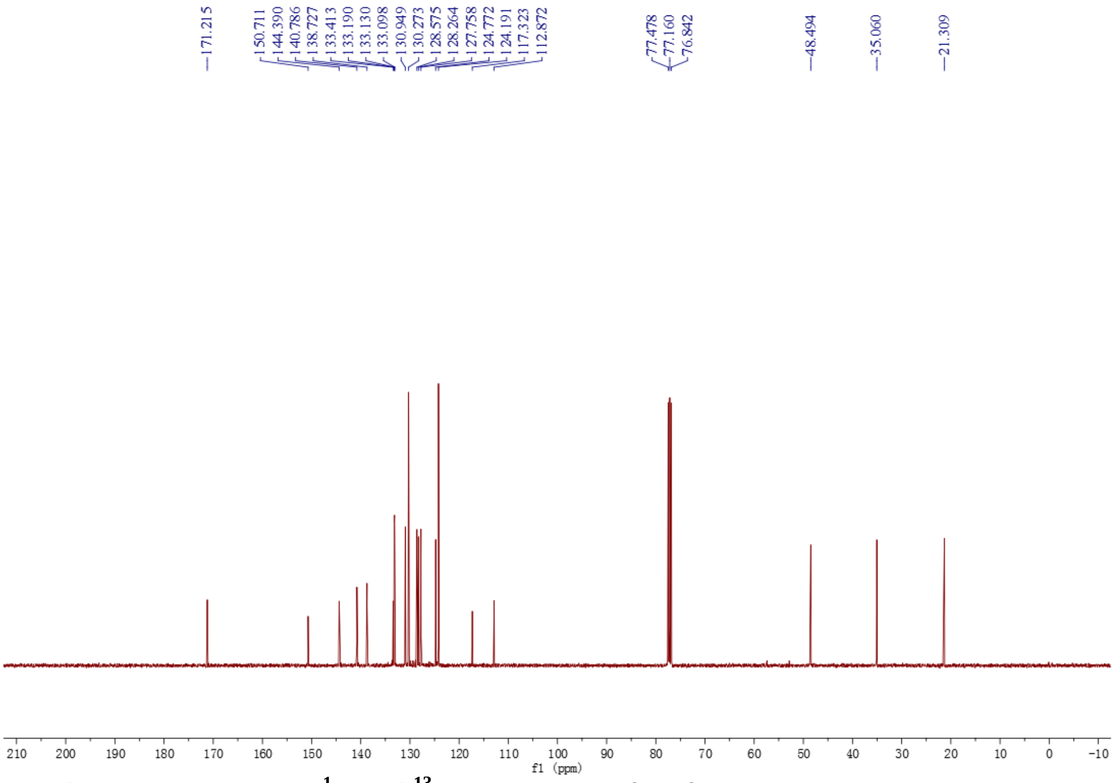

Supplementary Figure 15. ¹H and ¹³C NMR spectra for 1f

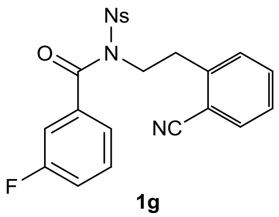

1g

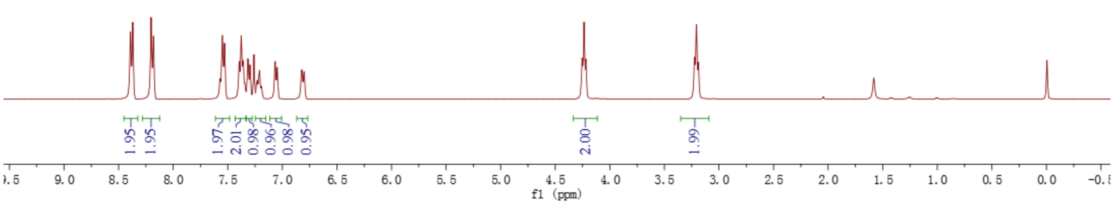

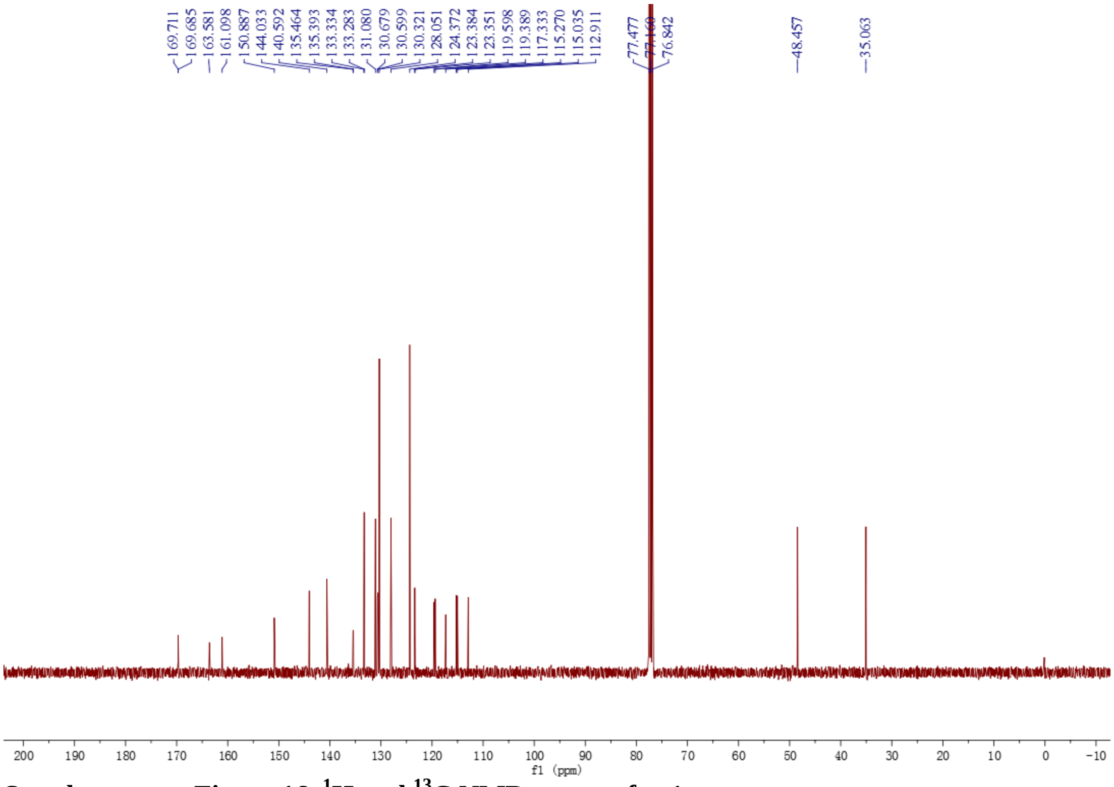

Supplementary Figure 16. ^{1}H and ^{13}C NMR spectra for 1g

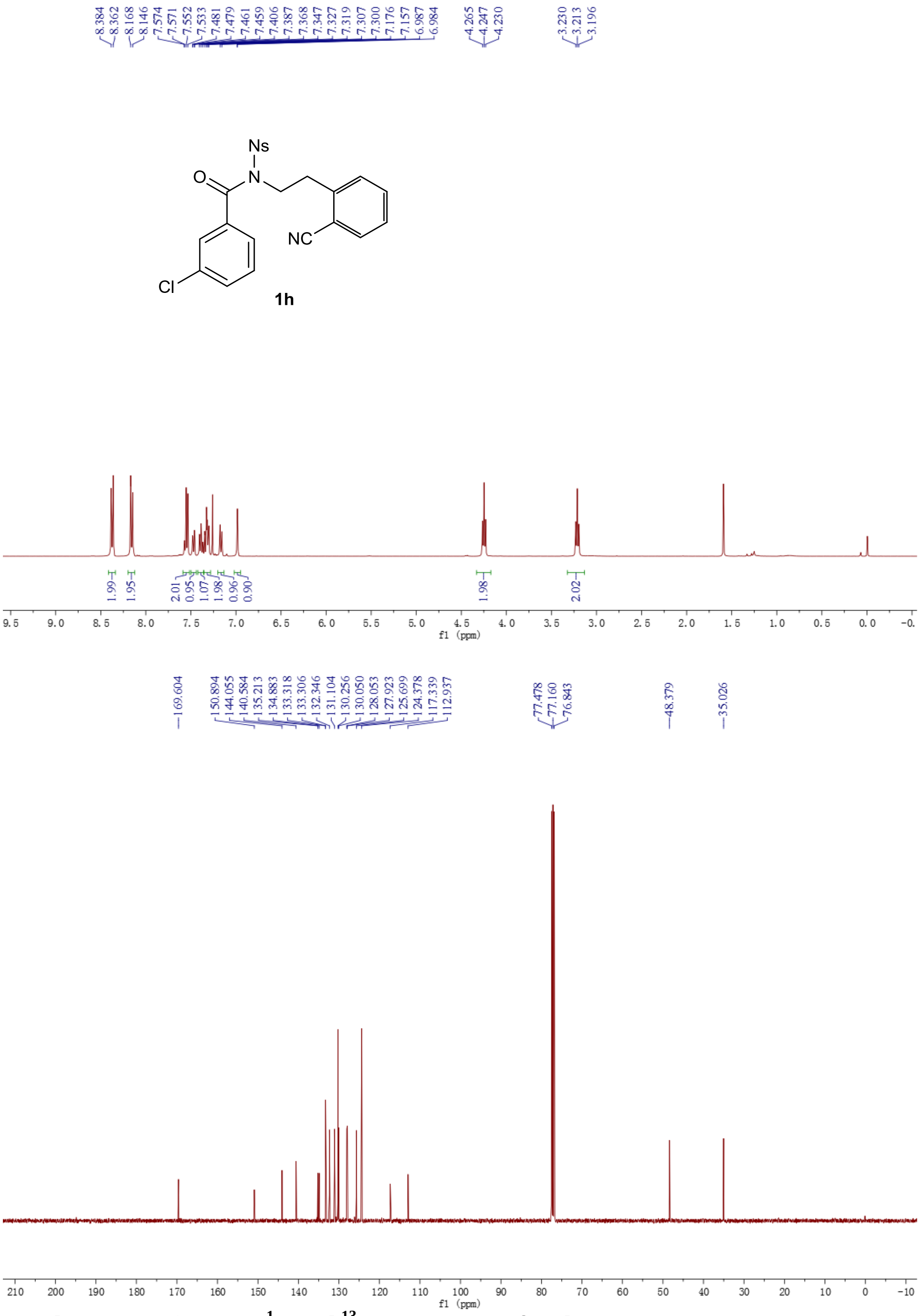

Supplementary Figure 17. ^{1}H and ^{13}C NMR spectra for 1h

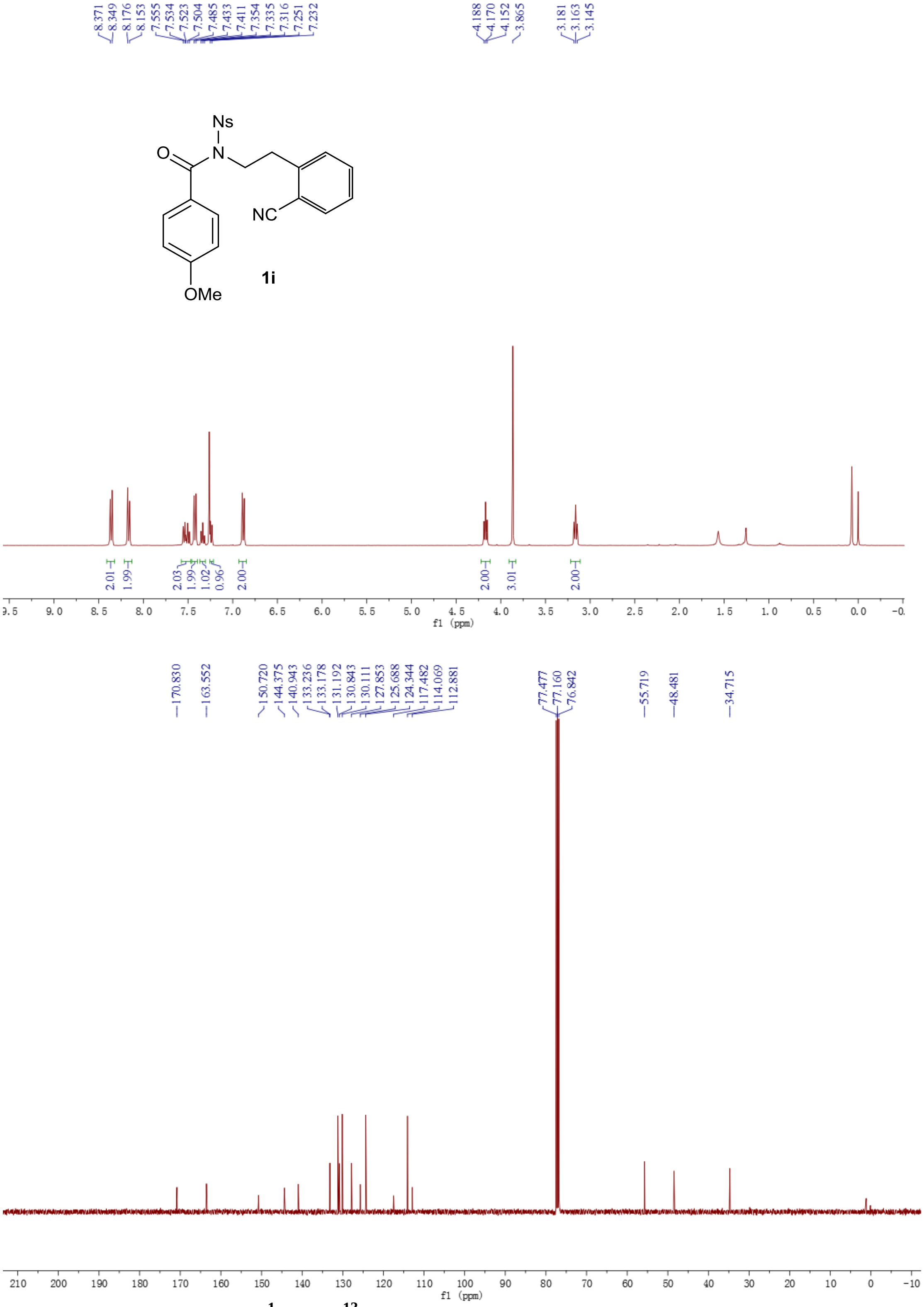

Supplementary Figure 18. ^{1}H and ^{13}C NMR spectra for 1i

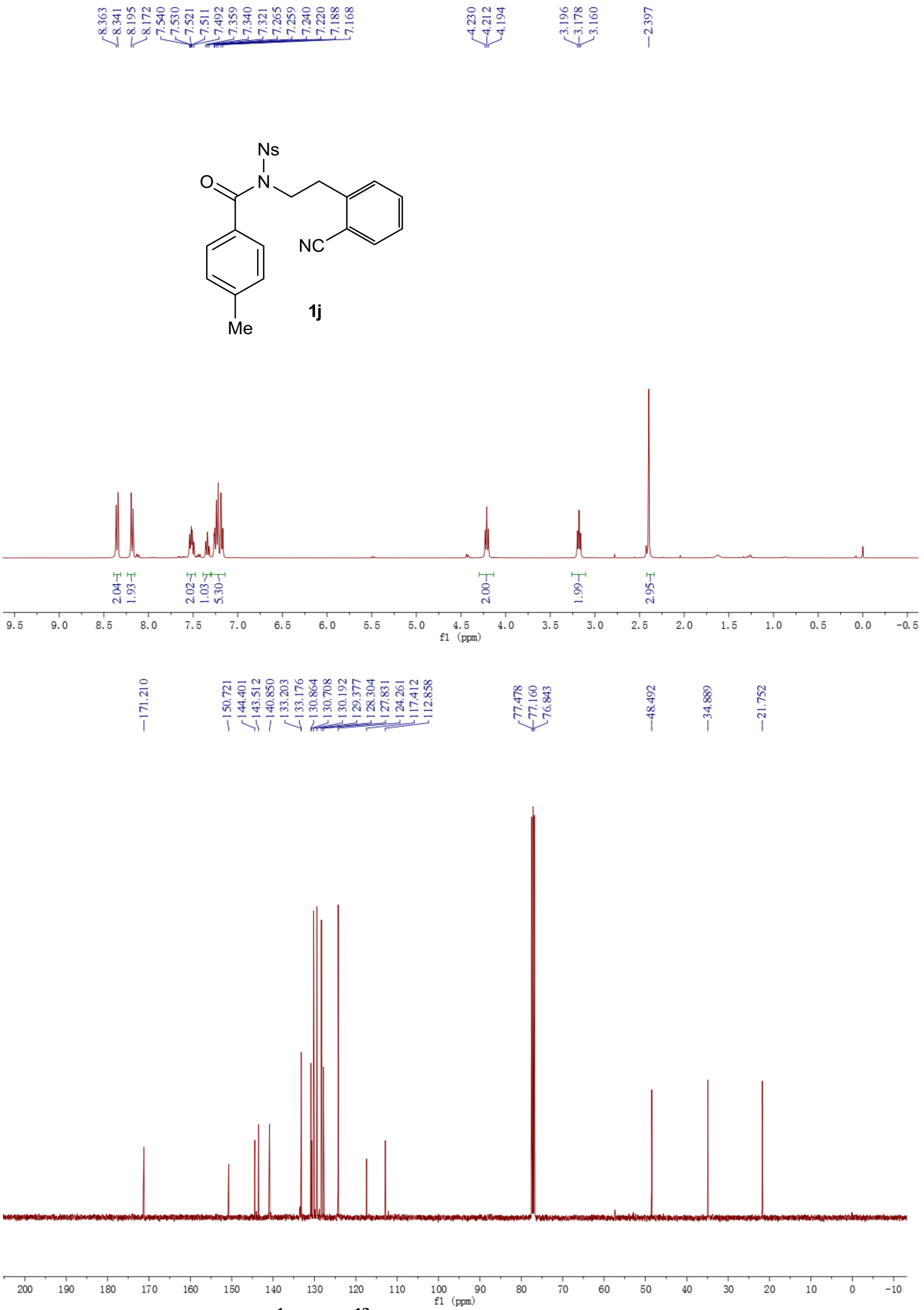

Supplementary Figure 19. ^{1}**H and** ^{13}**C NMR spectra for 1j**

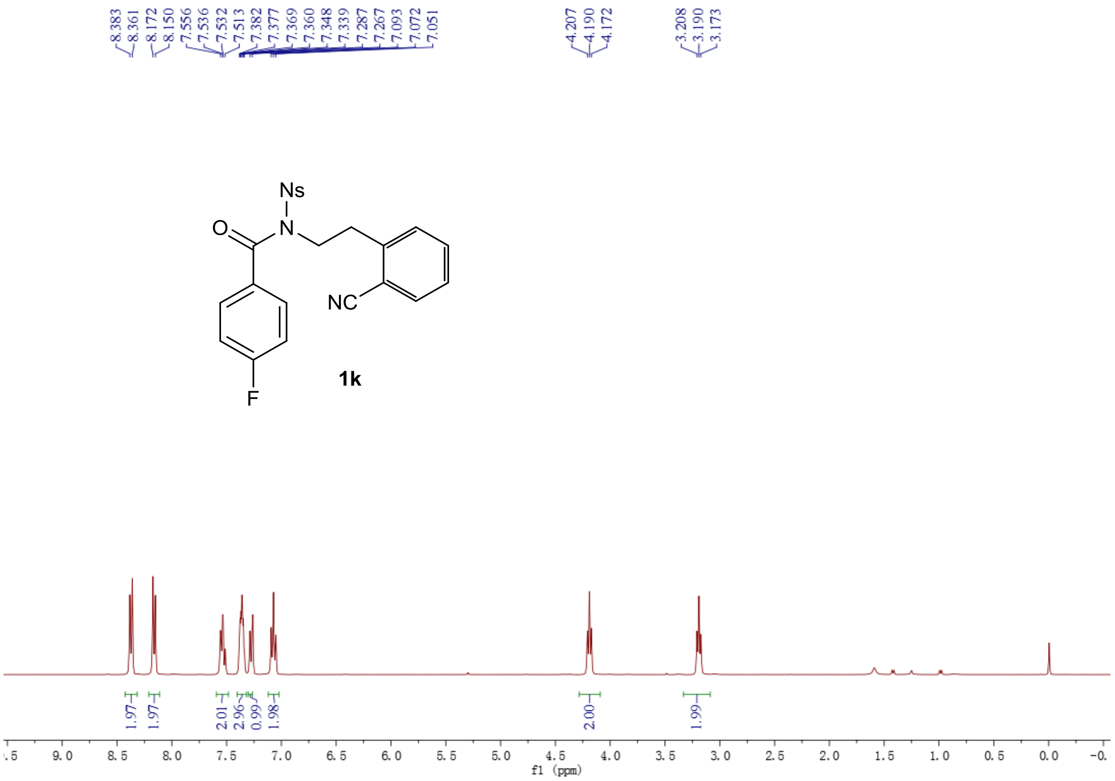

1k

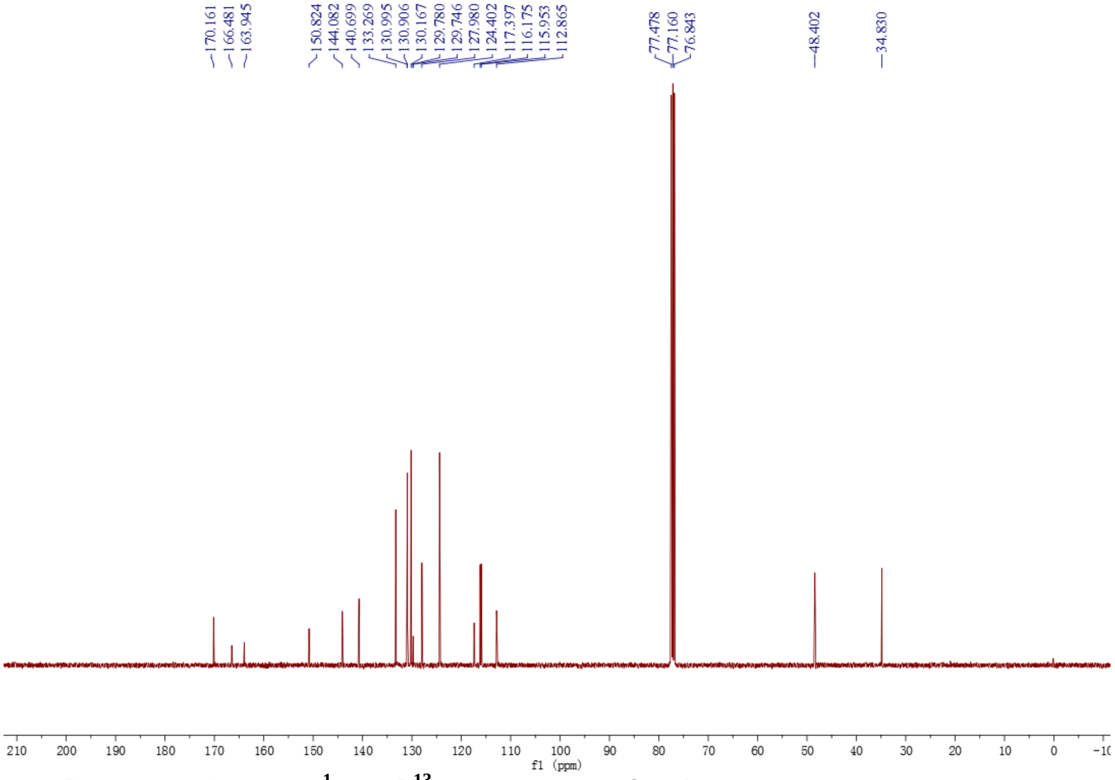

Supplementary Figure 20. ^{1}**H and** ^{13}**C NMR spectra for 1k**

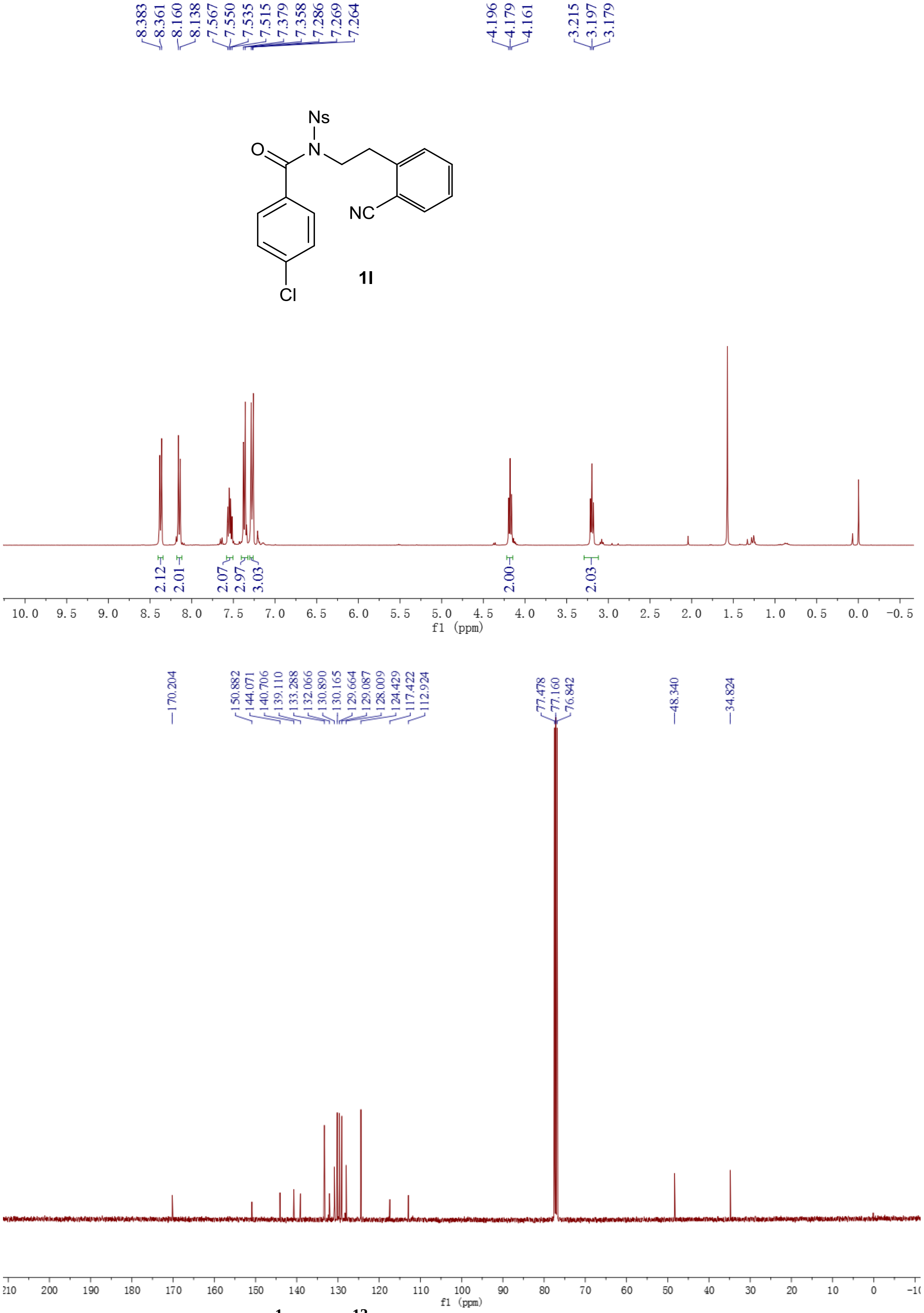

Supplementary Figure 21. ^{1}H and ^{13}C NMR spectra for 1l

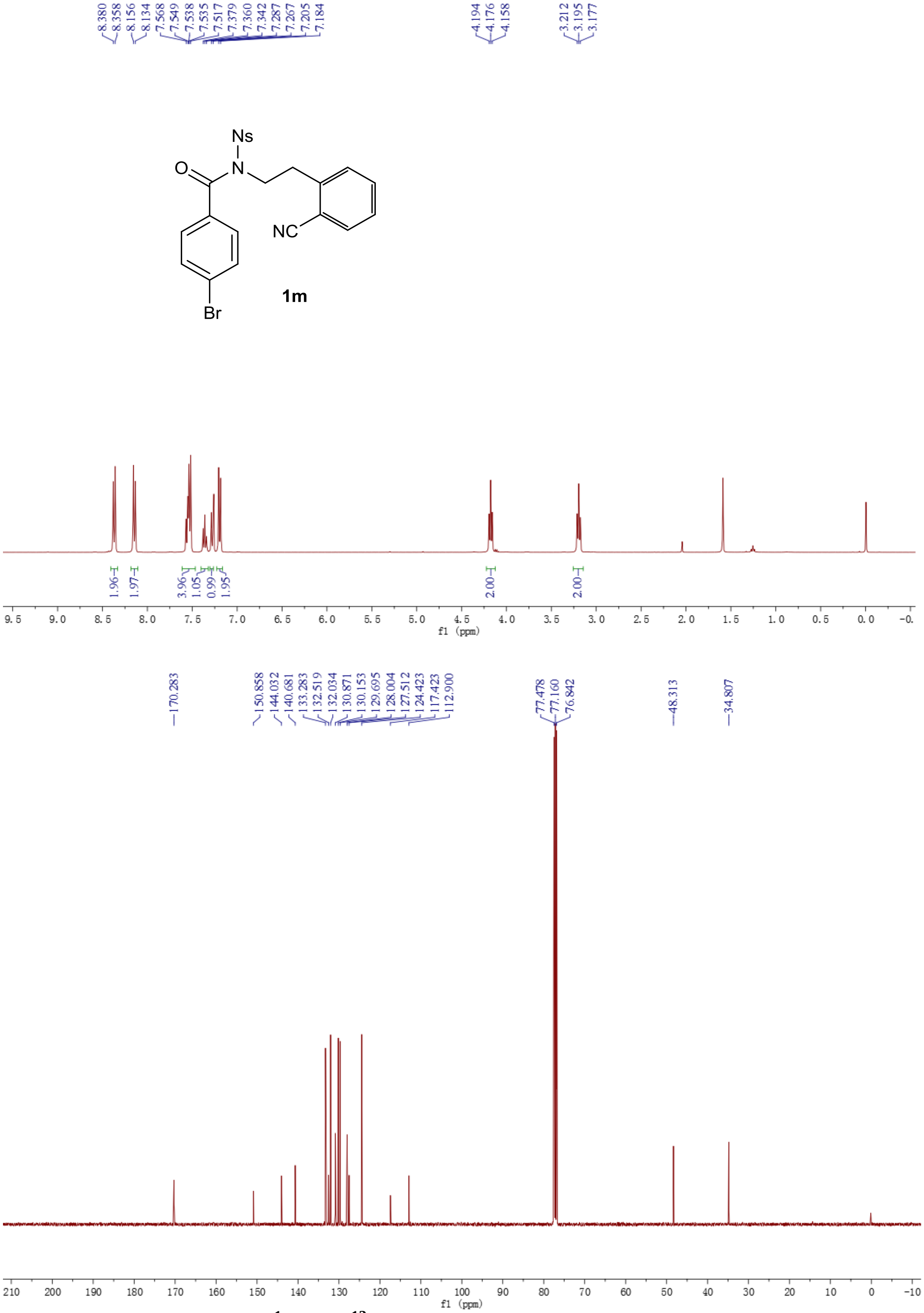

Supplementary Figure 22. ^{1}H and ^{13}C NMR spectra for 1m

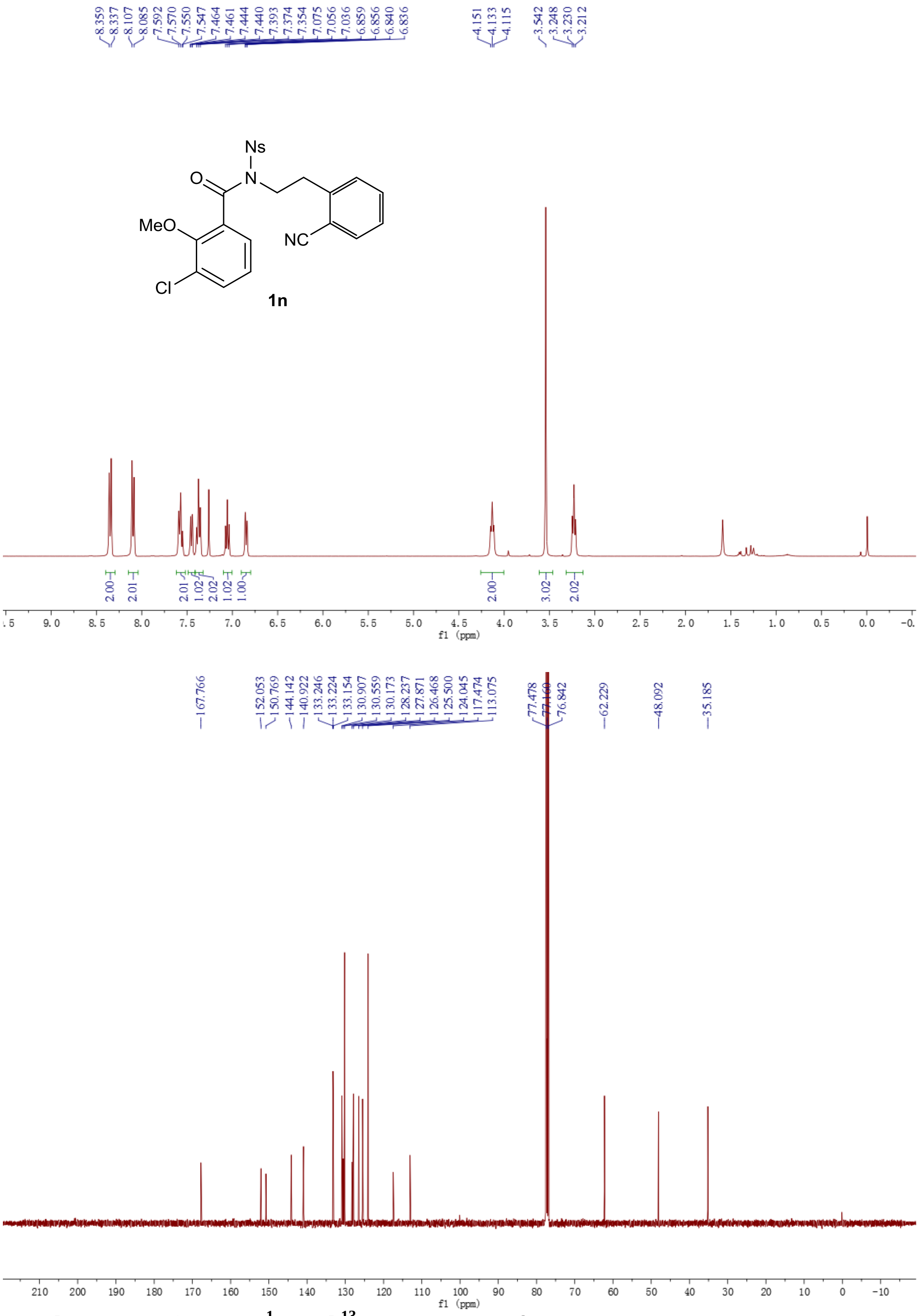

Supplementary Figure 23. ^{1}H and ^{13}C NMR spectra for 1n

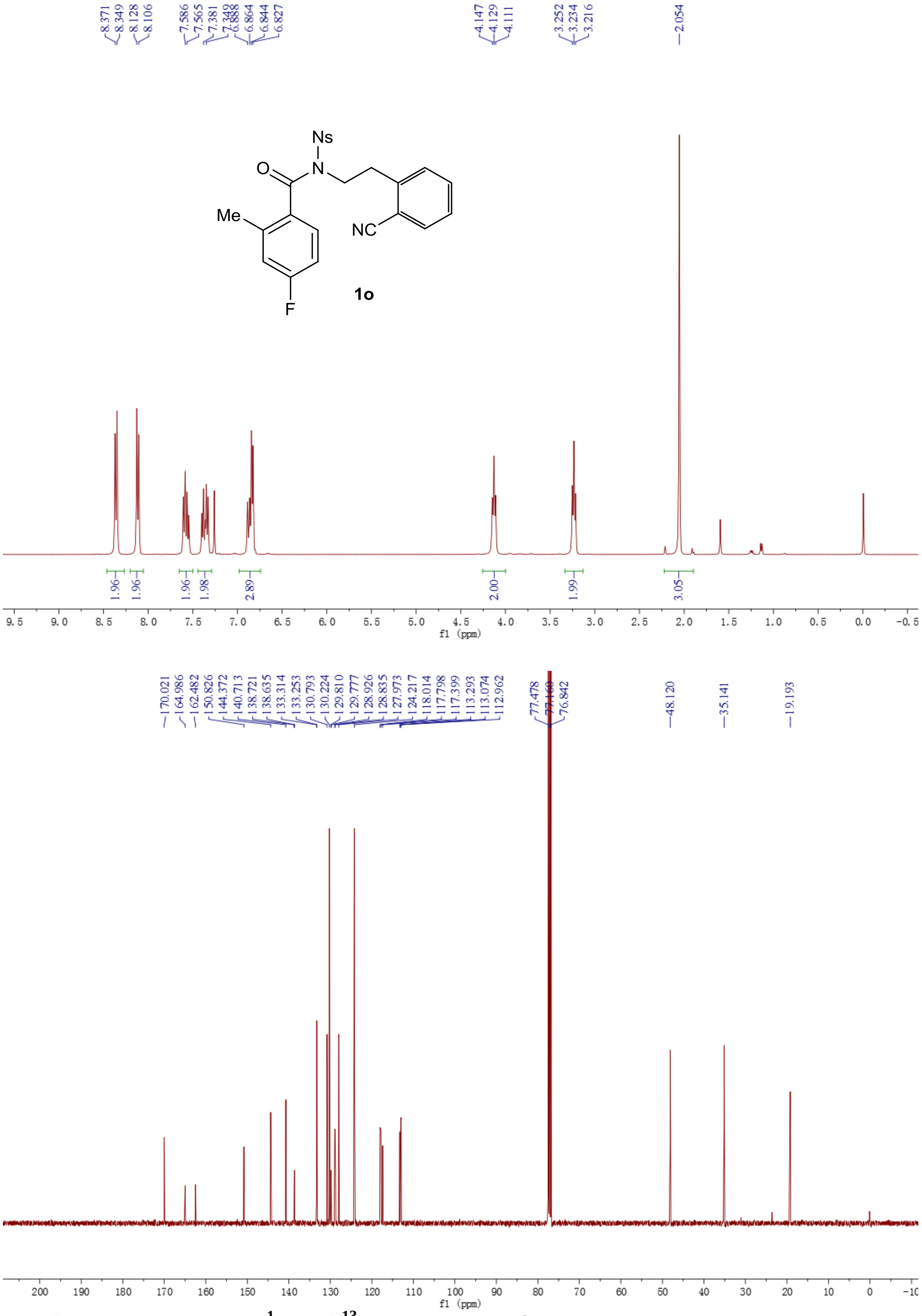

Supplementary Figure 24. ^{1}H and ^{13}C NMR spectra for 1o

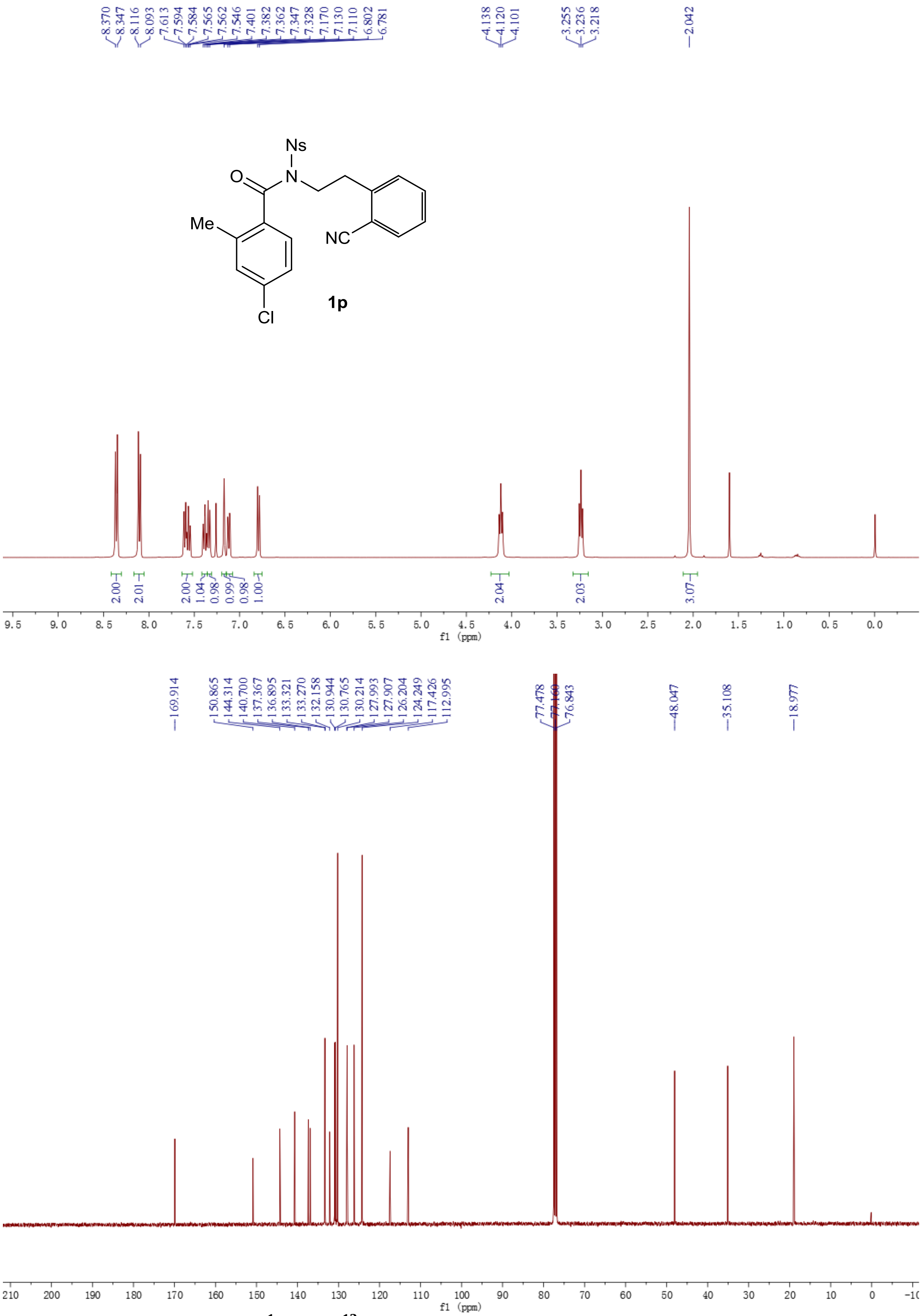

Supplementary Figure 25. ^{1}H and ^{13}C NMR spectra for 1p

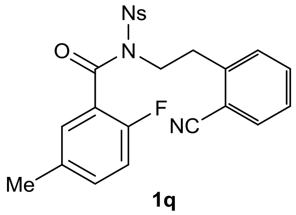

1q

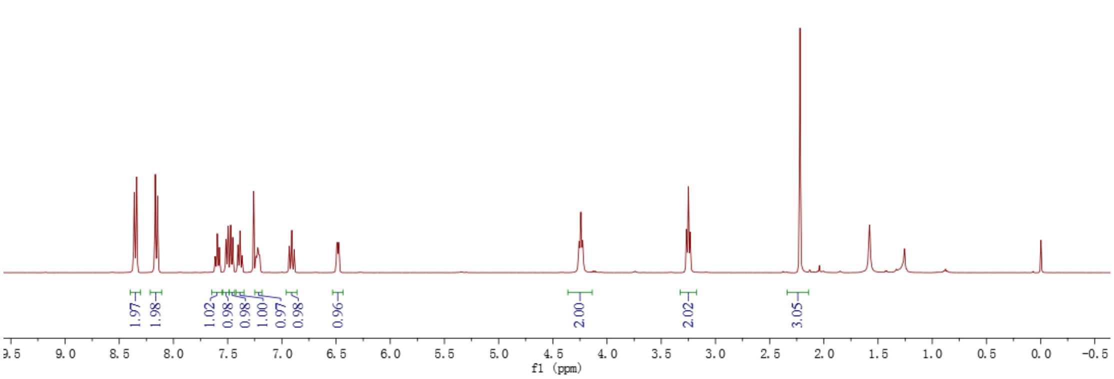

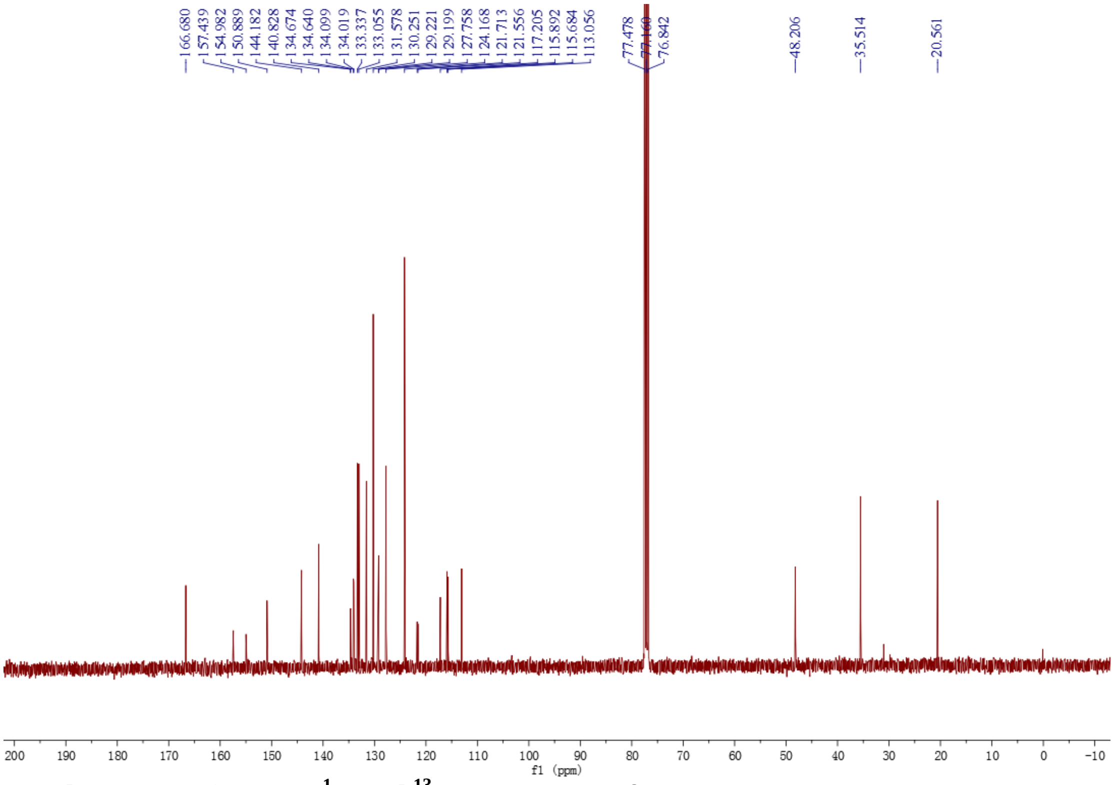

Supplementary Figure 26. ^{1}H and ^{13}C NMR spectra for 1q

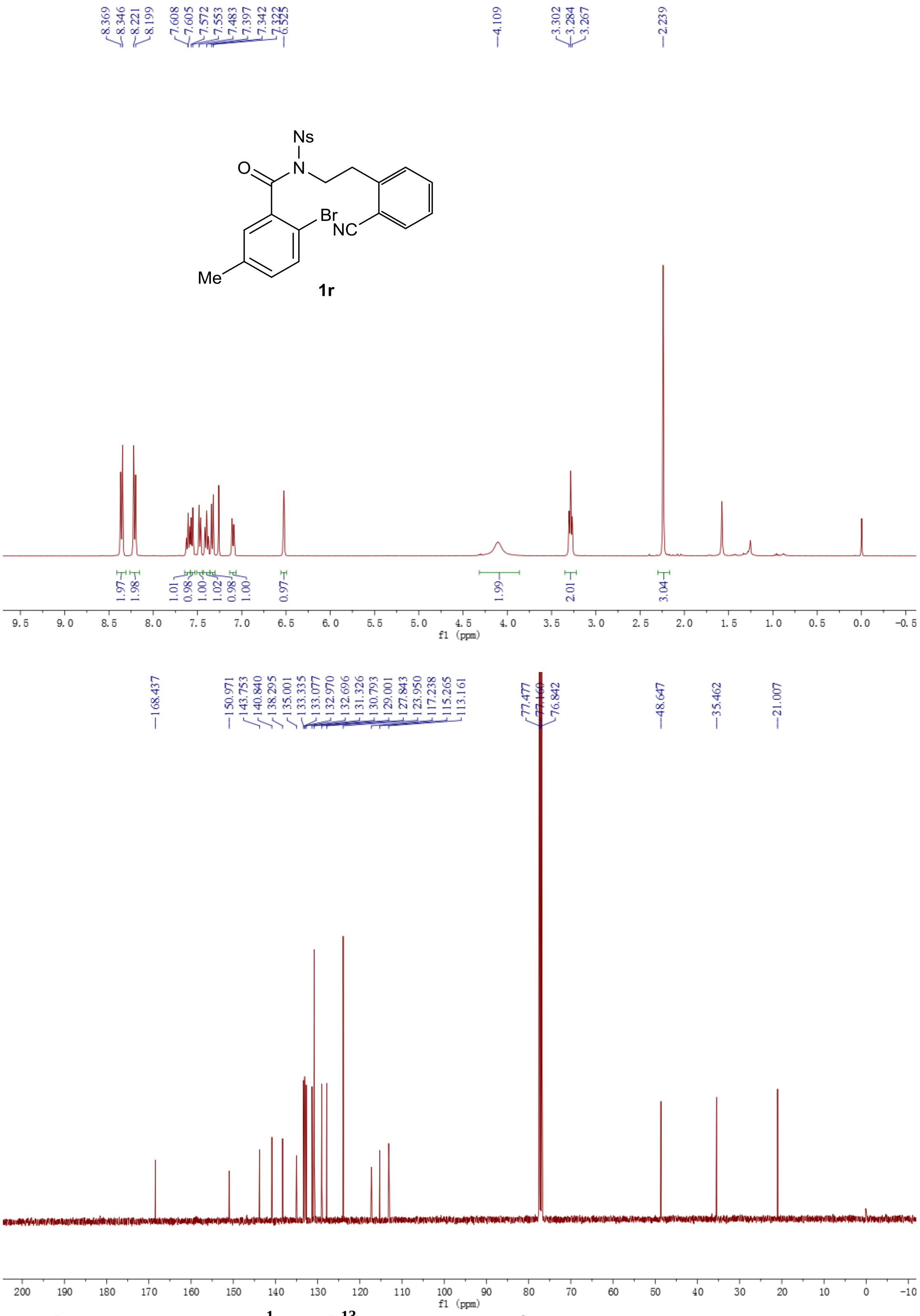

Supplementary Figure 27. ^{1}H and ^{13}C NMR spectra for 1r

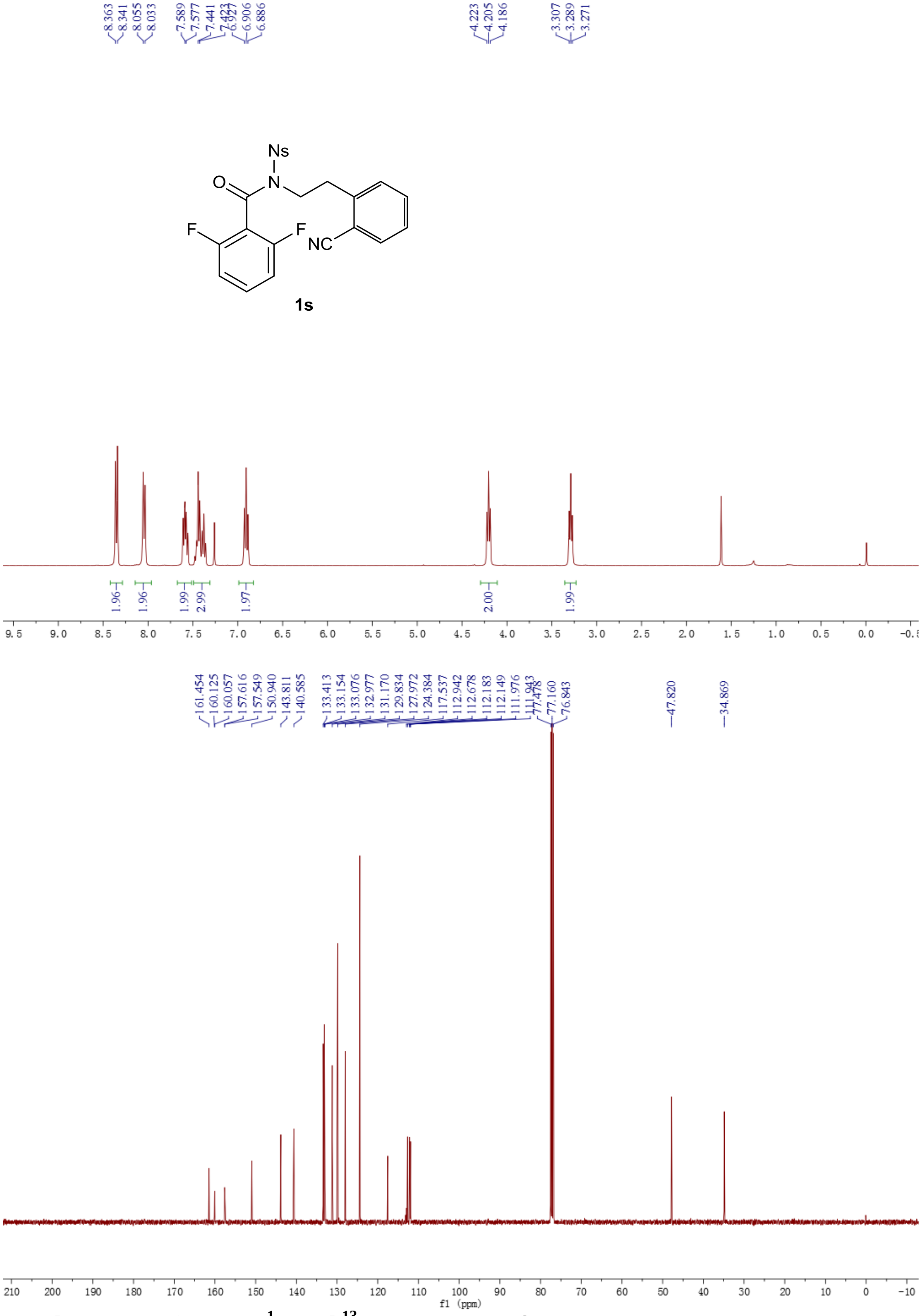

Supplementary Figure 28. ^{1}H and ^{13}C NMR spectra for 1s

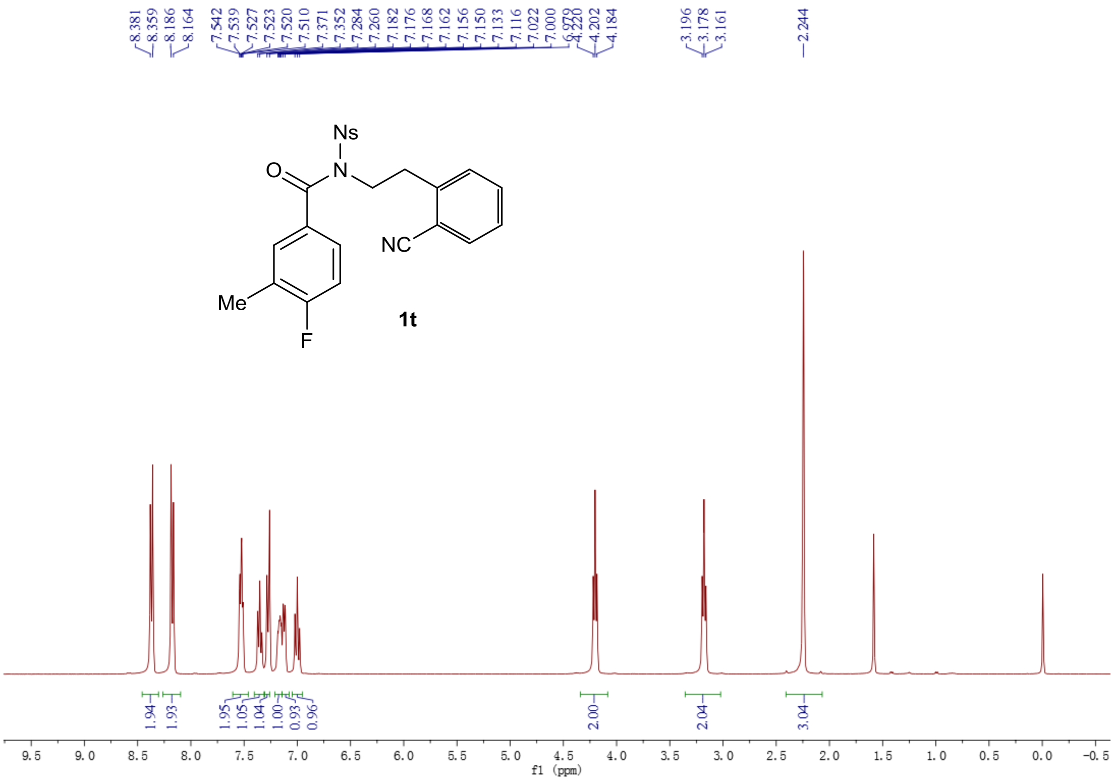

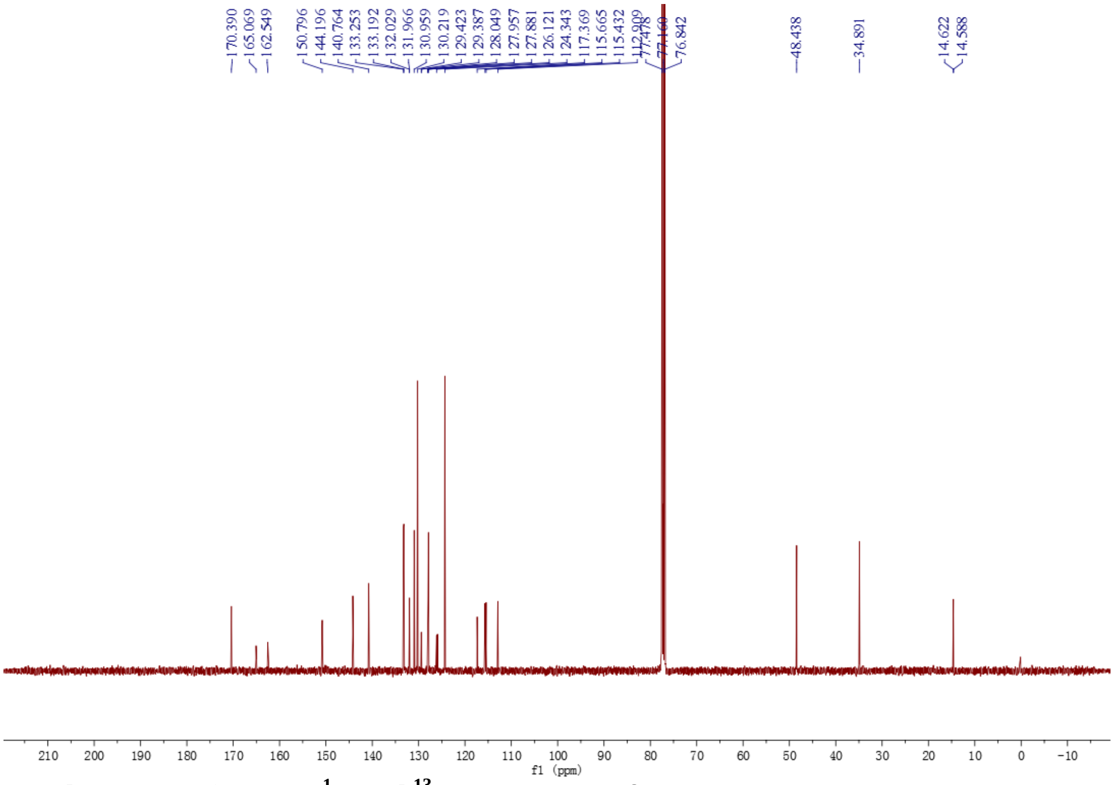

Supplementary Figure 29. ^{1}H and ^{13}C NMR spectra for 1t

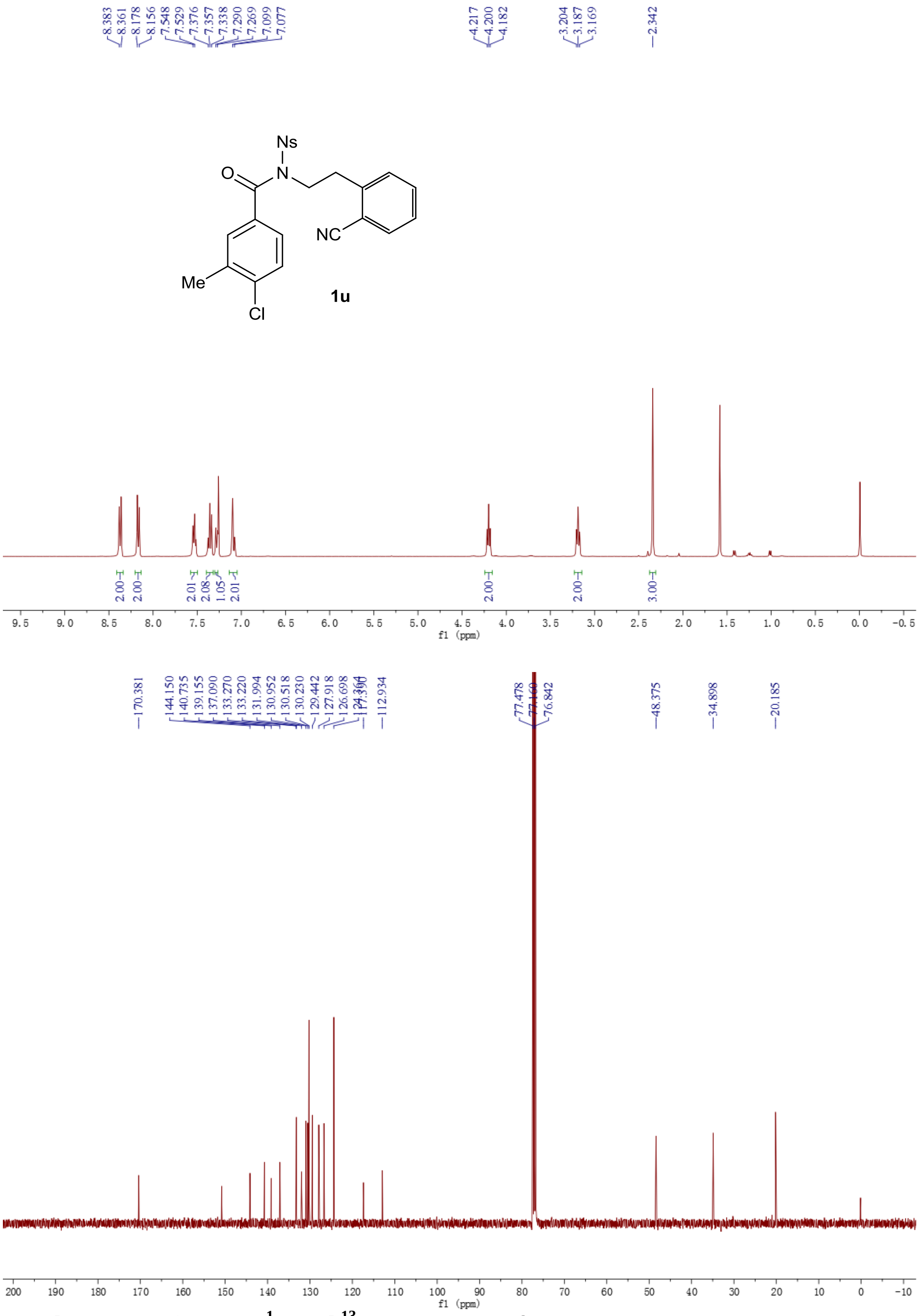

Supplementary Figure 30. ^{1}H and ^{13}C NMR spectra for 1u

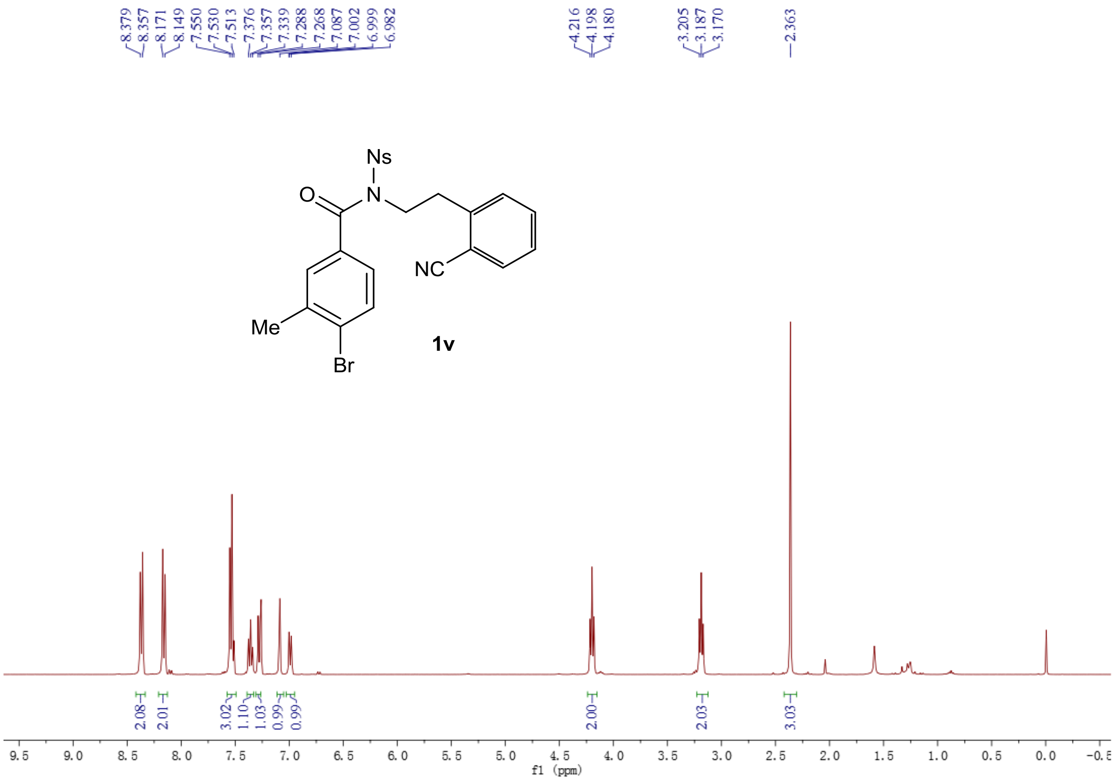

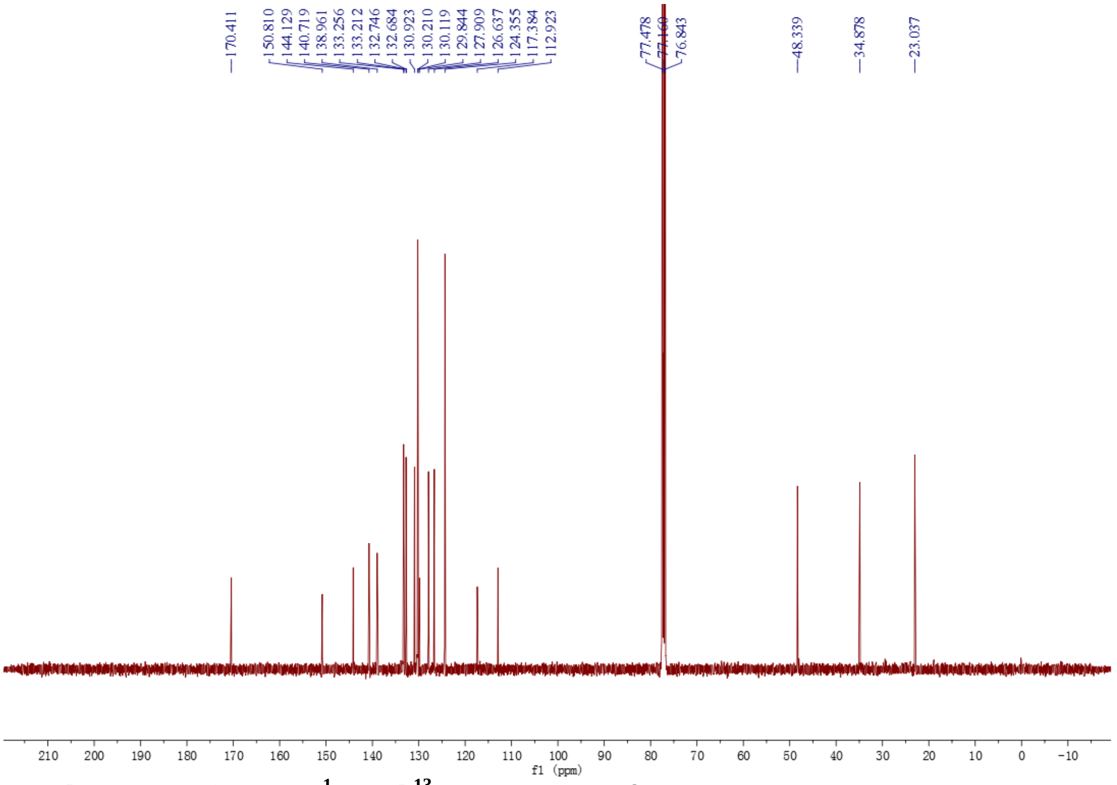

Supplementary Figure 31. ^{1}H and ^{13}C NMR spectra for 1v

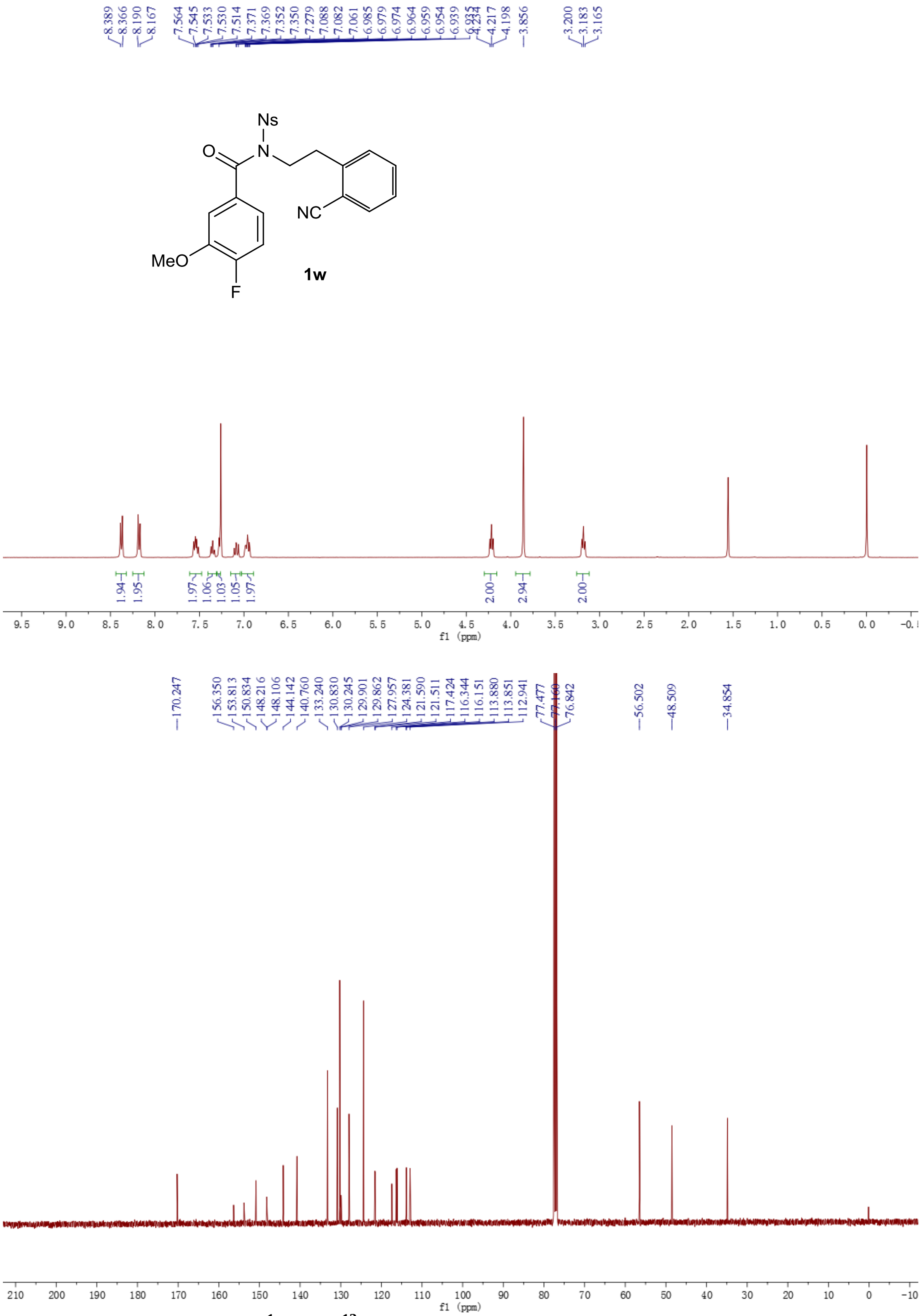

Supplementary Figure 32. ^{1}H and ^{13}C NMR spectra for 1w

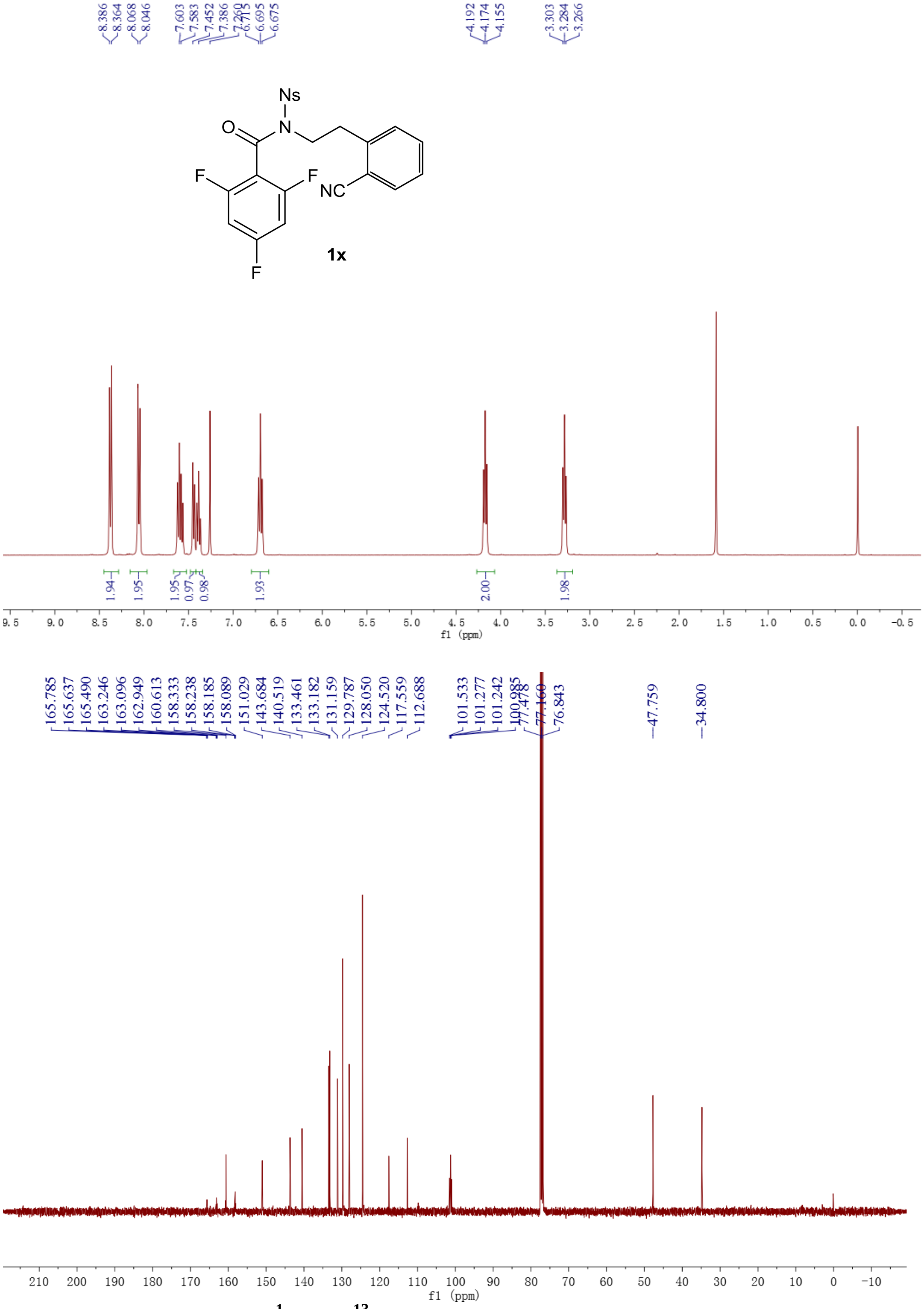

Supplementary Figure 33. ^{1}H and ^{13}C NMR spectra for 1x

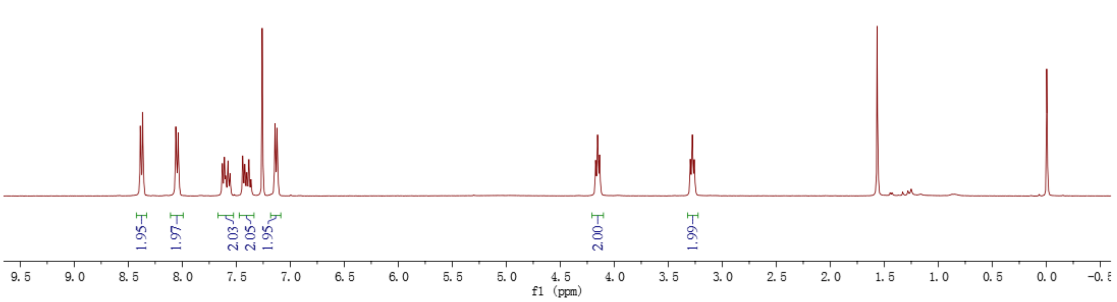

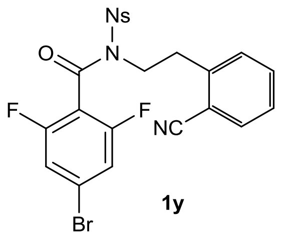

1y

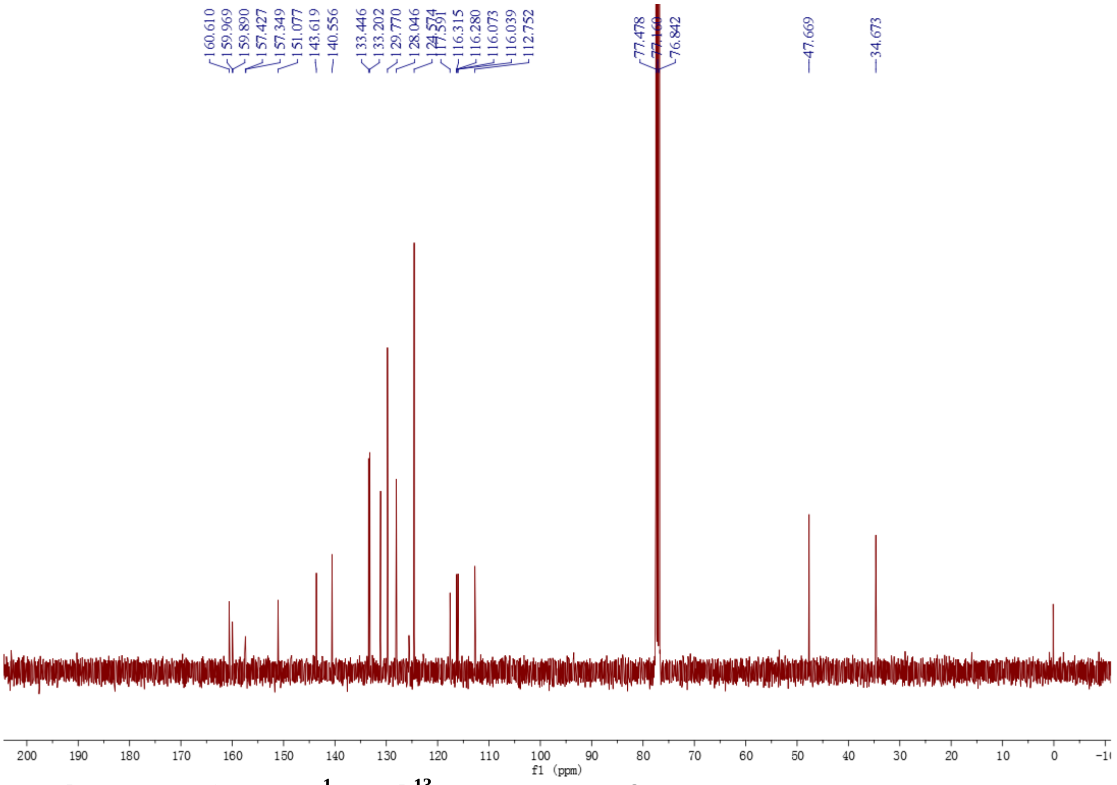

Supplementary Figure 34. ^{1}H and ^{13}C NMR spectra for 1y

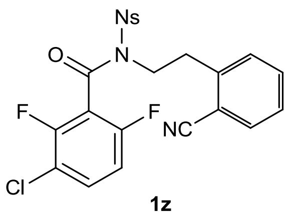

1z

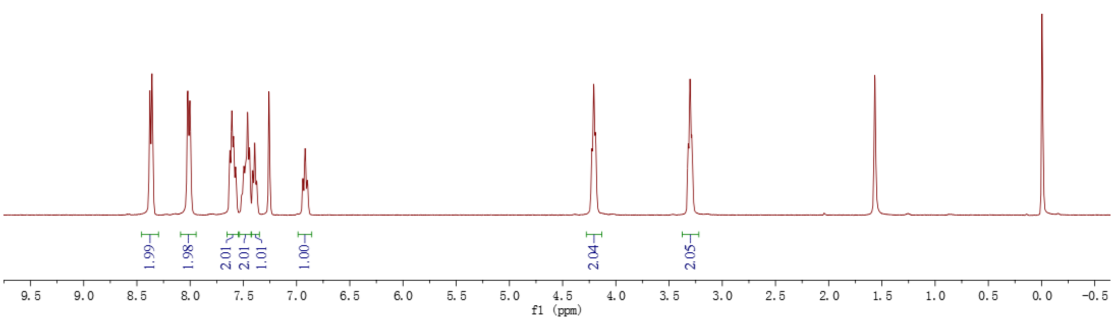

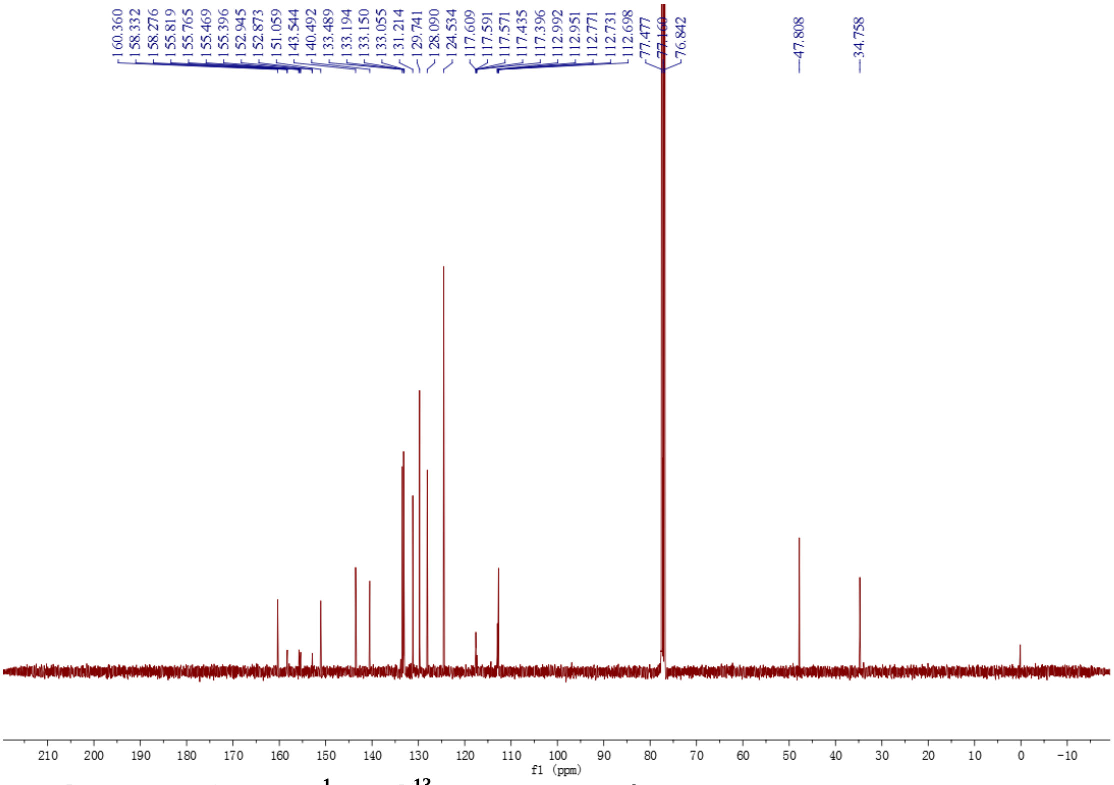

Supplementary Figure 35. ^{1}H and ^{13}C NMR spectra for 1z

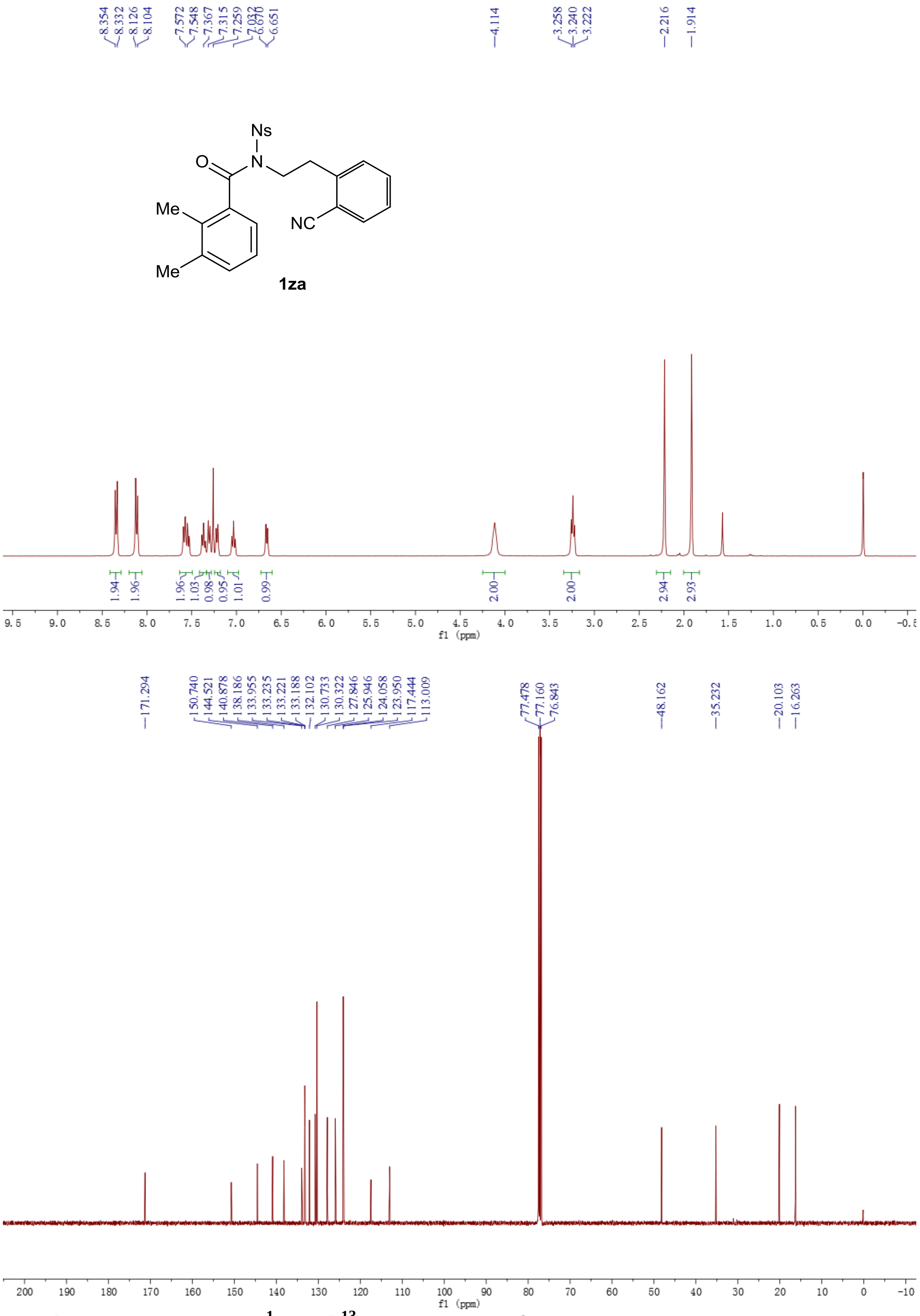

Supplementary Figure 36. ^{1}H and ^{13}C NMR spectra for 1za

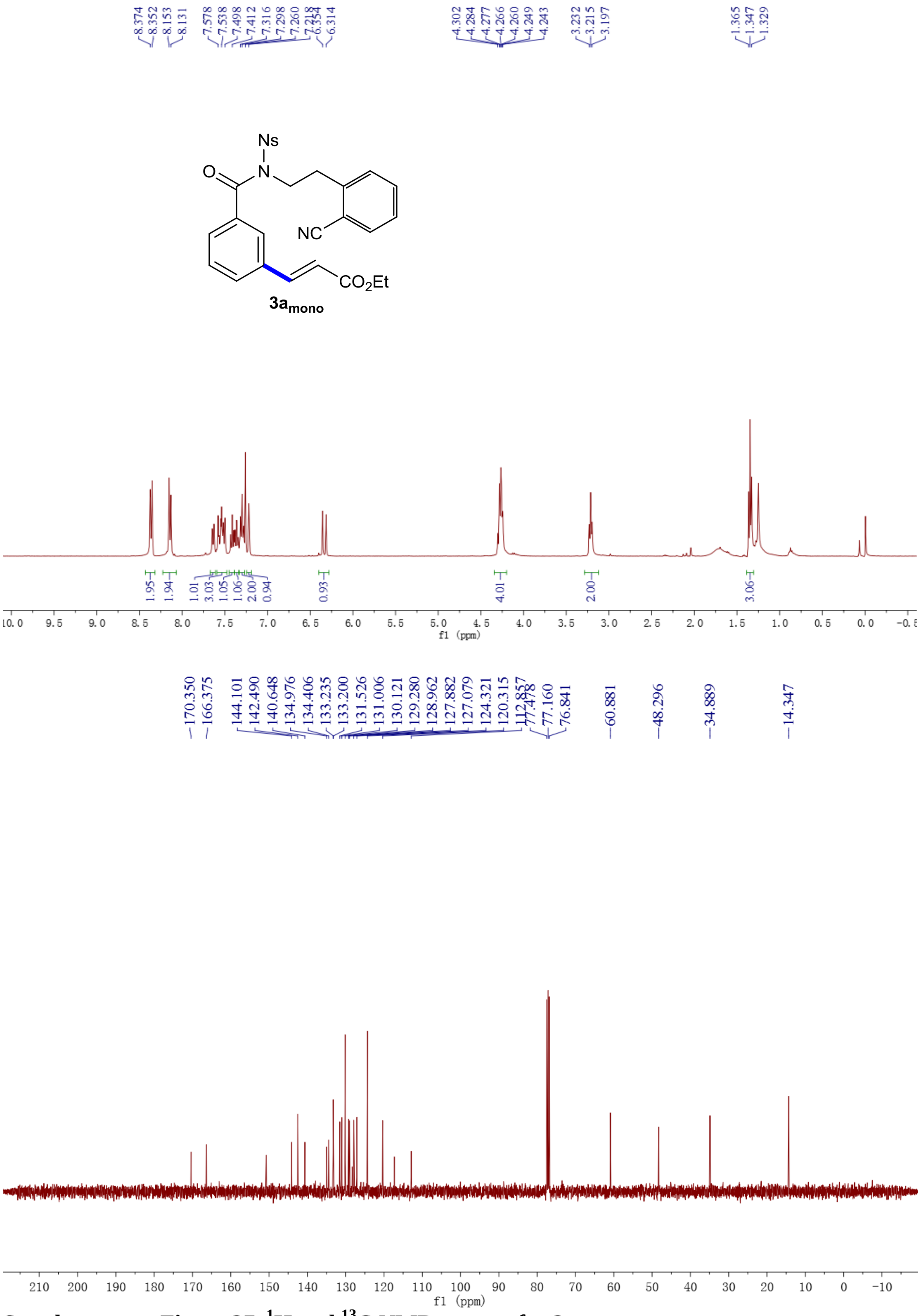

Supplementary Figure 37. ^{1}H and ^{13}C NMR spectra for 3a$_{mono}$

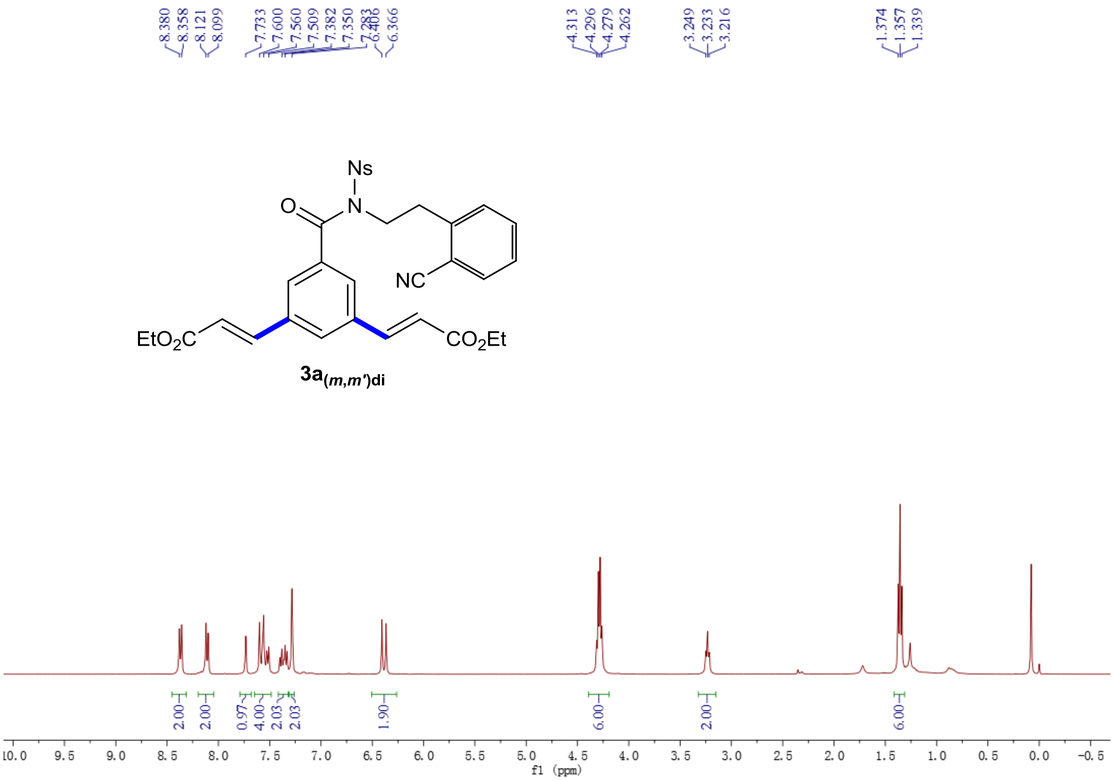

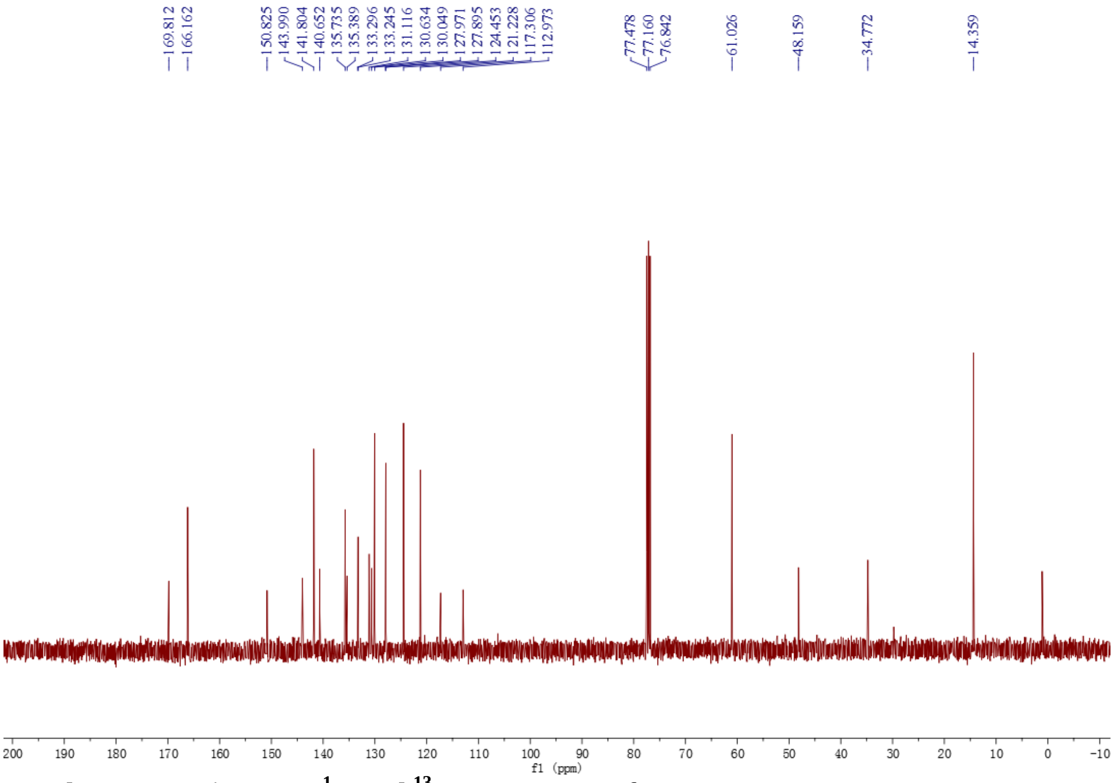

Supplementary Figure 38. ^{1}H and ^{13}C NMR spectra for 3a$_{(m,m')di}$

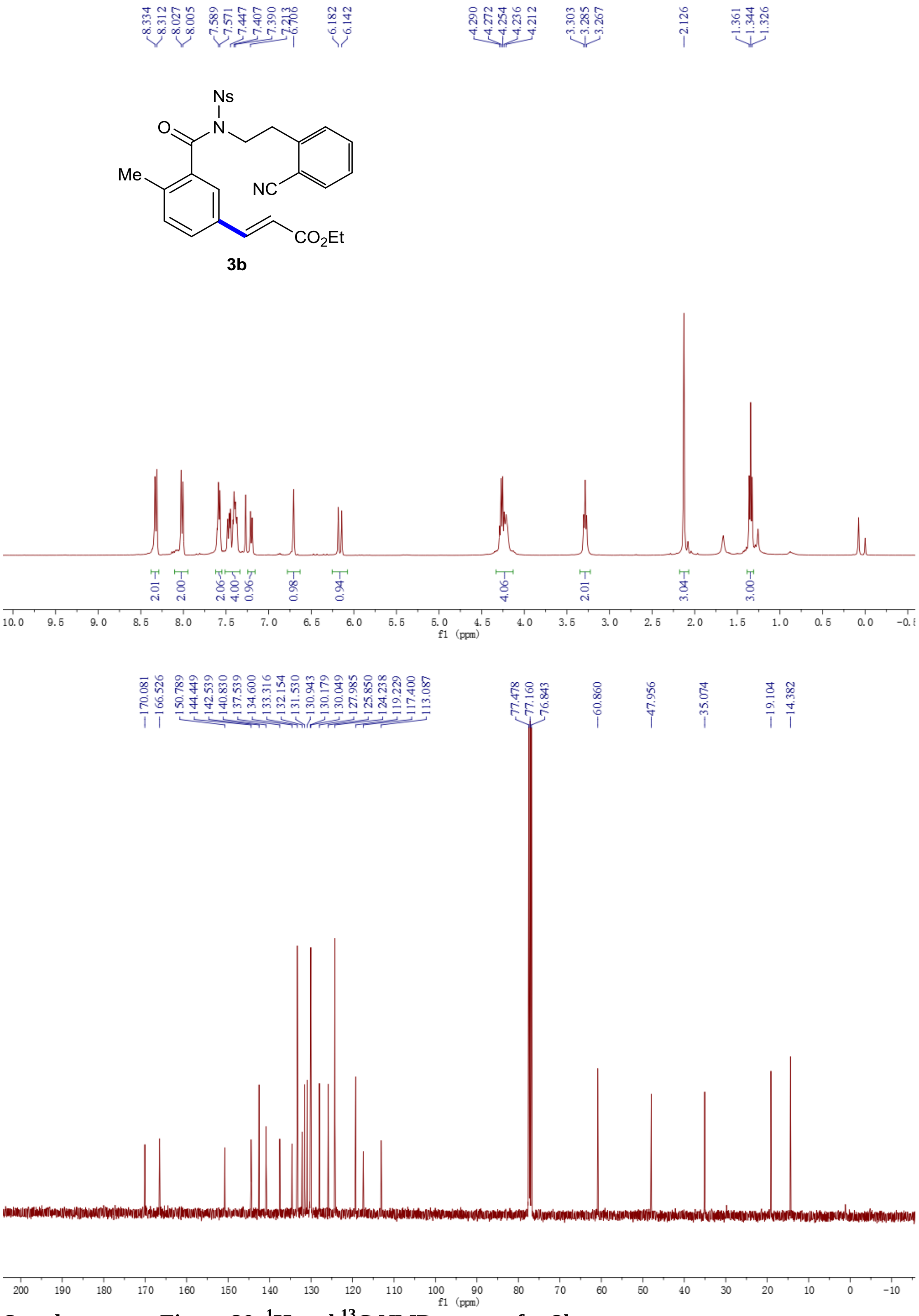

Supplementary Figure 39. ^{1}H and ^{13}C NMR spectra for 3b

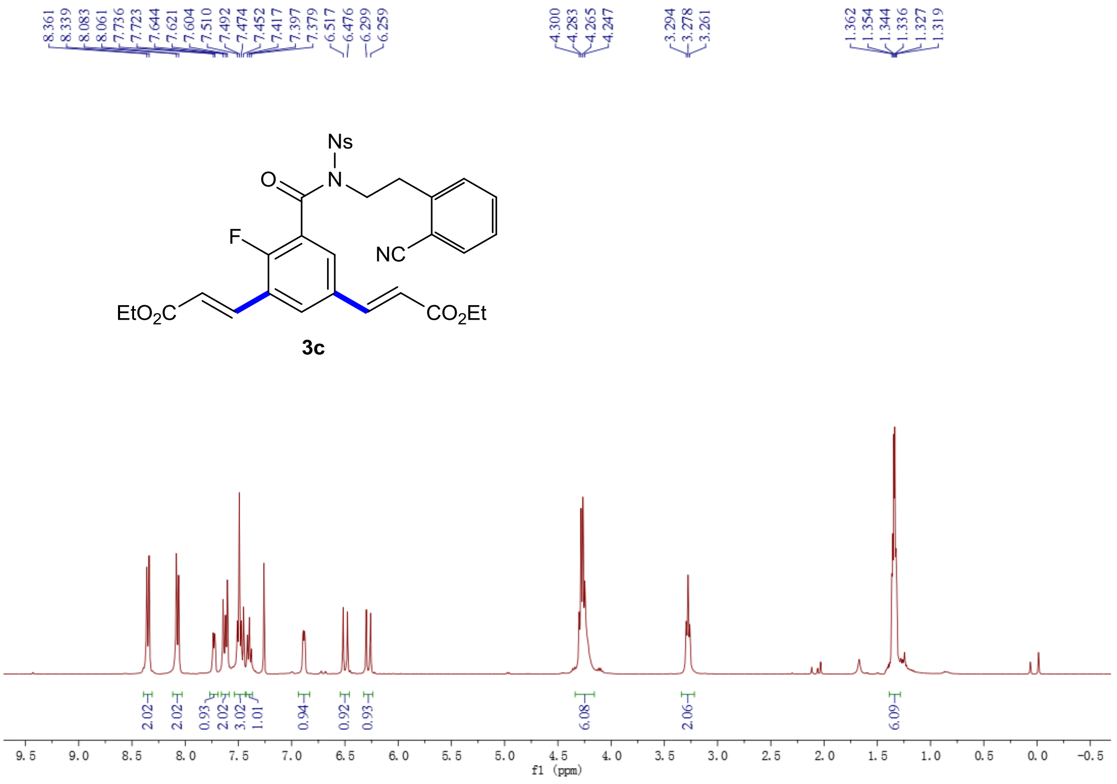

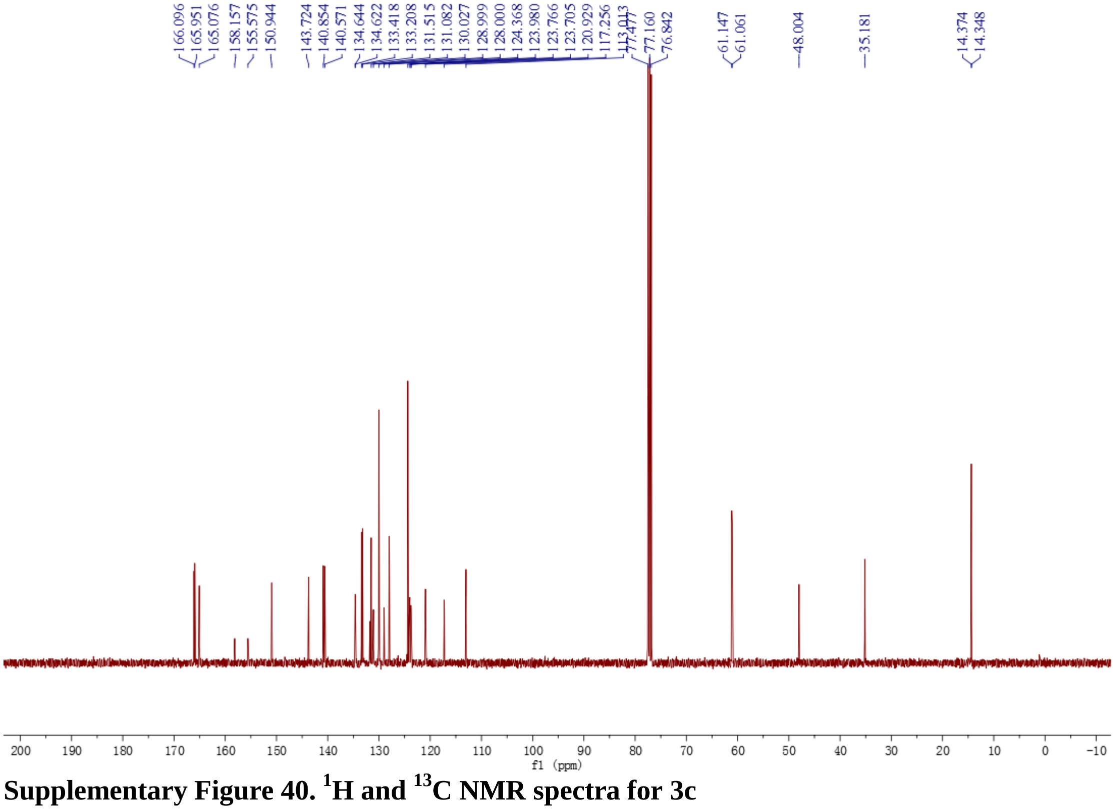

Supplementary Figure 40. ^{1}H and ^{13}C NMR spectra for 3c

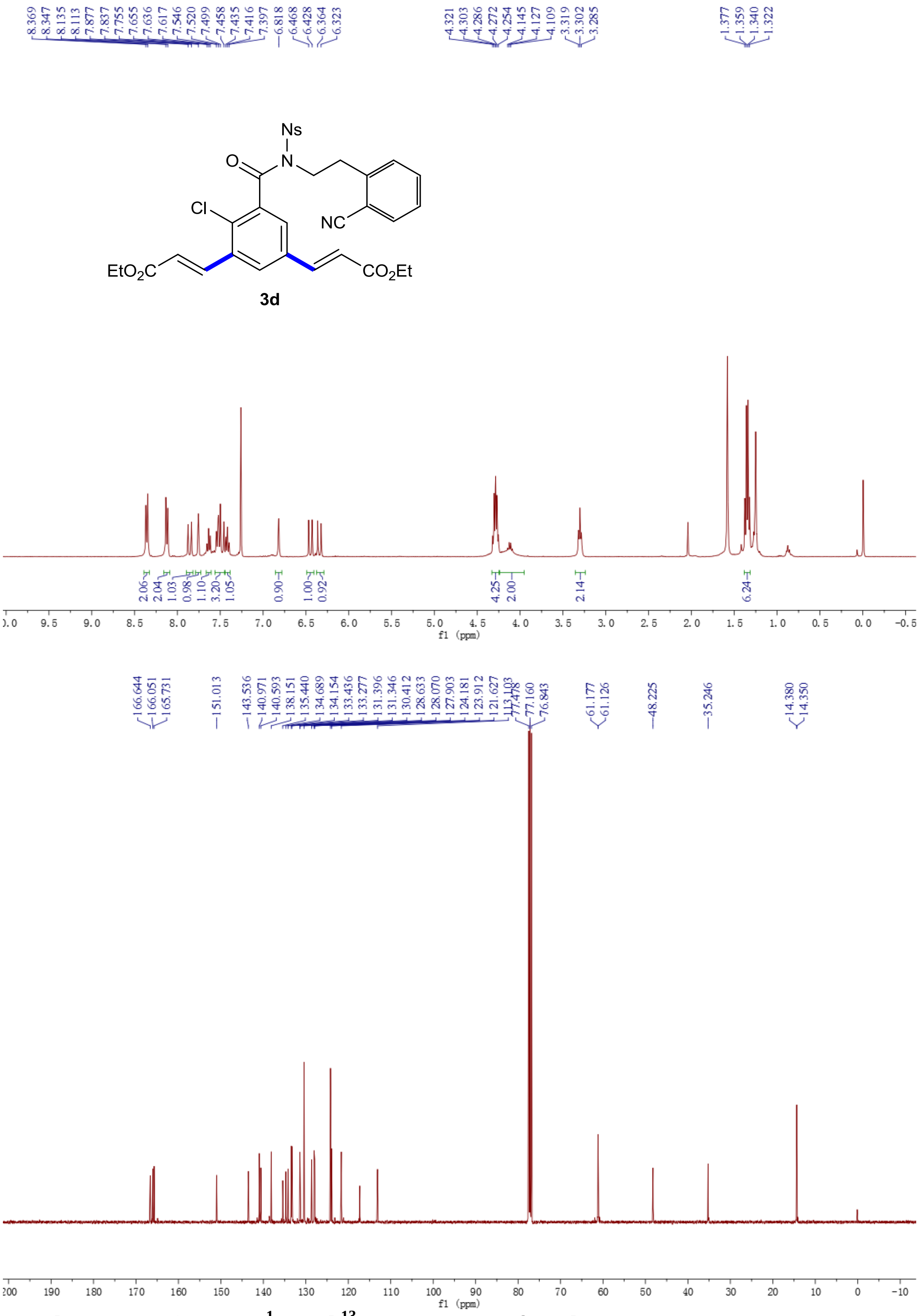

Supplementary Figure 41. ^{1}H and ^{13}C NMR spectra for 3d

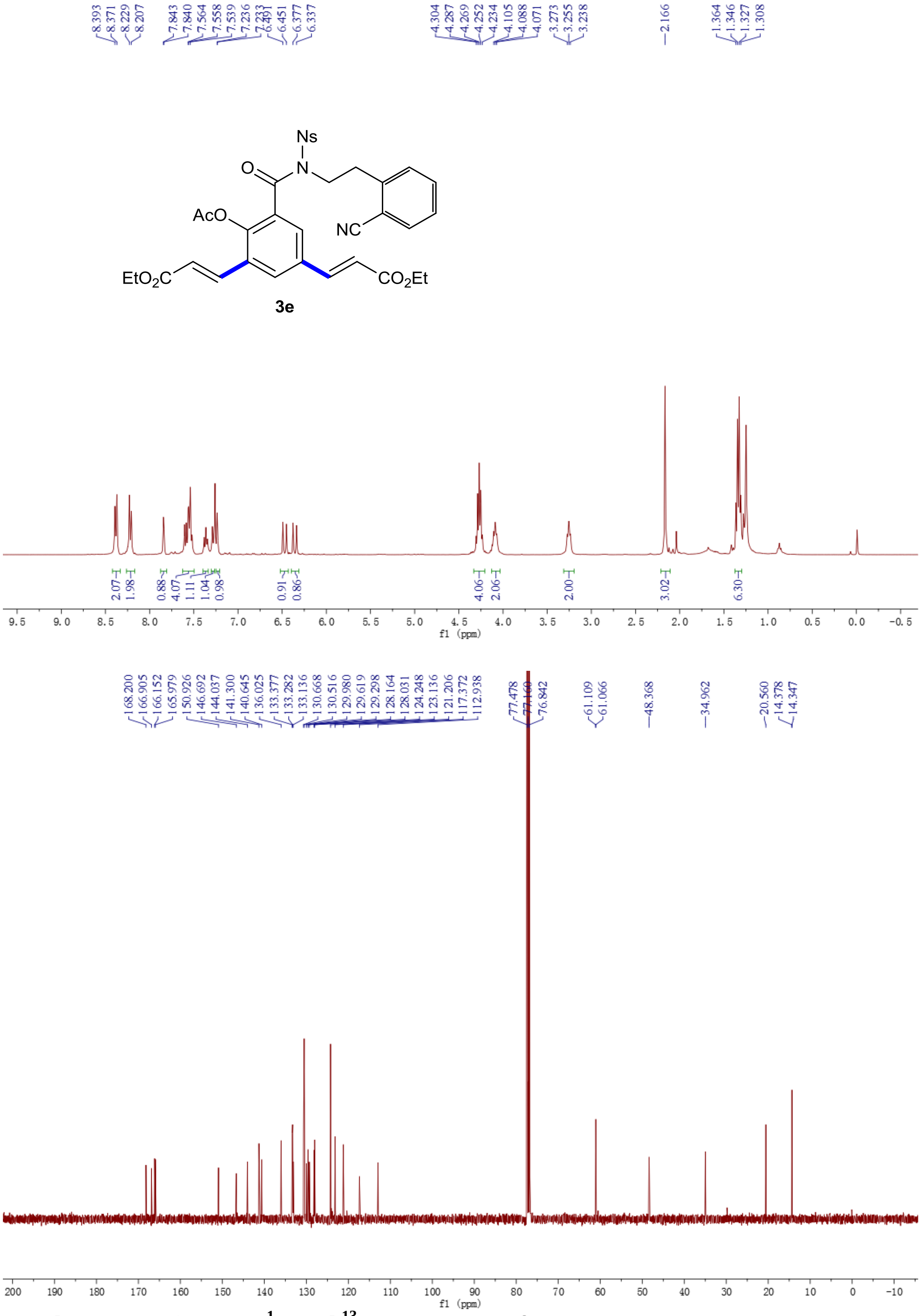

Supplementary Figure 42. ^{1}H and ^{13}C NMR spectra for 3e

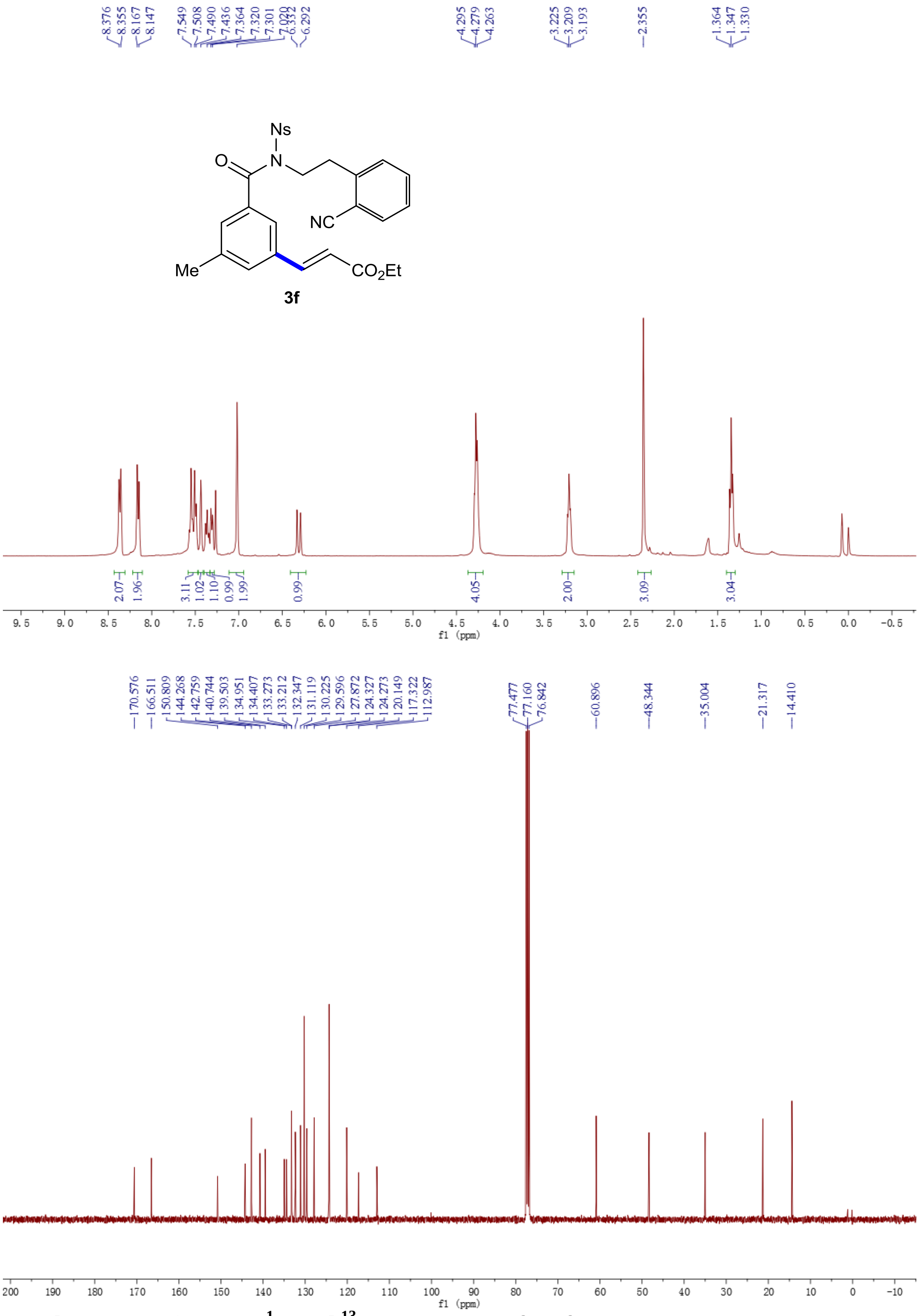

Supplementary Figure 43. ^{1}H and ^{13}C NMR spectra for 3f

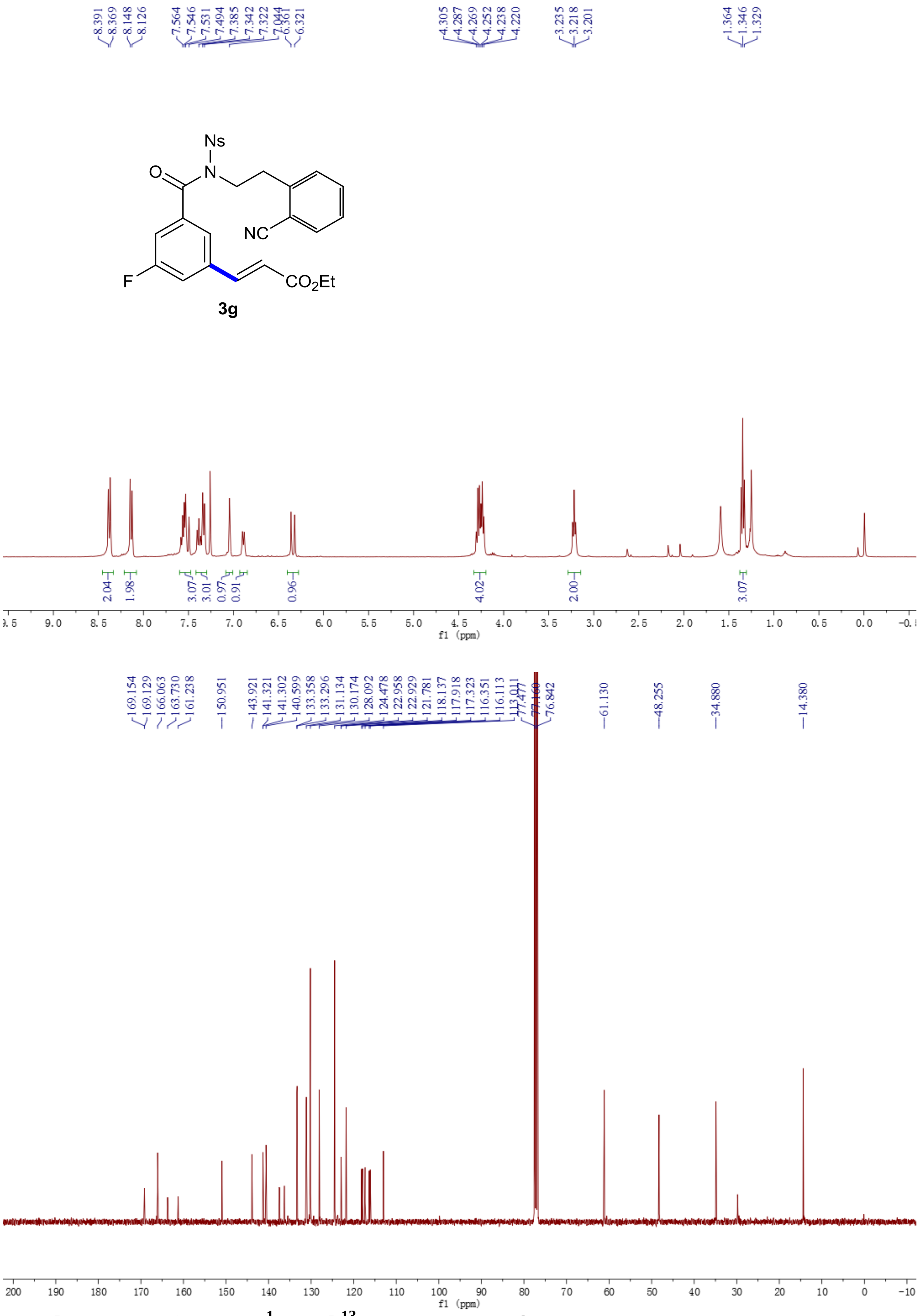

Supplementary Figure 44. ^{1}H and ^{13}C NMR spectra for 3g

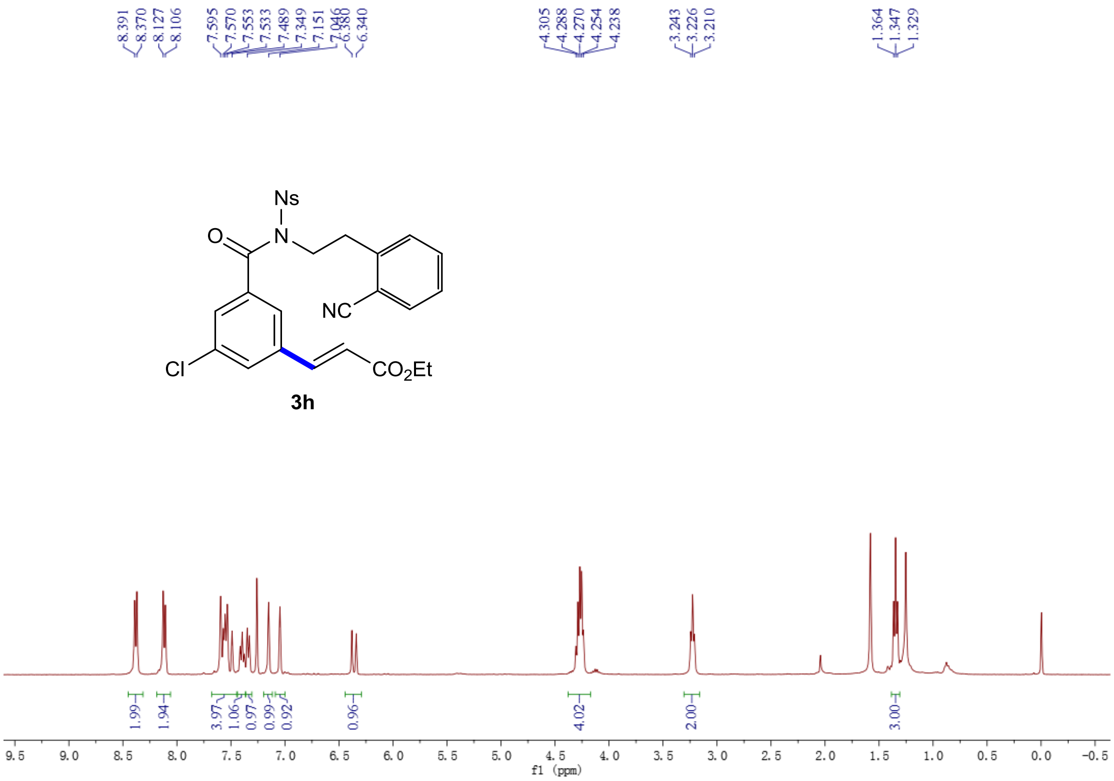

3h

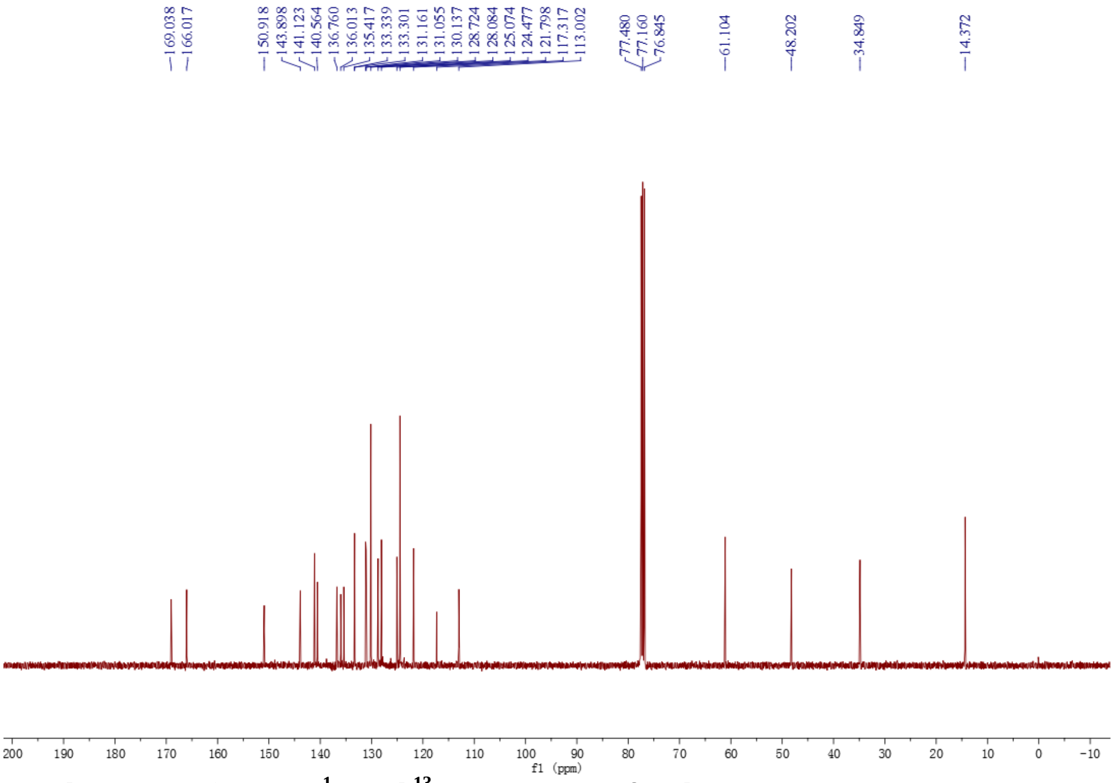

Supplementary Figure 45. ^{1}H and ^{13}C NMR spectra for 3h

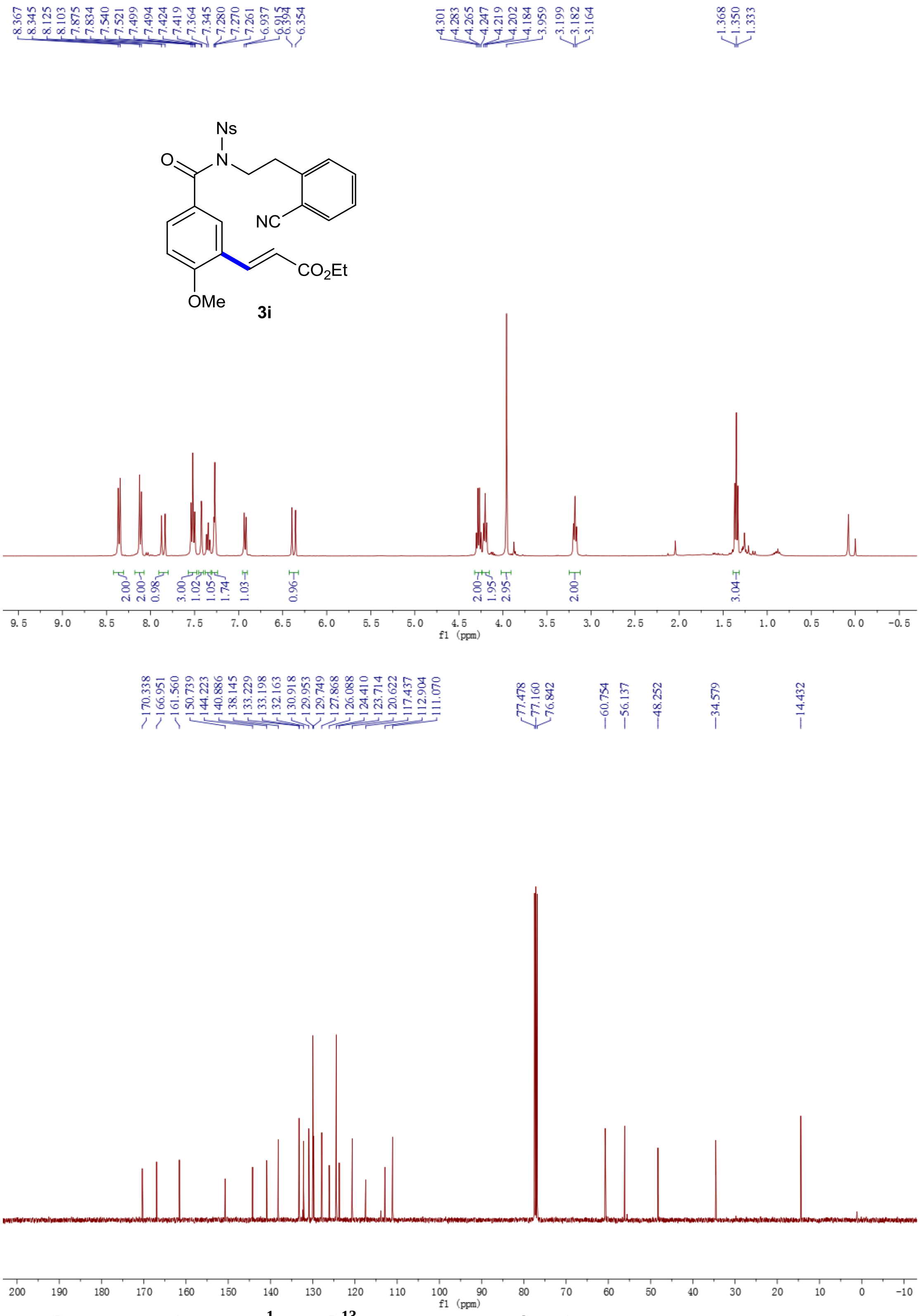

Supplementary Figure 46. ^{1}H and ^{13}C NMR spectra for 3i

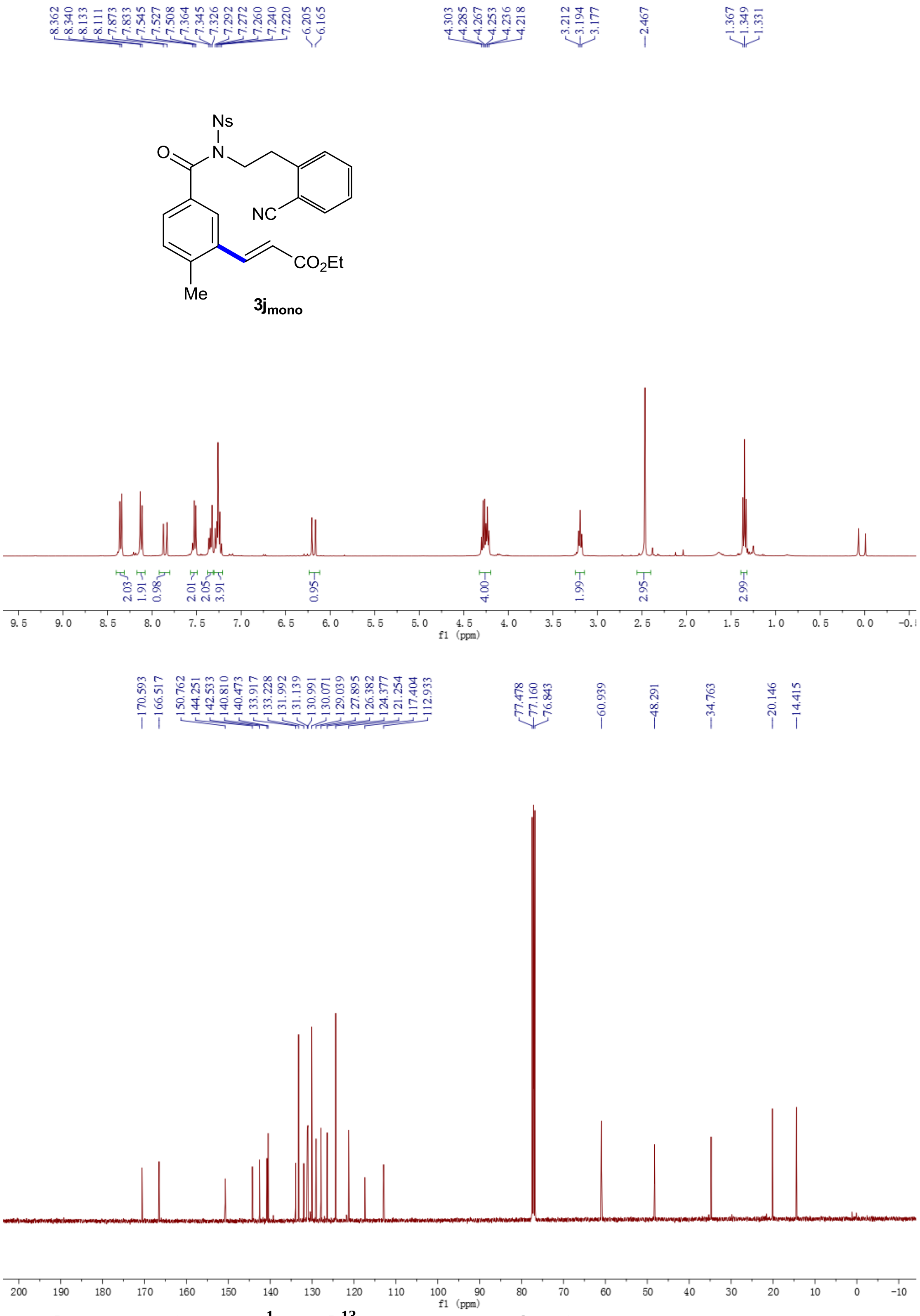

Supplementary Figure 47. ^{1}**H and** ^{13}**C NMR spectra for 3j$_{mono}$**

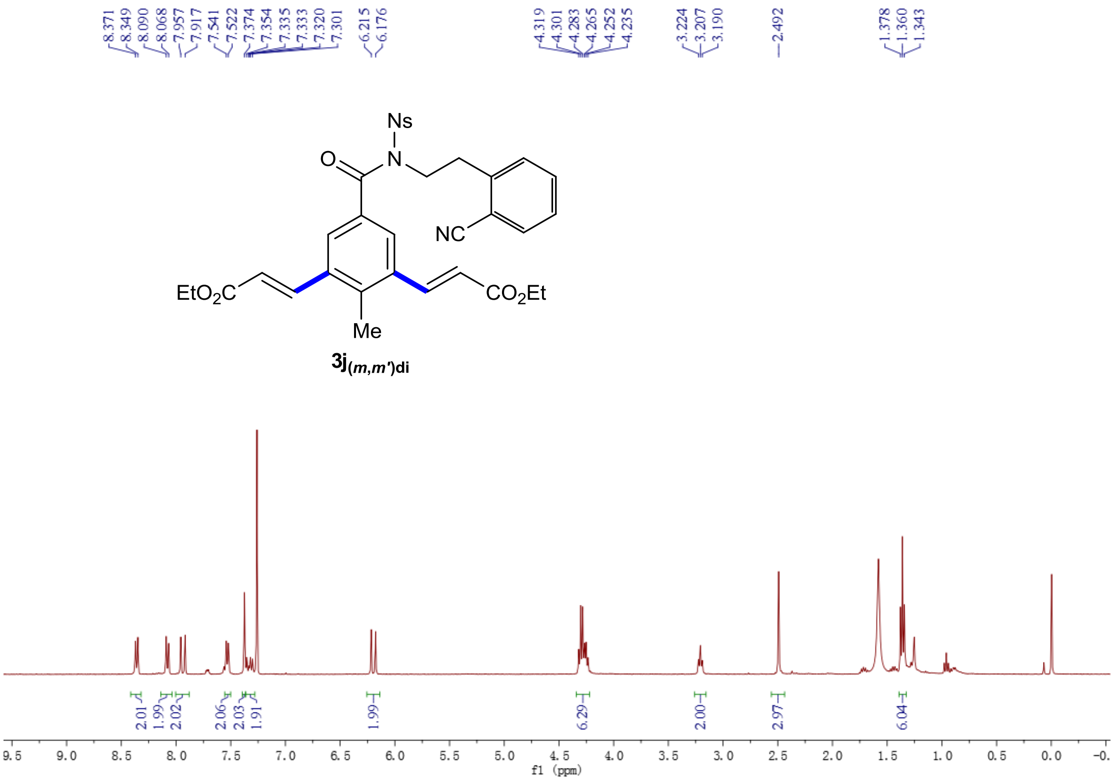

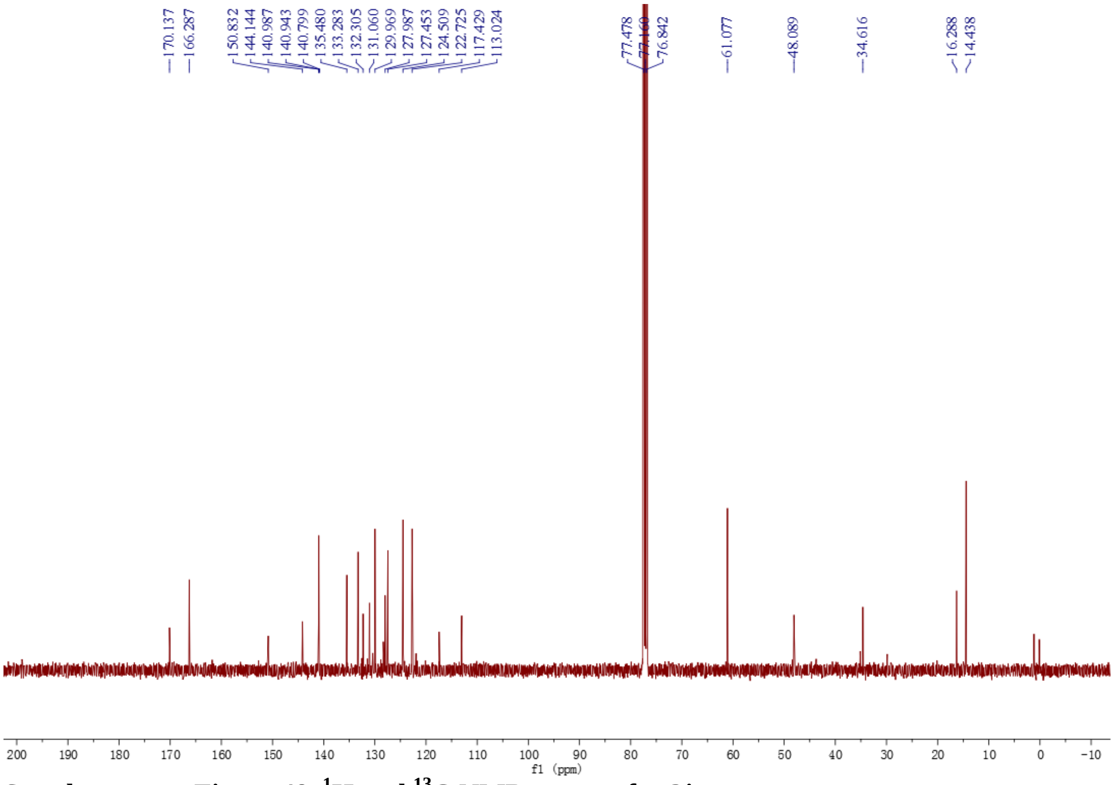

Supplementary Figure 48. ^{1}H and ^{13}C NMR spectra for $3j_{(m,m')di}$

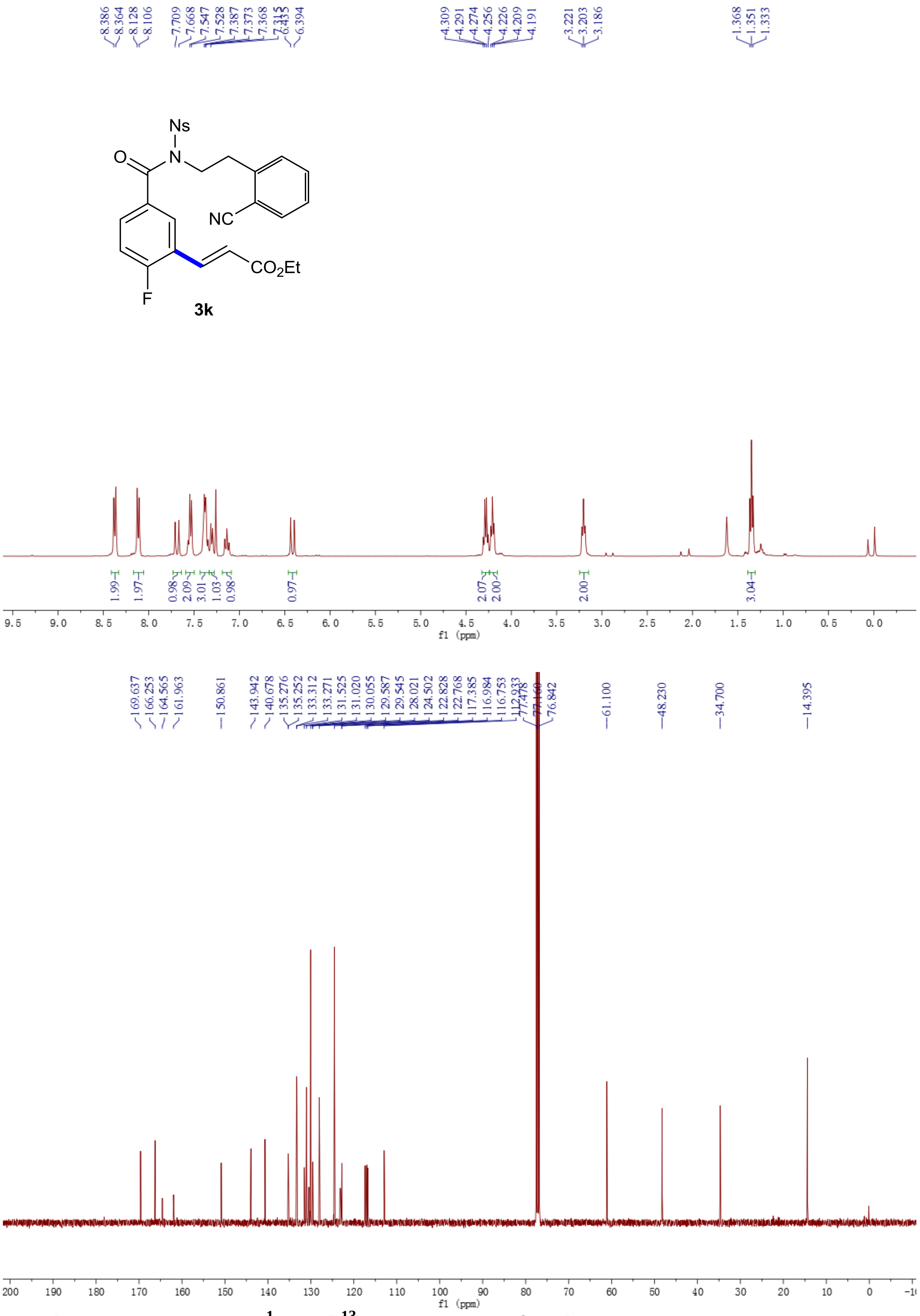

Supplementary Figure 49. ^{1}H and ^{13}C NMR spectra for 3k

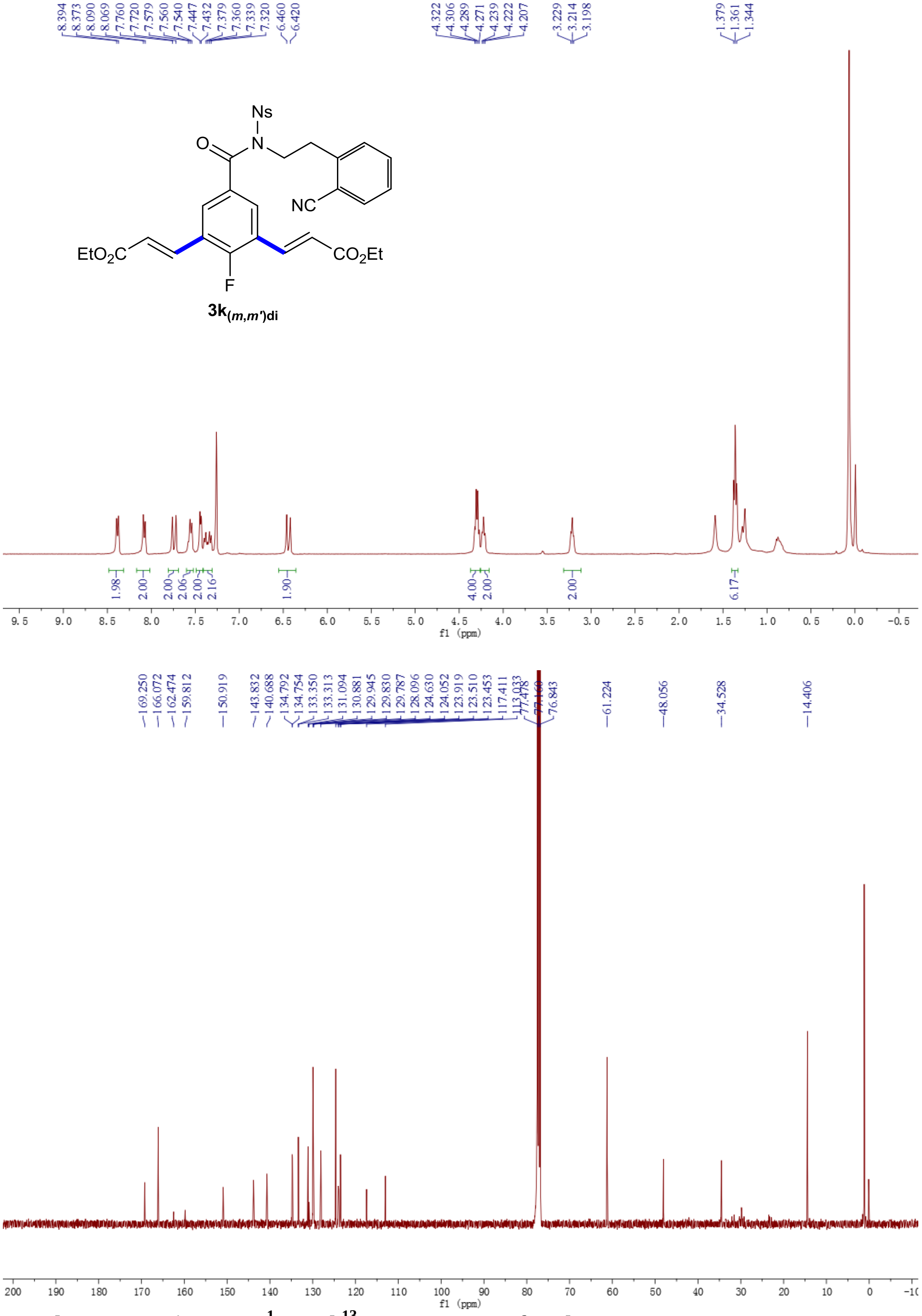

Supplementary Figure 50. ^{1}H and ^{13}C NMR spectra for 3k$_{(m,m')di}$

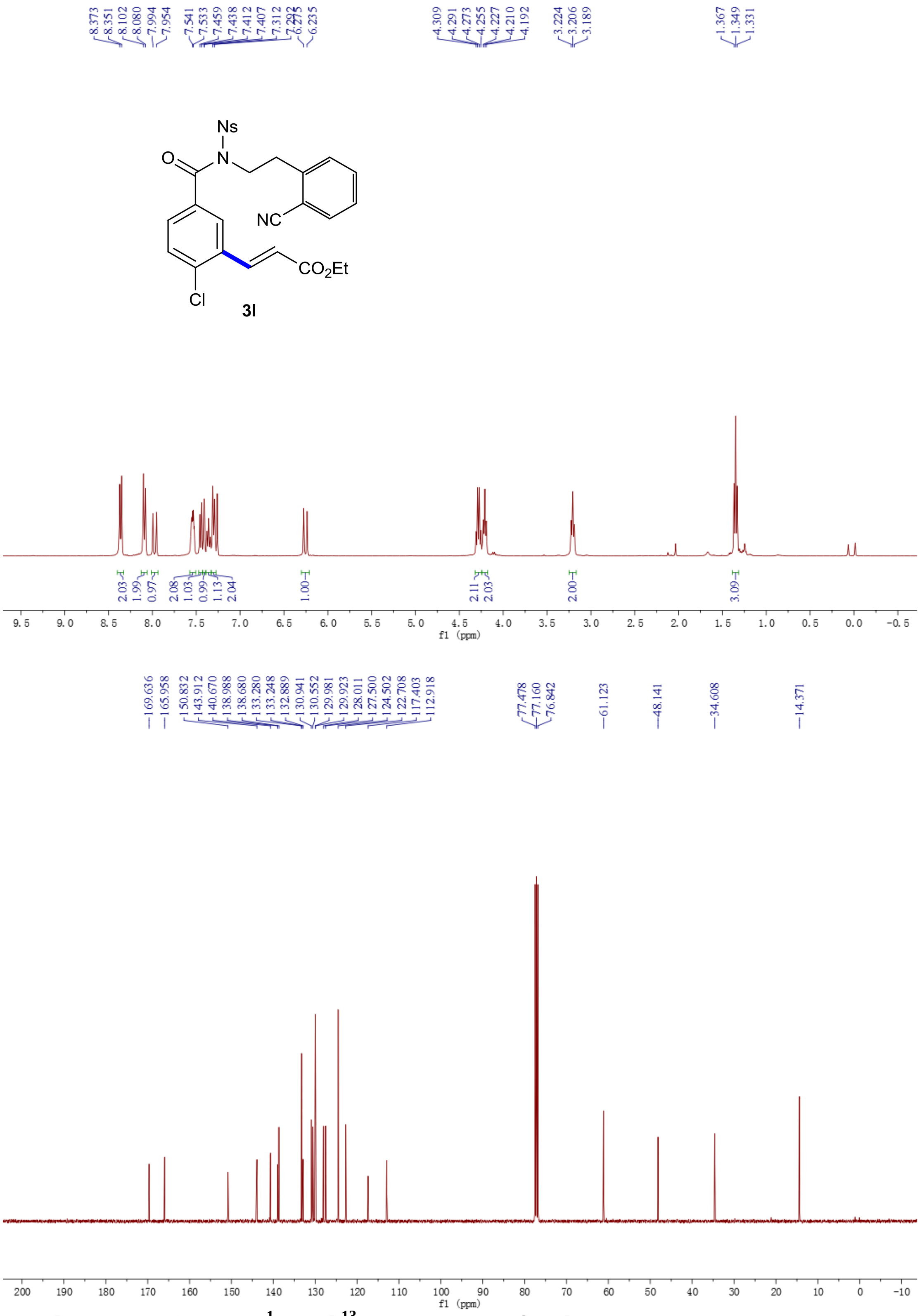

Supplementary Figure 51. ^{1}H and ^{13}C NMR spectra for 3l

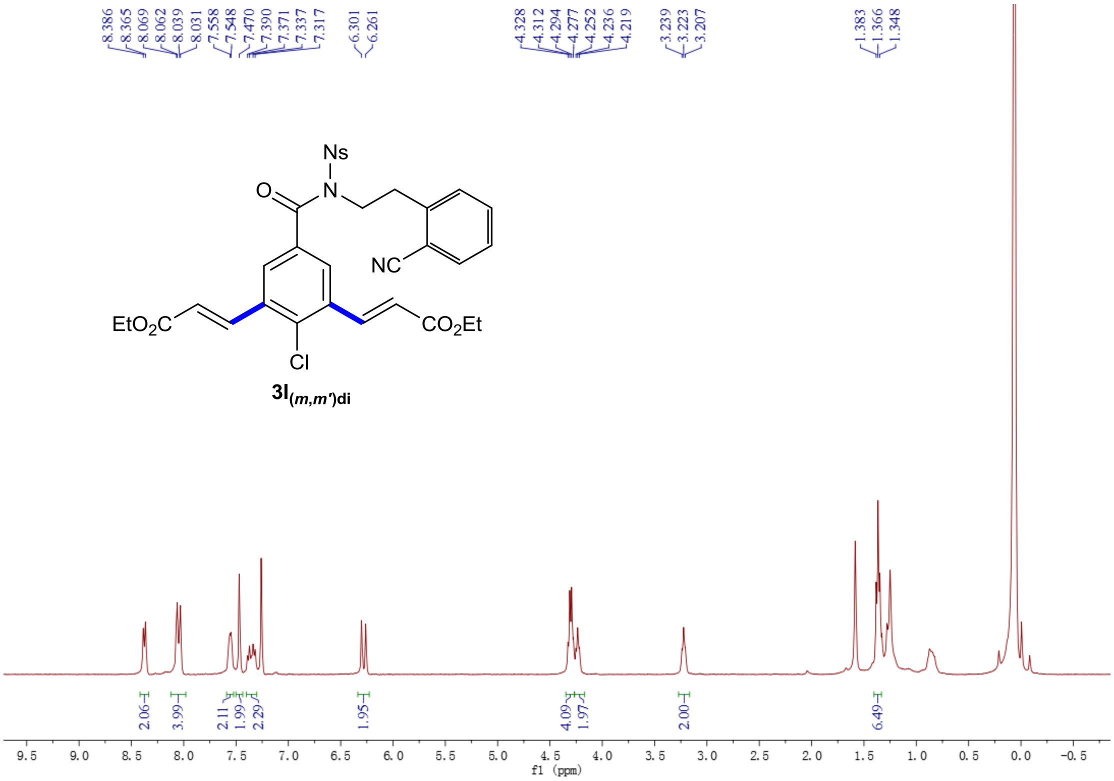

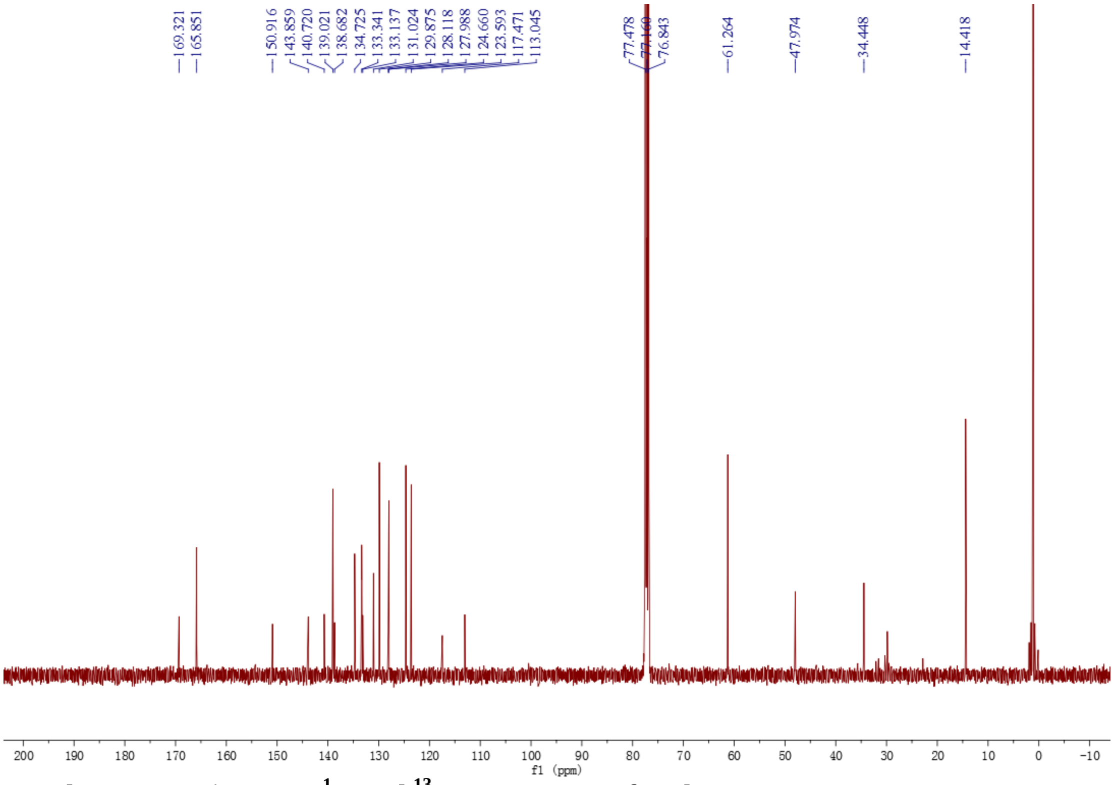

Supplementary Figure 52. ^{1}H and ^{13}C NMR spectra for $3l_{(m,m')di}$

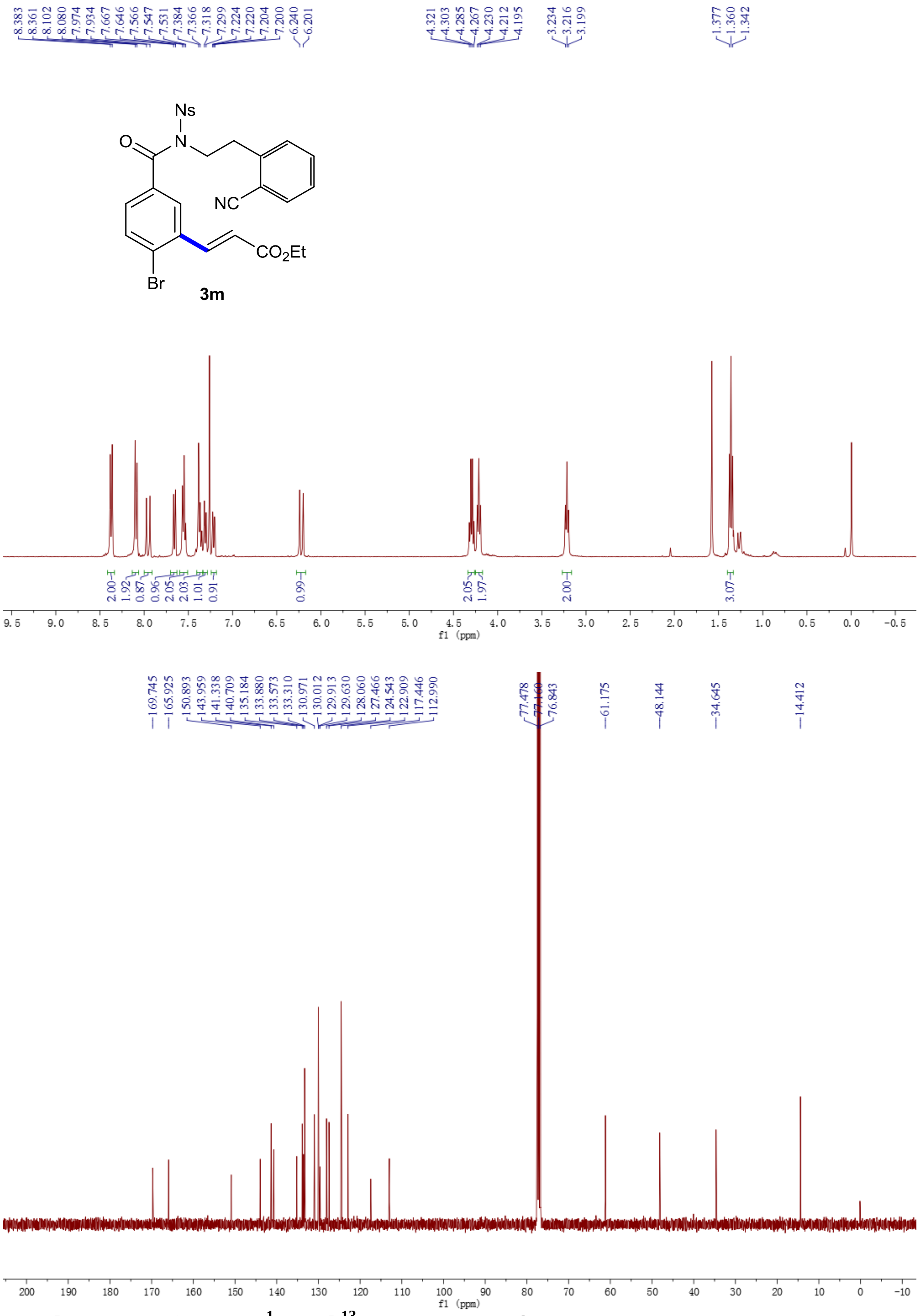

Supplementary Figure 53. ^{1}H and ^{13}C NMR spectra for 3m

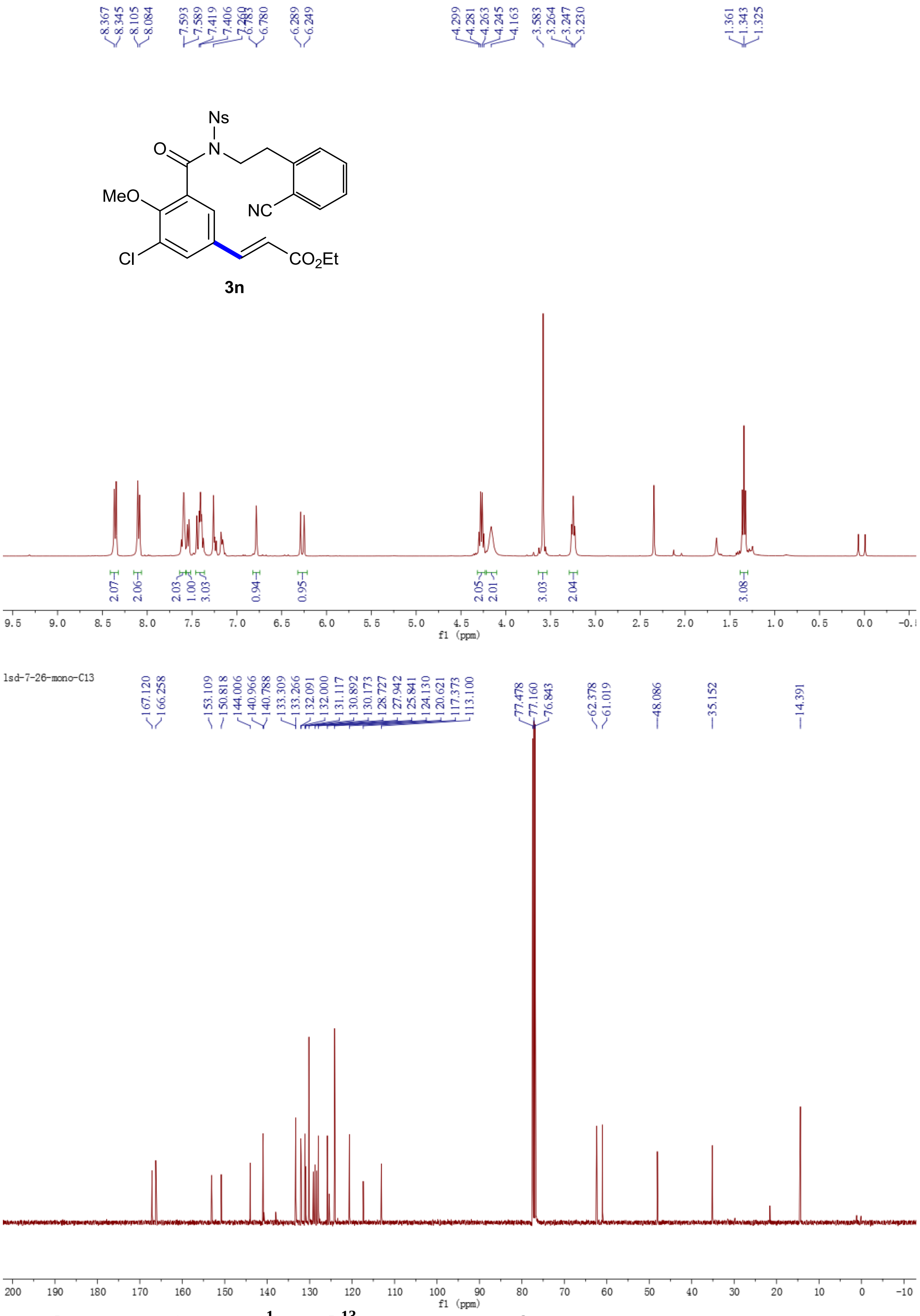

Supplementary Figure 54. **^{1}H and ^{13}C NMR spectra for 3n**

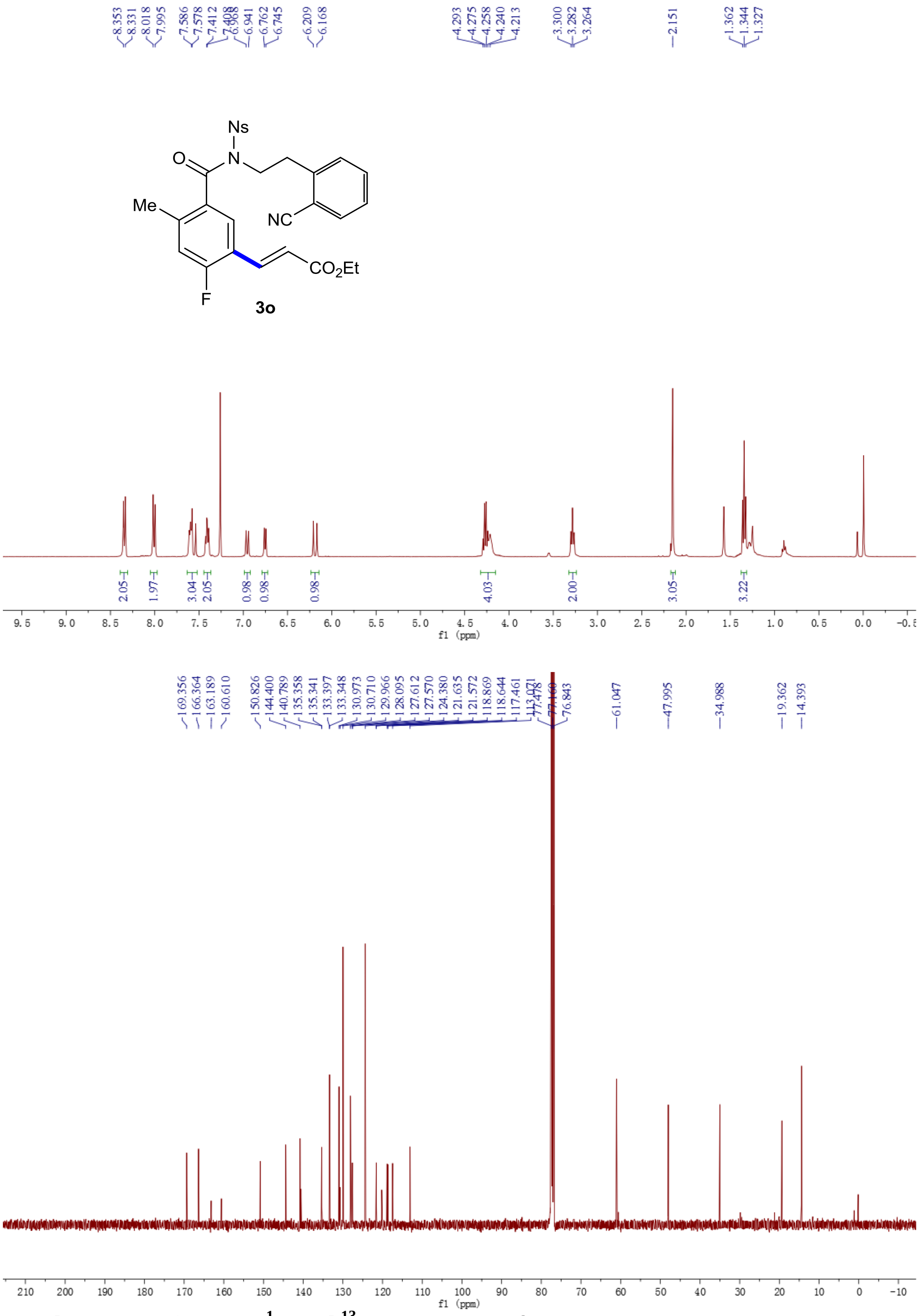

Supplementary Figure 55. ^{1}H and ^{13}C NMR spectra for 3o

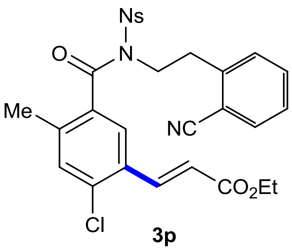

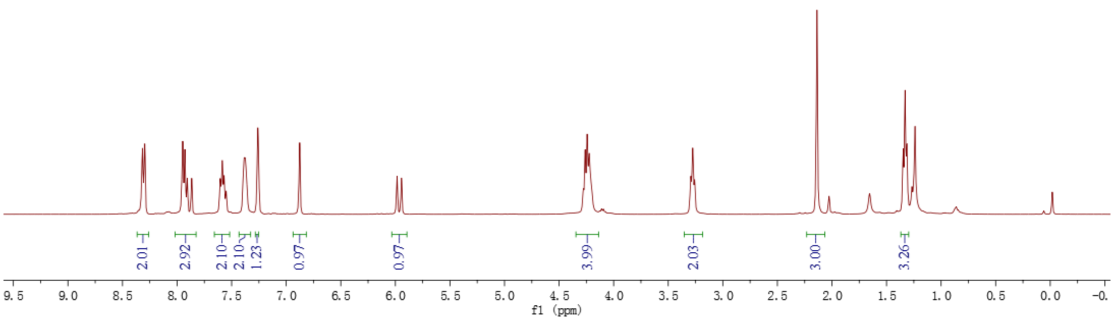

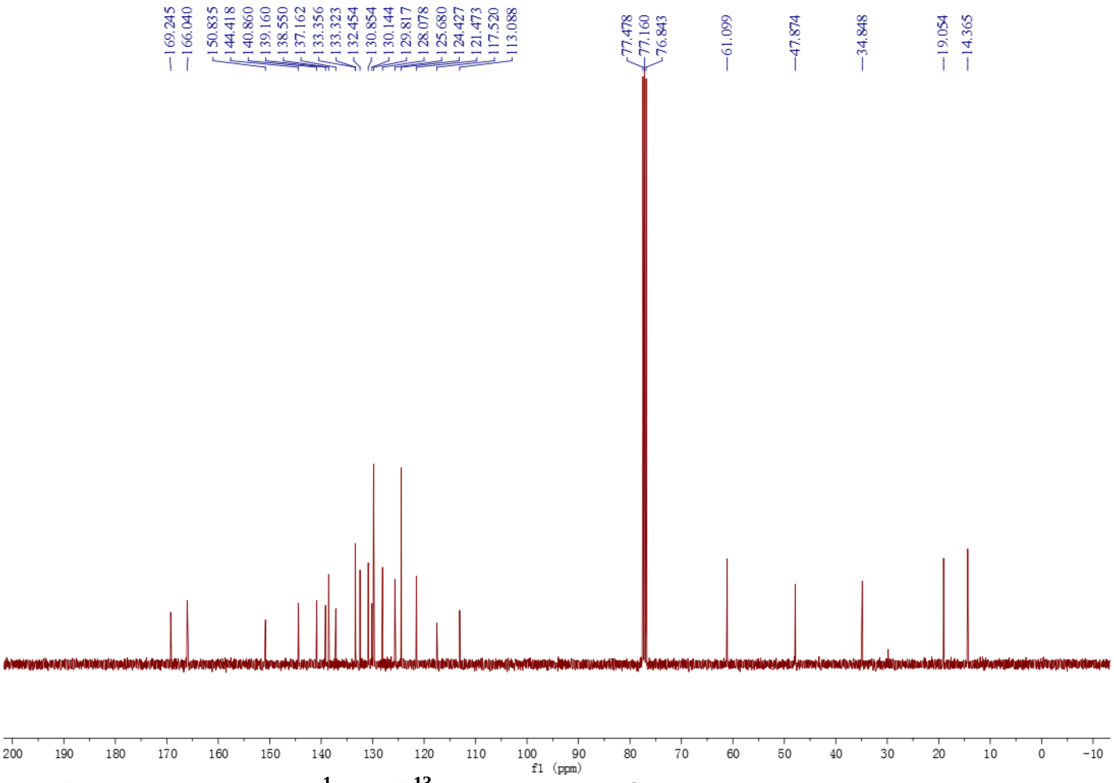

Supplementary Figure 56. ^{1}H and ^{13}C NMR spectra for 3p

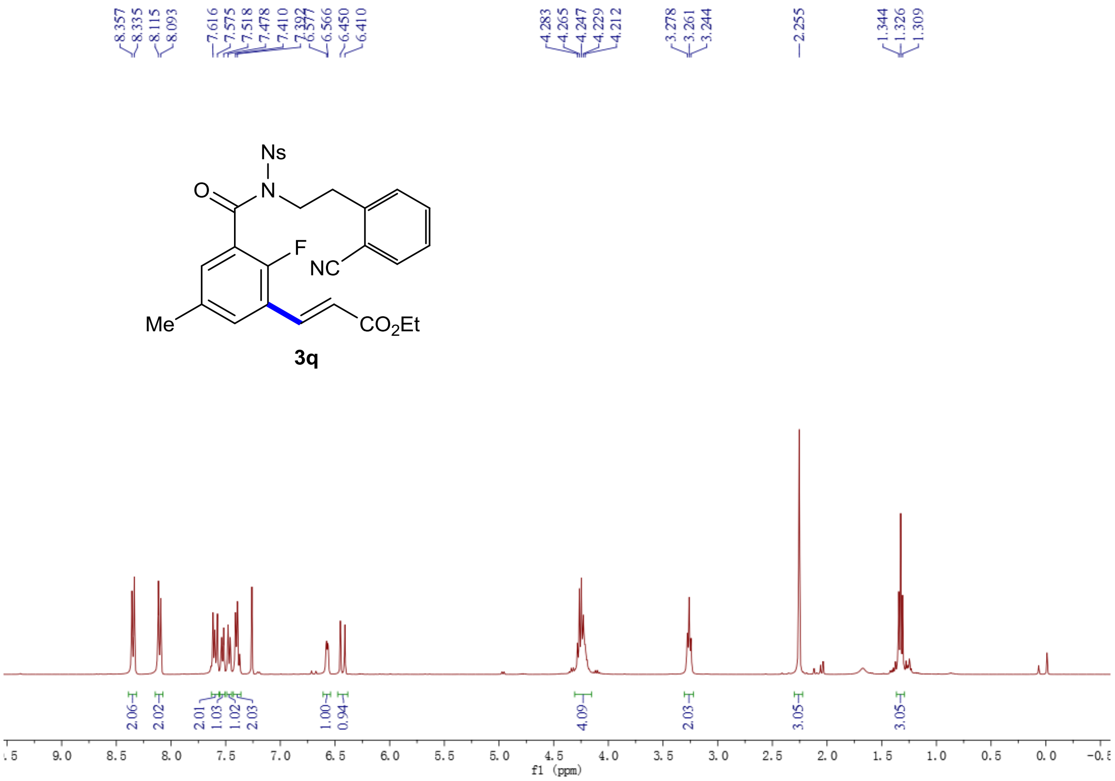

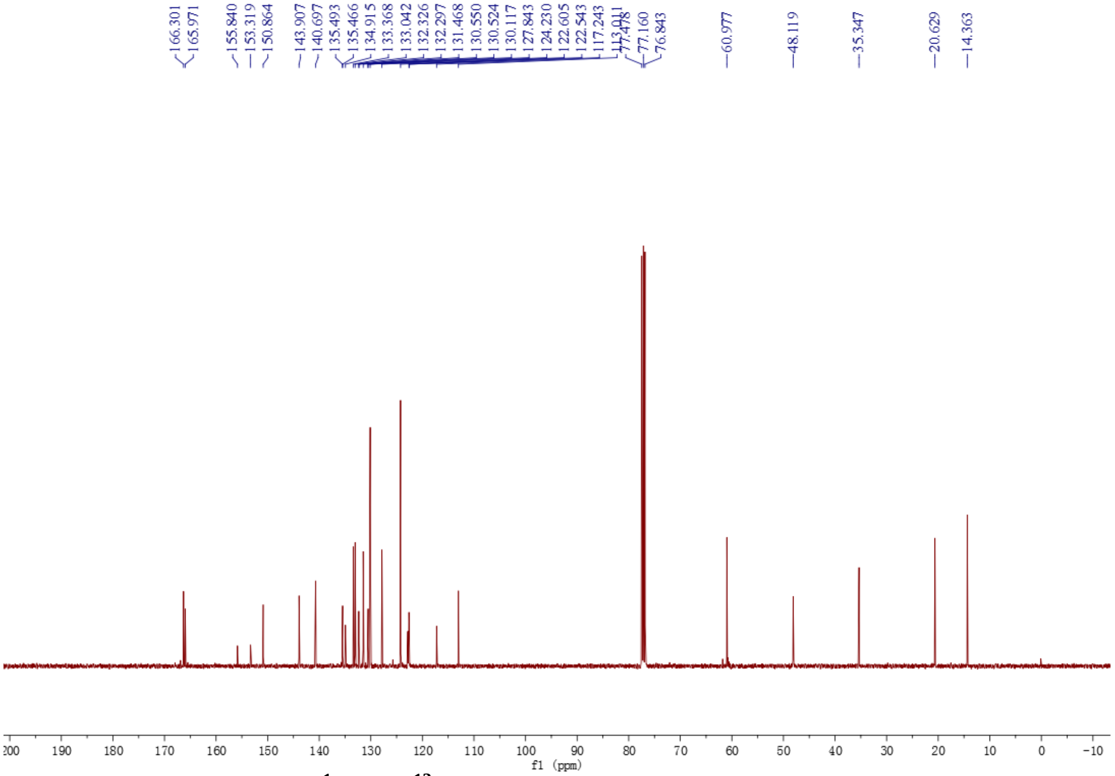

Supplementary Figure 57. ^{1}H and ^{13}C NMR spectra for 3q

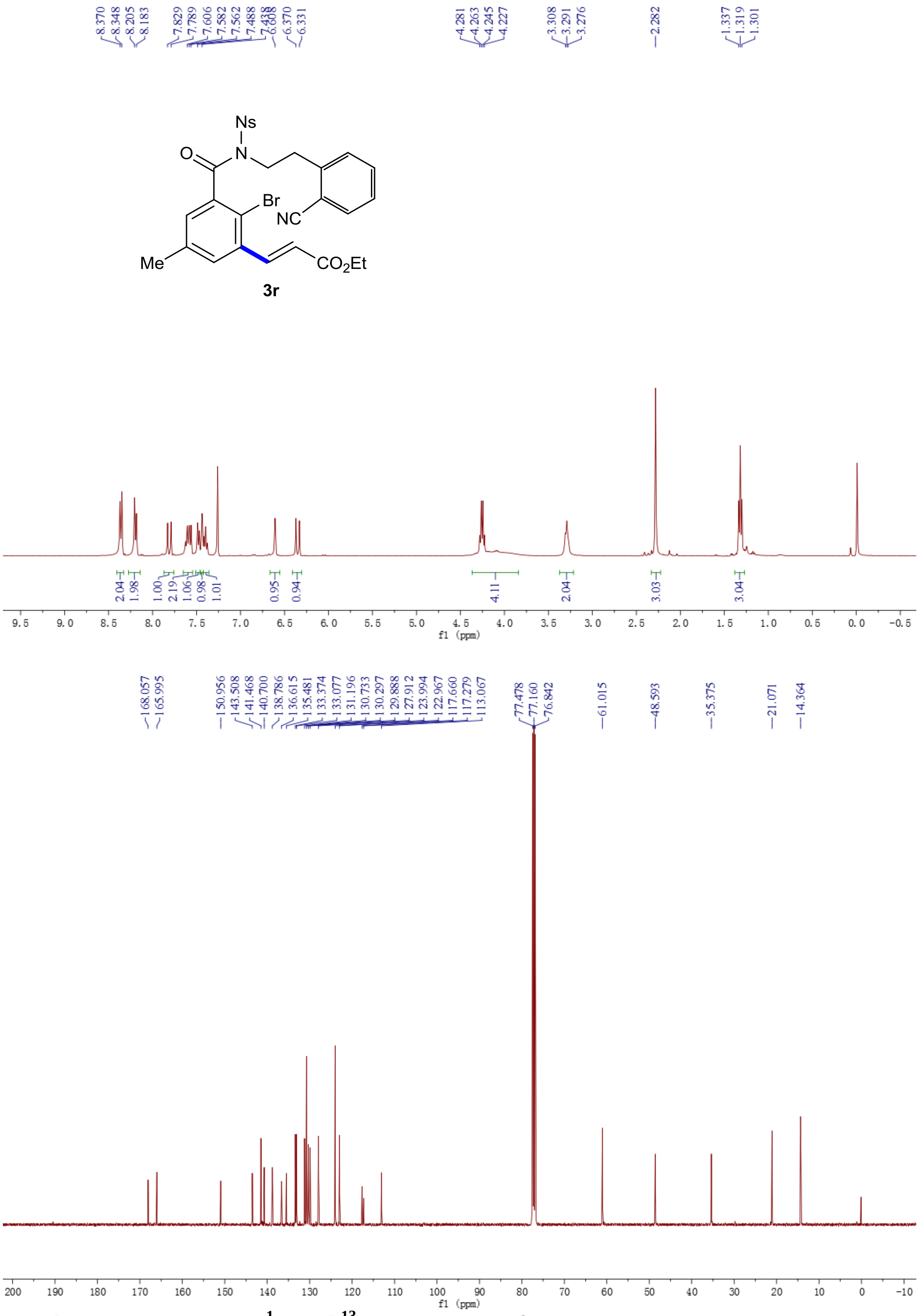

Supplementary Figure 58. ^{1}H and ^{13}C NMR spectra for 3r

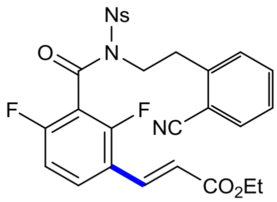

3s_mono

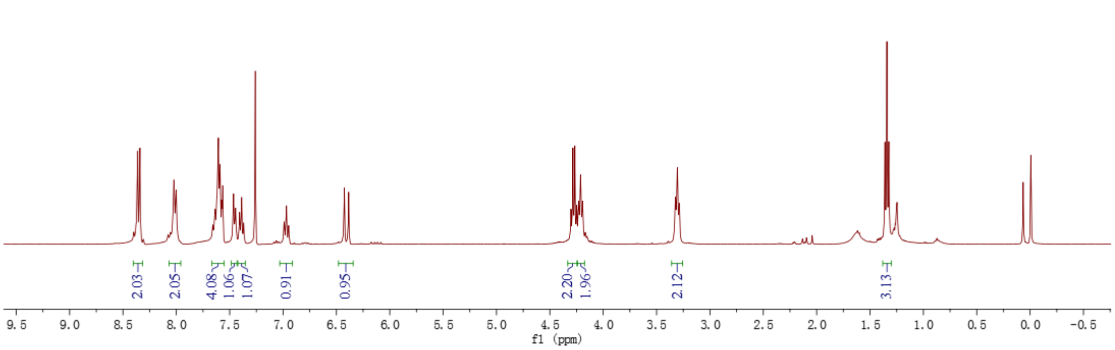

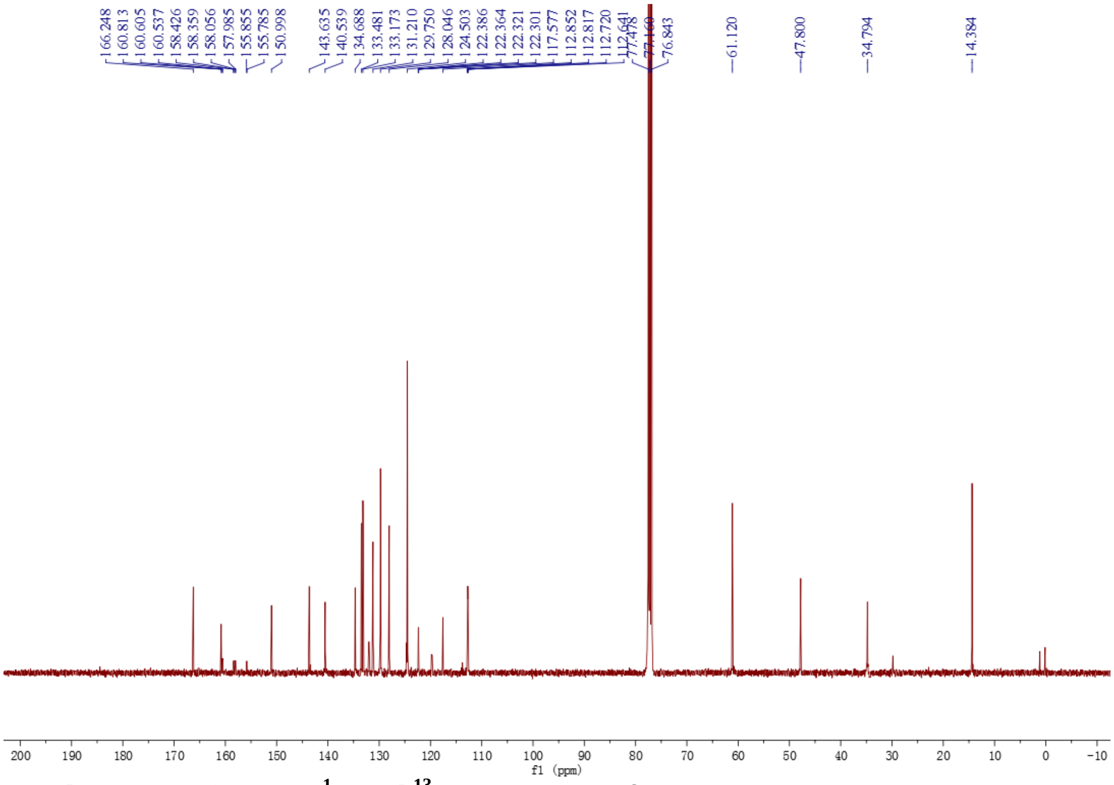

Supplementary Figure 59. ^{1}H and ^{13}C NMR spectra for 3s$_{mono}$

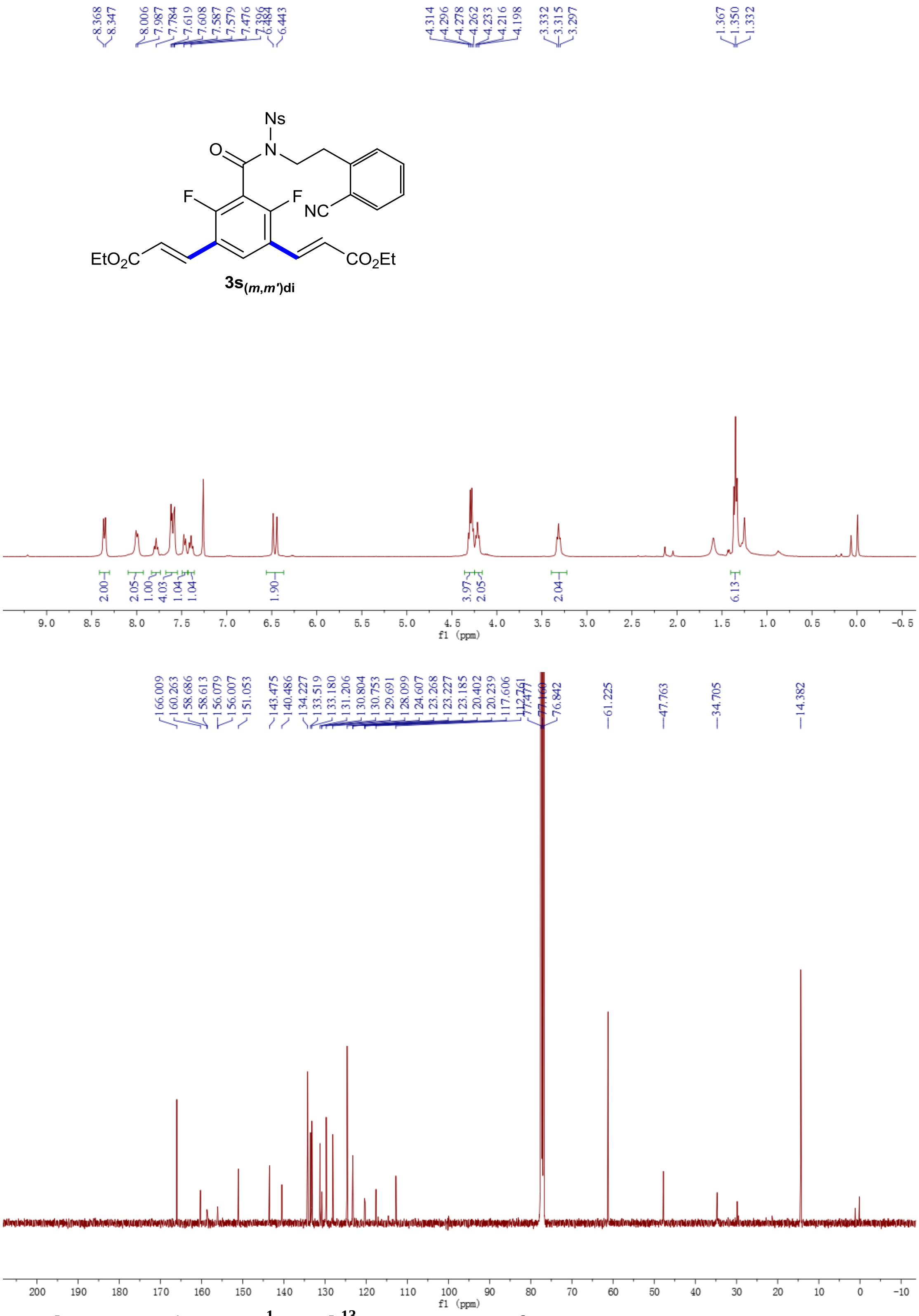

Supplementary Figure 60. ^{1}H and ^{13}C NMR spectra for 3s$_{(m,m')di}$

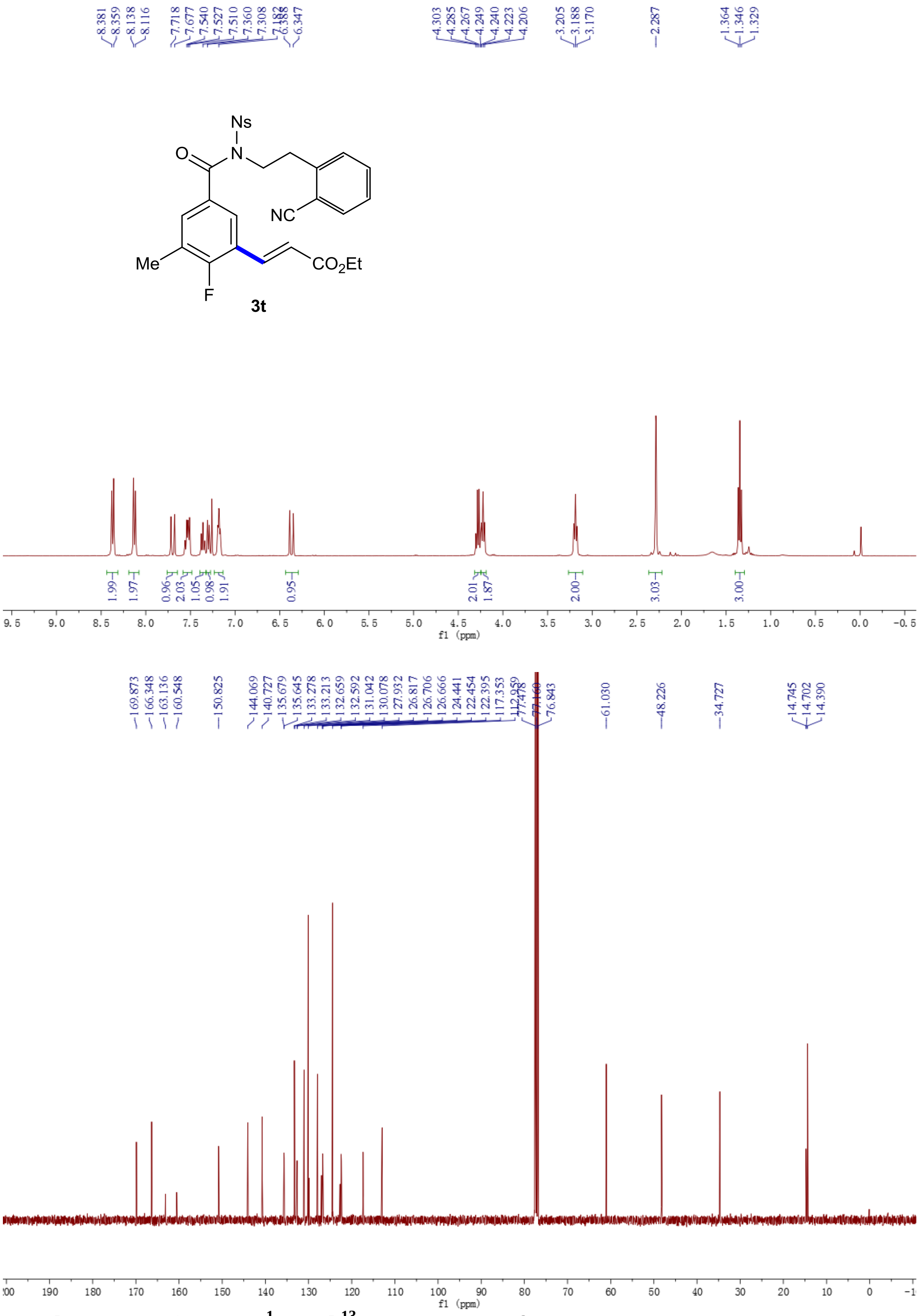

Supplementary Figure 61. ^{1}H and ^{13}C NMR spectra for 3t

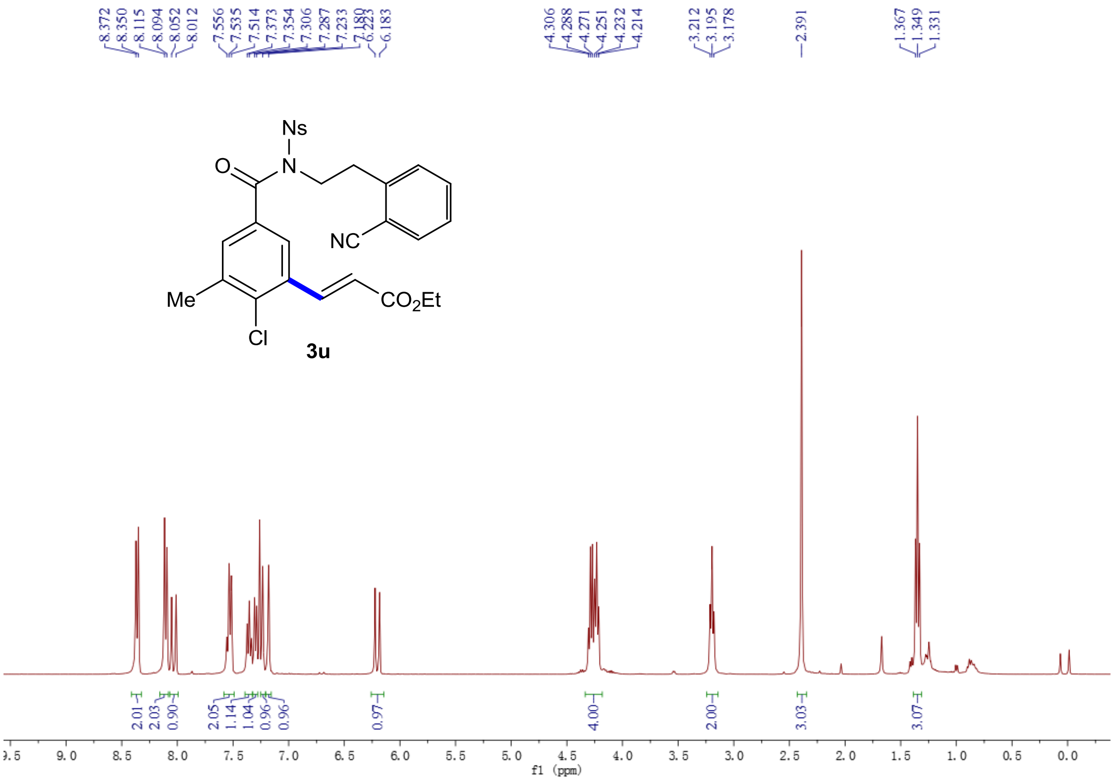

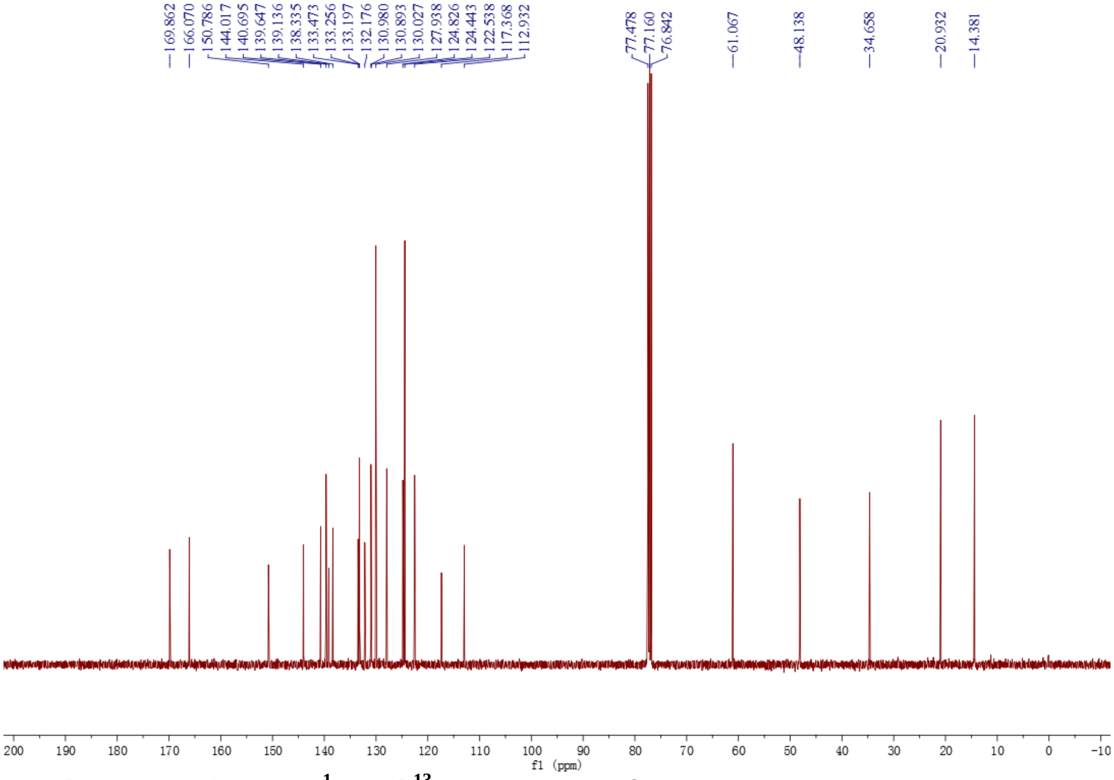

Supplementary Figure 62. ^{1}H and ^{13}C NMR spectra for 3u

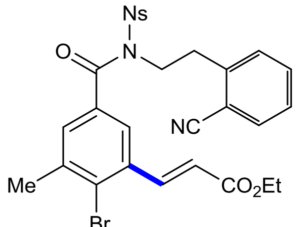

3v

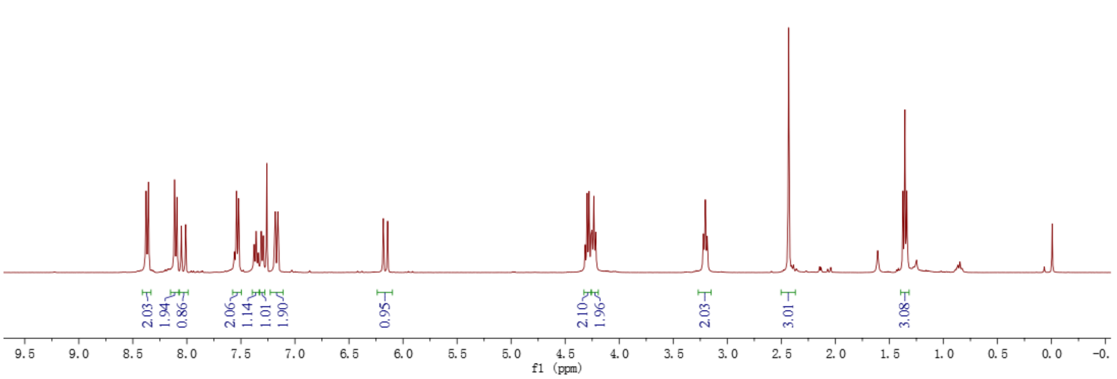

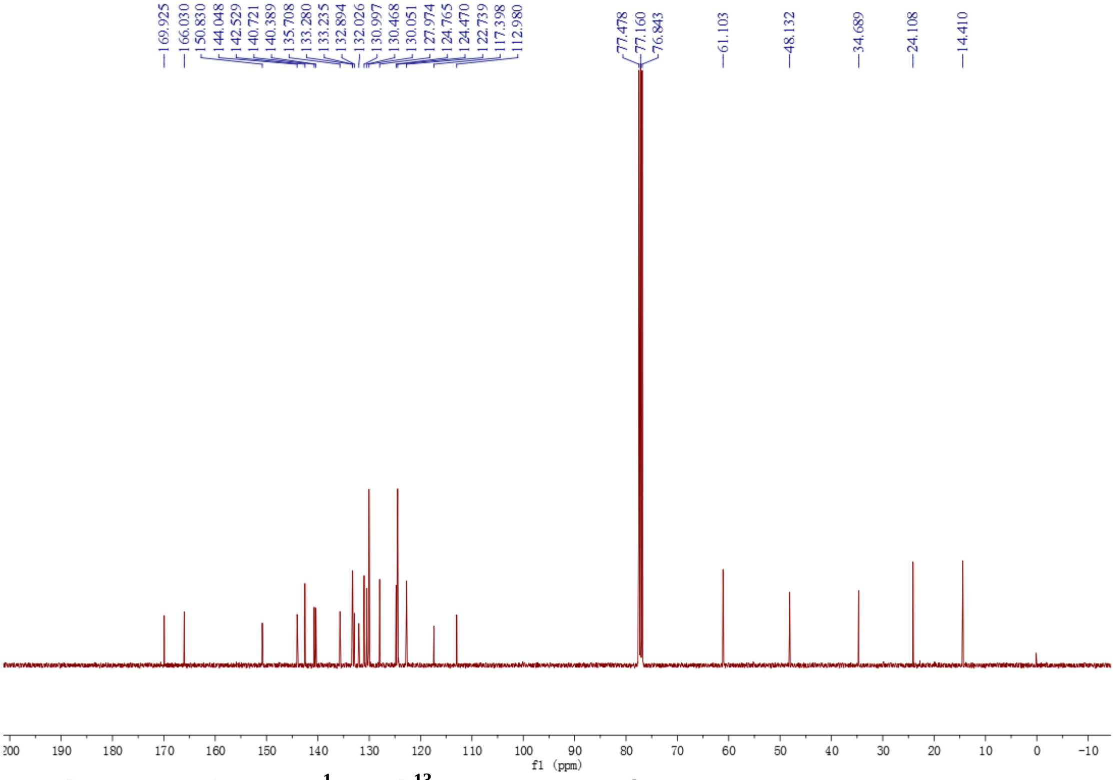

Supplementary Figure 63. ^{1}H and ^{13}C NMR spectra for 3v

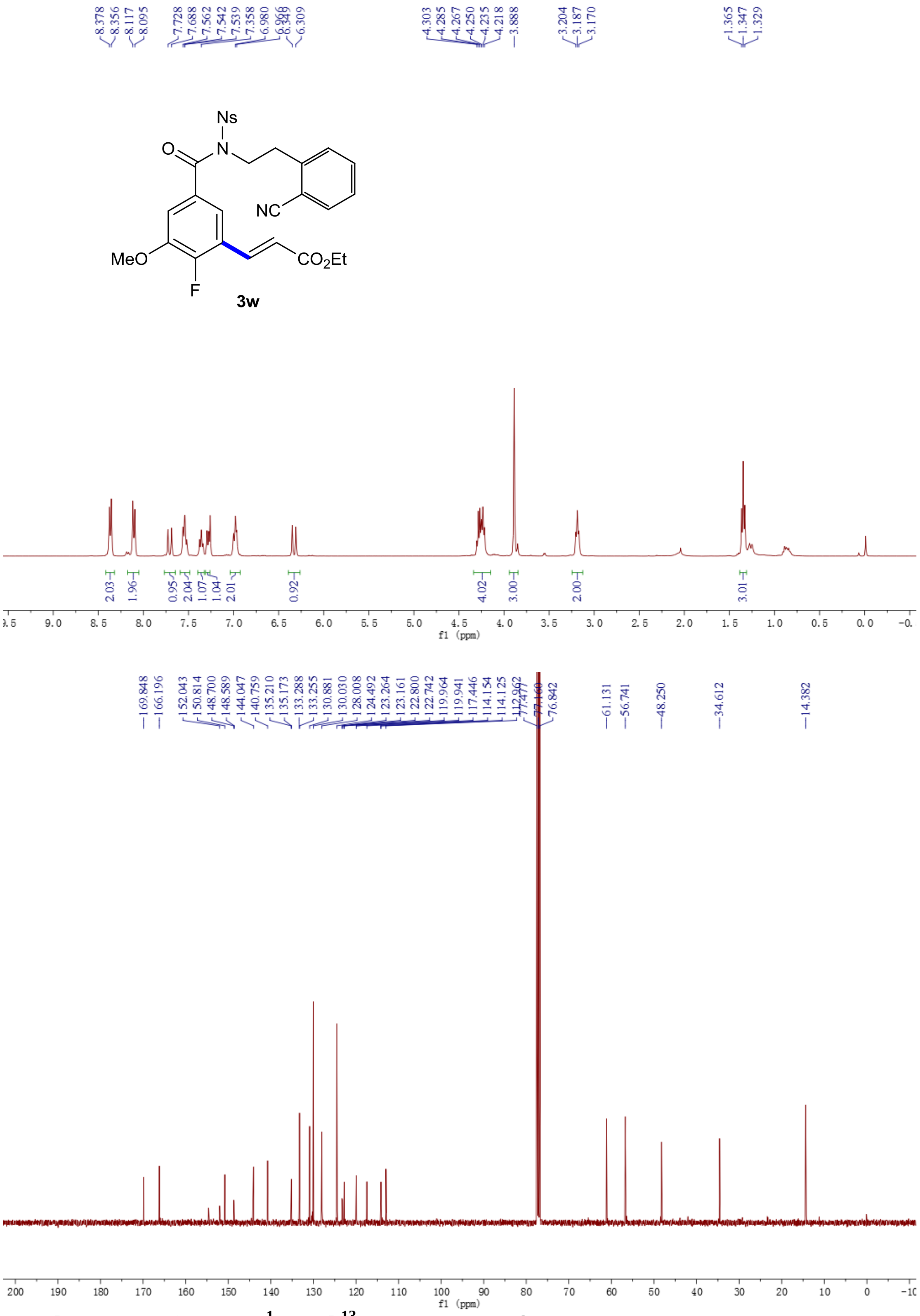

Supplementary Figure 64. ^{1}H and ^{13}C NMR spectra for 3w

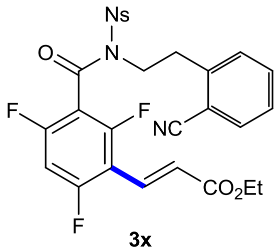

3x

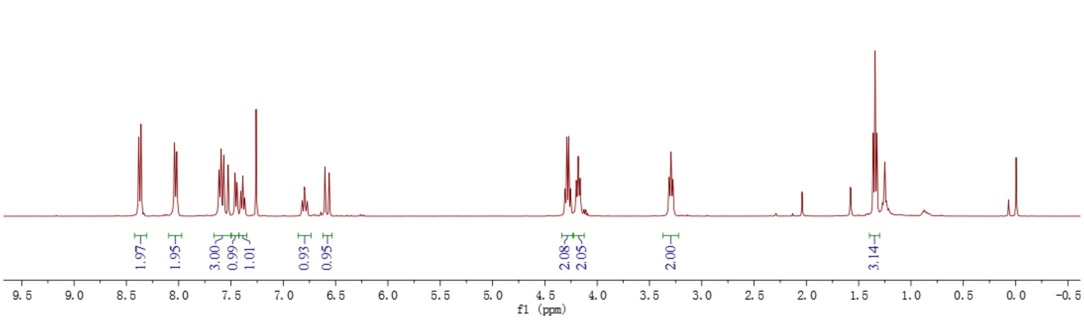

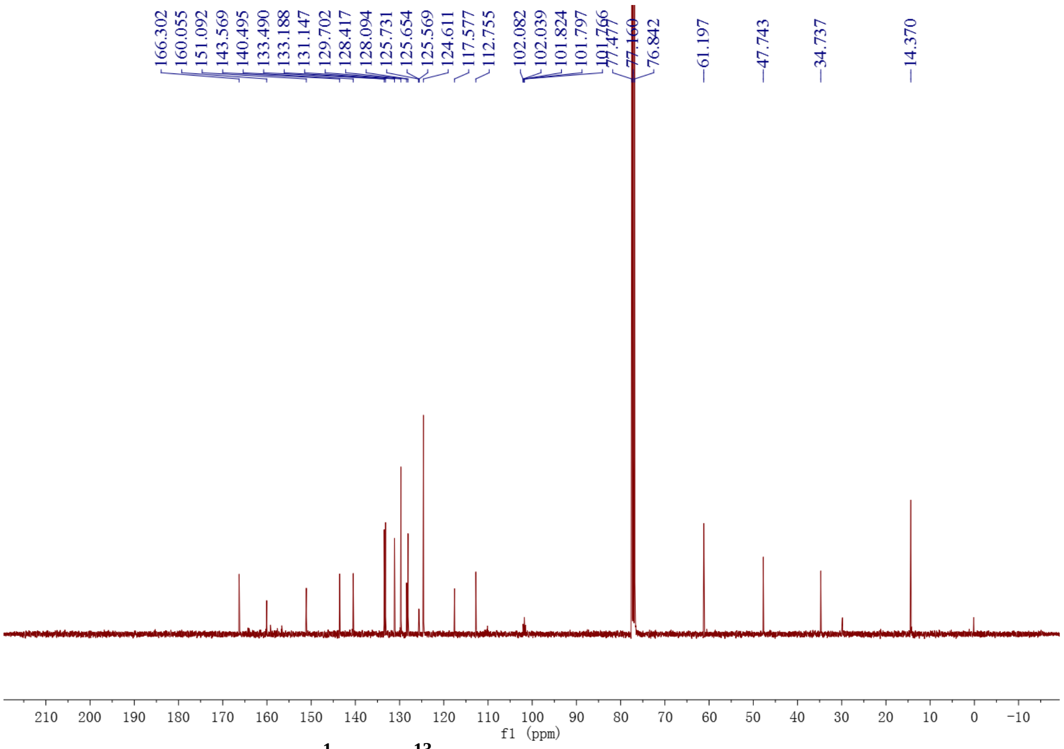

Supplementary Figure 65. ^{1}H and ^{13}C NMR spectra for 3x

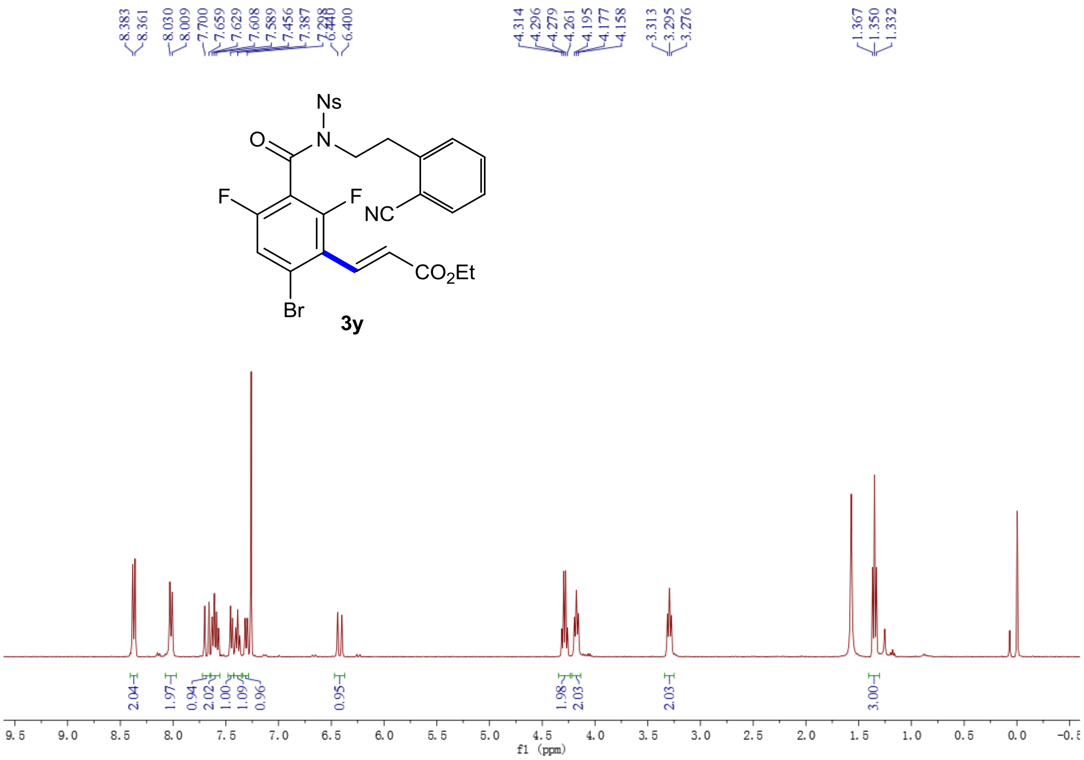

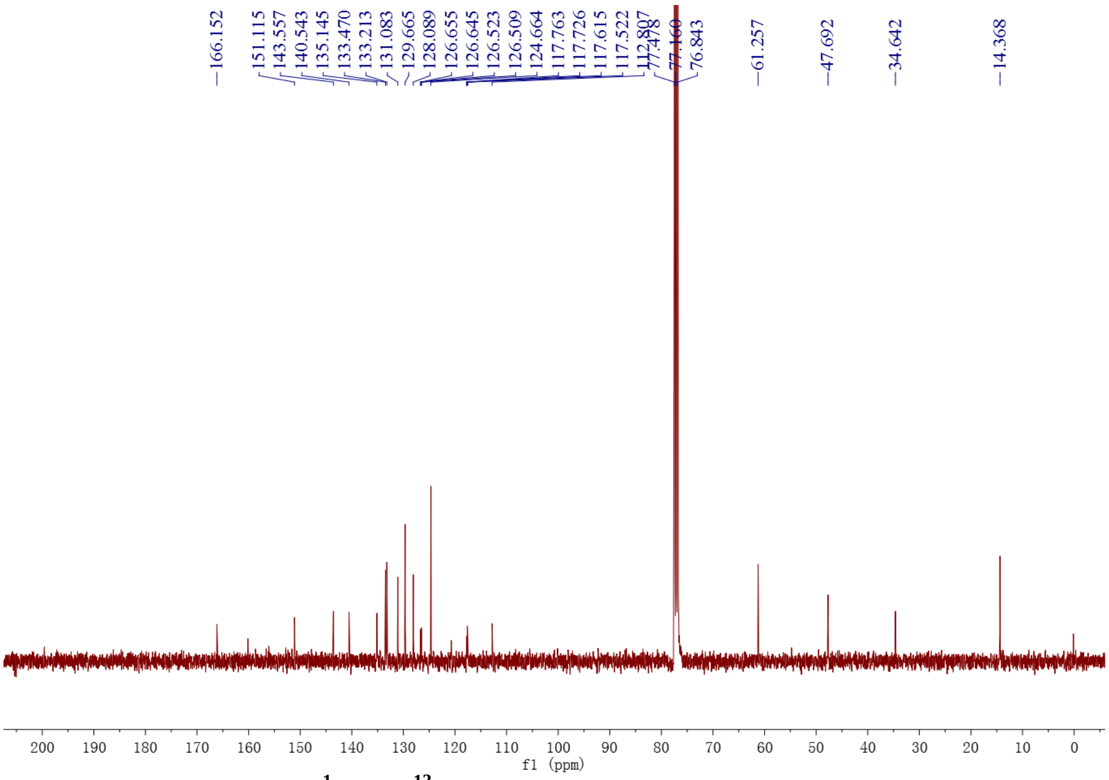

Supplementary Figure 66. ¹H and ¹³C NMR spectra for 3y

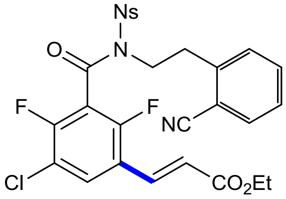

3z

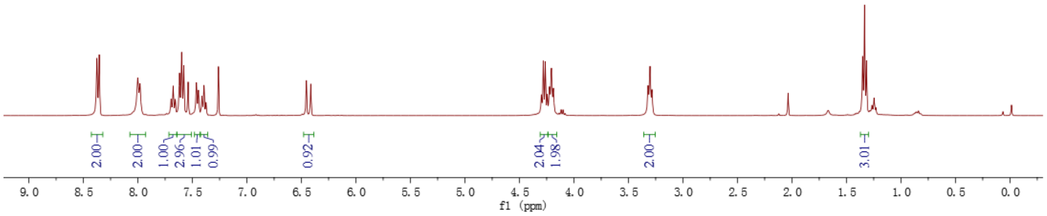

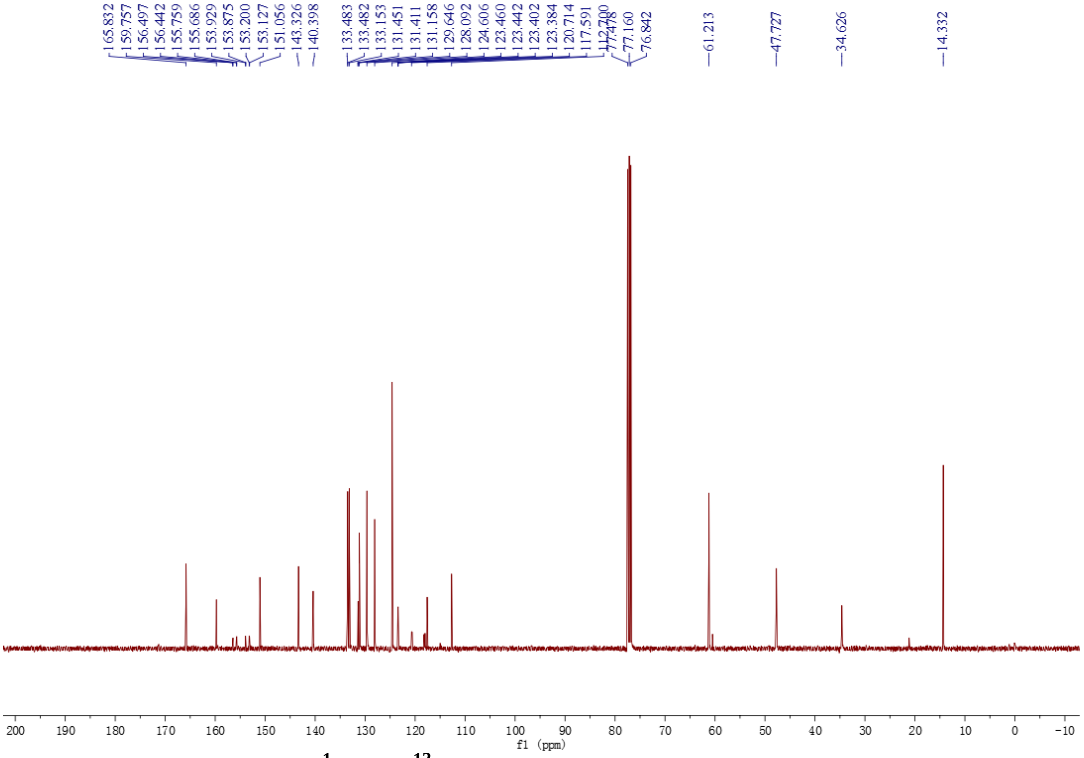

Supplementary Figure 67. ^{1}H and ^{13}C NMR spectra for 3z

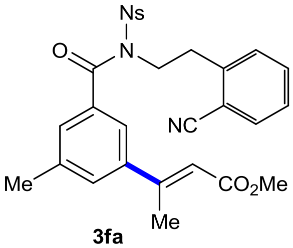

3fa

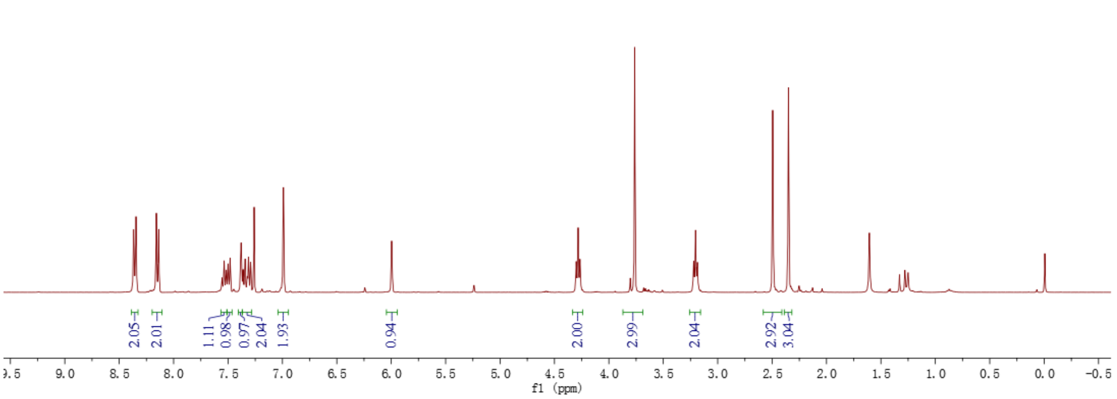

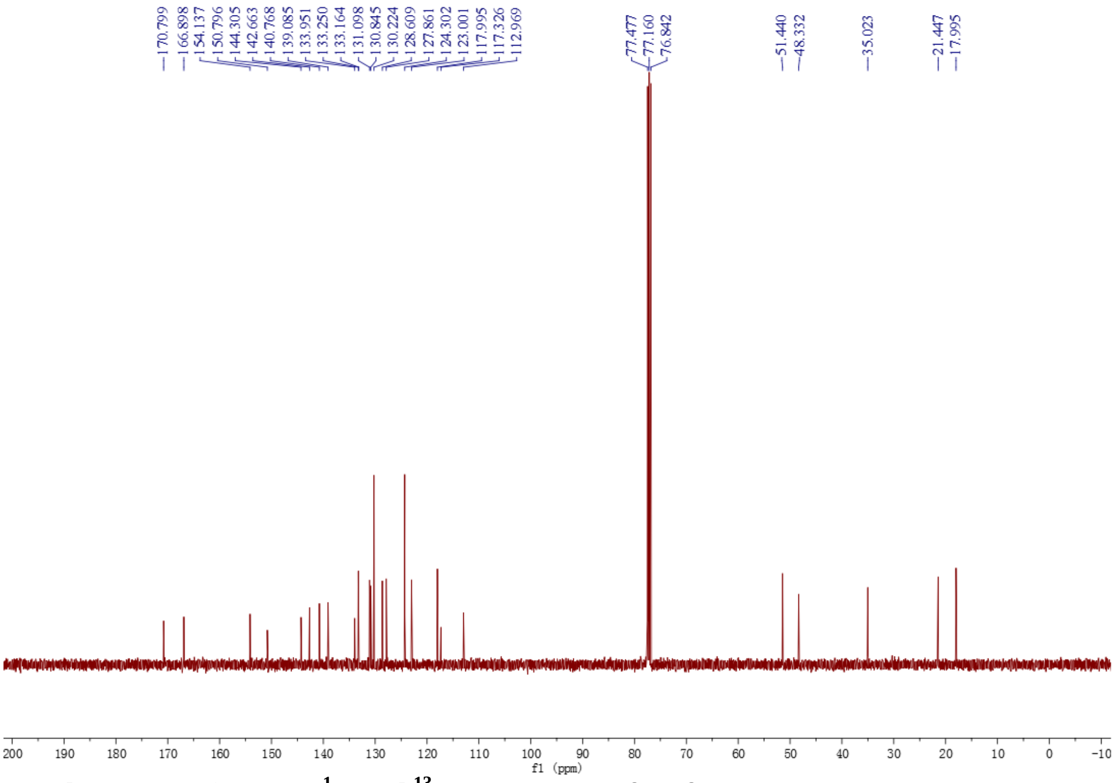

Supplementary Figure 68. [1]**H and** [13]**C NMR spectra for 3fa**

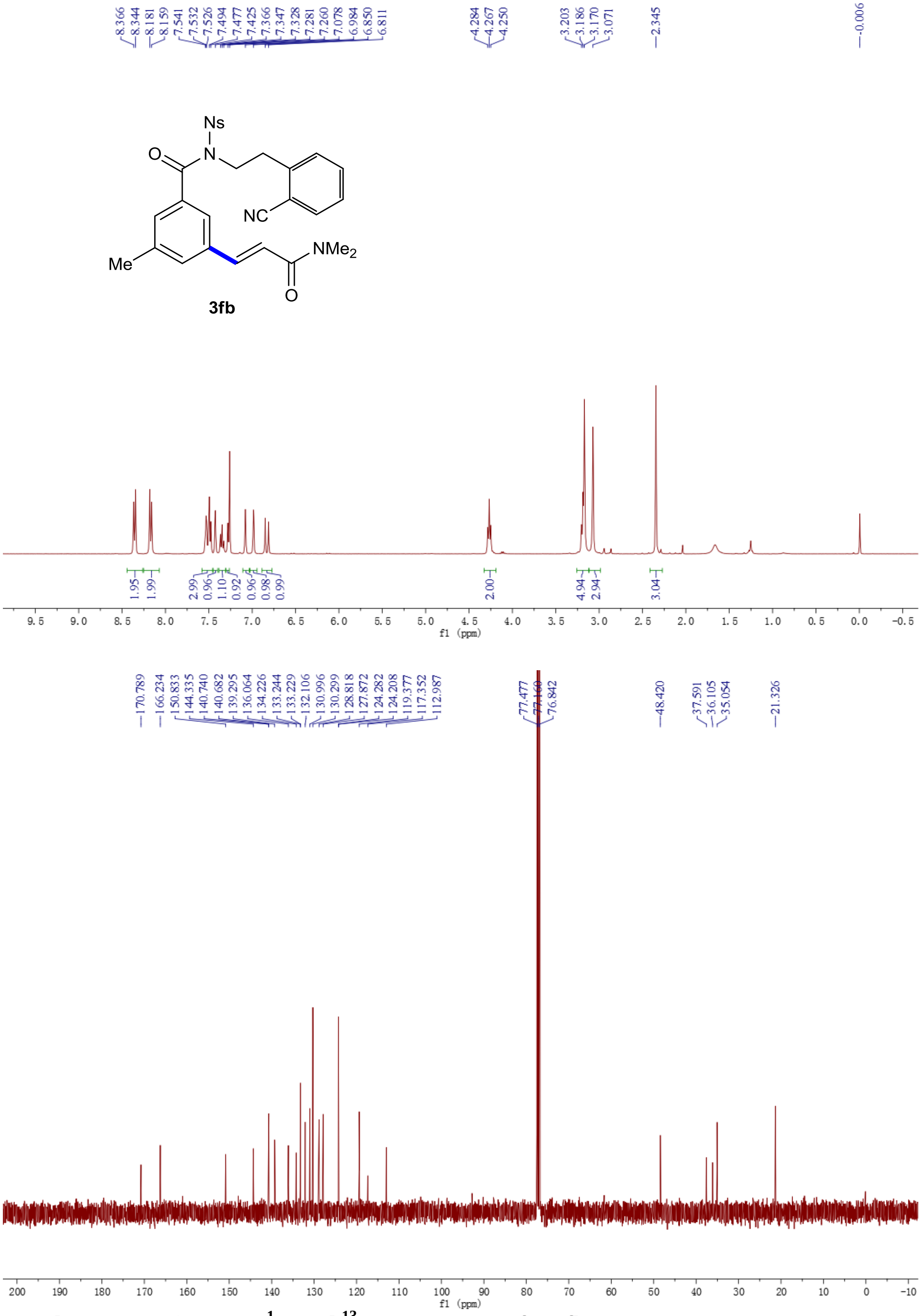

Supplementary Figure 69. ^{1}H and ^{13}C NMR spectra for 3fb

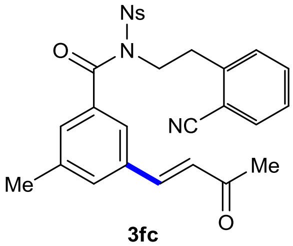

3fc

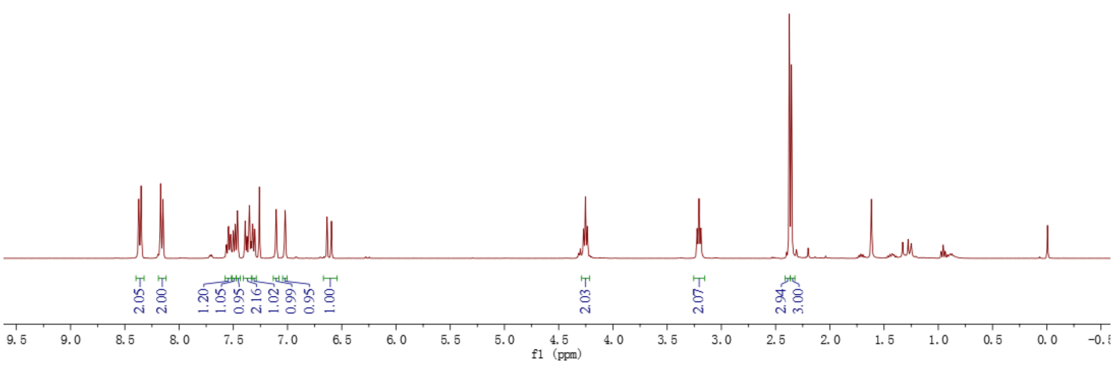

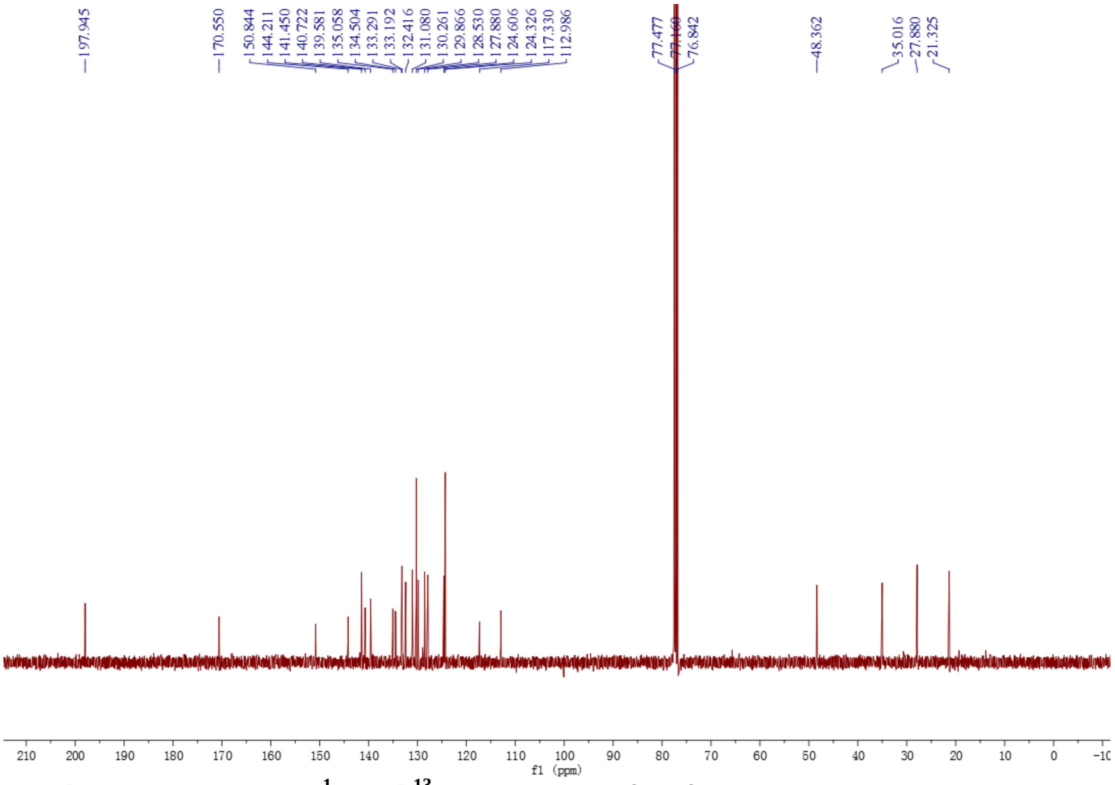

Supplementary Figure 70. ^{1}H and ^{13}C NMR spectra for 3fc

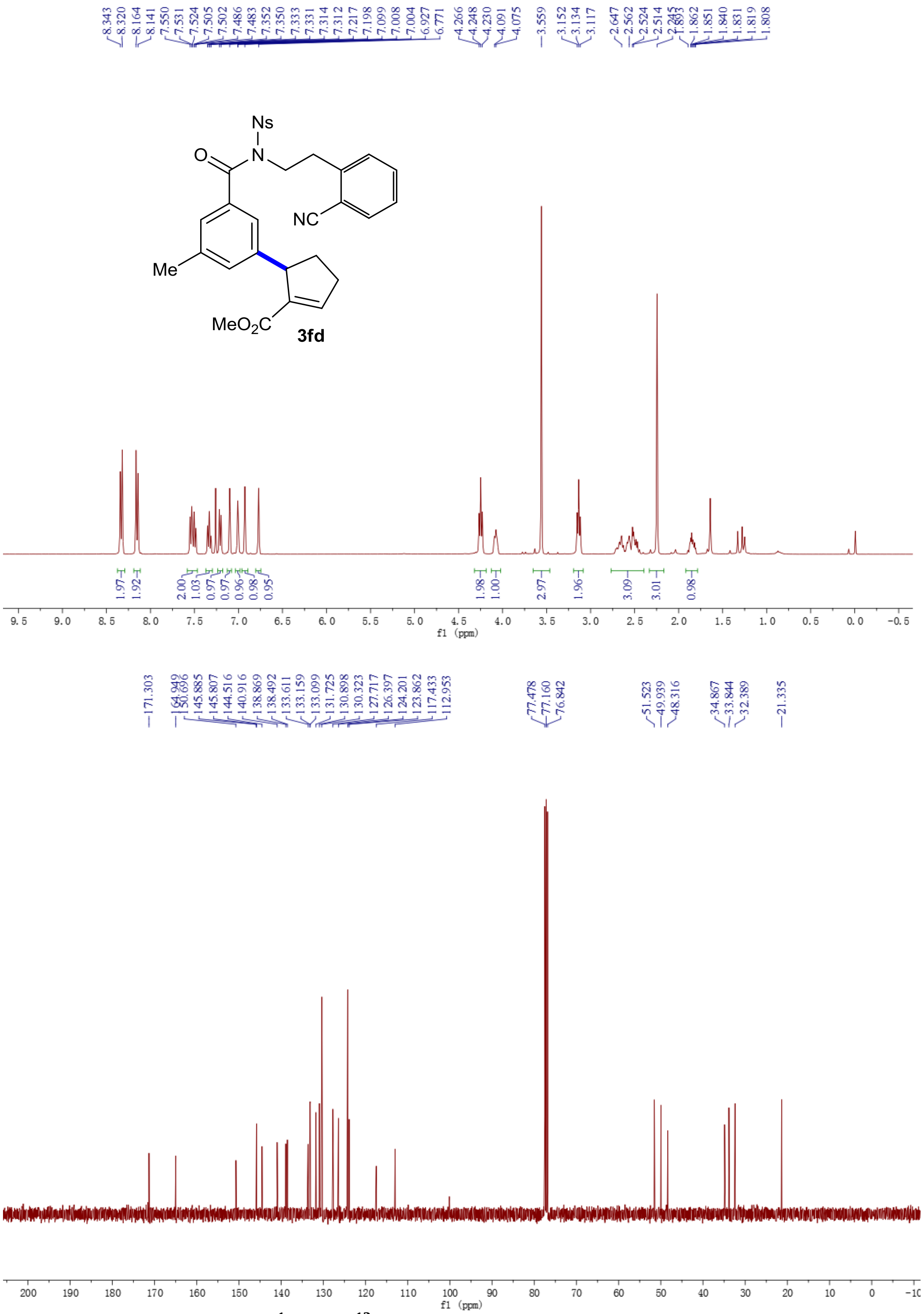

Supplementary Figure 71. ^{1}H and ^{13}C NMR spectra for 3fd

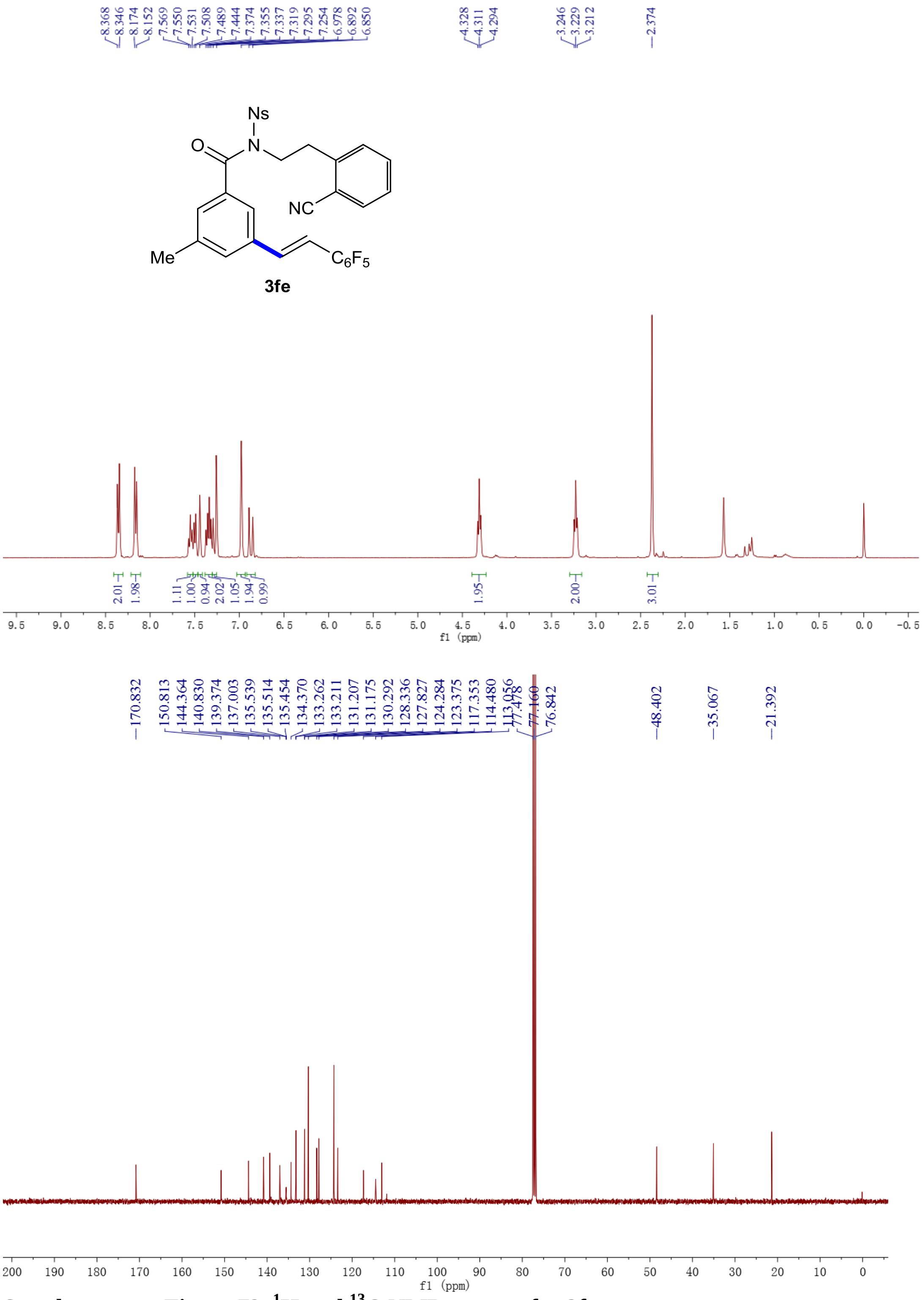

Supplementary Figure 72. ^{1}H and ^{13}C NMR spectra for 3fe

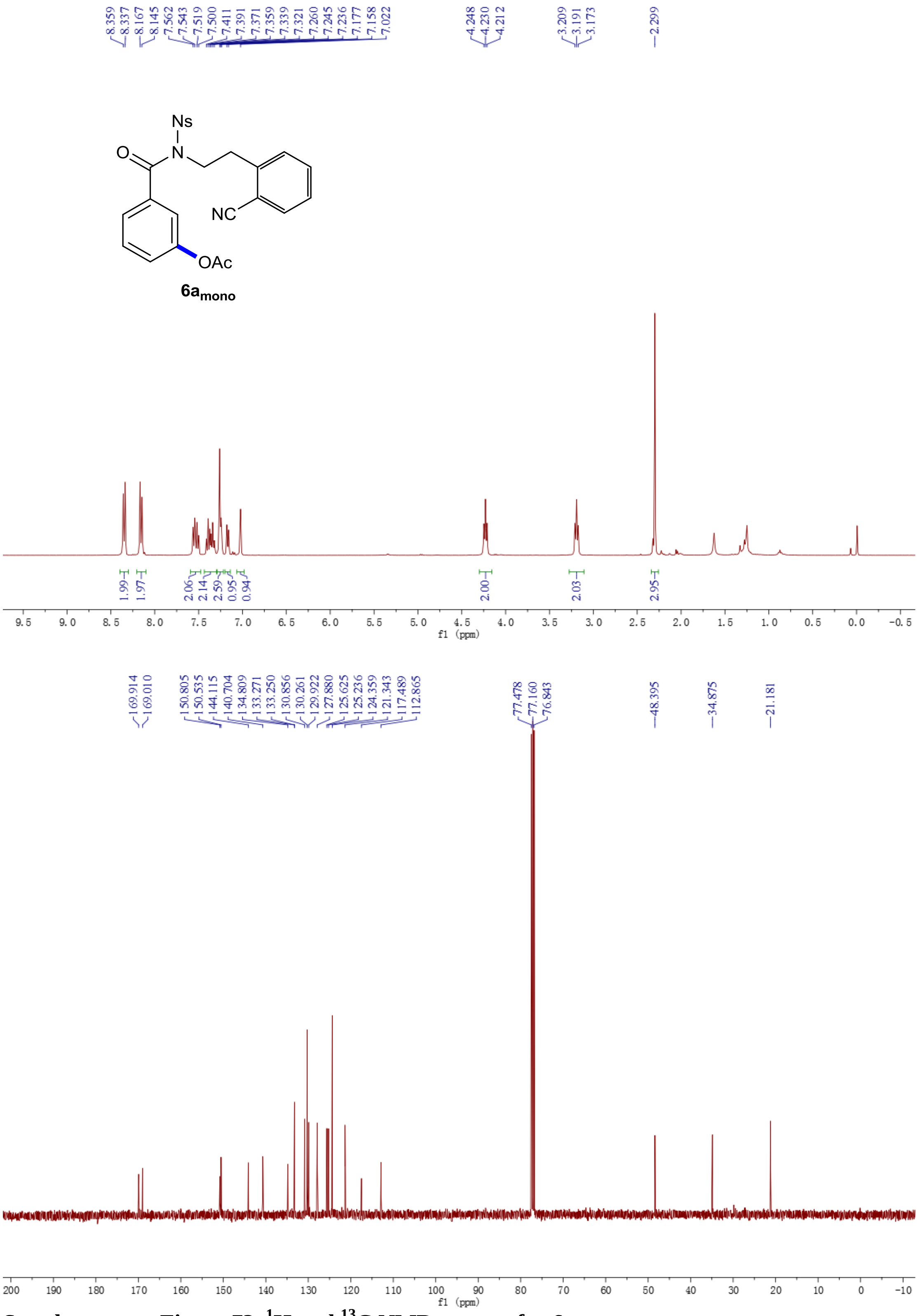

Supplementary Figure 73. ^{1}H and ^{13}C NMR spectra for $6a_{mono}$

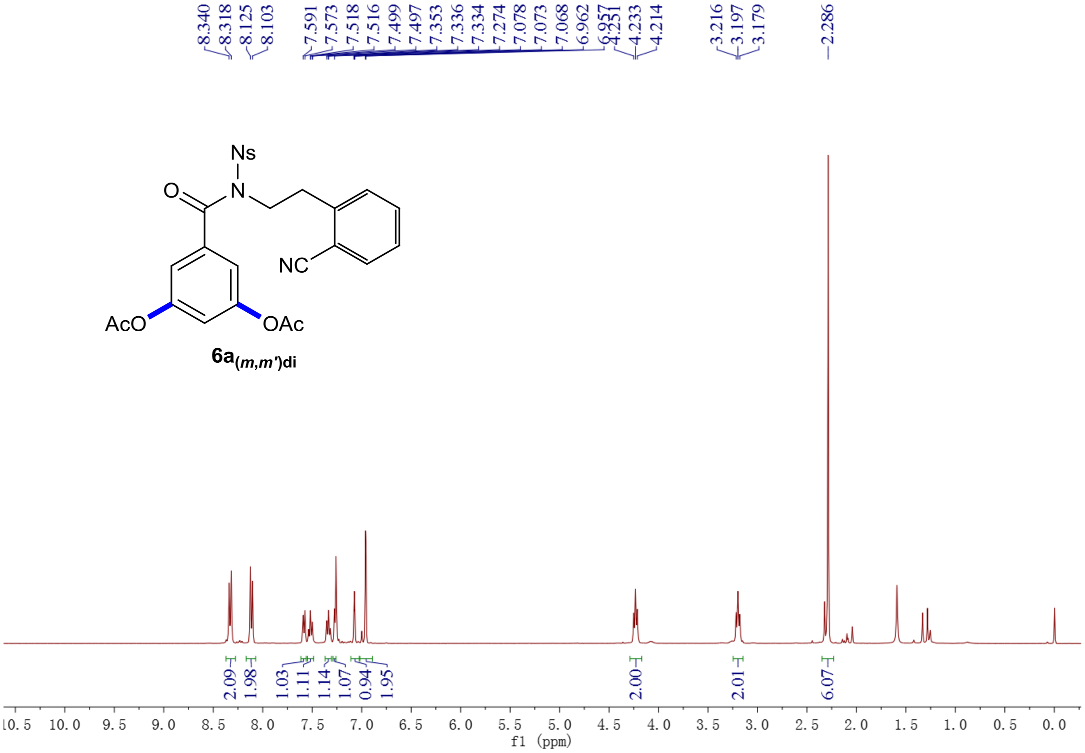

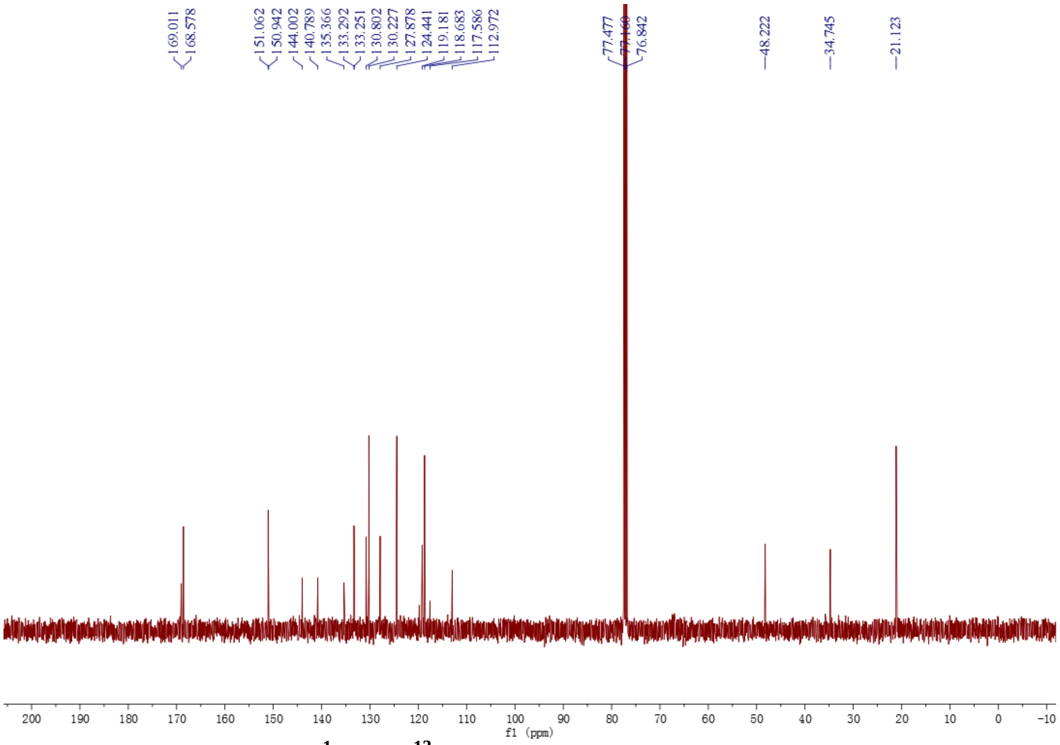

Supplementary Figure 74. ^{1}**H and** ^{13}**C NMR spectra for 6a**$_{(m,m')di}$

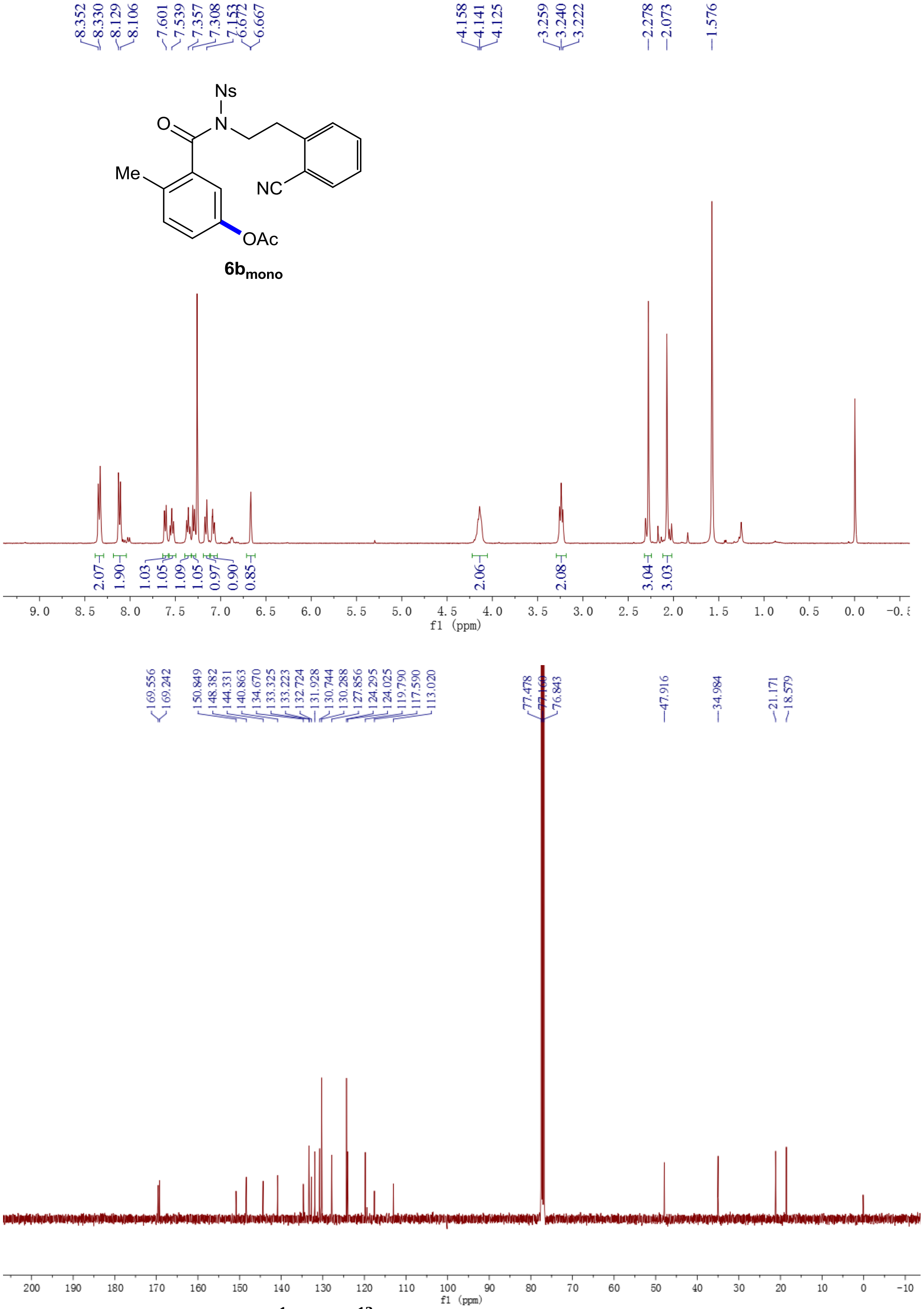

Supplementary Figure 75. ^{1}H and ^{13}C NMR spectra for 6b$_{mono}$

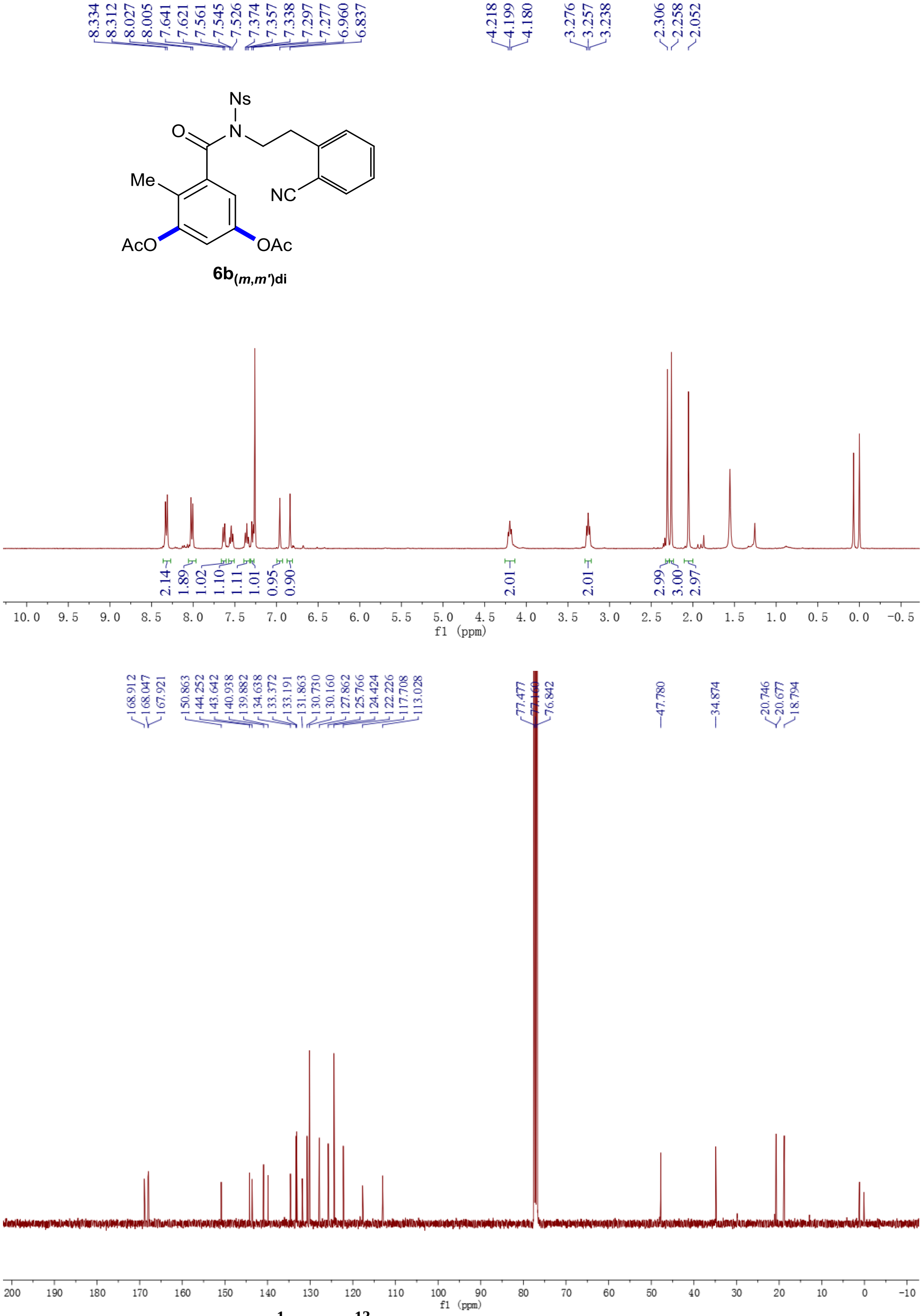

Supplementary Figure 76. ^{1}H and ^{13}C NMR spectra for 6b$_{(m,m')di}$

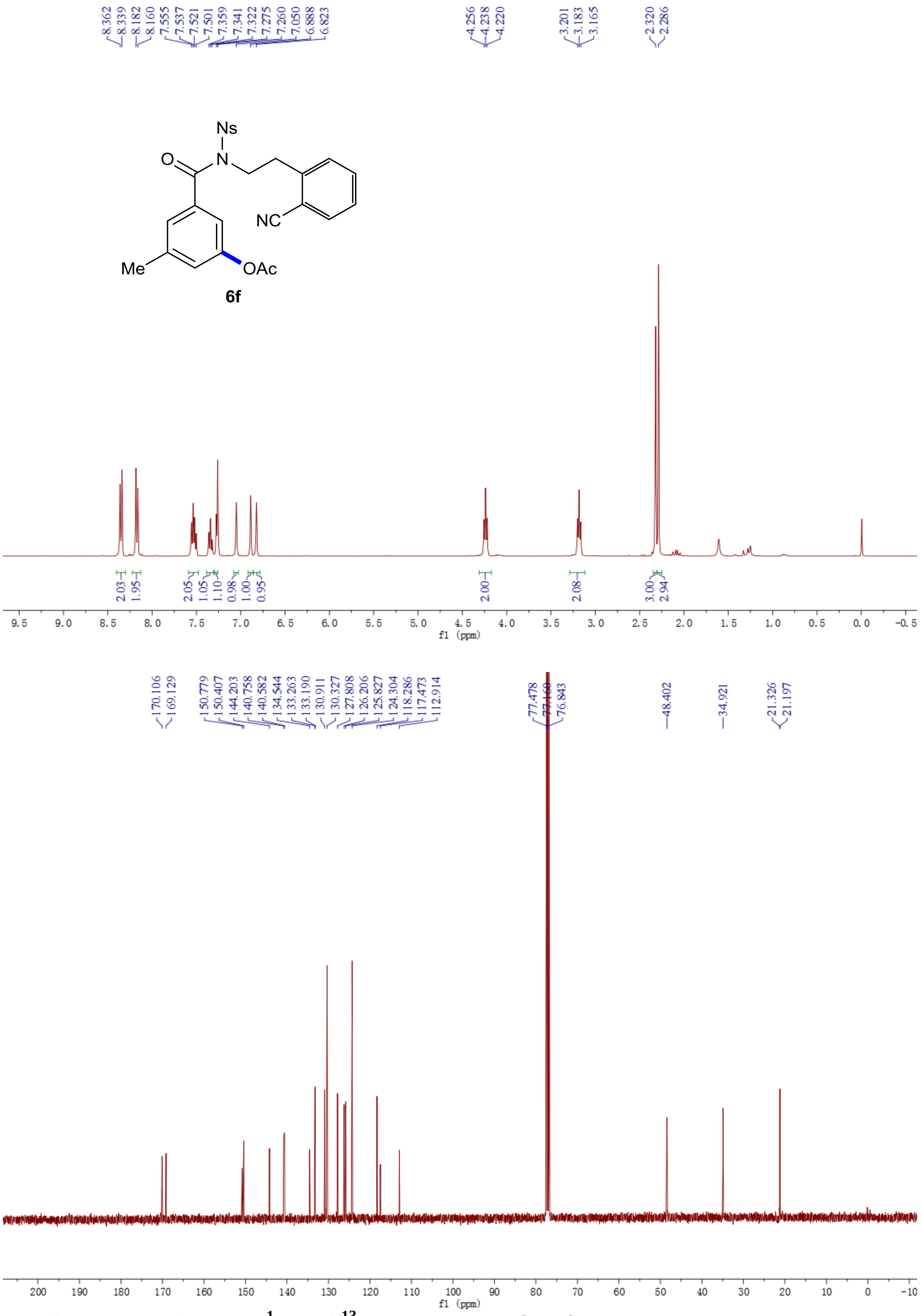

Supplementary Figure 77. ^{1}H and ^{13}C NMR spectra for 6f

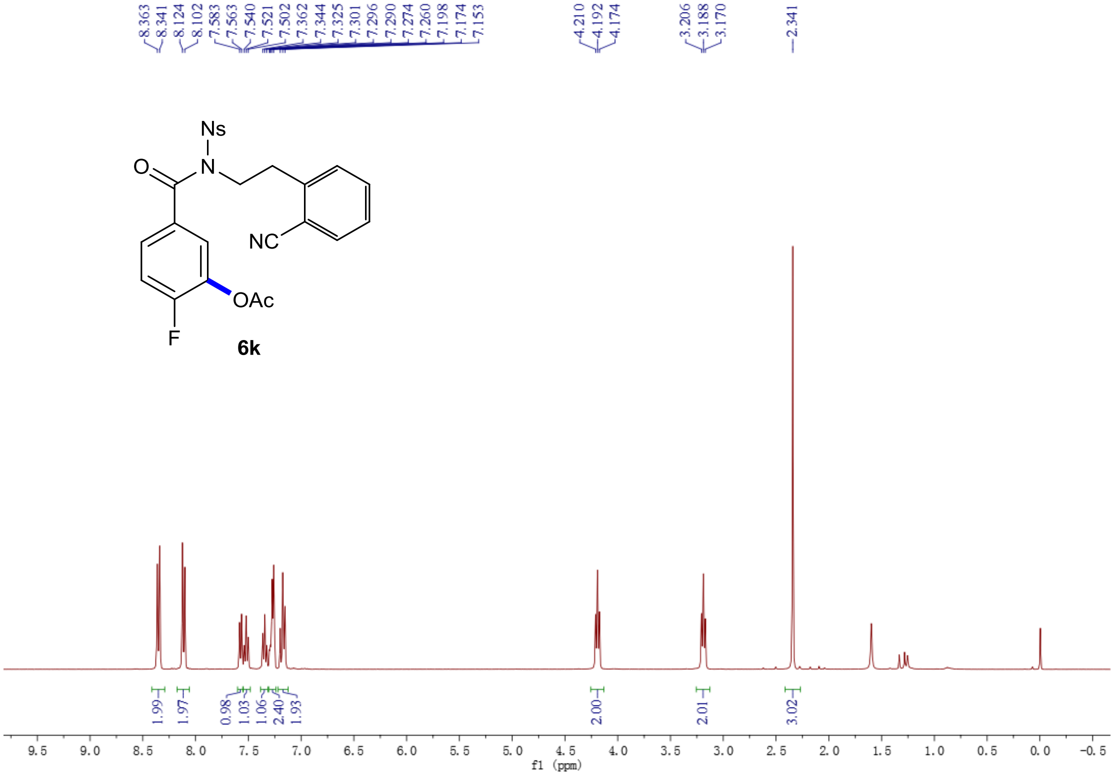

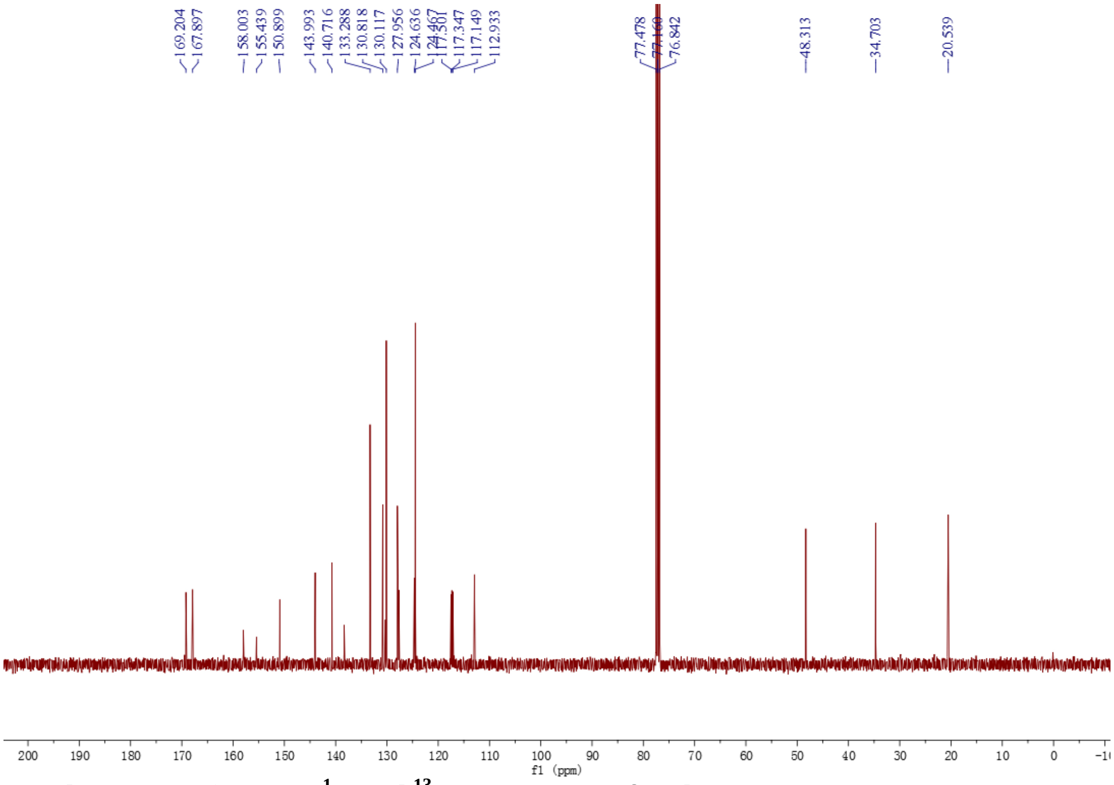

Supplementary Figure 78. ^{1}H and ^{13}C NMR spectra for 6k

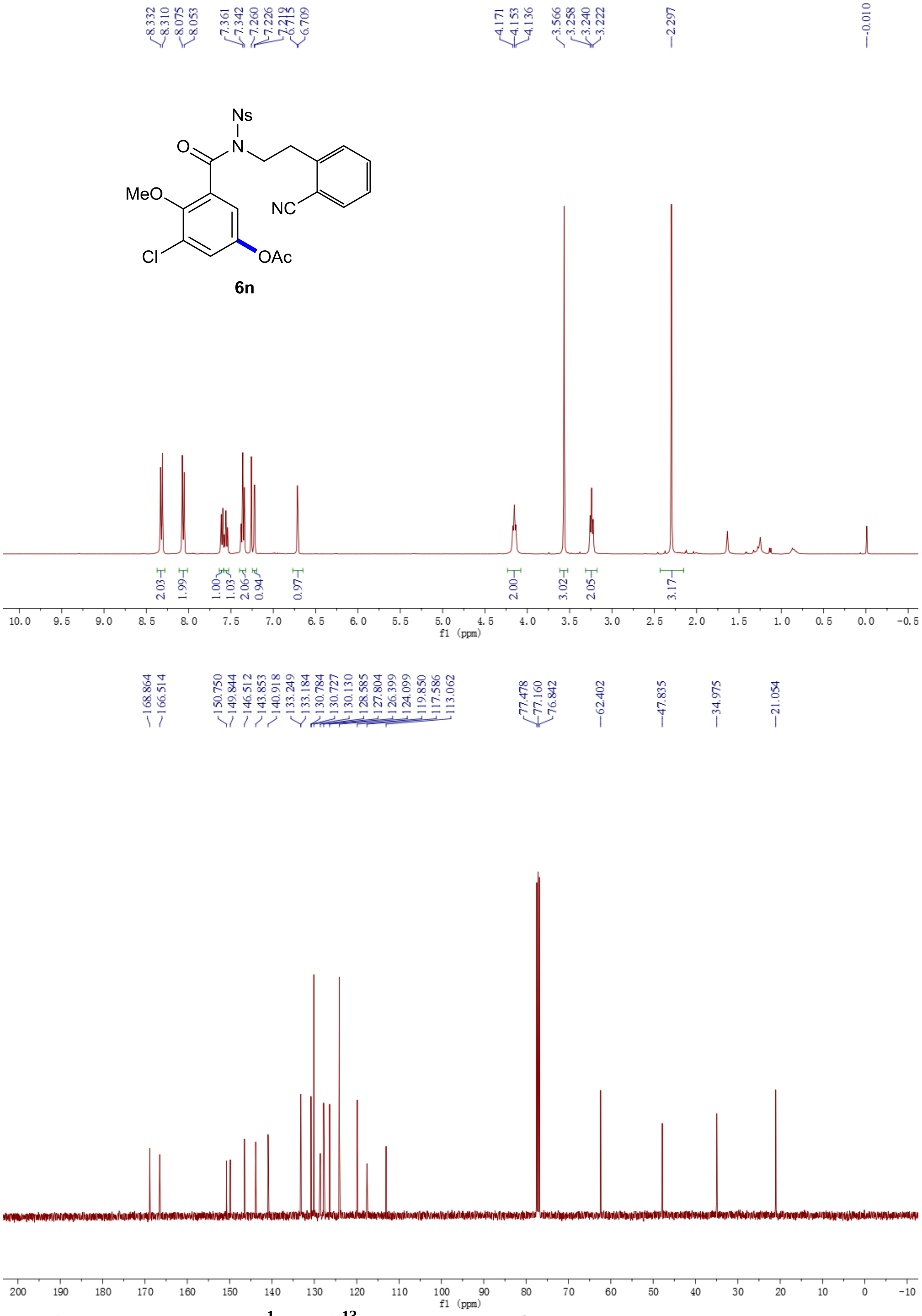

Supplementary Figure 79. ^{1}H and ^{13}C NMR spectra for 6n

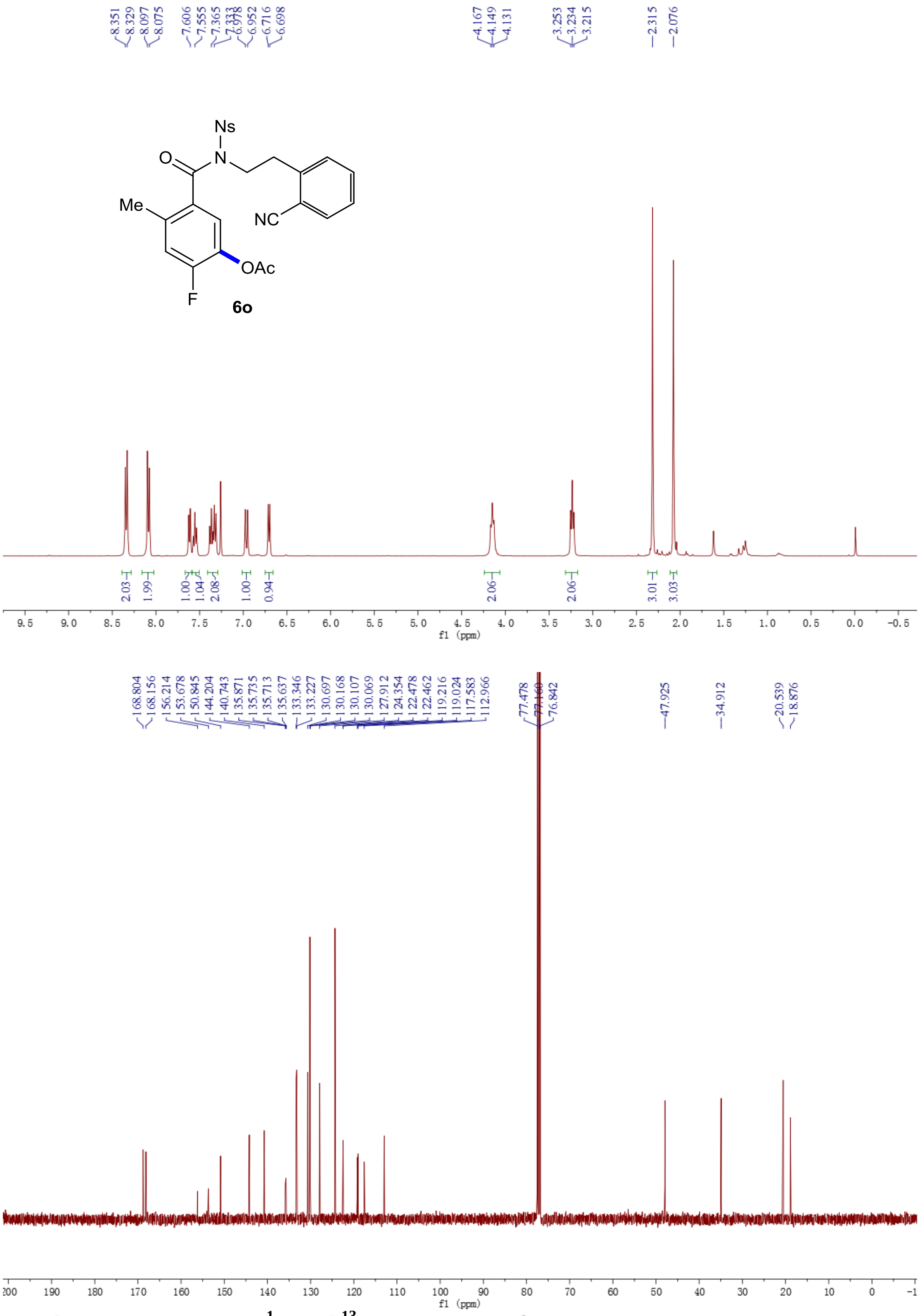

Supplementary Figure 80. ^{1}**H and** ^{13}**C NMR spectra for 6o**

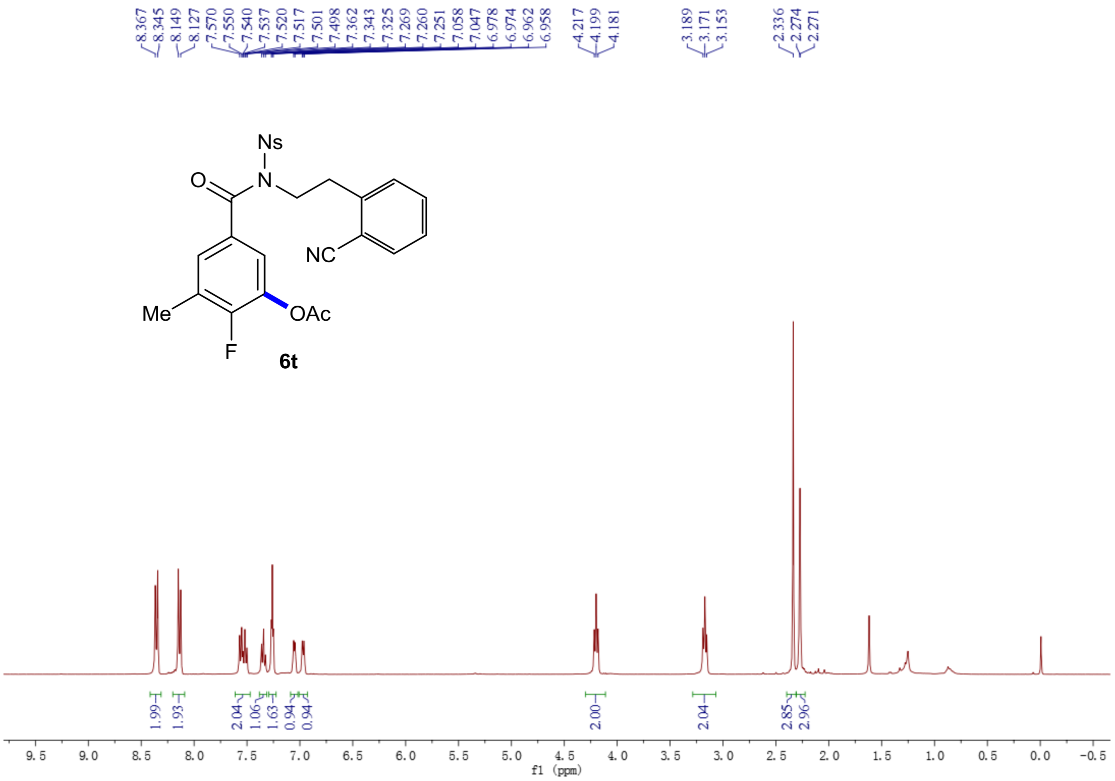

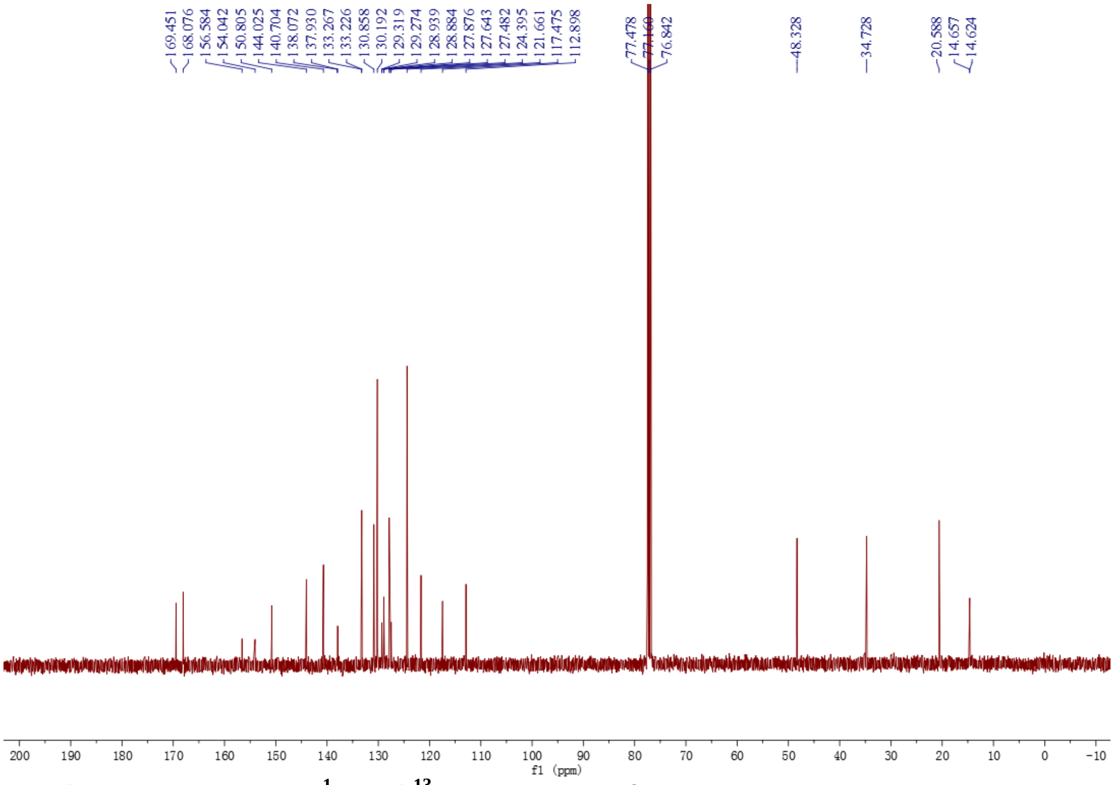

Supplementary Figure 81. ^{1}H and ^{13}C NMR spectra for 6t

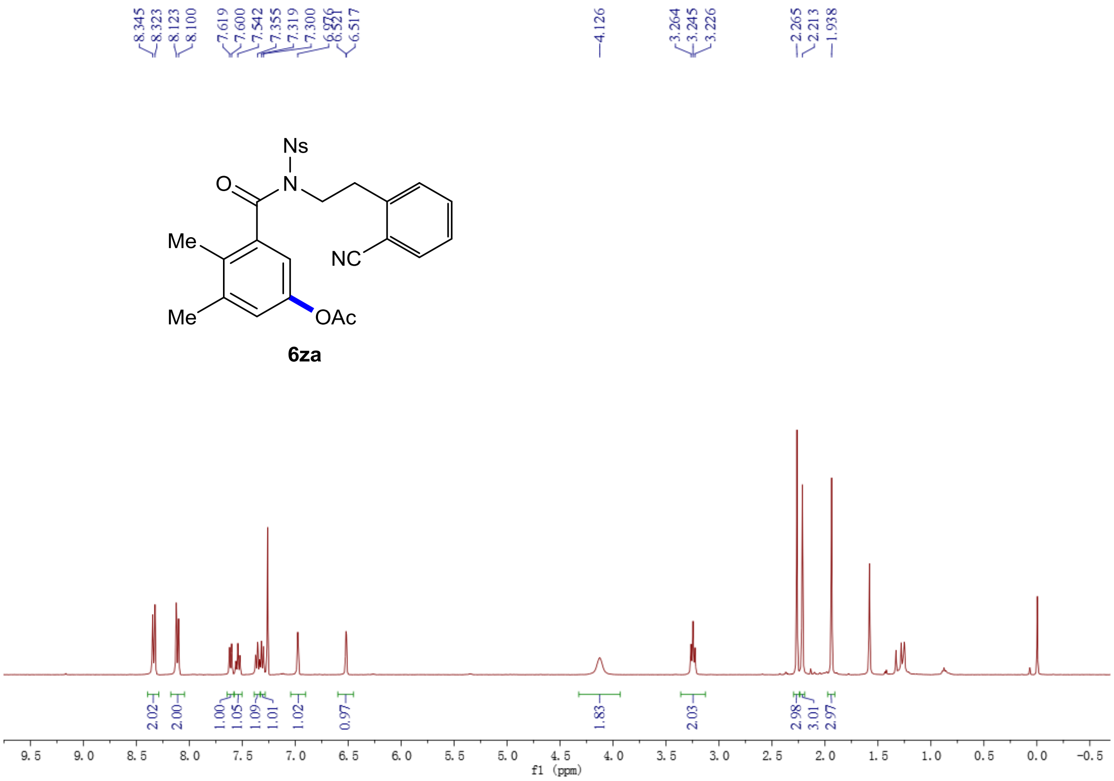

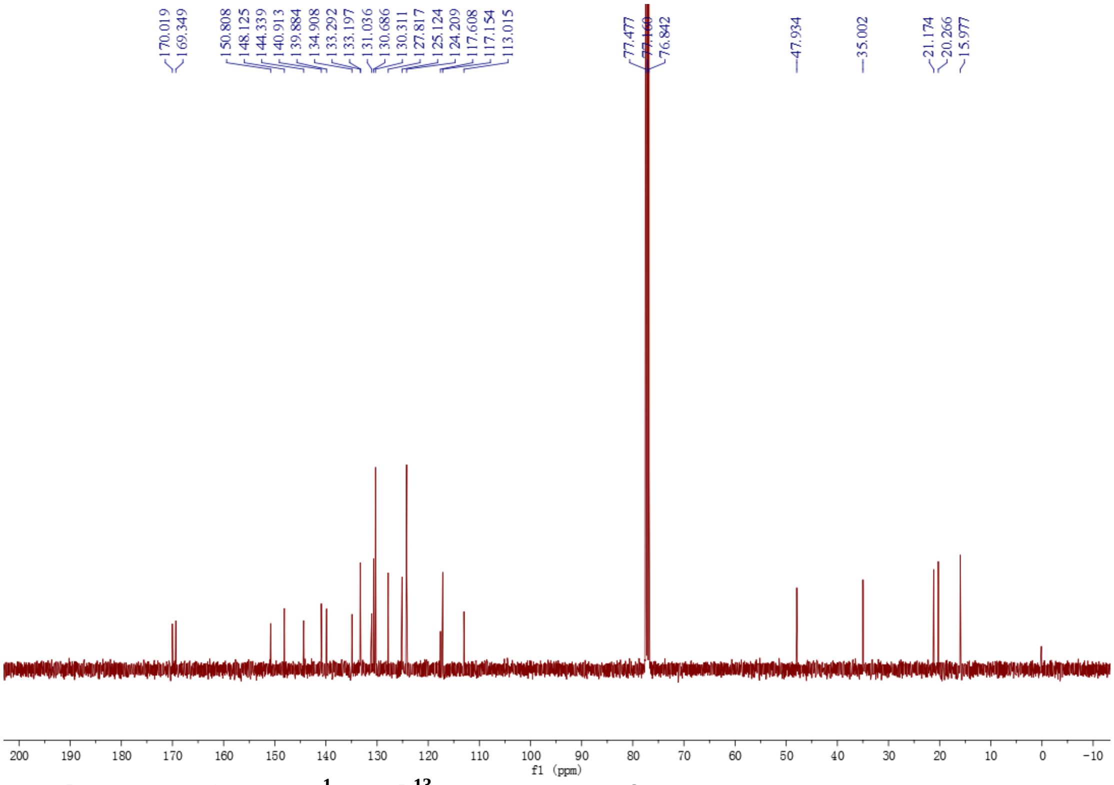

Supplementary Figure 82. ^{1}H and ^{13}C NMR spectra for 6za

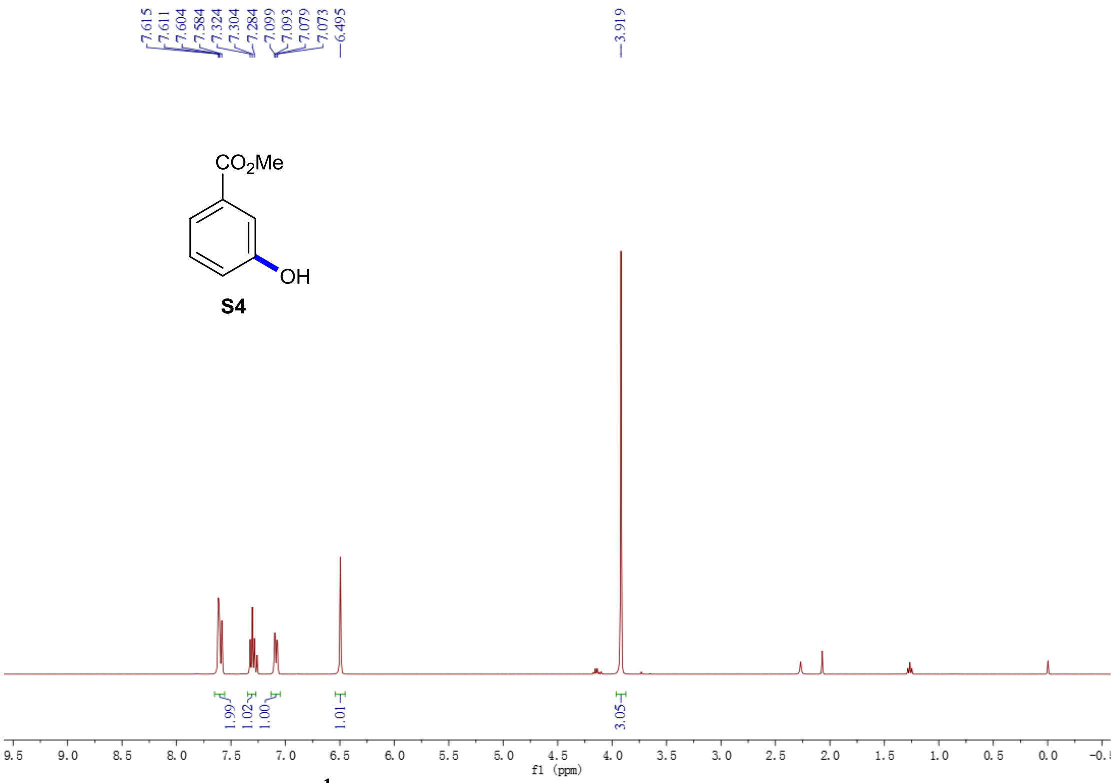

Supplementary Figure 83. ^{1}**H NMR spectra for S4**

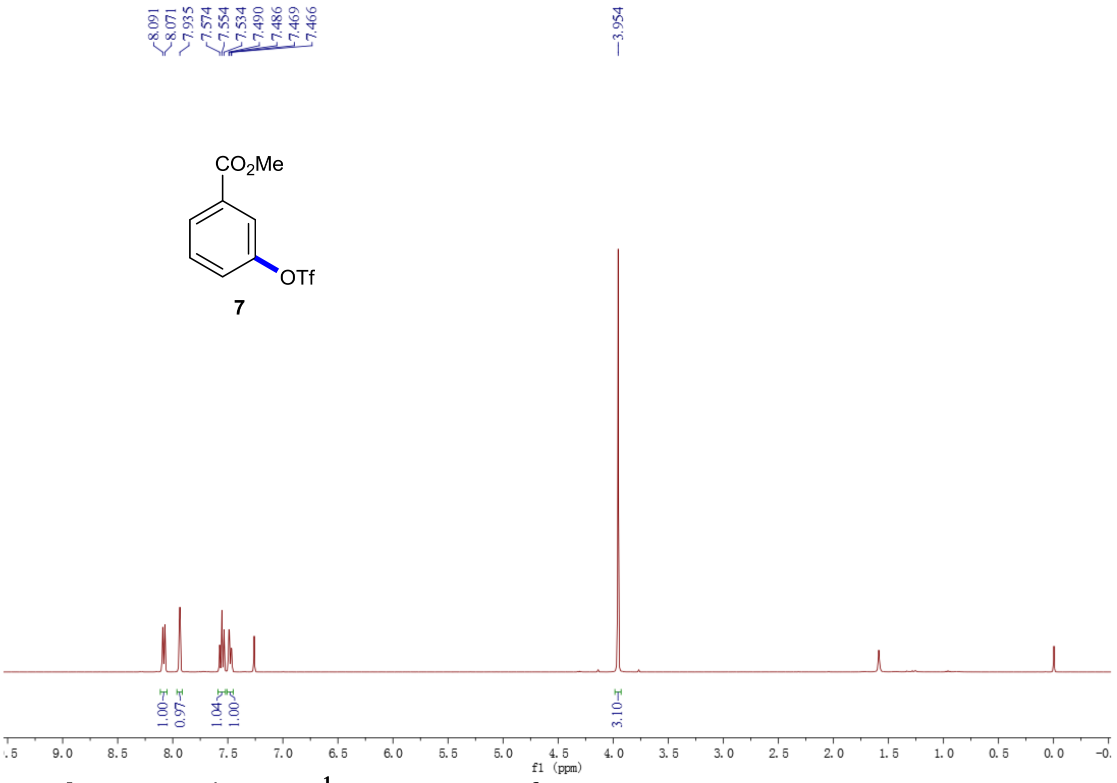

Supplementary Figure 84. ^{1}**H NMR spectra for 7**

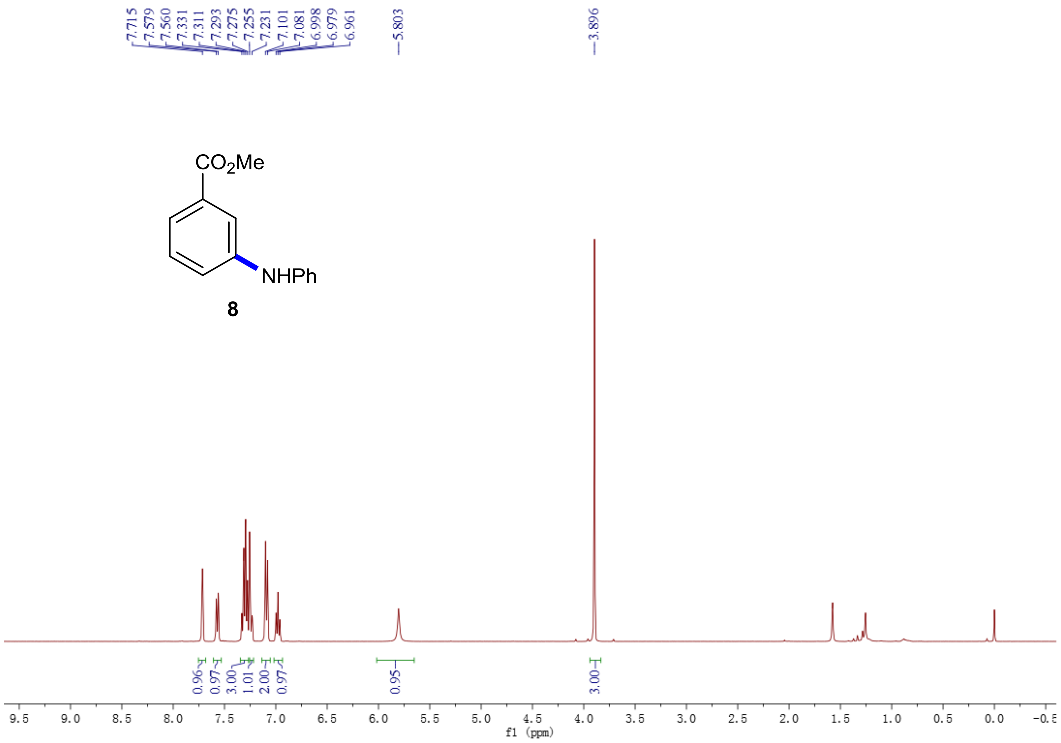

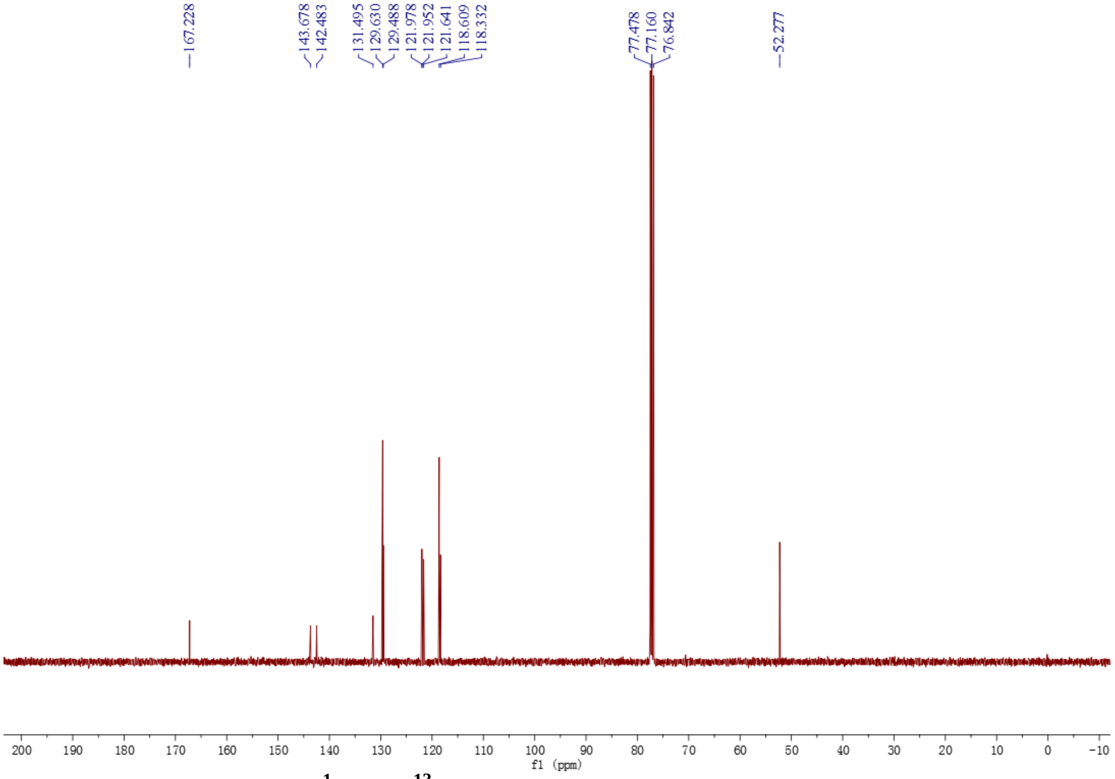

Supplementary Figure 85. ^{1}H and ^{13}C NMR spectra for 8

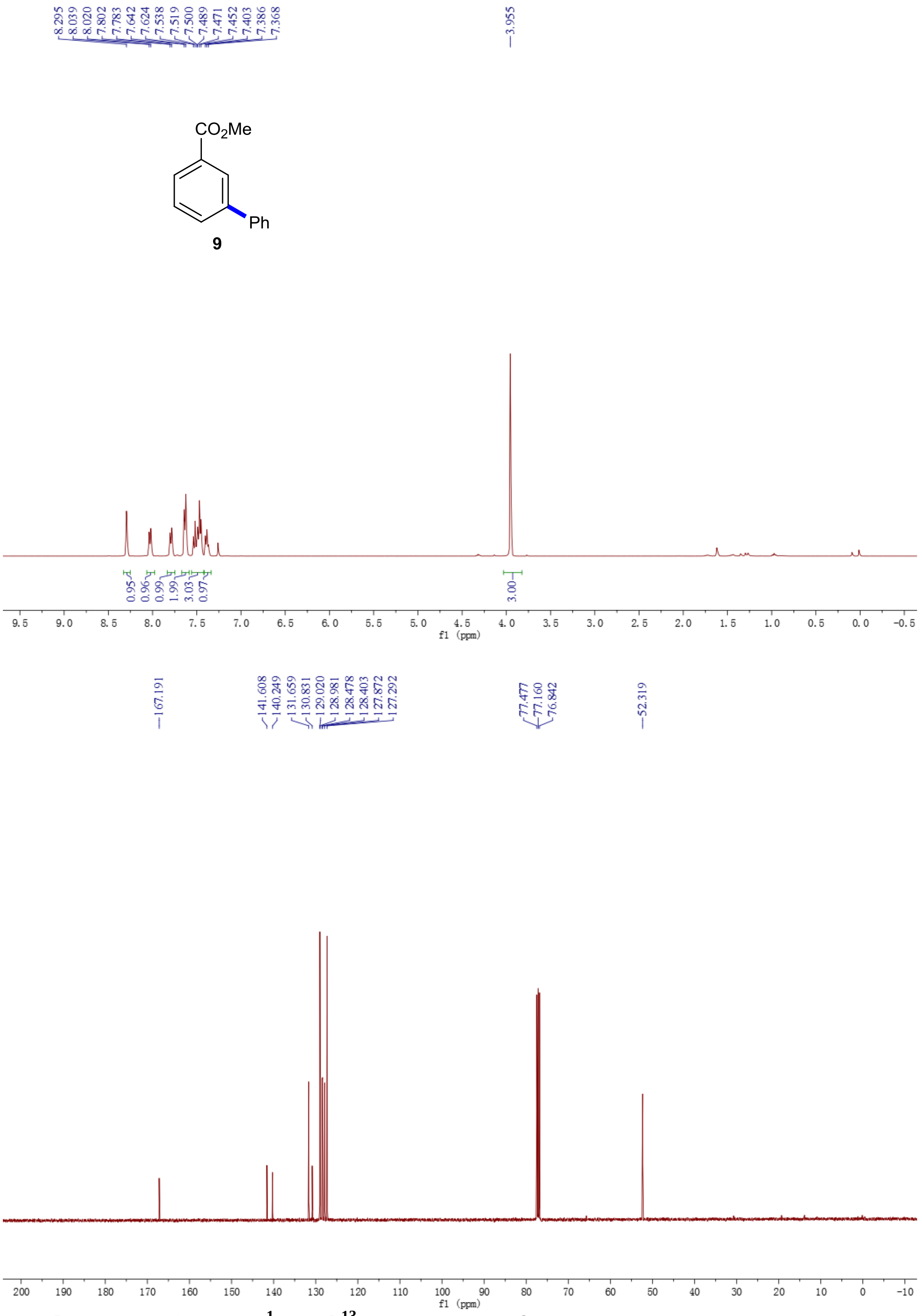

Supplementary Figure 86. ^{1}H and ^{13}C NMR spectra for 9

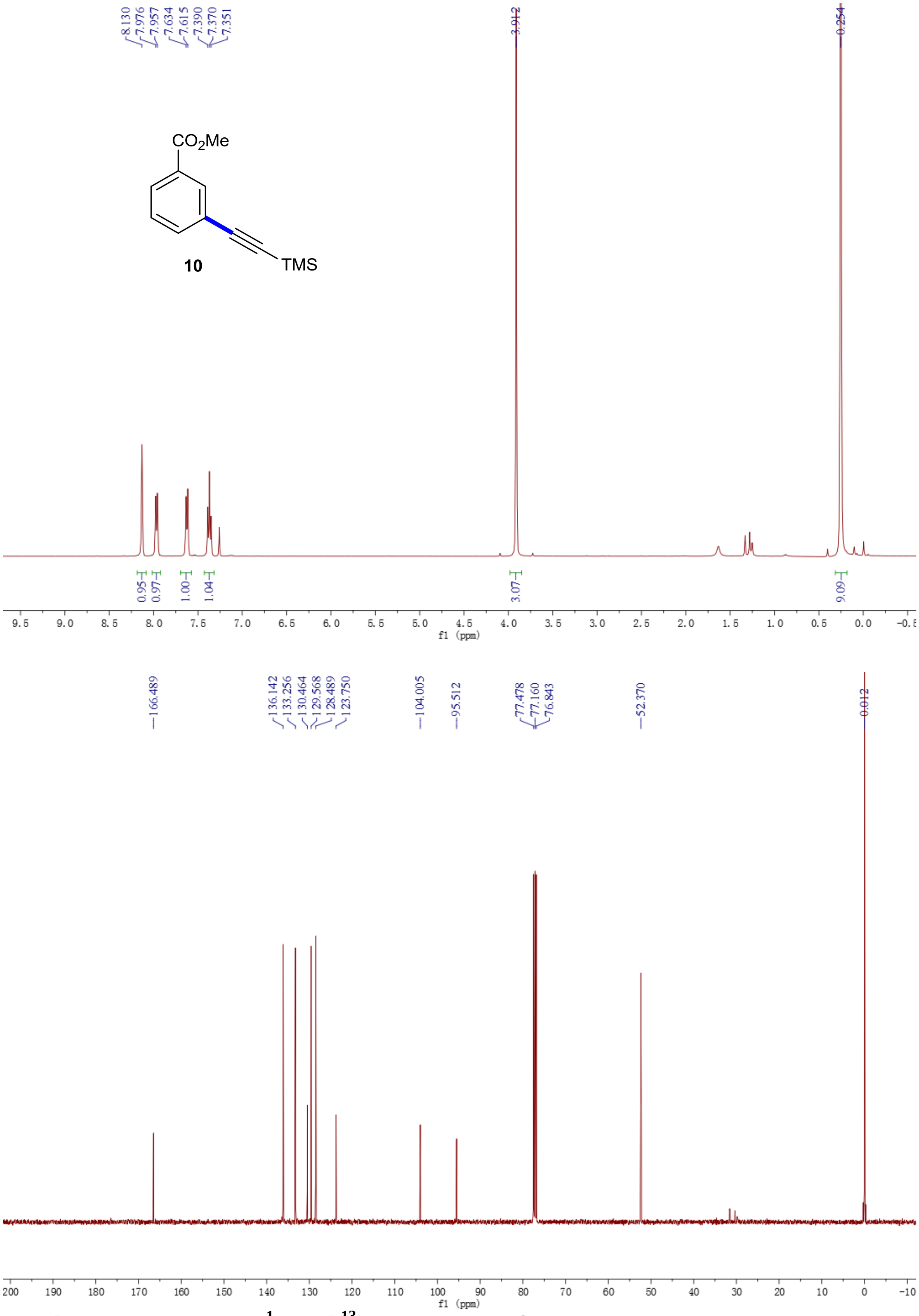

Supplementary Figure 87. ^{1}H and ^{13}C NMR spectra for 10

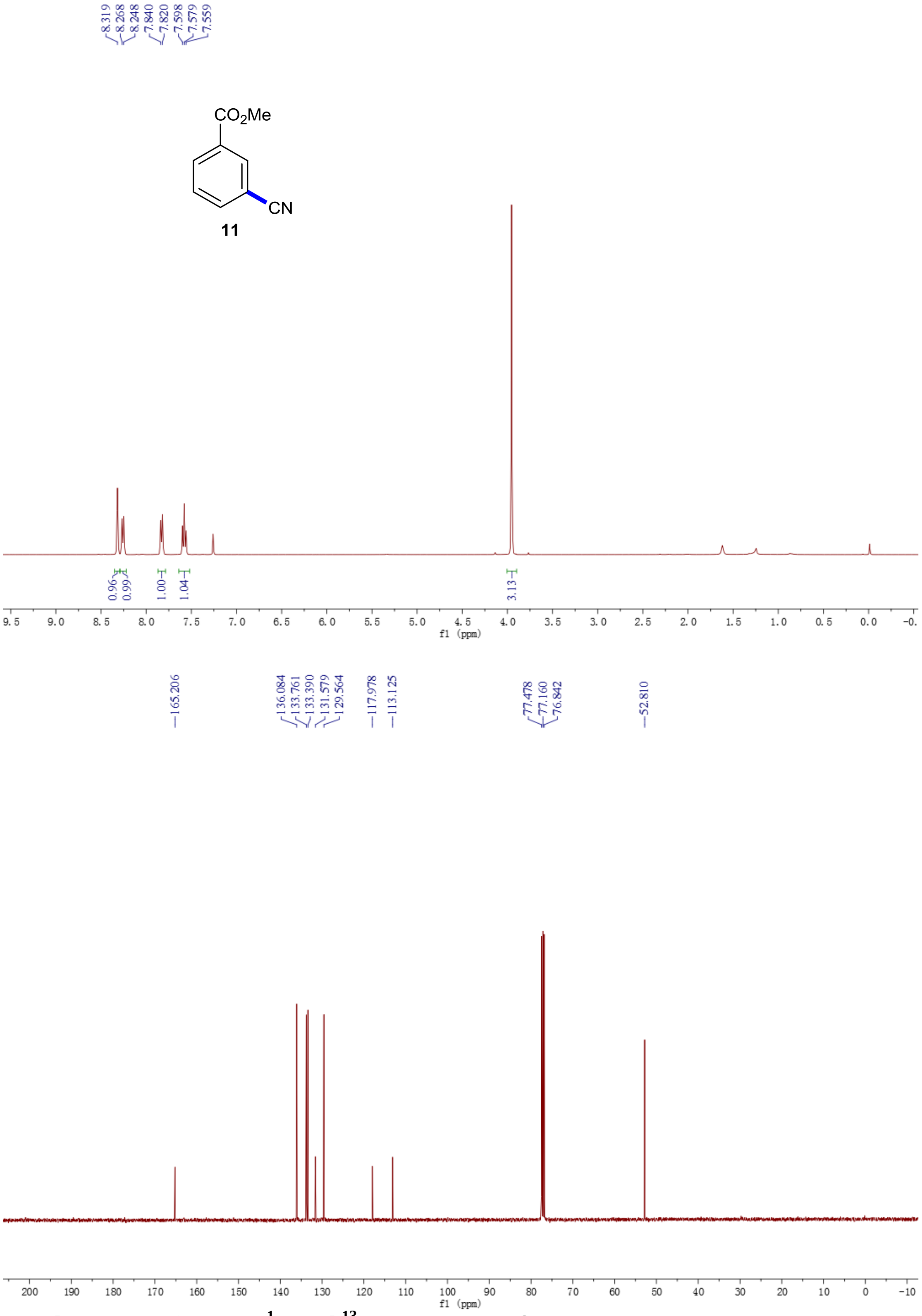

Supplementary Figure 88. ^{1}H and ^{13}C NMR spectra for 11

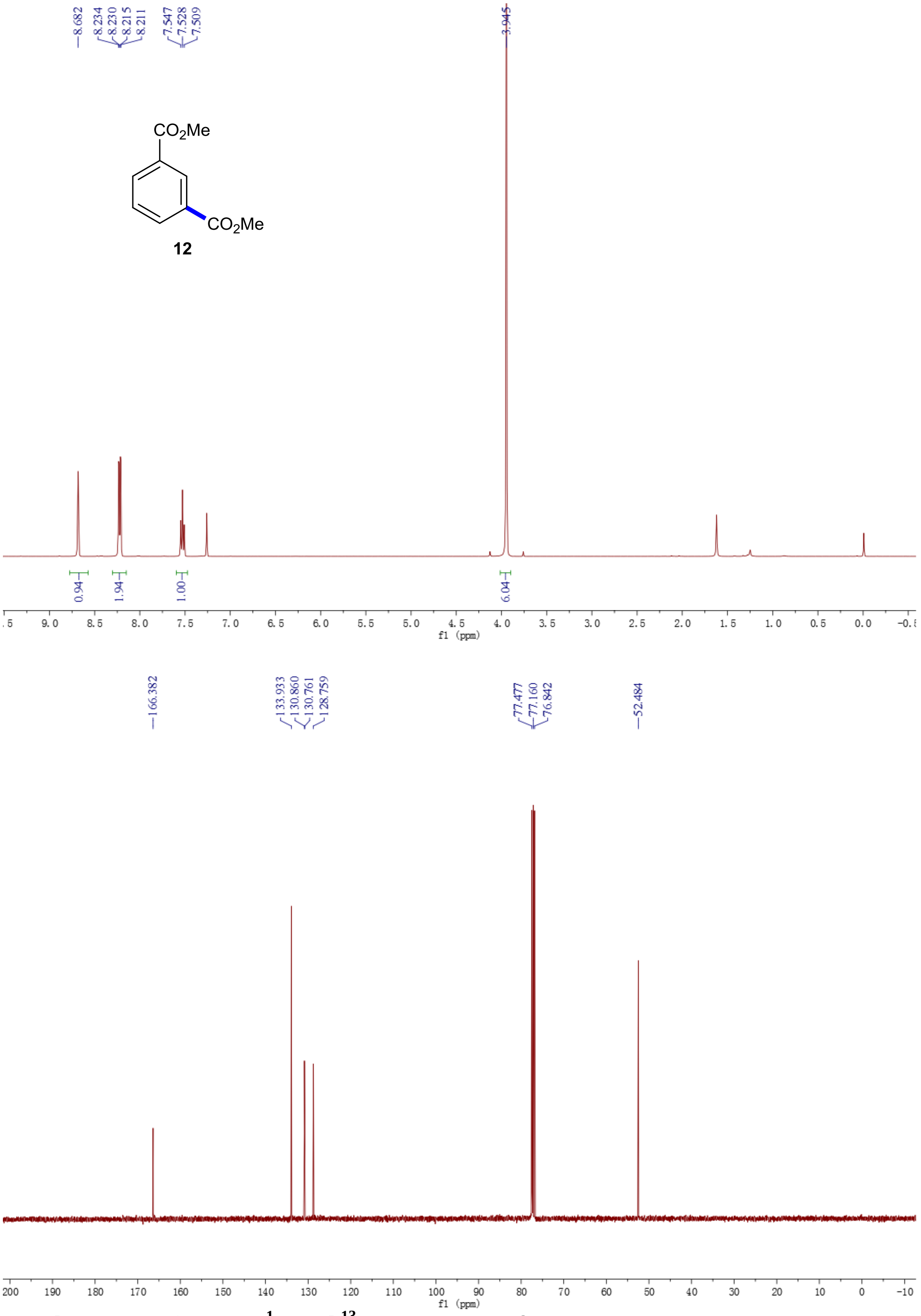

Supplementary Figure 89. ^{1}H and ^{13}C NMR spectra for 12

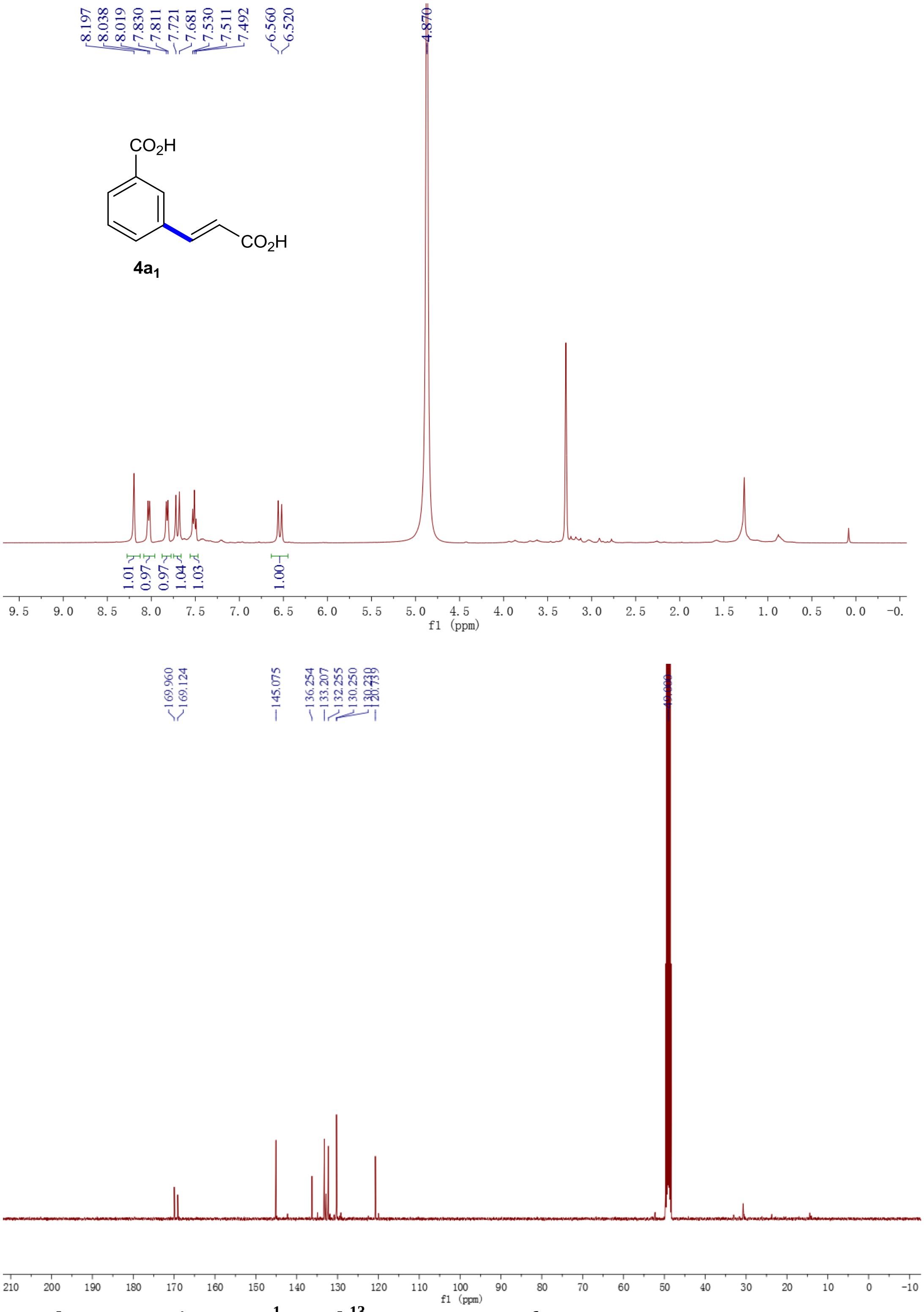

Supplementary Figure 90. ^{1}**H and** ^{13}**C NMR spectra for 4a$_1$**

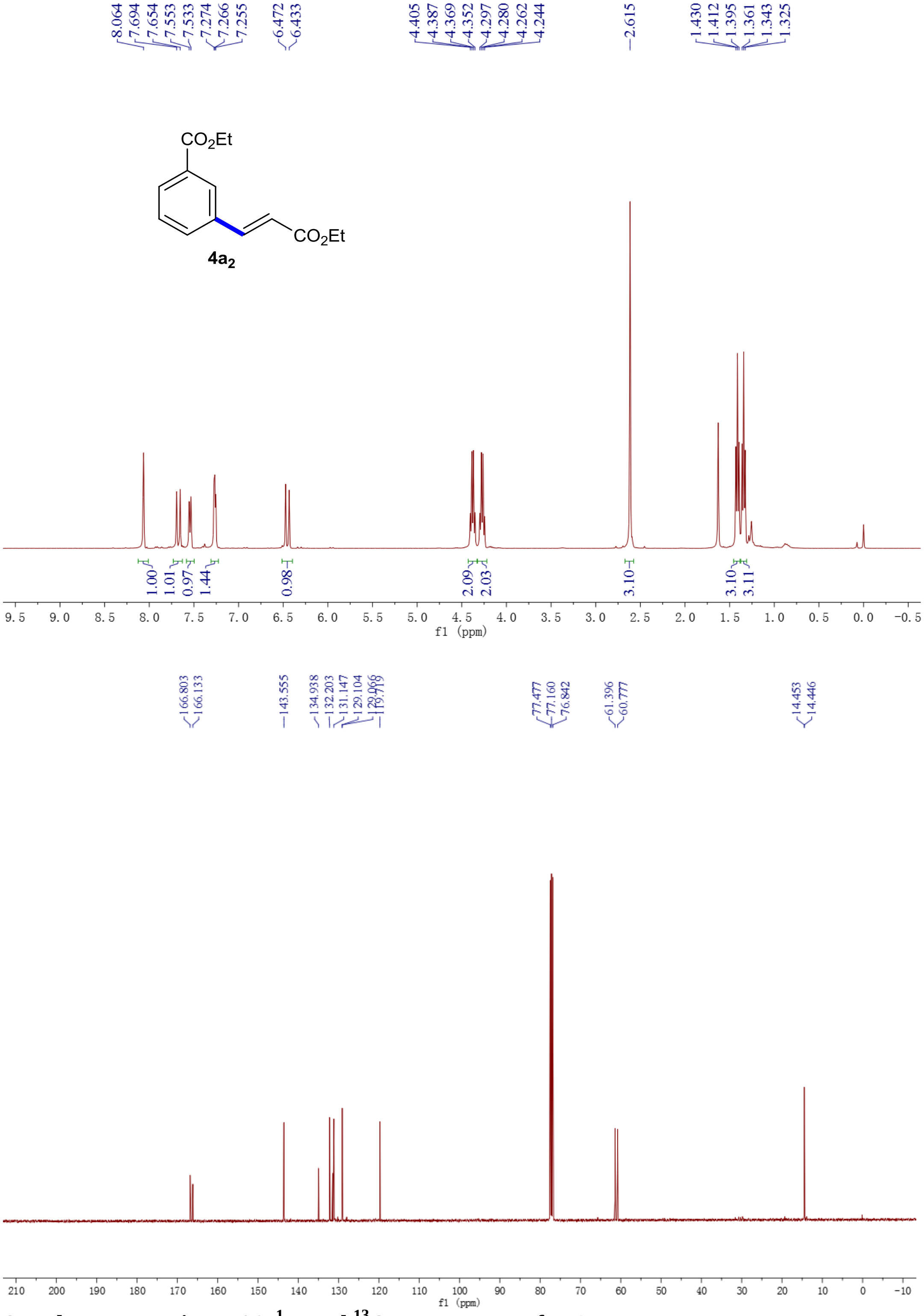

Supplementary Figure 91. ^{1}H and ^{13}C NMR spectra for 4a$_2$

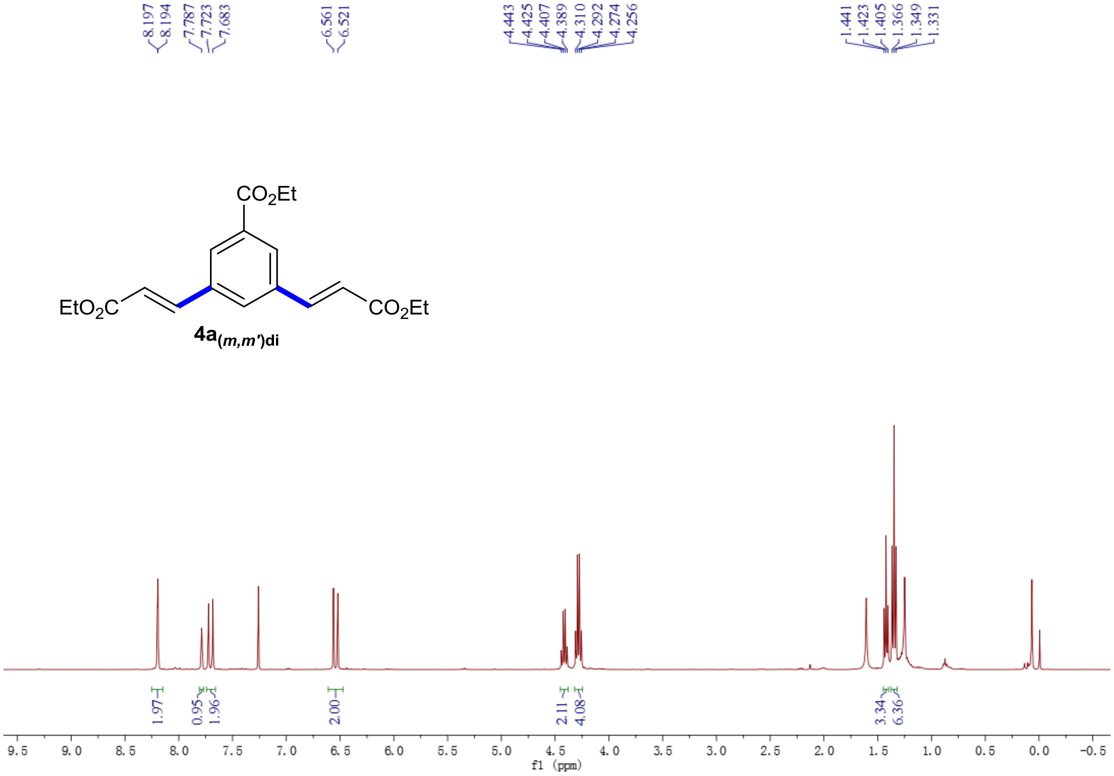

Supplementary Figure 92. ^{1}H NMR spectra for 4a$_{(m,m')di}$

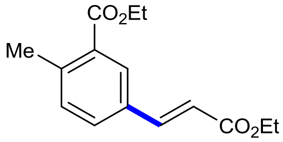

4b

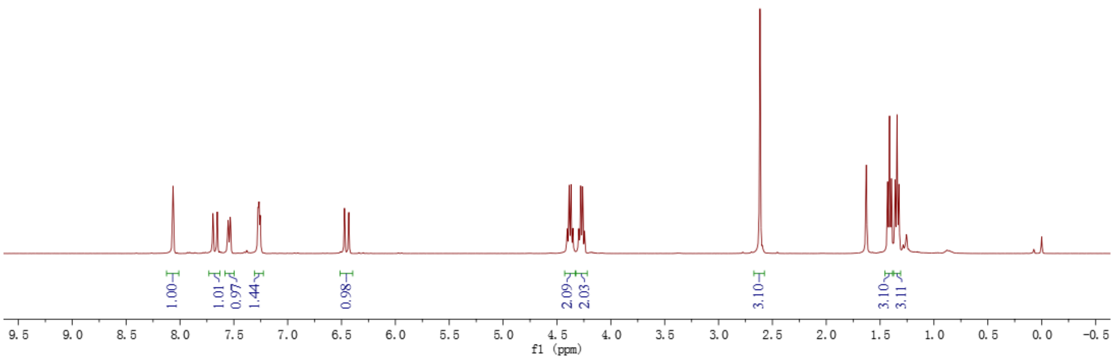

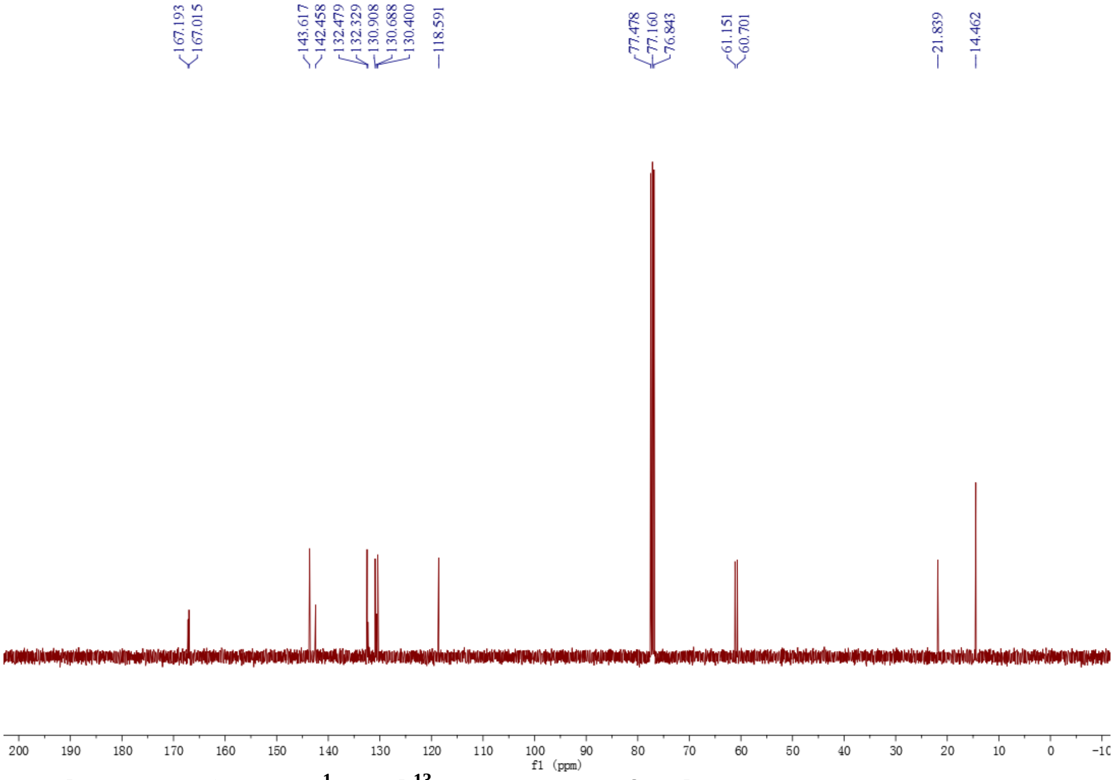

Supplementary Figure 93. ^{1}H and ^{13}C NMR spectra for 4b

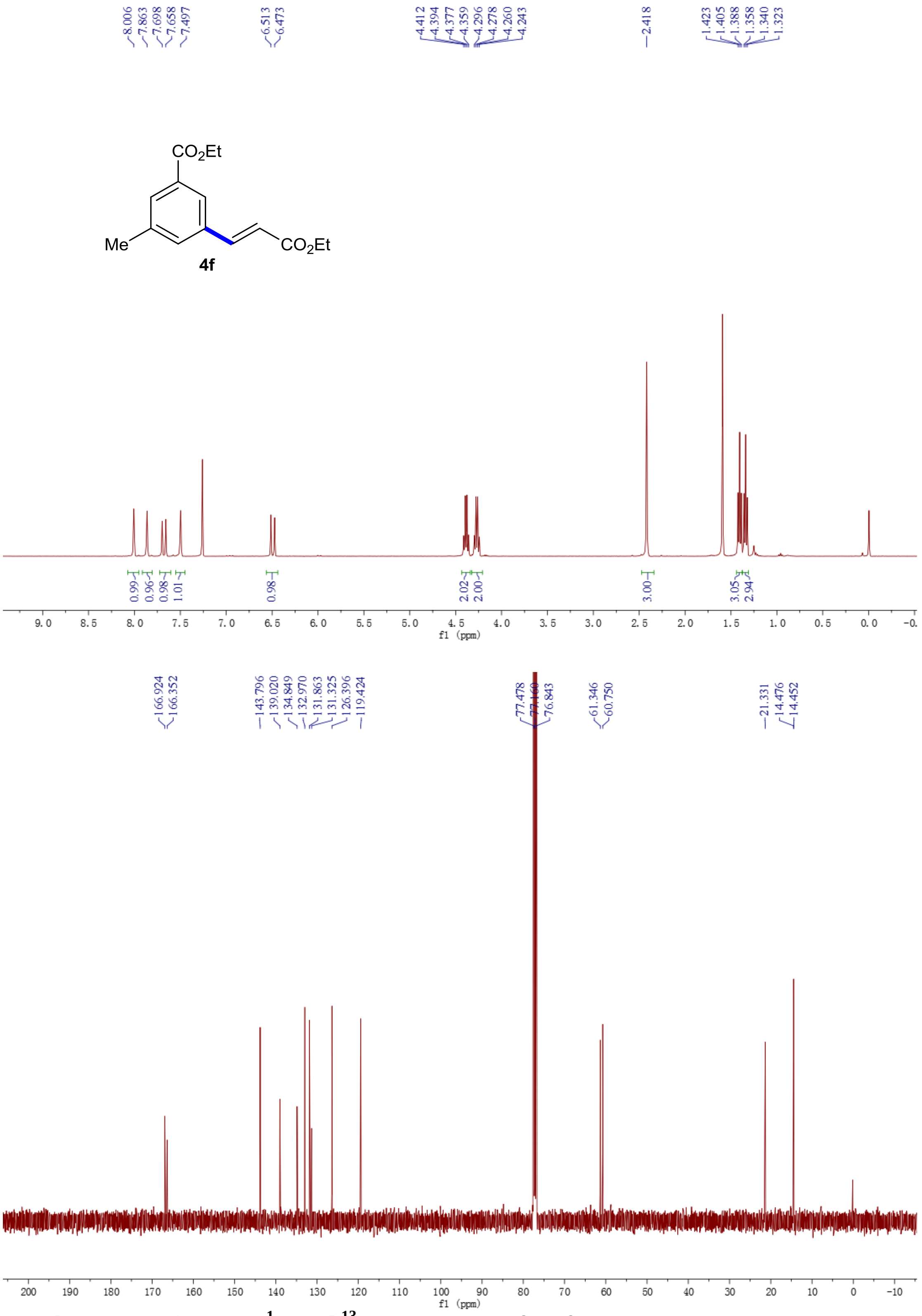

Supplementary Figure 94. ^{1}H and ^{13}C NMR spectra for 4f

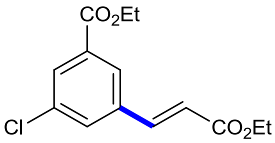

4h

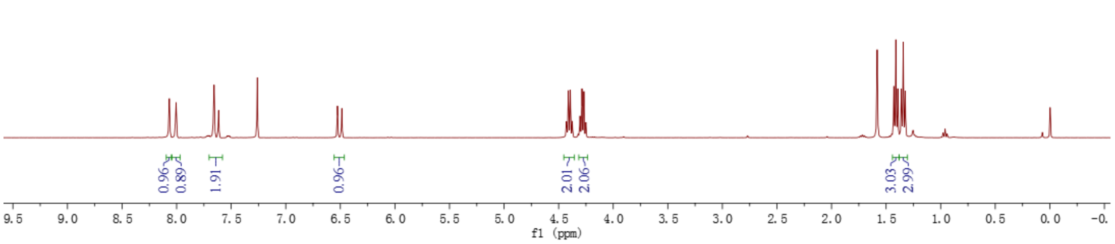

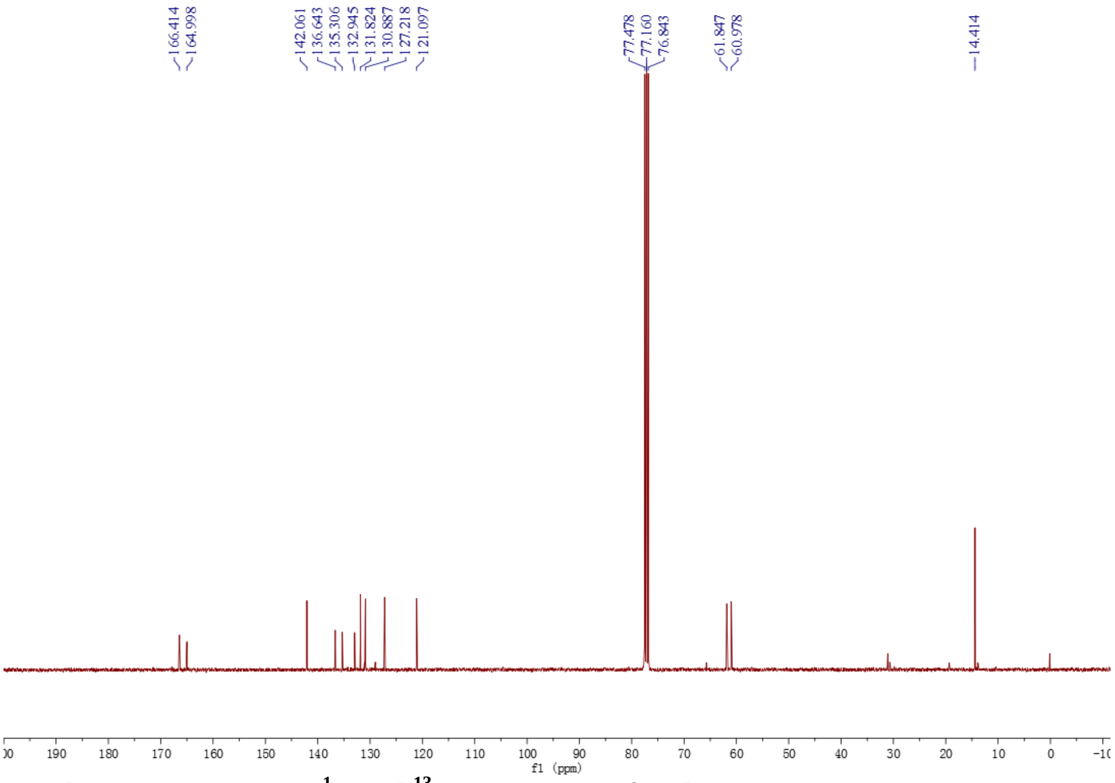

Supplementary Figure 95. ^{1}H and ^{13}C NMR spectra for 4h

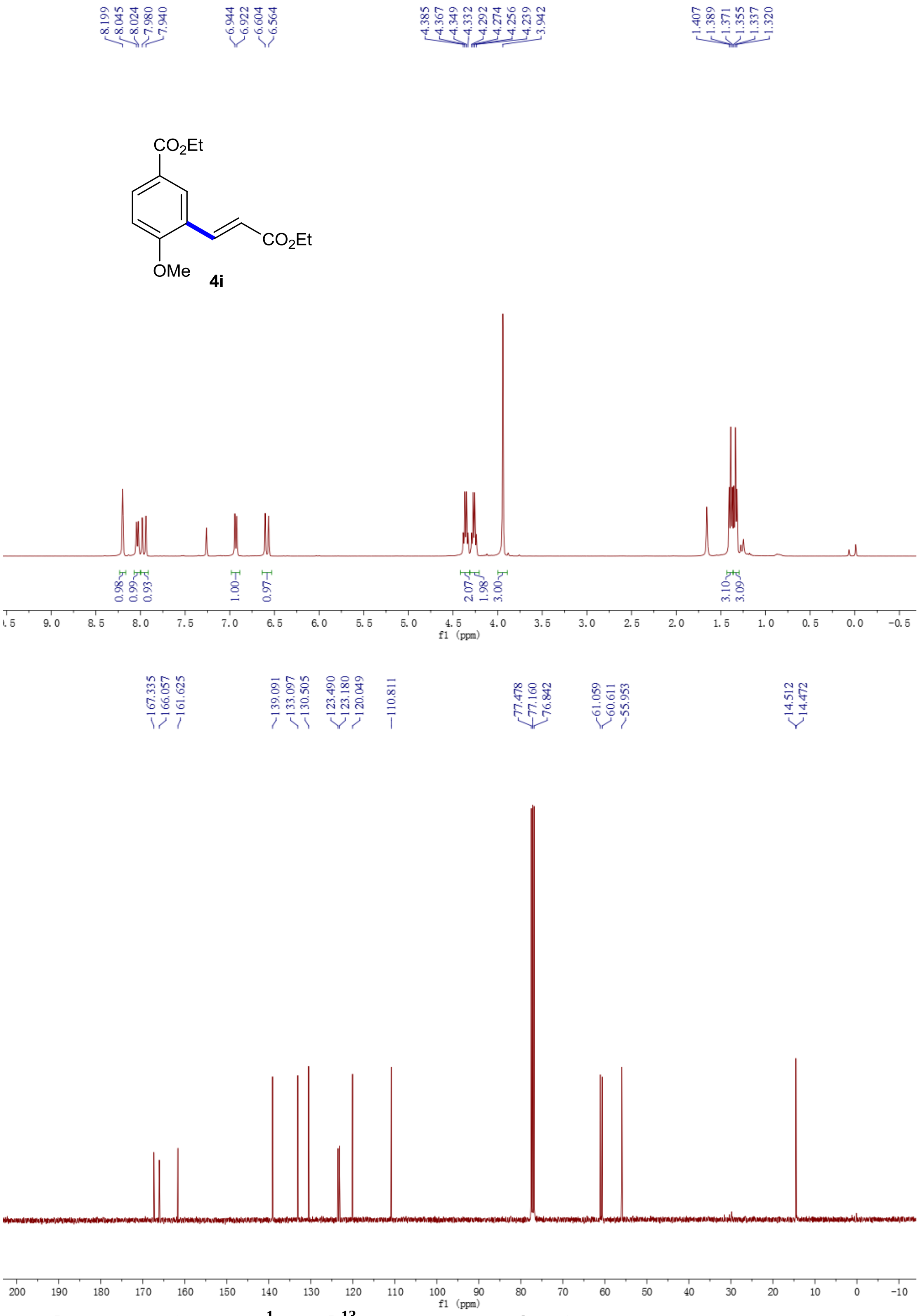

Supplementary Figure 96. ^{1}H and ^{13}C NMR spectra for 4i

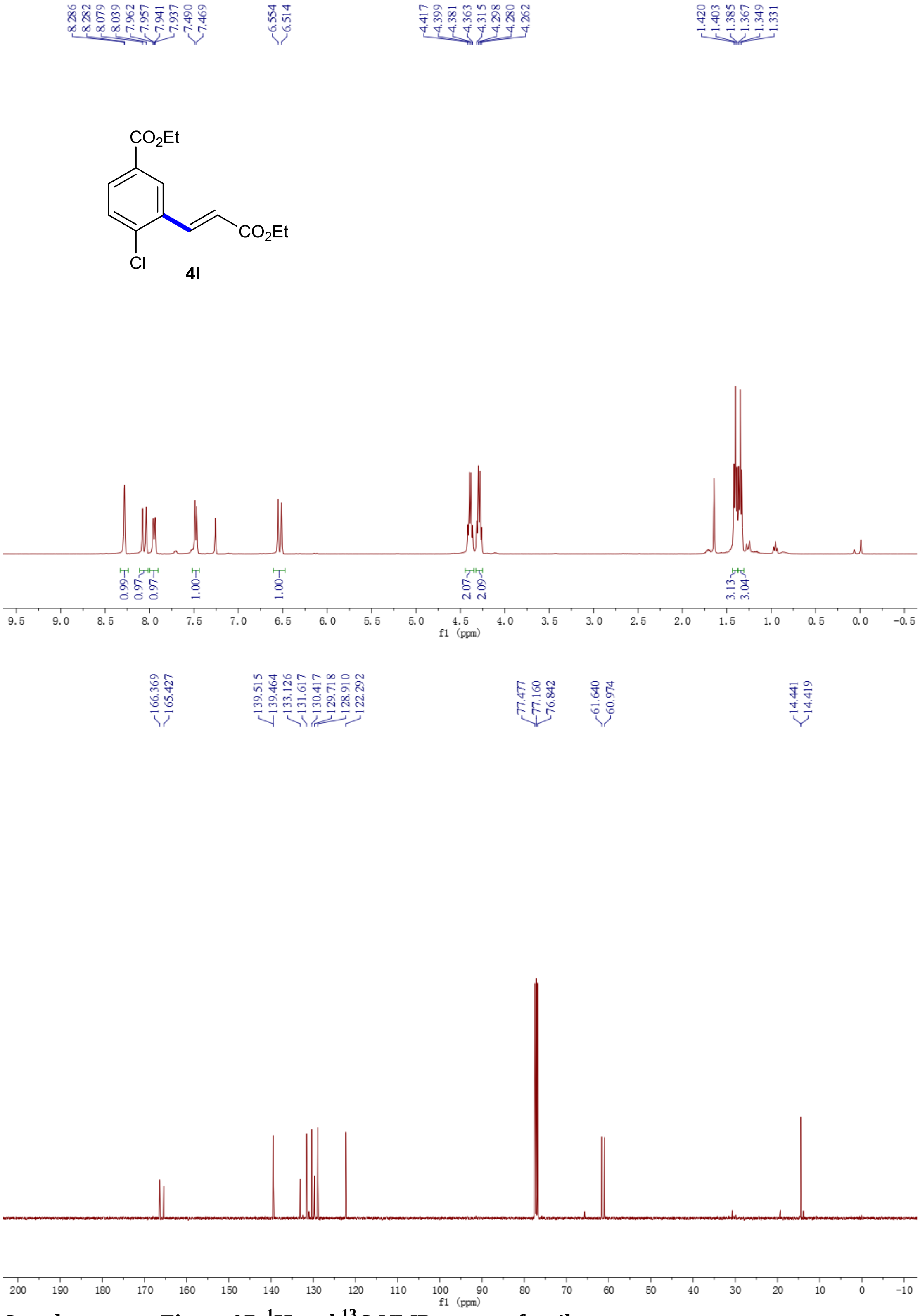

Supplementary Figure 97. ^{1}H and ^{13}C NMR spectra for 4l

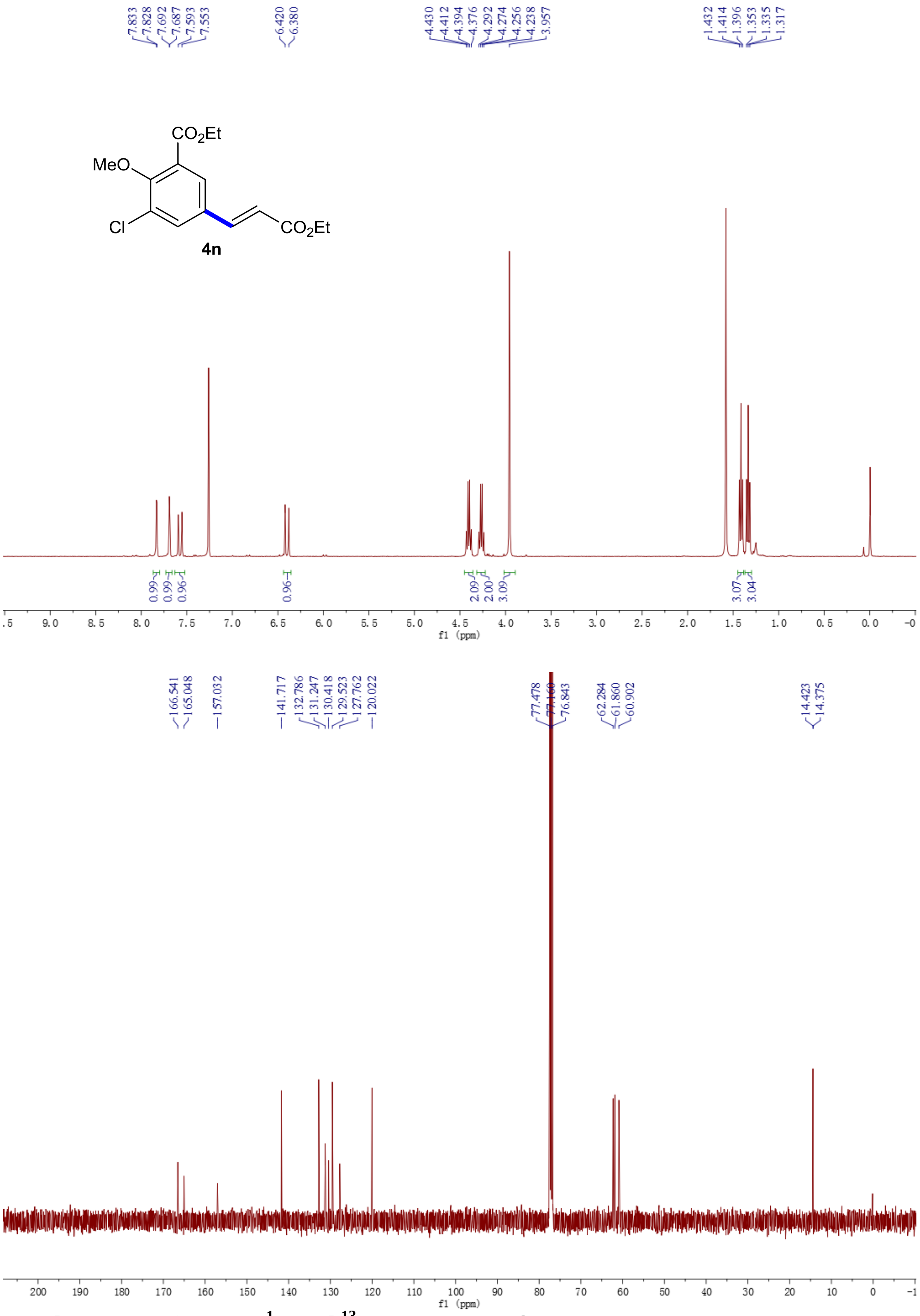

Supplementary Figure 98. ^{1}H and ^{13}C NMR spectra for 4n

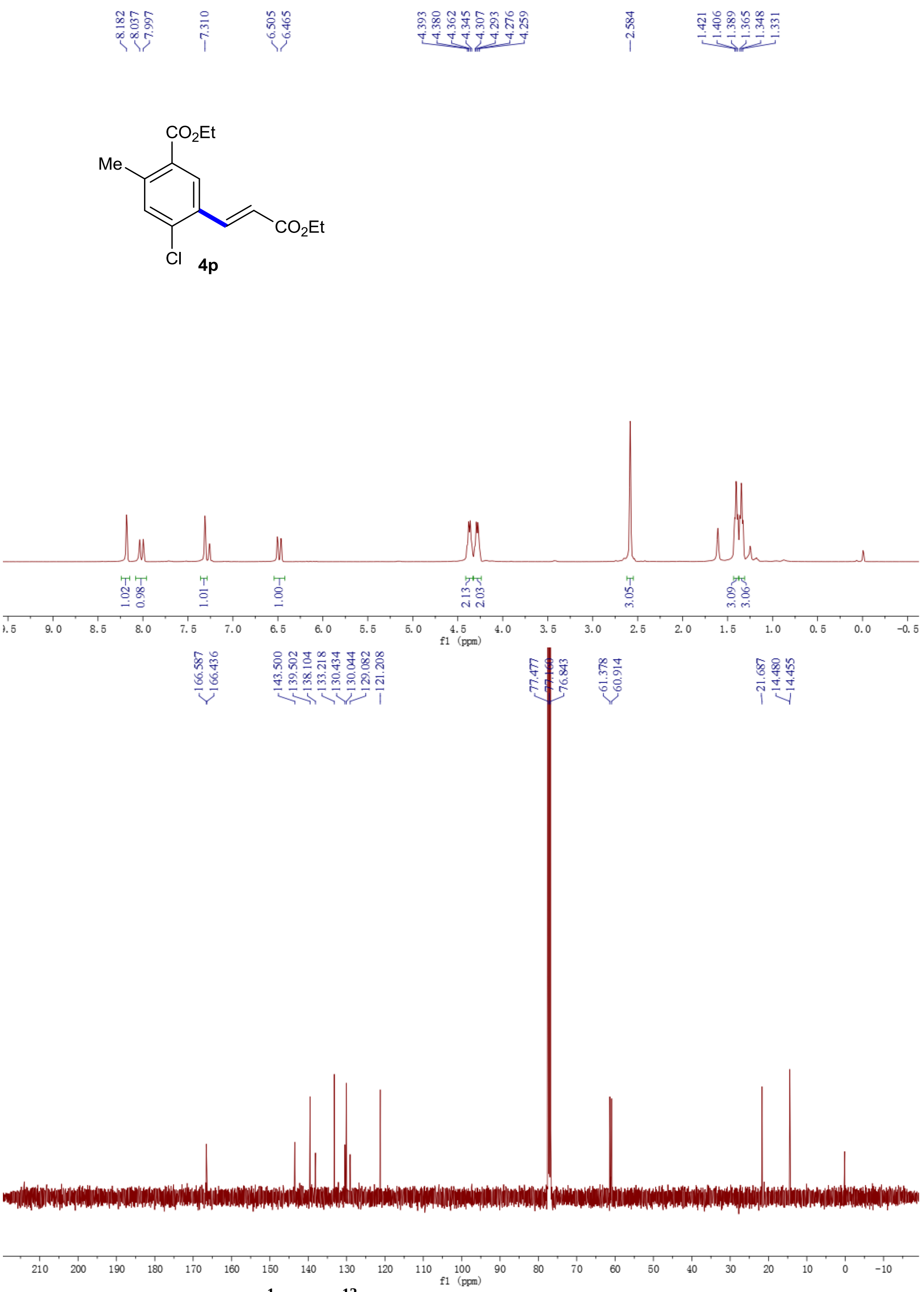

Supplementary Figure 99. ^{1}H and ^{13}C NMR spectra for 4p

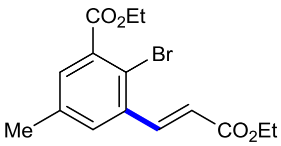

4r

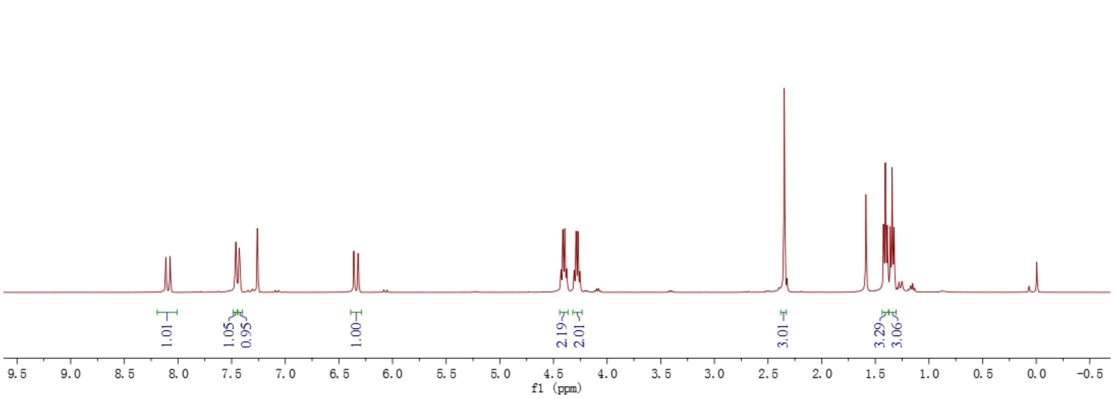

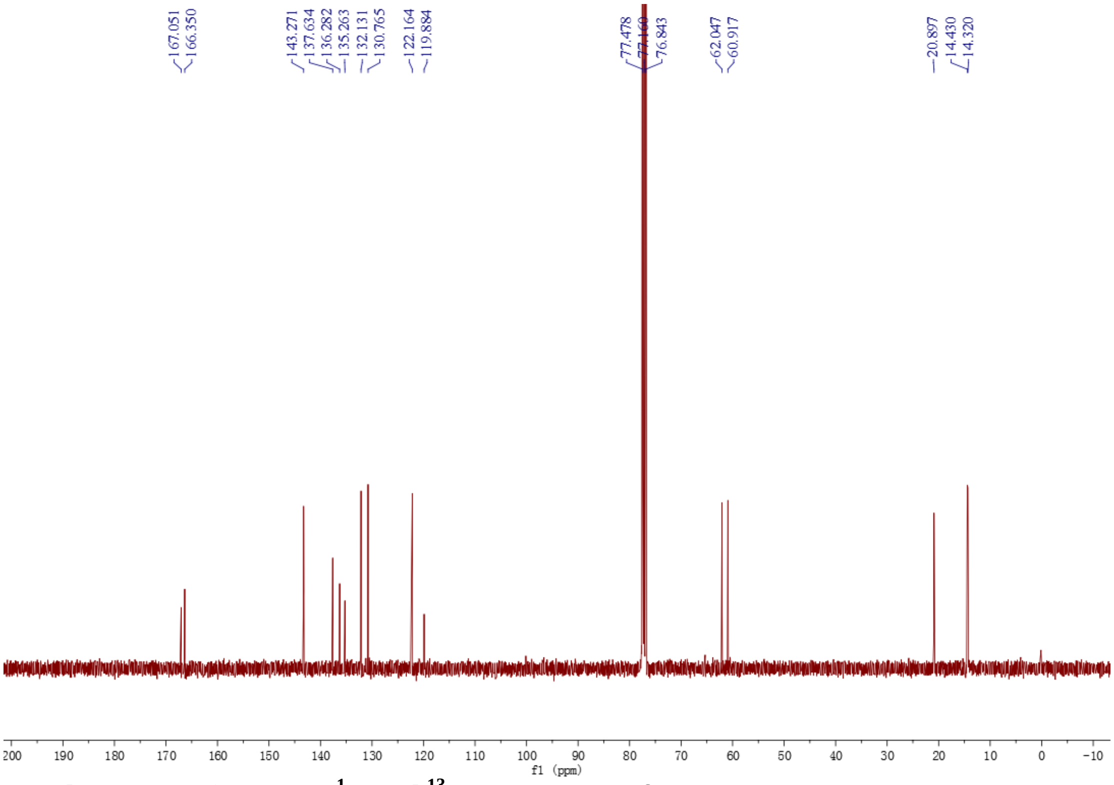

Supplementary Figure 100. ^{1}H and ^{13}C NMR spectra for 4r

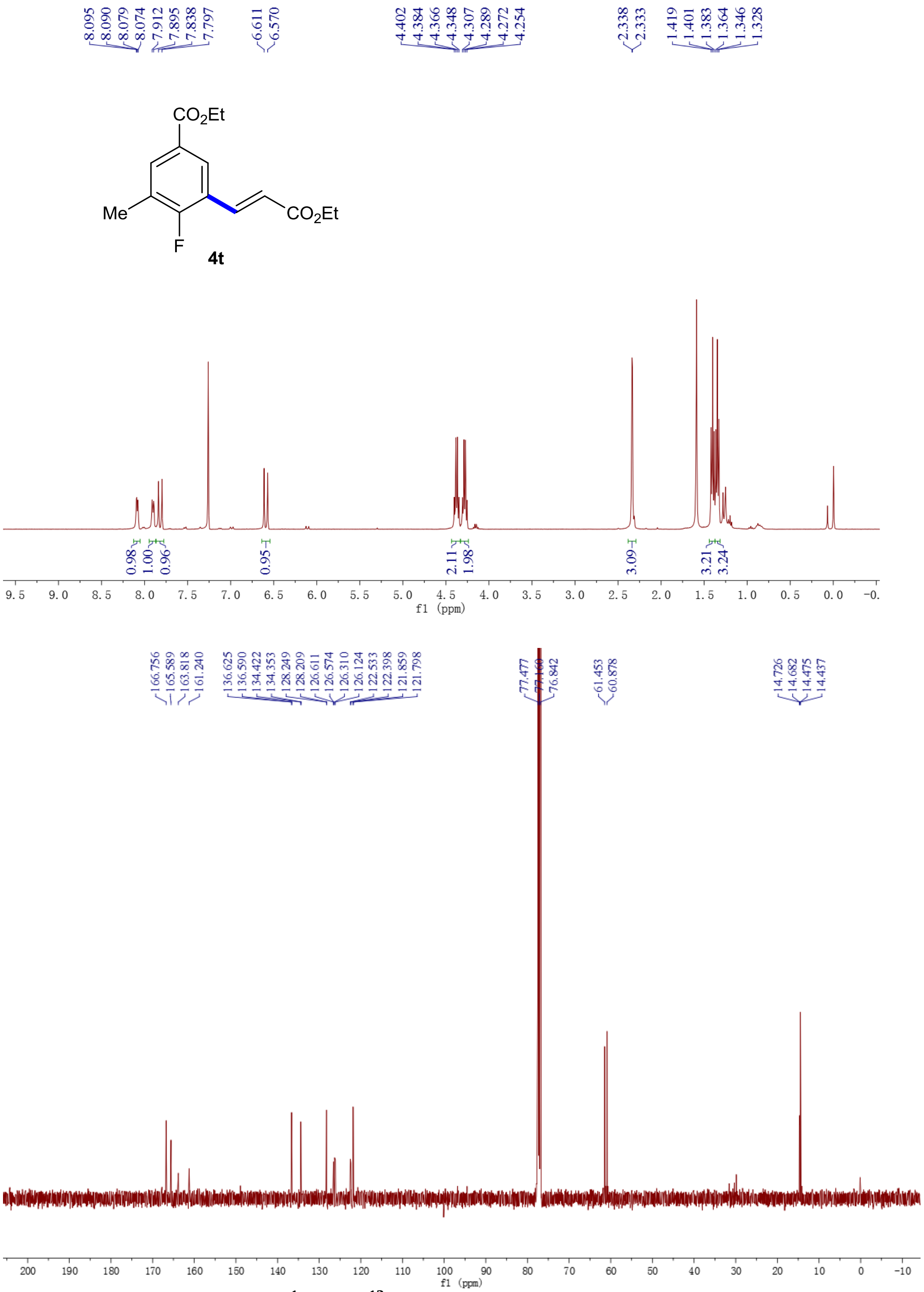

Supplementary Figure 101. ^{1}H and ^{13}C NMR spectra for 4t

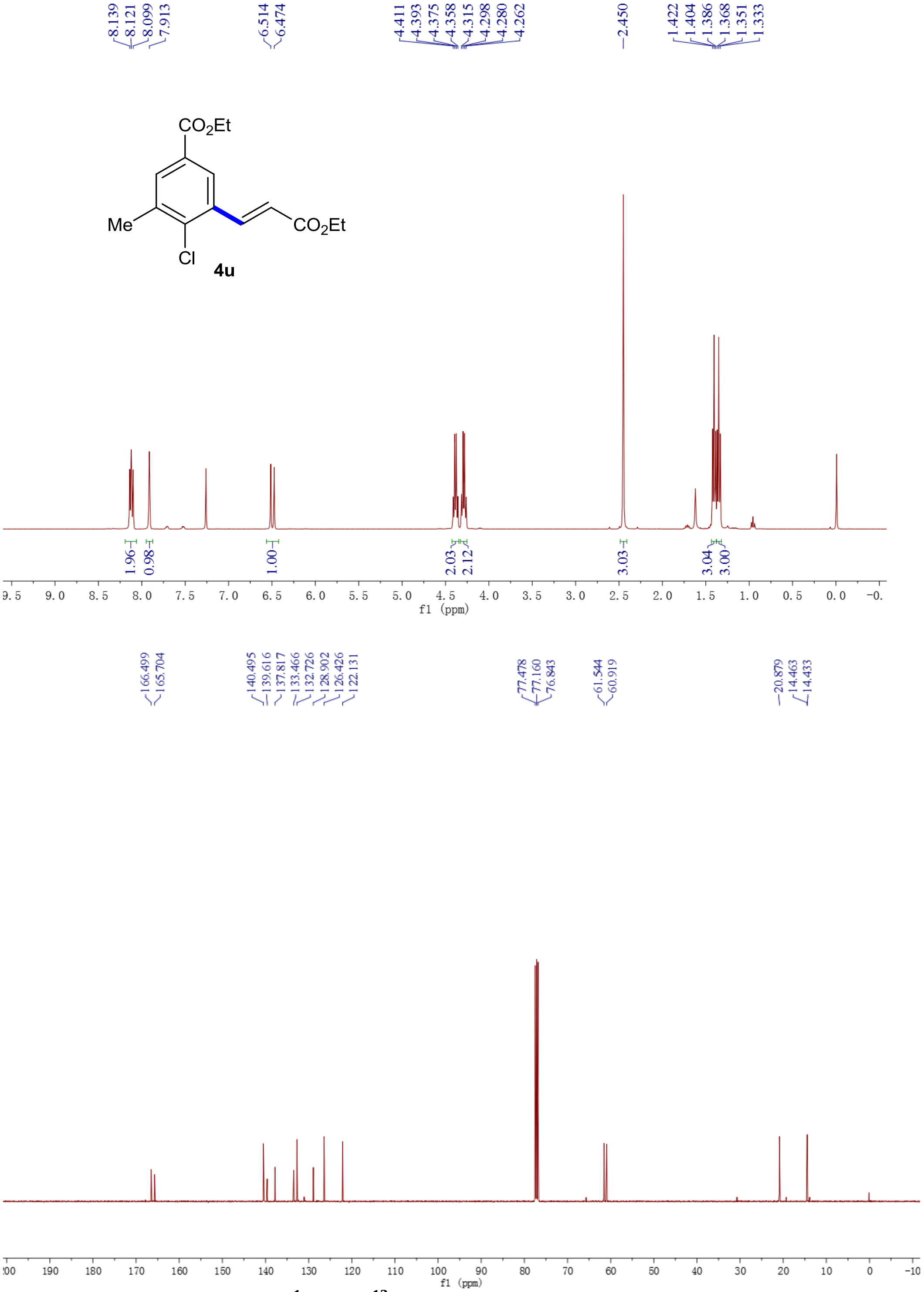

Supplementary Figure 102. ^{1}H and ^{13}C NMR spectra for 4u

Supplementary Tables

Supplementary Table 1. Substrate optimization and condition screenings[a]

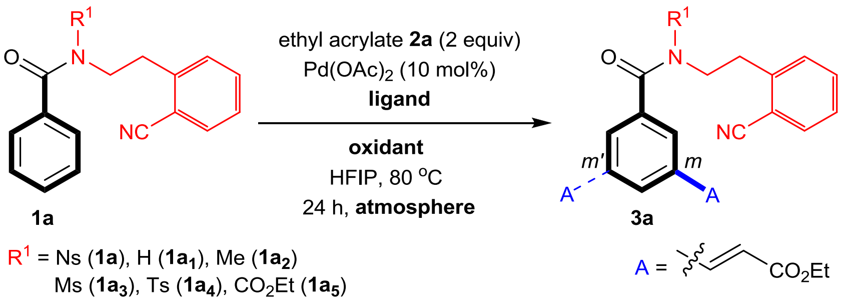

entry	ligand (equiv)	oxidant (equiv)	atmosphere (1 atm)	substrate	yield (%) [$\mathbf{3a}_{mono}$/$\mathbf{3a}_{(m,m')di}$]
1[b,c]	Ac-Gly-OH (0.2)	AgOAc (3)	air	**1a**	85 [1.3/1][d]
2[c]	Ac-Gly-OH (0.2)	AgOAc (3)	air	**1a**	90 [1.5/1][d]
3[b]	Ac-Gly-OH (0.2)	AgOAc (3)	air	$\mathbf{1a_1}$	(23)[e]
4[b,c]	Ac-Gly-OH (0.2)	AgOAc (3)	air	$\mathbf{1a_2}$	(91)[f]
5[b,c]	Ac-Gly-OH (0.2)	AgOAc (3)	air	$\mathbf{1a_3}$	(67)[e]
6[b,c]	Ac-Gly-OH (0.2)	AgOAc (3)	air	$\mathbf{1a_4}$	(84)[e]
7[b,c]	Ac-Gly-OH (0.2)	AgOAc (3)	air	$\mathbf{1a_5}$	(63)[e]
8	Ac-Gly-OH (0.2)	$Cu(OAc)_2$ (2)	O_2	**1a**	72 [5/1]
9	Ac-Gly-OH (0.2)	$Cu(OAc)_2$ (2)	N_2	**1a**	67 [5/1]
10	Ac-Gly-OH (1.0)	$Cu(OAc)_2$ (0.5)	O_2	**1a**	88 [1/2.4]
11[g]	Ac-Gly-OH (1.0)	$Cu(OAc)_2$ (0.2)	O_2	**1a**	86 [1/2.6]
12[c,g]	Ac-Ala-OH (0.6)	$Cu(OAc)_2$ (0.2)	O_2	**1a**	72 [1.4/1][e]
13[c,g]	Ac-Ile-OH (0.6)	$Cu(OAc)_2$ (0.2)	O_2	**1a**	66 [2.7/1][e]
14[g,h]	Ac-Gly-OH (0.6)	$Cu(OAc)_2$ (0.5)	O_2	**1a**	50 [--/1][i]
15[g,h,j]	Ac-Gly-OH (0.6)	$Cu(OAc)_2$ (0.5)	O_2	**1a**	80 [1.7/1][e]
16[g,k]	Ac-Gly-OH (0.3)	$Cu(OAc)_2$ (0.2)	O_2	**1a**	74 [2.7/1]
17	Ac-Gly-OH (0.6)	$Cu(OAc)_2$ (0.5)	O_2	**1a**	--[l]
18	Ac-Gly-OH (0.6)	--	O_2	**1a**	12 [1/--]

[a] Reaction conditions: **1a** (0.1 mmol), **2a** (0.2 mmol), $Pd(OAc)_2$ (10 mol%), Ac-Gly-OH (20-100 mol%), oxidant (0.2-3 equiv), HFIP (1 mL), 24 h, 80 °C, under air or O_2. Yield was determined by ^{1}H NMR analysis using CH_2Br_2 as internal standard. Unless otherwise noted, only traces of isomers were formed. [b] 90°C. [c] **1a** (0.2 mmol). [d] Isolated yield. [e] Combined yields, including minor or traces of regioisomers; for $\mathbf{1a_3}$ only traces of isomers were observed. [f] Products are mixture of mono- and di-rotamers, which may include isomers. See Supplementary Methods for hydrolysis. [g] 48 h. [h] 60 °C, **2a** (0.15 mmol). [i] A trace of mono-olefinated product. [j] HFIP (0.5 mL). [k] $Pd(OAc)_2$ (5 mol%). [l] No reaction. Ms = methanesulfonyl, Ts = *p*-toluenesulfonyl, Ns = 4-nitrobenzenesulfonyl.

Procedure: To a 38 mL sealed tube or 50 mL Schlenk sealed tube (with a Teflon cap, Schlenk sealed tube was used when oxygen was used) equipped with a magnetic stir bar was charged with amide **1a** (0.10 mmol, 1.0 equiv), $Pd(OAc)_2$ (2.3 mg, 0.010 mmol, 10 mol%), ligand (20-100 mol%), oxidant (0.2-3 equiv) and solid additive (if additive was used) sequentially. HFIP (1.0 mL) was added to the mixture along the inside wall of the tube, followed by ethyl acrylate.

The reaction tube was capped, then evacuated briefly under vacuum and charged with O_2 (1 atm, balloon, × 3), if oxygen was used. The tube was then submerged into a preheated 80 or 90 °C oil bath. The reaction was stirred for 24 to 48 h and cooled to room temperature. The crude reaction mixture was diluted with EtOAc (5 mL) and filtered through a short pad of Celite. The sealed tube and Celite pad were washed with an additional 20 mL of EtOAc. The filtrate was concentrated *in vacuo*, and crude ^{1}H NMR spectrum was taken using CH_2Br_2 as internal standard. The residue could be purified by flash silica gel chromatography or preparative thin layer chromatography using petroleum ether/EtOAc as the eluent. The site selectivity was assigned by ^{1}H NMR. (Note: all the chemicals are added as their pure form, for example, not in a solution.)

Supplementary Methods

General Information

Unless otherwise noted, commercial available reagents were purchased from commercial suppliers (such as Energy Chemical, Strem, J&K Chemical Co., Sinopharm, and HWRK Chem), and used as received. Solvents were generally dried over 3 Å molecular seives. Hexafluoroisopropanol (HFIP) was distilled before use. Unless otherwise noted, all reactions were run under air and the indicated reaction temperature was that of the oil bath. The reaction vessels used for C–H functionalisation were 50 mL Schlenk tube or 38 mL sealed tube (Synthware). Purification of products was performed by flash chromatography (FC) using silica gel or preparative thin layer chromatography. ^{1}H and ^{13}C NMR spectra were recorded on a Bruker AVANCE III spectrometer (400 MHz and 101 MHz, respectively). Chemical shifts are reported parts per million (ppm) referenced to $CDCl_3$ (δ 7.26 ppm) for ^{1}H NMR, $CDCl_3$ (δ 77.16 ppm) for ^{13}C NMR or tetramethylsilane (TMS, δ 0.00 ppm for ^{1}H NMR). The following abbreviations (or combinations thereof) were used to explain multiplicities: s = singlet, d = doublet, t = triplet, q = quartet, hept = heptaplet, m = multiplet, and br = broad. High-resolution mass spectra (HRMS) were obtained on an AB Sciex TripleTOF 5600 LC/MS equipped with an ESI source at the Shanghai Institute of Organic Chemistry (CAS).

Experimental Section

Preparation and characterization of substrates.

Method A for preparation of substrates: preparation of **1a-1a$_5$**.
(NOTE: 1a was prepared in large scale using method B**)**

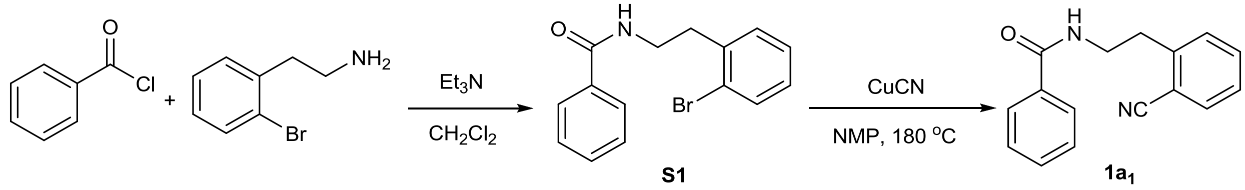

Step 1:

To a solution of 2-(2-bromophenyl)ethanamine (1.0 g, 5 mmol) and Et_3N (1.5 mL, 10 mmol) in CH_2Cl_2 (15 mL) under N_2 at 0 °C was added benzoyl chloride (0.7 g, 5 mmol) dropwise. The mixture was stirred for another 2 h at room temperature and then quenched by addition of H_2O (30 mL). The organic phase was separated and the aqueous phase was extracted with CH_2Cl_2 (20 mL × 2). The combined organic phase was dried over anhydrous Na_2SO_4 and concentrated under reduced pressure. The residue was purified by silica gel chromatography with petroleum ether/EtOAc (2:1) to afford compound **S1** (1.4 g) in 92% yield.

Step 2:
A mixture of **S1** (1.5 g, 5 mmol) and CuCN (672 mg, 7.5 mmol) in NMP (25 mL) was heated at 180 °C for 24 h. After cooled to room temperature, H_2O (100 mL) was added and the aqueous phase was extracted with EtOAc (20 mL × 3). The combined organic phase was dried over anhydrous Na_2SO_4 and concentrated under reduced pressure. The residue was purified by silica gel chromatography with petroleum ether/EtOAc (2:1) to afford compound **1a₁** (1.0 g) in 80% yield.

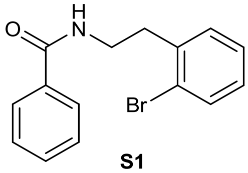

S1

***N*-(2-bromophenethyl)benzamide**: ^{1}H NMR (400 MHz, $CDCl_3$) δ 7.72 (d, *J* = 7.4 Hz, 2H), 7.55 (d, *J* = 8.0 Hz, 1H), 7.47 (t, *J* = 7.2 Hz, 1H), 7.39 (t, *J* = 7.3 Hz, 2H), 7.29-7.20 (m, 2H), 7.14-7.04 (m, 1H), 6.40 (s, 1H), 3.72 (q, *J* = 6.3 Hz, 2H), 3.09 (t, *J* = 6.7 Hz, 2H). ^{13}C NMR (101 MHz, $CDCl_3$) δ 167.7, 138.5, 134.6, 133.1, 131.5, 131.2, 128.6, 128.4, 127.8, 127.0, 124.7, 40.0, 35.8.

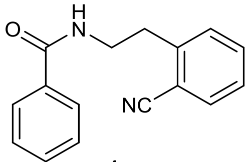

$1a_1$

***N*-(2-cyanophenethyl)benzamide**: ^{1}H NMR (400 MHz, $CDCl_3$) δ 7.73 (d, *J* = 7.0 Hz, 2H), 7.62 (d, *J* = 7.4 Hz, 1H), 7.58-7.45 (m, 2H), 7.45-7.37 (m, 3H), 7.33 (t, *J* = 7.1 Hz, 1H), 6.45 (s, 1H), 3.77 (q, *J* = 6.1 Hz, 2H), 3.18 (t, *J* = 6.2 Hz, 2H). ^{13}C NMR (101 MHz, $CDCl_3$) δ 167.8, 143.0, 134.4, 133.2, 133.0, 131.7, 130.4, 128.7, 127.4, 127.0, 118.3, 112.7, 40.6, 34.6.

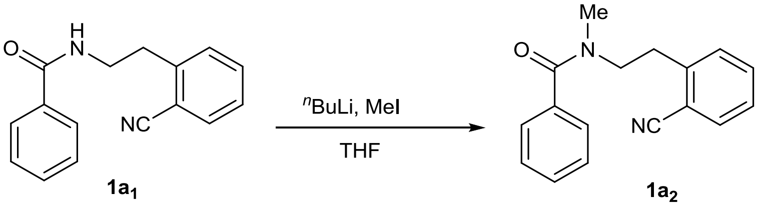

$1a_1$ **$1a_2$**

To a solution of **$1a_1$** (50 mg, 0.2 mmol) in anhydrous THF (2 mL) under N_2 at -78 °C was added ^{n}BuLi (100 μL, 2.4 M in hexane, 0.24 mmol) dropwise. After stirring for 5 min at -78 °C, MeI (57 mg, 25 μL, 0.4 mmol) was added to the mixture. Then the reaction was warmed gradually to room temperature and stirred for another 1 h. Then H_2O (5 mL) was added slowly to quench the reaction. The aqueous phase was extracted with EtOAc (10 mL × 3). The combined organic phase was dried over anhydrous Na_2SO_4 and concentrated under reduced pressure. The residue

was purified by silica gel chromatography with petroleum ether/EtOAc (2:1) to afford compound **1a$_2$** (37 mg) in 70% yield.

1a-1a$_5$ were prepared using similar method as 1a$_2$ (NOTE: 1a was prepared in large scale using method B**).**

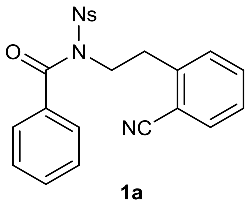

1a ***N*-(2-cyanophenethyl)-*N*-(4-nitrophenylsulfonyl)benzamide**: ^{1}H NMR (400 MHz, $CDCl_3$) δ 8.36 (d, *J* = 8.9 Hz, 2H), 8.19 (d, *J* = 8.8 Hz, 2H), 7.57-7.48 (m, 3H), 7.41-7.32 (m, 3H), 7.26 (t, *J* = 6.4 Hz, 3H), 4.24 (t, *J* = 7.1 Hz, 2H), 3.20 (t, *J* = 7.1 Hz, 2H). ^{13}C NMR (101 MHz, $CDCl_3$) δ 171.1, 150.8, 144.4, 140.8, 133.5, 133.25, 133.24, 132.4, 131.0, 130.3, 128.8, 127.9, 124.3, 117.4, 112.9, 48.5, 35.1. HRMS (m/z, ESI-TOF): Calcd for $C_{22}H_{18}N_3O_5S^+$ [M+H$^+$] 436.0962, found 436.0969.

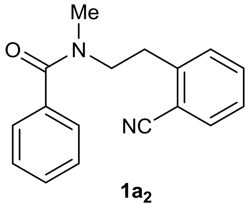

1a$_2$ ***N*-(2-cyanophenethyl)-*N*-methylbenzamide**: ^{1}H NMR (400 MHz, $CDCl_3$) δ 7.70-7.28 (m, 8H), 7.16-7.03 (m, 1H), 3.83 (t, *J* = 6.8 Hz, 1.20H), 3.60 (s, 0.79H), 3.24 (t, *J* = 6.8 Hz, 1.20H), 3.18 (s, 1.24H), 3.03 (s, 0.83H), 2.95 (s, 1.81H). ^{13}C NMR (101 MHz, $CDCl_3$) δ 172.1, 171.5, 142.9, 141.7, 136.2, 136.0, 133.1, 132.8, 130.5, 130.2, 129.6, 129.5, 128.4, 127.4, 127.3, 126.9, 126.4, 118.1, 117.4, 112.6, 51.7, 48.4, 38.1, 33.3, 33.2, 32.1.

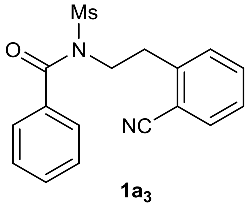

1a$_3$ ***N*-(2-cyanophenethyl)-*N*-(methylsulfonyl)benzamide**: ^{1}H NMR (400 MHz, $CDCl_3$) δ 7.57-7.46 (m, 3H), 7.40 (t, *J* = 7.4 Hz, 2H), 7.36-7.27 (m, 4H), 4.16 (t, *J* = 6.9 Hz, 2H), 3.37 (s, 3H), 3.15 (t, *J* = 6.9 Hz, 2H). ^{13}C NMR (101 MHz, $CDCl_3$) δ 172.2, 141.0, 133.8, 133.2, 133.2, 131.9, 130.9, 128.9, 127.8, 127.4, 117.3, 112.9, 48.2, 42.9, 35.1.

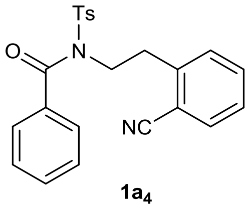

1a$_4$ ***N*-(2-cyanophenethyl)-*N*-tosylbenzamide**: ^{1}H NMR (400 MHz, $CDCl_3$) δ 7.79 (d, *J* = 8.3 Hz, 2H), 7.54-7.48 (m, 2H), 7.45 (t, *J* = 7.4 Hz, 1H), 7.36-7.27 (m, 6H), 7.24-7.17 (m, 2H), 4.19 (t, *J* = 7.0 Hz, 2H), 3.22 (t, *J* = 7.0 Hz, 2H), 2.41 (s, 3H). ^{13}C NMR (101

MHz, $CDCl_3$) δ 171.1, 145.1, 141.4, 135.8, 134.5, 133.1, 133.0, 131.7, 131.0, 129.7, 128.5, 128.3, 127.8, 127.5, 117.4, 112.8, 47.7, 34.7, 21.7.

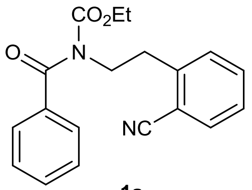

1a$_5$ **ethyl benzoyl(2-cyanophenethyl)carbamate**: ^{1}H NMR (400 MHz, $CDCl_3$) δ 7.54 (d, *J* = 7.5 Hz, 1H), 7.43 (td, *J* = 7.6, 1.2 Hz, 1H), 7.40-7.34 (m, 3H), 7.32 (d, *J* = 7.7 Hz, 1H), 7.30-7.18 (m, 3H), 4.10 (t, *J* = 7.2 Hz, 2H), 3.87 (q, *J* = 7.1 Hz, 2H), 3.20 (t, *J* = 7.2 Hz, 2H), 0.87 (t, *J* = 7.1 Hz, 3H). ^{13}C NMR (101 MHz, $CDCl_3$) δ 172.7, 154.6, 142.3, 136.8, 132.9, 132.8, 131.2, 130.4, 128.0, 127.3, 127.3, 117.7, 113.0, 63.0, 45.7, 33.7, 13.6.

Method B for preparation of substrates: preparation of **1a-1za.**

Preparation and characterization of the auxiliary 5.

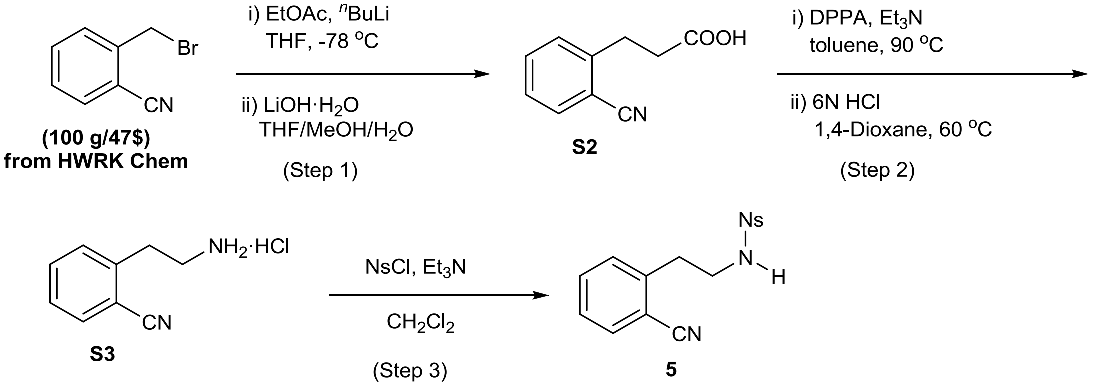

Step 1:

To an oven dried 500 mL round bottom flask, diisopropylamine (15 mL, 107 mmol) was dissolved in anhydrous THF (200 mL) under N_2 and the reaction vessel was cooled to -78 °C. To this cooled solution was dropwise added ^{n}BuLi (2.4 M in hexane, 42.5 mL, 102 mmol) and the mixture was allowed to stir for an additional 1 h below -30 °C. The solution was cooled to -78 °C again and EtOAc (10 mL, 102 mmol, diluted with 50 mL anhydrous THF) was added dropwise. After completion of the addition, the mixture was stirred at -78 °C for 1 h. Then 2-(bromomethyl)benzonitrile (16.6 g, 85 mmol, dissolved in 100 mL anhydrous THF) was added dropwise. The stirring solution was gradually warmed to room temperature in about 4 h and quenched with H_2O (300 mL). The organic phase was separated and the aqueous phase was extracted with EtOAc (100 mL × 3). The combined organic phase was concentrated under reduced pressure and the residue was used without further purification.

The above residue was dissolved in THF (150 mL), MeOH (100 mL) and H_2O (50 mL). The solution was cooled to 0 ^{o}C and $LiOH{\cdot}H_2O$ (10.7 g, 255 mmol) was added in batches. The mixture was warmed to room temperature gradually and stirred for another 3 h. H_2O (200 mL) was then added and most of the organic solvent was removed under reduced pressure. The aqueous phase was extracted with Et_2O (30 mL × 2), and acidized with concentrated HCl (36 wt%, 30 mL). The aqueous phase was extracted with CH_2Cl_2 (100 mL × 4). The combined

organic phase was dried over anhydrous Na_2SO_4 and concentrated under reduced pressure to give compound **S2** (11.9 g) in 80% yield for two steps.

Step 2:
To a stirred solution of **S2** (5.25 g, 30 mmol) in toluene (100 mL) was added DPPA (6.8 mL, 31.5 mmol) and Et_3N (5.0 mL, 36 mmol). The mixture was heated at 90 ^oC and nitrogen gas release was observed. After completion of the gas release (in about 30 min), solvent was removed under reduced pressure. The residue was dissolved in 1,4-Dioxane (50 mL) and 6N HCl (50 mL) was added. The mixture was heated at 60 ^oC for 3 h. After cooled to room temperature, the solution was diluted with H_2O (100 mL). The aqueous phase was extracted with EtOAc (20 mL × 3) and concentrated under reduced pressure to give a mixture of compound **S3** and $Et_3N{\cdot}HCl$ (9.4 g). This mixture containing **S3** could be used conveniently for the next step. The yield of **S3** was 70%, which was determined by ^1H NMR of the mixture using CH_2Br_2 as internal standard (the mass fraction of **S3** was 40.8% in the mixture).

Alternatively, pure **S3** could be prepared as follows:
To a stirred solution of **S2** (88 mg, 0.5 mmol) in toluene (5 mL) was added DPPA (108 μL, 0.5 mmol) and Et_3N (83 μL, 0.6 mmol). The mixture was heated at 90 ^oC and nitrogen gas release was observed. After completion of the gas release (about 30 min), the reaction was cooled to room temperature. Then H_2O (15 mL) and sat. $NaHCO_3$ solution (5 mL) was added. The layers were separated and the aqueous phase was extracted with EtOAc (10 mL × 3). Organic phases were combined and the solvent was removed under reduced pressure. The residue was dissolved in 1,4-Dioxane (3 mL) and 6N HCl (3 mL) was added. The mixture was heated at 60 ^oC for 3 h. After cooled to room temperature, the solution was diluted with H_2O (15 mL). The aqueous phase was extracted with EtOAc (15 mL × 3) and concentrated under reduced pressure to give pure **S3** (64 mg) in 70% yield.

Step 3:
To a suspension of **S3** (actual mass: 3.8 g, 20.9 mmol, mixed with $Et_3N{\cdot}HCl$) and NsCl (4.6 g, 20.9 mmol) in CH_2Cl_2 (100 mL) at -30 ^oC was added Et_3N (7.3 mL, 52.2 mmol). The mixture was gradually warmed to room temperature and stirred for another 5 h. Then H_2O (150 mL) was added and the organic phase was separated. The aqueous phase was extracted with CH_2Cl_2 (100 mL × 2). The combined organic phase was dried over anhydrous Na_2SO_4 and concentrated under reduced pressure. The residue was purified by silica gel chromatography with petroleum ether/EtOAc (1:1) to afford compound **5** (6.5 g) in 94% yield.

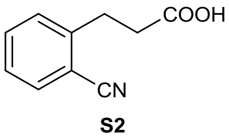

S2

3-(2-cyanophenyl)propanoic acid: ^1H NMR (400 MHz, $CDCl_3$) δ 7.64 (d, *J* = 7.7 Hz, 1H), 7.53 (td, *J* = 7.8, 1.1 Hz, 1H), 7.38 (d, *J* = 7.8 Hz, 1H), 7.33 (t, *J* = 7.6 Hz, 1H), 3.19 (t, *J* = 7.6 Hz, 2H), 2.78 (t, *J* = 7.6 Hz, 2H). ^{13}C NMR (101 MHz, $CDCl_3$) δ 177.8, 144.1, 133.2, 133.1, 129.7, 127.3, 117.8, 112.6, 34.4, 29.2. HRMS (m/z, ESI-TOF): Calcd for $C_{10}H_{10}NO_2^+$ $[M+H^+]$ 176.0706, found 176.0703.

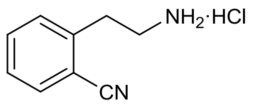

S3 **2-(2-aminoethyl)benzonitrile hydrochloride**: ^{1}H NMR (400 MHz, D_2O) δ 7.50 (d, *J* = 7.7 Hz, 1H), 7.45 (t, *J* = 7.7 Hz, 1H), 7.27 (d, *J* = 7.8 Hz, 1H), 7.23 (t, *J* = 7.7 Hz, 1H), 3.11 (t, *J* = 7.6 Hz, 2H), 3.03-2.91 (m, 2H). ^{13}C NMR (101 MHz, D_2O) δ 139.9, 133.9, 133.4, 130.0, 128.0, 118.1, 111.2, 39.4, 31.4.

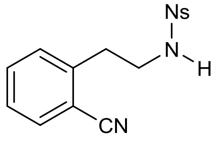

5 ***N*-(2-cyanophenethyl)-4-nitrobenzenesulfonamide**: ^{1}H NMR (400 MHz, $CDCl_3$) δ 8.31 (d, *J* = 8.5 Hz, 2H), 7.99 (d, *J* = 8.5 Hz, 2H), 7.58 (d, *J* = 7.7 Hz, 1H), 7.53 (t, *J* = 7.6 Hz, 1H), 7.34 (t, *J* = 8.2 Hz, 2H), 4.89 (t, *J* = 6.1 Hz, 1H), 3.38 (q, *J* = 6.6 Hz, 2H), 3.06 (t, *J* = 6.9 Hz, 2H). ^{13}C NMR (101 MHz, $CDCl_3$) δ 150.2, 145.9, 141.5, 133.4, 133.2, 130.5, 128.4, 127.8, 124.6, 117.8, 112.8, 43.7, 35.1. HRMS (m/z, ESI-TOF): Calcd for $C_{15}H_{14}N_3O_4S^+$ [M+H$^+$] 332.0700, found 332.0702.

Preparation of substrates (1a-1za) with auxiliary 5:

method a*: using acyl chloride (for substrates* **1a** *and* **1e***)*

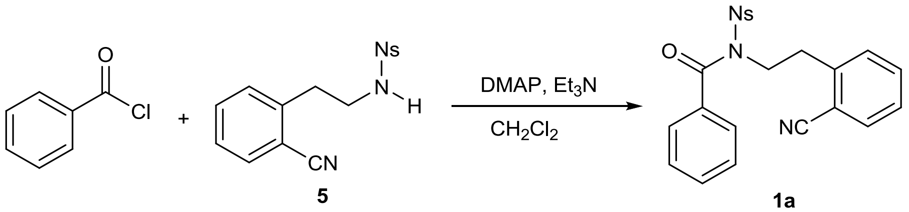

To a stirred solution of auxiliary **5** (3.3 g, 10 mmol) and DMAP (122 mg, 0.1 mmol) in CH_2Cl_2 (100 mL) was added benzoyl chloride (2.3 mL, 20 mmol) dropwise at room temperature. With vigorous stirring, Et_3N (2.8 mL, 20 mmol) was added dropwise afterwards. The mixture was stirred for another 2 h at room temperature and then quenched by addition of H_2O (100 mL). The organic phase was separated and the aqueous phase was extracted with CH_2Cl_2 (50 mL × 2). The combined organic phase was dried over anhydrous Na_2SO_4 and concentrated under reduced pressure. The residue was purified by silica gel chromatography with petroleum ether/EtOAc (2:1) to afford compound **1a** (3.9 g) in 90% yield.

method b*: using carboxylic acid (for substrates* **1b**-**1za**, *except* **1e***)*

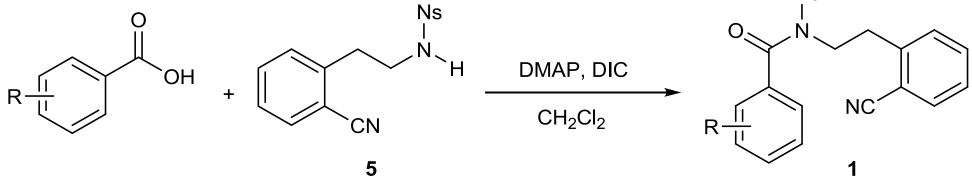

General procedure: To a solution of auxiliary **5** (331 mg, 1.0 mmol), benzoic acid (1.3 mmol) and DMAP (12 mg, 0.1 mmol) in CH_2Cl_2 (10 mL) under N_2 was added DIC (186 μL, 1.2 mmol)

at room temperature. The resulting mixture was stirred for another 10-24 h. The insoluble substance was filtered off and the filter cake was washed with CH_2Cl_2 (40 mL). The combined filtrate was washed successively with 1N HCl (20 mL) and saturated $NaHCO_3$ aqueous solution (20 mL), and then dried over anhydrous Na_2SO_4. The solvent was removed under reduced pressure and the residue was purified by silica gel chromatography with petroleum ether/EtOAc (2:1) to afford compound **1** in 70-90% yields.

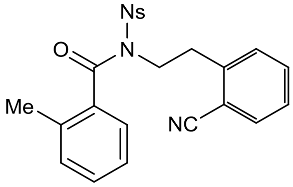

1b ***N*-(2-cyanophenethyl)-2-methyl-*N*-(4-nitrophenylsulfonyl)benzamide**: ^{1}H NMR (400 MHz, $CDCl_3$) δ 8.35 (d, *J* = 8.7 Hz, 2H), 8.13 (d, *J* = 8.8 Hz, 2H), 7.62-7.51 (m, 2H), 7.40-7.28 (m, 3H), 7.18-7.09 (m, 2H), 6.82 (d, *J* = 7.6 Hz, 1H), 4.13 (t, *J* = 7.3 Hz, 2H), 3.24 (t, *J* = 7.3 Hz, 2H), 2.04 (s, 3H). ^{13}C NMR (101 MHz, $CDCl_3$) δ 170.8, 150.8, 144.5, 140.9, 135.1, 133.7, 133.3, 130.9, 130.9, 130.8, 130.3, 127.9, 126.5, 126.0, 124.1, 117.4, 113.1, 48.1, 35.2, 19.0. HRMS (m/z, ESI-TOF): Calcd for $C_{23}H_{20}N_3O_5S^+$ [M+H$^+$] 450.1118, found 450.1126.

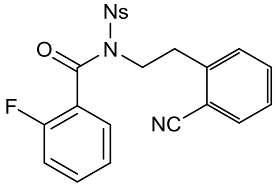

1c ***N*-(2-cyanophenethyl)-2-fluoro-*N*-(4-nitrophenylsulfonyl)benzamide**: ^{1}H NMR (400 MHz, $CDCl_3$) δ 8.35 (d, *J* = 8.8 Hz, 2H), 8.14 (d, *J* = 8.8 Hz, 2H), 7.59 (t, *J* = 7.6 Hz, 1H), 7.52 (d, *J* = 7.6 Hz, 1H), 7.50-7.42 (m, 2H), 7.38 (t, *J* = 7.6 Hz, 1H), 7.08 (t, *J* = 7.6 Hz, 1H), 7.03 (t, *J* = 9.1 Hz, 1H), 6.81 (t, *J* = 6.6 Hz, 1H), 4.24 (t, *J* = 6.7 Hz, 2H), 3.25 (t, *J* = 6.8 Hz, 2H). ^{13}C NMR (101 MHz, $CDCl_3$) δ 166.4, 158.0 (d, J_{C-F} = 251 Hz), 150.9, 144.1, 140.7, 133.6 (d, J_{C-F} = 8.4 Hz), 133.3, 133.2, 131.4, 130.2, 129.2 (d, J_{C-F} = 2.4 Hz), 127.9, 124.9 (d, J_{C-F} = 3.4 Hz), 124.2, 122.2 (d, J_{C-F} = 15.7 Hz), 117.3, 116.1 (d, J_{C-F} = 20.9 Hz), 112.9, 48.1 (d, J_{C-F} = 2.0 Hz), 35.4. HRMS (m/z, ESI-TOF): Calcd for $C_{22}H_{17}FN_3O_5S^+$ [M+H$^+$] 454.0867, found 454.0875.

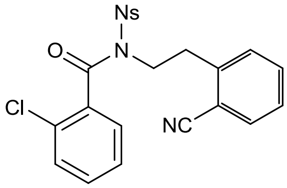

1d **2-chloro-*N*-(2-cyanophenethyl)-*N*-(4-nitrophenylsulfonyl)benzamide**: ^{1}H NMR (400 MHz, $CDCl_3$) δ 8.36 (d, *J* = 8.8 Hz, 2H), 8.16 (d, *J* = 8.9 Hz, 2H), 7.64-7.54 (m, 2H), 7.45 (d, *J* = 7.7 Hz, 1H), 7.43-7.36 (m, 2H), 7.30 (d, *J* = 8.1 Hz, 1H), 7.22 (t, *J* = 7.5 Hz, 1H), 6.77 (d, *J* = 7.6 Hz, 1H), 4.13 (brs, 2H), 3.27 (t, *J* = 6.9 Hz, 2H). ^{13}C NMR (101 MHz, $CDCl_3$) δ 167.7, 150.9, 143.8, 140.8, 133.3, 133.3, 132.0, 131.3, 130.5, 130.1, 129.8, 128.5, 127.9, 127.4, 124.1, 117.3, 113.1, 48.3, 35.4. HRMS (m/z, ESI-TOF): Calcd for $C_{22}H_{17}ClN_3O_5S^+$ [M+H$^+$] 470.0572, found 470.0578.

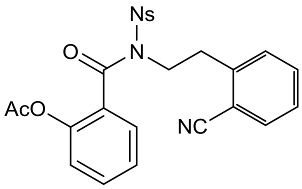

1e **2-((2-cyanophenethyl)(4-nitrophenylsulfonyl)carbamoyl)phenyl acetate**: ^{1}H NMR (400 MHz, $CDCl_3$) δ 8.37 (d, *J* = 8.9 Hz, 2H), 8.24 (d, *J* = 8.8 Hz, 2H), 7.57 (d, *J* = 7.7 Hz, 1H), 7.55-7.48 (m, 2H), 7.35 (t, *J* = 7.6 Hz, 1H), 7.31-7.26 (m, 2H), 7.21 (dd, *J* = 7.6, 1.3 Hz, 1H), 7.16 (d, *J* = 8.2 Hz, 1H), 4.06 (t, *J* = 7.6 Hz, 2H), 3.28 (t, *J* = 7.6 Hz, 2H), 2.06 (s, 3H). ^{13}C NMR (101 MHz, $CDCl_3$) δ 168.6, 167.9, 150.8, 147.2, 144.3, 140.8, 133.3, 133.2, 132.5, 130.5, 130.5, 127.8, 127.1, 126.3, 124.1, 123.2, 117.5, 112.8, 48.6, 35.1, 20.8. HRMS (m/z, ESI-TOF): Calcd for $C_{24}H_{20}N_3O_7S^+$ [M+H$^+$] 494.1016, found 494.1017.

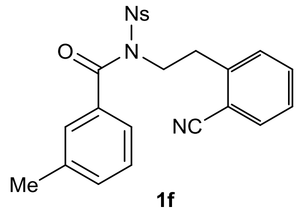

1f ***N*-(2-cyanophenethyl)-3-methyl-*N*-(4-nitrophenylsulfonyl)benzamide**: ^{1}H NMR (400 MHz, $CDCl_3$) δ 8.35 (d, *J* = 8.8 Hz, 2H), 8.20 (d, *J* = 8.9 Hz, 2H), 7.57-7.48 (m, 2H), 7.38-7.24 (m, 4H), 7.05 (d, *J* = 7.5 Hz, 1H), 6.96 (s, 1H), 4.26 (t, *J* = 7.0 Hz, 2H), 3.19 (t, *J* = 7.0 Hz, 2H), 2.31 (s, 3H). ^{13}C NMR (101 MHz, $CDCl_3$) δ 171.2, 150.7, 144.4, 140.8, 138.7, 133.4, 133.2, 133.1, 133.1, 130.9, 130.3, 128.6, 128.3, 127.8, 124.8, 124.2, 117.3, 112.9, 48.5, 35.1, 21.3. HRMS (m/z, ESI-TOF): Calcd for $C_{23}H_{20}N_3O_5S^+$ [M+H$^+$] 450.1118, found 450.1125.

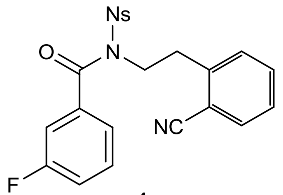

1g ***N*-(2-cyanophenethyl)-3-fluoro-*N*-(4-nitrophenylsulfonyl)benzamide**: ^{1}H NMR (400 MHz, $CDCl_3$) δ 8.38 (d, *J* = 8.5 Hz, 2H), 8.19 (d, *J* = 8.5 Hz, 2H), 7.61-7.48 (m, 2H), 7.43-7.34 (m, 2H), 7.31 (d, *J* = 7.6 Hz, 1H), 7.21 (t, *J* = 8.2 Hz, 1H), 7.06 (d, *J* = 7.4 Hz, 1H), 6.81 (d, *J* = 8.5 Hz, 1H), 4.24 (t, *J* = 6.9 Hz, 2H), 3.21 (t, *J* = 6.8 Hz, 2H). ^{13}C NMR (101 MHz, $CDCl_3$) δ 169.7 (d, J_{C-F} = 2.6 Hz), 162.3 (d, J_{C-F} = 251 Hz), 150.9, 144.0, 140.6, 135.4 (d, J_{C-F} = 7.2 Hz), 133.3, 133.3, 131.1, 130.6 (d, J_{C-F} = 8.0 Hz), 130.3, 128.1, 124.4, 123.4 (d, J_{C-F} = 3.2 Hz), 119.5 (d, J_{C-F} = 21 Hz), 117.3, 115.2 (d, J_{C-F} = 24 Hz), 112.9, 48.5, 35.1. HRMS (m/z, ESI-TOF): Calcd for $C_{22}H_{17}FN_3O_5S^+$ [M+H$^+$] 454.0867, found 454.0872.

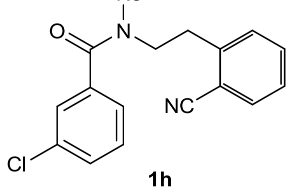

1h **3-chloro-*N*-(2-cyanophenethyl)-*N*-(4-nitrophenylsulfonyl)benzamide**: ^{1}H NMR (400 MHz, $CDCl_3$) δ 8.37 (d, *J* = 8.9 Hz, 2H), 8.16 (d, *J* = 8.9 Hz, 2H), 7.59-7.51 (m, 2H), 7.50-7.44 (m, 1H), 7.39 (t, *J* = 7.6 Hz, 1H), 7.36-7.28 (m, 2H), 7.17 (d, *J* = 7.7 Hz, 1H), 6.99 (t, *J* = 1.6 Hz, 1H), 4.25 (t, *J* = 6.9 Hz, 2H), 3.21 (t, *J* = 6.9 Hz, 2H). ^{13}C NMR (101 MHz, $CDCl_3$) δ 169.6, 150.9, 144.1, 140.6, 135.2, 134.9, 133.3, 133.3, 132.3, 131.1, 130.3, 130.0,

128.1, 127.9, 125.7, 124.4, 117.3, 112.9, 48.4, 35.0. HRMS (m/z, ESI-TOF): Calcd for $C_{22}H_{17}ClN_3O_5S^+$ [M+H$^+$] 470.0572, found 470.0580.

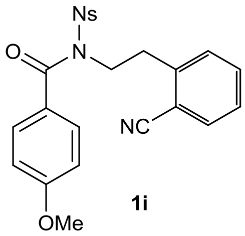
1i

***N*-(2-cyanophenethyl)-4-methoxy-*N*-(4-nitrophenylsulfonyl)benzamide**: ^{1}H NMR (400 MHz, $CDCl_3$) δ 8.36 (d, *J* = 8.9 Hz, 2H), 8.16 (d, *J* = 8.9 Hz, 2H), 7.58-7.47 (m, 2H), 7.42 (d, *J* = 8.8 Hz, 2H), 7.34 (t, *J* = 7.6 Hz, 1H), 7.24 (d, *J* = 7.8 Hz, 1H), 6.88 (d, *J* = 8.8 Hz, 2H), 4.17 (t, *J* = 7.2 Hz, 2H), 3.86 (s, 3H), 3.16 (t, *J* = 7.2 Hz, 2H). ^{13}C NMR (101 MHz, $CDCl_3$) δ 170.8, 163.6, 150.7, 144.4, 140.9, 133.2, 133.2, 131.2, 130.8, 130.1, 127.9, 125.7, 124.3, 117.5, 114.1, 112.9, 55.7, 48.5, 34.7. HRMS (m/z, ESI-TOF): Calcd for $C_{23}H_{20}N_3O_6S^+$ [M+H$^+$] 466.1067, found 466.1073.

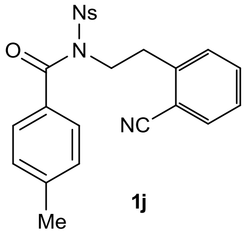
1j

***N*-(2-cyanophenethyl)-4-methyl-*N*-(4-nitrophenylsulfonyl)benzamide**: ^{1}H NMR (400 MHz, $CDCl_3$) δ 8.35 (d, *J* = 8.9 Hz, 2H), 8.18 (d, *J* = 8.9 Hz, 2H), 7.57-7.48 (m, 2H), 7.34 (t, *J* = 7.6 Hz, 1H), 7.30-7.14 (m, 5H), 4.21 (t, *J* = 7.2 Hz, 2H), 3.18 (t, *J* = 7.1 Hz, 2H), 2.40 (s, 3H). ^{13}C NMR (101 MHz, $CDCl_3$) δ 171.2, 150.7, 144.4, 143.5, 140.8, 133.2, 133.2, 130.9, 130.7, 130.2, 129.4, 128.3, 127.8, 124.3, 117.4, 112.9, 48.5, 34.9, 21.8. HRMS (m/z, ESI-TOF): Calcd for $C_{23}H_{20}N_3O_5S^+$ [M+H$^+$] 450.1118, found 450.1121.

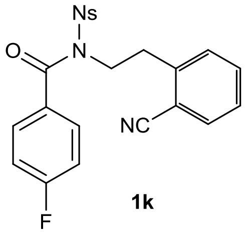
1k

***N*-(2-cyanophenethyl)-4-fluoro-*N*-(4-nitrophenylsulfonyl)benzamide**: ^{1}H NMR (400 MHz, $CDCl_3$) δ 8.37 (d, *J* = 8.9 Hz, 2H), 8.16 (d, *J* = 8.9 Hz, 2H), 7.59-7.48 (m, 2H), 7.40-7.32 (m, 3H), 7.28 (d, *J* = 8.0 Hz, 1H), 7.07 (t, *J* = 8.5 Hz, 2H), 4.19 (t, *J* = 7.1 Hz, 2H), 3.19 (t, *J* = 7.1 Hz, 2H). ^{13}C NMR (101 MHz, $CDCl_3$) δ 170.2, 165.2 (d, J_{C-F} = 256 Hz), 150.8, 144.1, 140.7, 133.3, 131.0, 130.9, 130.2, 129.8 (d, *J* = 3.4 Hz), 128.0, 124.4, 117.4, 116.2, 116.0, 112.9, 48.4, 34.8. HRMS (m/z, ESI-TOF): Calcd for $C_{22}H_{17}FN_3O_5S^+$ [M+H$^+$] 454.0867, found 454.0874.

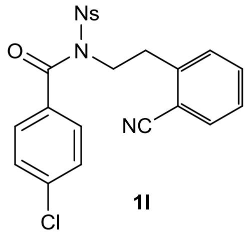

1l

4-chloro-*N*-(2-cyanophenethyl)-*N*-(4-nitrophenylsulfonyl)benzamide: ^{1}H NMR (400 MHz, $CDCl_3$) δ 8.37 (d, *J* = 9.0 Hz, 2H), 8.15 (d, *J* = 9.0 Hz, 2H), 7.58-7.51 (m, 2H), 7.40-7.33 (m, 3H), 7.30-7.26 (m, 3H), 4.18 (t, *J* = 7.2 Hz, 2H), 3.20 (t, *J* = 7.1 Hz, 2H). ^{13}C NMR (101 MHz, $CDCl_3$) δ 170.2, 150.9, 144.1, 140.7, 139.1, 133.3, 132.1, 130.9, 130.2, 129.7, 129.1, 128.0, 124.4, 117.4, 112.9, 48.3, 34.8. HRMS (m/z, ESI-TOF): Calcd for $C_{22}H_{17}ClN_3O_5S^+$ [M+H$^+$] 470.0572, found 470.0577.

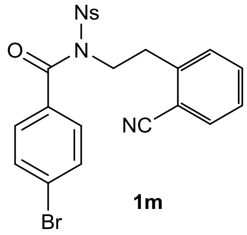

1m

4-bromo-*N*-(2-cyanophenethyl)-*N*-(4-nitrophenylsulfonyl)benzamide: ^{1}H NMR (400 MHz, $CDCl_3$) δ 8.37 (d, *J* = 8.8 Hz, 2H), 8.14 (d, *J* = 8.8 Hz, 2H), 7.61-7.47 (m, 4H), 7.36 (t, *J* = 7.4 Hz, 1H), 7.28 (d, *J* = 7.9 Hz, 1H), 7.19 (d, *J* = 8.4 Hz, 2H), 4.18 (t, *J* = 7.1 Hz, 2H), 3.19 (t, *J* = 7.1 Hz, 2H). ^{13}C NMR (101 MHz, $CDCl_3$) δ 170.3, 150.9, 144.0, 140.7, 133.3, 132.5, 132.0, 130.9, 130.2, 129.7, 128.0, 127.5, 124.4, 117.4, 112.9, 48.3, 34.8. HRMS (m/z, ESI-TOF): Calcd for $C_{22}H_{17}BrN_3O_5S^+$ [M+H$^+$] 514.0067, found 514.0071.

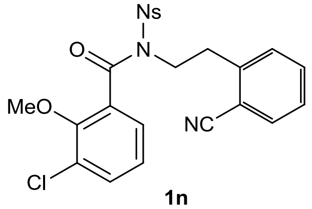

1n

3-chloro-*N*-(2-cyanophenethyl)-2-methoxy-*N*-(4-nitrophenylsulfonyl)benzamide: ^{1}H NMR (400 MHz, $CDCl_3$) δ 8.35 (d, *J* = 8.9 Hz, 2H), 8.10 (d, *J* = 8.9 Hz, 2H), 7.62-7.52 (m, 2H), 7.45 (dd, *J* = 8.0, 1.5 Hz, 1H), 7.37 (t, *J* = 8.0 Hz, 2H), 7.06 (t, *J* = 7.9 Hz, 1H), 6.85 (dd, *J* = 7.7, 1.5 Hz, 1H), 4.13 (t, *J* = 7.1 Hz, 2H), 3.54 (s, 3H), 3.23 (t, *J* = 7.2 Hz, 2H). ^{13}C NMR (101 MHz, $CDCl_3$) δ 167.8, 152.1, 150.8, 144.1, 140.9, 133.2, 133.2, 133.2, 130.9, 130.6, 130.2, 128.2, 127.9, 126.5, 125.5, 124.0, 117.5, 113.1, 62.2, 48.1, 35.2. HRMS (m/z, ESI-TOF): Calcd for $C_{23}H_{19}ClN_3O_6S^+$ [M+H$^+$] 500.0678, found 500.0681.

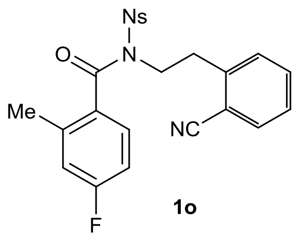

1o

***N*-(2-cyanophenethyl)-4-fluoro-2-methyl-*N*-(4-nitrophenylsulfonyl)benzamide**: ^{1}H NMR (400 MHz, $CDCl_3$) δ 8.36 (d, *J* = 8.9 Hz, 2H), 8.12 (d, *J* = 8.9 Hz, 2H), 7.65-7.50 (m, 2H), 7.44-7.29 (m, 2H), 6.98-6.74 (m, 3H), 4.13 (t, *J* = 7.3 Hz, 2H), 3.23 (t, *J* = 7.3 Hz, 2H), 2.05 (s, 3H). ^{13}C NMR (101 MHz, $CDCl_3$) δ 170.0, 163.7 (d, $J_{C\text{-}F}$ = 253 Hz), 150.8, 144.4, 140.7, 138.7 (d, $J_{C\text{-}F}$ = 8.6 Hz), 133.3, 133.3, 130.8, 130.3, 129.8 (d, $J_{C\text{-}F}$

= 3.3 Hz), 128.9 (d, J_{C-F} = 9.1 Hz), 128.0, 124.2, 117.9 (d, J_{C-F} = 22 Hz), 117.4, 113.2 (d, J_{C-F} = 22 Hz), 113.0, 48.1, 35.1, 19.2. HRMS (m/z, ESI-TOF): Calcd for $C_{23}H_{19}FN_3O_5S^+$ [M+H$^+$] 468.1024, found 468.1026.

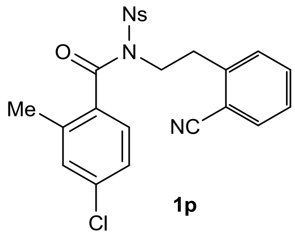

1p

4-chloro-*N*-(2-cyanophenethyl)-2-methyl-*N*-(4-nitrophenylsulfonyl)benzamide: ^{1}H NMR (400 MHz, $CDCl_3$) δ 8.36 (d, *J* = 8.9 Hz, 2H), 8.10 (d, *J* = 8.9 Hz, 2H), 7.63-7.52 (m, 2H), 7.38 (t, *J* = 7.7 Hz, 1H), 7.34 (d, *J* = 7.7 Hz, 1H), 7.17 (s, 1H), 7.12 (d, *J* = 8.2 Hz, 1H), 6.79 (d, *J* = 8.2 Hz, 1H), 4.12 (t, *J* = 7.3 Hz, 2H), 3.24 (t, *J* = 7.4 Hz, 2H), 2.04 (s, 3H). ^{13}C NMR (101 MHz, $CDCl_3$) δ 169.9, 150.9, 144.3, 140.7, 137.4, 136.9, 133.3, 133.3, 132.2, 130.9, 130.8, 130.2, 128.0, 127.9, 126.2, 124.2, 117.4, 113.0, 48.0, 35.1, 19.0. HRMS (m/z, ESI-TOF): Calcd for $C_{23}H_{19}ClN_3O_5S^+$ [M+H$^+$] 484.0728, found 484.0731.

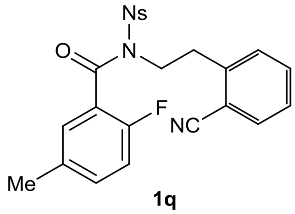

1q

***N*-(2-cyanophenethyl)-2-fluoro-5-methyl-*N*-(4-nitrophenylsulfonyl)benzamide**: ^{1}H NMR (400 MHz, $CDCl_3$) δ 8.35 (d, *J* = 8.9 Hz, 2H), 8.16 (d, *J* = 8.9 Hz, 2H), 7.59 (td, *J* = 7.7, 1.1 Hz, 1H), 7.51 (d, *J* = 7.7 Hz, 1H), 7.46 (d, *J* = 7.7 Hz, 1H), 7.39 (t, *J* = 7.6 Hz, 1H), 7.25-7.18 (m, 1H), 6.91 (t, *J* = 9.0 Hz, 1H), 6.48 (d, *J* = 6.1 Hz, 1H), 4.24 (t, *J* = 6.5 Hz, 2H), 3.25 (t, *J* = 6.7 Hz, 2H), 2.22 (s, 3H). ^{13}C NMR (101 MHz, $CDCl_3$) δ 166.7, 156.2 (d, J_{C-F} = 248 Hz), 150.9, 144.2, 140.8, 134.7 (d, J_{C-F} = 3.4 Hz), 134.1 (d, J_{C-F} = 8.0 Hz), 133.3, 133.1, 131.6, 130.3, 129.2 (d, J_{C-F} = 2.2 Hz), 127.8, 124.2, 121.6 (d, J_{C-F} = 15.8 Hz), 117.2, 115.8 (d, J_{C-F} = 21 Hz), 113.1, 48.2, 35.5, 20.6. HRMS (m/z, ESI-TOF): Calcd for $C_{23}H_{19}FN_3O_5S^+$ [M+H$^+$] 468.1024, found 468.1030.

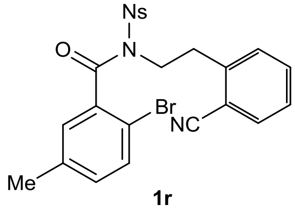

1r

2-bromo-*N*-(2-cyanophenethyl)-5-methyl-*N*-(4-nitrophenylsulfonyl)benzamide: ^{1}H NMR (400 MHz, $CDCl_3$) δ 8.36 (d, *J* = 9.0 Hz, 2H), 8.21 (d, *J* = 9.0 Hz, 2H), 7.61 (td, *J* = 7.7, 1.2 Hz, 1H), 7.56 (d, *J* = 7.7 Hz, 1H), 7.47 (d, *J* = 7.6 Hz, 1H), 7.40 (t, *J* = 7.6 Hz, 1H), 7.33 (d, *J* = 8.2 Hz, 1H), 7.10 (dd, *J* = 8.2, 1.3 Hz, 1H), 6.53 (s, 1H), 4.11 (brs, 2H), 3.28 (t, *J* = 7.0 Hz, 2H), 2.24 (s, 3H). ^{13}C NMR (101 MHz, $CDCl_3$) δ 168.4, 151.0, 143.8, 140.8, 138.3, 135.0, 133.3, 133.1, 133.0, 132.7, 131.3, 130.8, 129.0, 127.8, 124.0, 117.2, 115.3, 113.2, 48.6, 35.5, 21.0. HRMS (m/z, ESI-TOF): Calcd for $C_{23}H_{19}BrN_3O_5S^+$ [M+H$^+$] 528.0223, found 528.0232.

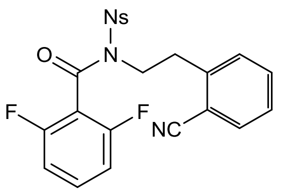

1s

***N*-(2-cyanophenethyl)-2,6-difluoro-*N*-(4-nitrophenylsulfonyl)benzamide**: ^{1}H NMR (400 MHz, $CDCl_3$) δ 8.35 (d, *J* = 8.9 Hz, 2H), 8.04 (d, *J* = 8.8 Hz, 2H), 7.67-7.51 (m, 2H), 7.49-7.31 (m, 3H), 6.91 (t, *J* = 8.1 Hz, 2H), 4.20 (t, *J* = 7.2 Hz, 2H), 3.29 (t, *J* = 7.2 Hz, 2H). ^{13}C NMR (101 MHz, $CDCl_3$) δ 161.5, 158.8 (dd, $J_{C\text{-}F}$ = 253, 6.9 Hz), 150.9, 143.8, 140.6, 133.4, 133.2, 133.1 (t, $J_{C\text{-}F}$ = 10 Hz), 131.2, 129.8, 128.0, 124.4, 117.5, 112.9 (t, $J_{C\text{-}F}$ = 21 Hz), 112.7, 112.1 (dd, $J_{C\text{-}F}$ = 21, 3.4 Hz), 47.8, 34.9. HRMS (m/z, ESI-TOF): Calcd for $C_{22}H_{16}F_2N_3O_5S^+$ [M+H$^+$] 472.0773, found 472.0773.

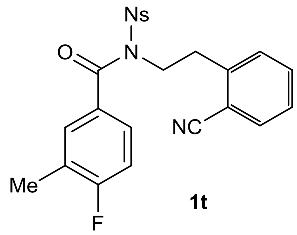

1t

***N*-(2-cyanophenethyl)-4-fluoro-3-methyl-*N*-(4-nitrophenylsulfonyl)benzamide**: ^{1}H NMR (400 MHz, $CDCl_3$) δ 8.37 (d, *J* = 8.8 Hz, 2H), 8.17 (d, *J* = 8.8 Hz, 2H), 7.61-7.46 (m, 2H), 7.35 (t, *J* = 7.6 Hz, 1H), 7.27 (d, *J* = 7.6 Hz, 1H), 7.21-7.14 (m, 1H), 7.12 (d, *J* = 6.8 Hz, 1H), 7.00 (t, *J* = 8.7 Hz, 1H), 4.20 (t, *J* = 7.1 Hz, 2H), 3.18 (t, *J* = 7.0 Hz, 2H), 2.24 (s, 3H). ^{13}C NMR (101 MHz, $CDCl_3$) δ 170.4, 163.8 (d, $J_{C\text{-}F}$ = 254 Hz), 150.8, 144.2, 140.8, 133.3, 133.2, 132.0 (d, $J_{C\text{-}F}$ = 6.4 Hz), 131.0, 130.2, 129.4 (d, $J_{C\text{-}F}$ = 3.6 Hz), 128.0 (d, $J_{C\text{-}F}$ = 9.3 Hz), 127.9, 126.0 (d, $J_{C\text{-}F}$ = 18.2 Hz), 124.3, 117.4, 115.5 (d, $J_{C\text{-}F}$ = 24 Hz), 112.9, 48.4, 34.9 , 14.6 (d, $J_{C\text{-}F}$ = 3.4 Hz). HRMS (m/z, ESI-TOF): Calcd for $C_{23}H_{19}FN_3O_5S^+$ [M+H$^+$] 468.1024, found 468.1029.

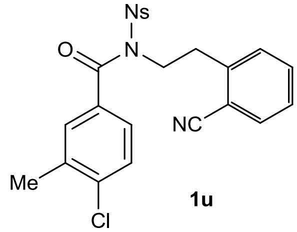

1u

4-chloro-*N*-(2-cyanophenethyl)-3-methyl-*N*-(4-nitrophenylsulfonyl)benzamide: ^{1}H NMR (400 MHz, $CDCl_3$) δ 8.37 (d, *J* = 8.9 Hz, 2H), 8.17 (d, *J* = 8.9 Hz, 2H), 7.58-7.50 (m, 2H), 7.40-7.32 (m, 2H), 7.28 (d, *J* = 8.2 Hz, 1H), 7.14-7.05 (m, 2H), 4.20 (t, *J* = 7.1 Hz, 2H), 3.19 (t, *J* = 7.0 Hz, 2H), 2.34 (s, 3H). ^{13}C NMR (101 MHz, $CDCl_3$) δ 170.4, 150.8, 144.2, 140.7, 139.2, 137.1, 133.3, 133.2, 132.0, 131.0, 130.5, 130.2, 129.4, 127.9, 126.7, 124.4, 117.4, 112.9, 48.4, 34.9, 20.2. HRMS (m/z, ESI-TOF): Calcd for $C_{23}H_{19}ClN_3O_5S^+$ [M+H$^+$] 484.0728, found 484.0732.

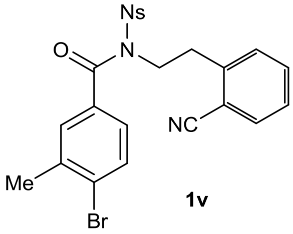

1v

4-bromo-*N*-(2-cyanophenethyl)-3-methyl-*N*-(4-nitrophenylsulfonyl)benzamide: ^{1}H NMR (400 MHz, $CDCl_3$) δ 8.37 (d, J = 8.8 Hz, 2H), 8.16 (d, J = 8.8 Hz, 2H), 7.57-7.49 (m, 3H), 7.36 (t, J = 7.5 Hz, 1H), 7.28 (d, J = 7.8 Hz, 1H), 7.09 (s, 1H), 6.99 (dd, J = 8.0, 1.2 Hz, 1H), 4.20 (t, J = 7.1 Hz, 2H), 3.19 (t, J = 7.1 Hz, 2H), 2.36 (s, 3H). ^{13}C NMR (101 MHz, $CDCl_3$) δ 170.4, 150.8, 144.1, 140.7, 139.0, 133.3, 133.2, 132.7, 132.7, 130.9, 130.2, 130.1, 129.8, 127.9, 126.6, 124.4, 117.4, 112.9, 48.3, 34.9, 23.0. HRMS (m/z, ESI-TOF): Calcd for $C_{23}H_{19}BrN_3O_5S^+$ [M+H$^+$] 528.0223, found 528.0232.

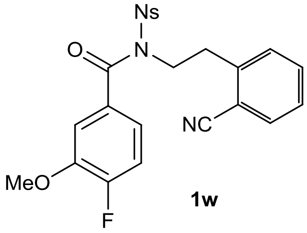

1w

***N*-(2-cyanophenethyl)-4-fluoro-3-methoxy-*N*-(4-nitrophenylsulfonyl)benzamide**: ^{1}H NMR (400 MHz, $CDCl_3$) δ 8.38 (d, J = 9.0 Hz, 2H), 8.18 (d, J = 9.0 Hz, 2H), 7.61-7.48 (m, 2H), 7.35 (td, J = 7.6, 1.0 Hz, 1H), 7.28 (s, 1H), 7.15-7.04 (m, 1H), 7.03-6.89 (m, 2H), 4.22 (t, J = 7.2 Hz, 2H), 3.86 (s, 3H), 3.18 (t, J = 7.2 Hz, 2H). ^{13}C NMR (101 MHz, $CDCl_3$) δ 170.2, 155.1 (d, J_{C-F} = 256 Hz), 150.8, 148.2 (d, J_{C-F} = 11.1 Hz), 144.1, 140.8, 133.2, 130.8, 130.2, 129.9 (d, J_{C-F} = 3.8 Hz), 128.0, 124.4, 121.6 (d, J_{C-F} = 7.9 Hz), 117.4, 116.3 (d, J_{C-F} = 19.4 Hz), 113.9 (d, J_{C-F} = 2.9 Hz), 112.9, 56.5, 48.5, 34.9. HRMS (m/z, ESI-TOF): Calcd for $C_{23}H_{19}FN_3O_6S^+$ [M+H$^+$] 484.0973, found 484.0975.

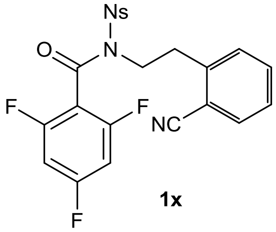

1x

***N*-(2-cyanophenethyl)-2,4,6-trifluoro-*N*-(4-nitrophenylsulfonyl)benzamide**: ^{1}H NMR (400 MHz, $CDCl_3$) δ 8.38 (d, J = 8.9 Hz, 2H), 8.06 (d, J = 8.8 Hz, 2H), 7.67-7.52 (m, 2H), 7.44 (d, J = 7.6 Hz, 1H), 7.39 (t, J = 7.6 Hz, 1H), 6.70 (t, J = 8.1 Hz, 2H), 4.17 (t, J = 7.3 Hz, 2H), 3.28 (t, J = 7.3 Hz, 2H). ^{13}C NMR (101 MHz, $CDCl_3$) δ 165.8-165.5 (m, multiplet due to the couplings of carbon and three fluorine atoms), 163.2-162.9 (m), 160.6, 158.3-158.1 (m), 151.0, 143.7, 140.5, 133.5, 133.2, 131.2, 129.8, 128.1, 124.5, 117.6, 112.7, 101.3 (td, J_{C-F} = 26, 3.9 Hz), 47.8, 34.8. HRMS (m/z, ESI-TOF): Calcd for $C_{22}H_{15}F_3N_3O_5S^+$ [M+H$^+$] 490.0679, found 490.0691.

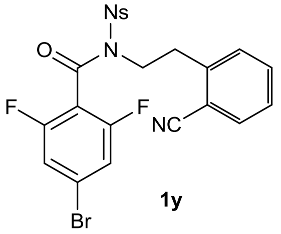

1y

4-bromo-*N*-(2-cyanophenethyl)-2,6-difluoro-*N*-(4-nitrophenylsulfonyl)benzamide: ^{1}H NMR (400 MHz, $CDCl_3$) δ 8.38 (d, *J* = 8.9 Hz, 2H), 8.05 (d, *J* = 8.8 Hz, 2H), 7.67-7.53 (m, 2H), 7.47-7.34 (m, 2H), 7.13 (d, *J* = 6.9 Hz, 2H), 4.15 (t, *J* = 7.3 Hz, 2H), 3.28 (t, *J* = 7.3 Hz, 2H). ^{13}C NMR (101 MHz, $CDCl_3$) δ 160.6, 158.7 (dd, $J_{C\text{-}F}$ = 257, 8.0 Hz), 151.1, 143.6, 140.6, 133.4, 133.2, 131.1, 129.8, 128.0, 124.6, 117.6, 116.2 (dd, $J_{C\text{-}F}$ = 24, 3.5 Hz), 112.8, 47.7, 34.7. HRMS (m/z, ESI-TOF): Calcd for $C_{22}H_{15}BrF_2N_3O_5S^+$ [M+H$^+$] 549.9878, found 549.9877.

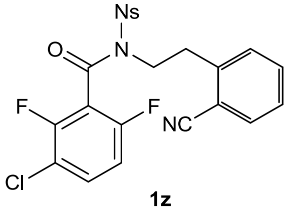

1z

3-chloro-*N*-(2-cyanophenethyl)-2,6-difluoro-*N*-(4-nitrophenylsulfonyl)benzamide: ^{1}H NMR (400 MHz, $CDCl_3$) δ 8.37 (d, *J* = 8.9 Hz, 2H), 8.01 (d, *J* = 8.7 Hz, 2H), 7.66-7.56 (m, 2H), 7.52-7.43 (m, 2H), 7.40 (t, *J* = 7.6 Hz, 1H), 6.96-6.88 (m, 1H), 4.21 (t, *J* = 7.2 Hz, 2H), 3.30 (t, *J* = 7.2 Hz, 2H). ^{13}C NMR (101 MHz, $CDCl_3$) δ 160.4, 157.0 (dd, $J_{C\text{-}F}$ = 254, 5.7 Hz), 154.2 (dd, $J_{C\text{-}F}$ = 255, 7.4 Hz), 151.1, 143.5, 140.5, 133.5, 133.2, 133.1 (d, $J_{C\text{-}F}$ = 9.6 Hz), 131.2, 129.7, 128.1, 124.5, 117.6-117.4 (m), 112.9 (dd, $J_{C\text{-}F}$ = 22, 4.1 Hz), 112.7, 47.8, 34.8. HRMS (m/z, ESI-TOF): Calcd for $C_{22}H_{15}ClF_2N_3O_5S^+$ [M+H$^+$] 506.0384, found 506.0392.

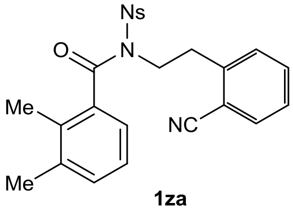

1za

***N*-(2-cyanophenethyl)-2,3-dimethyl-*N*-(4-nitrophenylsulfonyl)benzamide**: ^{1}H NMR (400 MHz, $CDCl_3$) δ 8.34 (d, *J* = 8.9 Hz, 2H), 8.11 (d, *J* = 8.9 Hz, 2H), 7.64-7.49 (m, 2H), 7.37 (t, *J* = 7.6 Hz, 1H), 7.31 (d, *J* = 7.7 Hz, 1H), 7.22 (d, *J* = 7.5 Hz, 1H), 7.03 (t, *J* = 7.6 Hz, 1H), 6.66 (d, *J* = 7.5 Hz, 1H), 4.11 (s, 2H), 3.24 (t, *J* = 7.4 Hz, 2H), 2.22 (s, 3H), 1.91 (s, 3H). ^{13}C NMR (101 MHz, $CDCl_3$) δ 171.3, 150.7, 144.5, 140.9, 138.2, 134.0, 133.2, 133.2, 133.2, 132.1, 130.7, 130.3, 127.8, 125.9, 124.1, 124.0, 117.4, 113.0, 48.2, 35.2, 20.1, 16.3. HRMS (m/z, ESI-TOF): Calcd for $C_{24}H_{22}N_3O_5S^+$ [M+H$^+$] 586.0846, found 586.0852.

General *meta*-C–H olefination procedure and characterization of olefinated products.

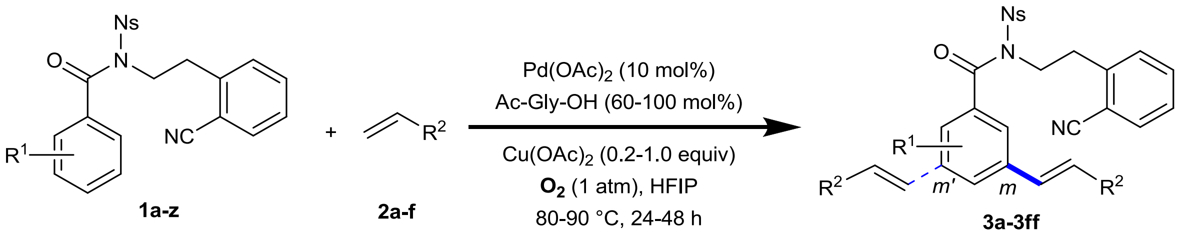

To a 50 mL Schlenk sealed tube (with a Teflon cap) equipped with a magnetic stir bar was charged with amide **1** (0.10 mmol, 1.0 equiv), $Pd(OAc)_2$ (2.3 mg, 0.010 mmol, 10 mol%), Ac-Gly-OH (20-100 mol%), and $Cu(OAc)_2$ (0.2-1.0 equiv) sequentially. HFIP (1.0 mL) was added to the mixture along the inside wall of the tube, followed by the corresponding alkene **2** (2.0 equiv). The reaction tube was capped, then evacuated briefly under vacuum and charged with O_2 (1 atm, balloon, × 3). The tube was then submerged into a preheated 80 or 90 °C oil bath. The reaction was stirred for 24–48 h and cooled to room temperature. The crude reaction mixture was diluted with EtOAc (5 mL) and filtered through a short pad of Celite. The sealed tube and Celite pad were washed with an additional 20 mL of EtOAc. The filtrate was concentrated in *vacuo*, and the resulting residue was purified by flash silica gel chromatography or preparative thin layer chromatography using petroleum ether/EtOAc as the eluent. The site selectivity was assigned by NMR analysis of the product or the hydrolyzed product (see below for hydrolysis).

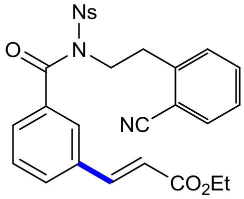

3a$_{mono}$

(*E*)-ethyl 3-(3-((2-cyanophenethyl)(4-nitrophenylsulfonyl)carbamoyl)phenyl)acrylate: Conditions variations: 0.2 mmol scale, Ac-Gly-OH (60 mol%), $Cu(OAc)_2$ (0.5 equiv), 80 °C, 48 h; 51.2 mg, 48%. ^{1}H NMR (400 MHz, $CDCl_3$) δ 8.36 (d, *J* = 8.8 Hz, 2H), 8.14 (d, *J* = 8.8 Hz, 2H), 7.64 (d, *J* = 7.7 Hz, 1H), 7.60-7.48 (m, 3H), 7.41 (t, *J* = 7.7 Hz, 1H), 7.36 (t, *J* = 7.6 Hz, 1H), 7.33-7.28 (m, 2H), 7.22 (s, 1H), 6.33 (d, *J* = 16.0 Hz, 1H), 4.34-4.20 (m, 4H), 3.21 (t, *J* = 6.9 Hz, 2H), 1.35 (t, *J* = 7.1 Hz, 3H). ^{13}C NMR (101 MHz, $CDCl_3$) δ 170.4, 166.4, 150.7, 144.1, 142.5, 140.6, 135.0, 134.4, 133.23, 133.20, 131.5, 131.0, 130.1, 129.3, 129.0, 127.9, 127.1, 124.3, 120.3, 117.3, 112.9, 60.9, 48.3, 34.9, 14.3. HRMS (m/z, ESI-TOF): Calcd for $C_{27}H_{24}N_3O_7S^+$ [M+H$^+$] 534.1329, found 534.1336.

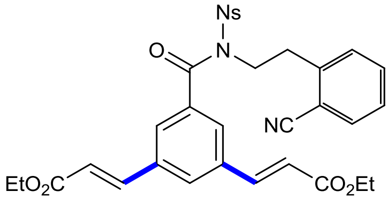

3a$_{(m,m')di}$

(2*E*,2'*E*)-diethyl 3,3'-(5-((2-cyanophenethyl)(4-nitrophenylsulfonyl)carbamoyl)-1,3-phenylene)diacrylate: Conditions variations: 0.2 mmol scale, Ac-Gly-OH (60 mol%), $Cu(OAc)_2$ (0.5 equiv), 80 °C, 48 h; 54.2 mg, 44%. ^{1}H NMR (400 MHz, $CDCl_3$) δ 8.37 (d, *J* = 8.7 Hz, 2H), 8.11 (d, *J* = 8.7 Hz, 2H), 7.73 (s, 1H), 7.65-7.48 (m, 4H), 7.42-7.32 (m, 2H), 7.28 (s, 2H), 6.39 (d, *J* = 16.0 Hz, 2H), 4.40-4.19 (m, 6H), 3.23 (t, *J* = 6.7 Hz, 2H), 1.36 (t, *J* = 7.1 Hz, 6H). ^{13}C NMR (101 MHz, $CDCl_3$) δ 169.8, 166.2, 150.8, 144.0,

141.8, 140.7, 135.7, 135.4, 133.3, 133.2, 131.1, 130.6, 130.0, 128.0, 127.9, 124.5, 121.2, 117.3, 113.0, 61.0, 48.2, 34.8, 14.4. HRMS (m/z, ESI-TOF): Calcd for $C_{32}H_{30}N_3O_9S^+$ [M+H$^+$] 632.1697, found 632.1703.

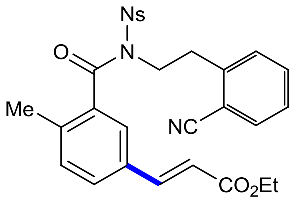

3b

(*E*)-ethyl 3-(3-((2-cyanophenethyl)(4-nitrophenylsulfonyl)carbamoyl)-4-methylphenyl)acrylate: Conditions variations: Formyl-Gly-OH (60 mol%), $Cu(OAc)_2$ (0.2 equiv), KH_2PO_4 (0.5 equiv), 80 °C, 24 h; 38 mg, 69%. ^{1}H NMR (400 MHz, $CDCl_3$) δ 8.32 (d, *J* = 8.8 Hz, 2H), 8.02 (d, *J* = 8.8 Hz, 2H), 7.62-7.55 (m, 2H), 7.51-7.34 (m, 4H), 7.20 (d, *J* = 7.9 Hz, 1H), 6.70 (s, 1H), 6.16 (d, *J* = 16.0 Hz, 1H), 4.33-4.13 (m, 4H), 3.29 (t, *J* = 7.1 Hz, 2H), 2.13 (s, 3H), 1.34 (t, *J* = 7.1 Hz, 3H). ^{13}C NMR (101 MHz, $CDCl_3$) δ 170.1, 166.5, 150.8, 144.4, 142.5, 140.8, 137.5, 134.6, 133.3, 132.2, 131.5, 130.9, 130.2, 130.0, 128.0, 125.9, 124.2, 119.2, 117.4, 113.1, 60.9, 48.0, 35.1, 19.1, 14.4. HRMS (m/z, ESI-TOF): Calcd for $C_{28}H_{26}N_3O_7S^+$ [M+H$^+$] 548.1486, found 548.1490.

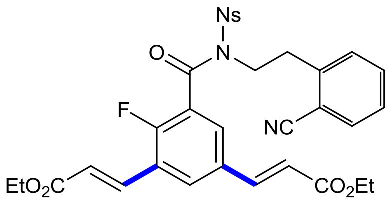

3c

(*E*)-ethyl 3-(3-((2-cyanophenethyl)(4-nitrophenylsulfonyl)carbamoyl)-4-fluorophenyl)acrylate: Conditions variations: 0.2 mmol scale, Ac-Gly-OH (60 mol%), $Cu(OAc)_2$ (0.5 equiv), 80 °C, 48 h; 113 mg, 87%. ^{1}H NMR (400 MHz, $CDCl_3$) δ 8.35 (d, *J* = 8.7 Hz, 2H), 8.07 (d, *J* = 8.7 Hz, 2H), 7.73 (d, *J* = 5.4 Hz, 1H), 7.66-7.59 (m, 2H), 7.54-7.44 (m, 3H), 7.40 (t, *J* = 7.6 Hz, 1H), 6.88 (d, *J* = 4.2 Hz, 1H), 6.50 (d, *J* = 16.2 Hz, 1H), 6.28 (d, *J* = 16.0 Hz, 1H), 4.34-4.16 (m, 6H), 3.28 (t, *J* = 6.6 Hz, 2H), 1.39-1.28 (m, 6H). ^{13}C NMR (101 MHz, $CDCl_3$) δ 166.1, 166.0, 165.1, 156.9 (d, J_{C-F} = 261 Hz), 150.9, 143.7, 140.9, 140.6, 134.6 (d, J_{C-F} = 2.2 Hz), 133.4, 133.2, 131.7 (d, J_{C-F} = 3.8 Hz), 131.5, 131.1 (d, J_{C-F} = 3.8 Hz), 130.0, 129.0 (d, J_{C-F} = 3.2 Hz), 128.0, 124.4, 124.0 (d, J_{C-F} = 31 Hz), 124.0, 123.7 (d, J_{C-F} = 6.2 Hz), 120.9 (d, J_{C-F} = 0.9 Hz), 117.3, 113.0, 61.1, 61.1, 48.0, 35.2, 14.4, 14.3. HRMS (m/z, ESI-TOF): Calcd for $C_{32}H_{29}FN_3O_9S^+$ [M+H$^+$] 650.1603, found 650.1613.

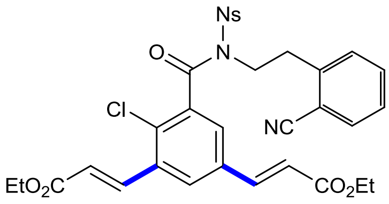

3d

(2*E*,2'*E*)-diethyl 3,3'-(4-chloro-5-((2-cyanophenethyl)(4-nitrophenylsulfonyl)carbamoyl)-1,3-phenylene)diacrylate: Conditions variations: Ac-Gly-OH (60 mol%), $Cu(OAc)_2$ (0.5 equiv), 80 °C, 48 h; 48 mg, 72%. ^{1}H NMR (400 MHz, $CDCl_3$) δ 8.36 (d, *J* = 8.7 Hz, 2H), 8.12 (d, *J* = 8.7 Hz, 2H), 7.86 (d, *J* = 16.0 Hz, 1H), 7.75 (d, *J* = 1.5 Hz, 1H), 7.64 (td, *J* = 7.7, 1.1 Hz, 1H), 7.56-7.45 (m, 3H), 7.42 (td, *J* = 7.6, 0.8 Hz, 1H), 6.82 (d, *J* = 1.1

Hz, 1H), 6.45 (d, *J* = 16.0 Hz, 1H), 6.34 (d, *J* = 16.0 Hz, 1H), 4.33-4.25 (m, 4H), 4.24-3.95 (m, 2H), 3.30 (t, *J* = 6.7 Hz, 2H), 1.38-1.31 (m, 6H). ^{13}C NMR (101 MHz, $CDCl_3$) δ 166.6, 166.1, 165.7, 151.0, 143.5, 141.0, 140.6, 138.2, 135.4, 134.7, 134.2, 133.4, 133.3, 131.4, 131.3, 130.4, 128.6, 128.1, 127.9, 124.2, 123.9, 121.6, 117.3, 113.1, 61.2, 61.1, 48.2, 35.2, 14.4, 14.4. HRMS (m/z, ESI-TOF): Calcd for $C_{32}H_{29}ClN_3O_9S^+$ [M+H$^+$] 666.1308, found 666.1320.

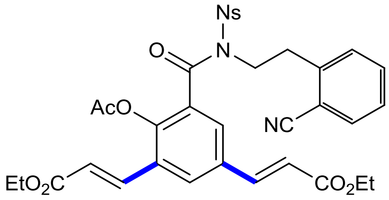

3e

(2*E*,2'*E*)-diethyl 3,3'-(4-acetoxy-5-((2-cyanophenethyl)(4-nitrophenylsulfonyl)carbamoyl)-1,3-phenylene)diacrylate: Conditions variations: Formyl-Gly-OH (60 mol%), $Cu(OAc)_2$ (0.2 equiv), KH_2PO_4 (0.5 equiv), 80 °C, 24 h; 53 mg, 77%. ^{1}H NMR (400 MHz, $CDCl_3$) δ 8.38 (d, *J* = 8.9 Hz, 2H), 8.22 (d, *J* = 8.9 Hz, 2H), 7.84 (d, *J* = 1.4 Hz, 1H), 7.62-7.50 (m, 4H), 7.36 (t, *J* = 7.4 Hz, 1H), 7.28 (d, *J* = 7.8 Hz, 1H), 7.23 (d, *J* = 1.5 Hz, 1H), 6.47 (d, *J* = 16.1 Hz, 1H), 6.36 (d, *J* = 16.0 Hz, 1H), 4.33-4.21 (m, 4H), 4.09 (t, *J* = 6.8 Hz, 2H), 3.26 (t, *J* = 7.1 Hz, 2H), 2.17 (s, 3H), 1.37-1.30 (m, 6H). ^{13}C NMR (101 MHz, $CDCl_3$) δ 168.2, 166.9, 166.2, 166.0, 150.9, 146.7, 144.0, 141.3, 140.6, 136.0, 133.4, 133.3, 133.1, 130.7, 130.5, 130.0, 129.6, 129.3, 128.2, 128.0, 124.2, 123.1, 121.2, 117.4, 112.9, 61.1, 61.1, 48.4, 35.0, 20.6, 14.4, 14.3. HRMS (m/z, ESI-TOF): Calcd for $C_{34}H_{32}N_3O_{11}S^+$ [M+H$^+$] 690.1752, found 690.1741.

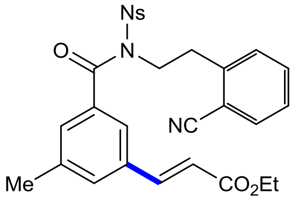

3f

(*E*)-ethyl 3-(3-((2-cyanophenethyl)(4-nitrophenylsulfonyl)carbamoyl)-5-methylphenyl)acrylate: Conditions variations: Ac-Gly-OH (60 mol%), $Cu(OAc)_2$ (1.0 equiv), 80 °C, 48 h; 45 mg, 82%. ^{1}H NMR (400 MHz, $CDCl_3$) δ 8.37 (d, *J* = 8.3 Hz, 2H), 8.16 (d, *J* = 8.0 Hz, 2H), 7.58-7.47 (m, 3H), 7.44 (s, 1H), 7.36 (t, *J* = 7.6 Hz, 1H), 7.31 (d, *J* = 7.6 Hz, 1H), 7.02 (s, 2H), 6.31 (d, *J* = 16.0 Hz, 1H), 4.37-4.20 (m, 4H), 3.21 (t, *J* = 6.4 Hz, 2H), 2.36 (s, 3H), 1.35 (t, *J* = 7.0 Hz, 3H). ^{13}C NMR (101 MHz, $CDCl_3$) δ 170.6, 166.5, 150.8, 144.3, 142.8, 140.7, 139.5, 135.0, 134.4, 133.3, 133.2, 132.3, 131.1, 130.2, 129.6, 127.9, 124.3, 124.3, 120.1, 117.3, 113.0, 60.9, 48.3, 35.0, 21.3, 14.4. HRMS (m/z, ESI-TOF): Calcd for $C_{28}H_{26}N_3O_7S^+$ [M+H$^+$] 548.1486, found 548.1496.

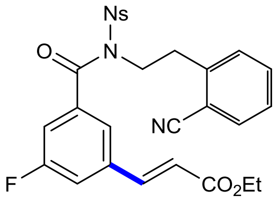

3g

(*E*)-ethyl 3-(3-((2-cyanophenethyl)(4-nitrophenylsulfonyl)carbamoyl)-5-fluorophenyl)acrylate: Conditions variations: Formyl-Gly-OH (60 mol%), $Cu(OAc)_2$ (0.5 equiv), 90 °C, 48 h; 28 mg, 51%. ^{1}H NMR (400 MHz, $CDCl_3$) δ 8.38 (d, *J* = 8.8 Hz, 2H), 8.14 (d, *J* = 8.8 Hz, 2H), 7.60-7.48 (m, 3H), 7.42-7.30 (m, 3H), 7.04 (s, 1H), 6.89 (d, *J* = 7.9 Hz, 1H),

6.34 (d, J = 16.0 Hz, 1H), 4.33-4.20 (m, 4H), 3.22 (t, J = 6.8 Hz, 2H), 1.35 (t, J = 7.1 Hz, 3H). ^{13}C NMR (101 MHz, $CDCl_3$) δ 169.1 (d, J_{C-F} = 2.4 Hz), 166.1, 162.5 (d, J_{C-F} = 252 Hz), 151.0, 143.9, 141.3 (d, J_{C-F} = 1.9 Hz), 140.6, 137.5 (d, J_{C-F} = 7.8 Hz), 136.3 (d, J_{C-F} = 7.4 Hz), 133.4, 133.3, 131.1, 130.2, 128.1, 124.5, 122.9 (d, J_{C-F} = 2.9 Hz), 121.8, 118.0 (d, J_{C-F} = 22 Hz), 117.3, 116.2 (d, J_{C-F} = 24 Hz), 113.0, 61.1, 48.3, 34.9, 14.4. HRMS (m/z, ESI-TOF): Calcd for $C_{27}H_{23}FN_3O_7S^+$ [M+H$^+$] 552.1235, found 552.1240.

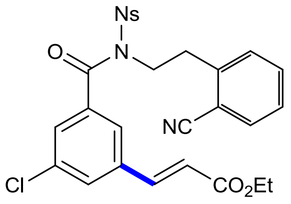

3h **(E)-ethyl 3-(3-chloro-5-((2-cyanophenethyl)(4-nitrophenylsulfonyl)carbamoyl)phenyl)acrylate**: Conditions variations: Ac-Gly-OH (60 mol%), $Cu(OAc)_2$ (0.2 equiv), 90 °C, 48 h; 41 mg, 72%. ^{1}H NMR (400 MHz, $CDCl_3$) δ 8.38 (d, J = 8.3 Hz, 2H), 8.12 (d, J = 8.2 Hz, 2H), 7.68-7.45 (m, 4H), 7.40 (t, J = 7.6 Hz, 1H), 7.34 (d, J = 7.7 Hz, 1H), 7.15 (s, 1H), 7.05 (s, 1H), 6.36 (d, J = 16.0 Hz, 1H), 4.38-4.17 (m, 4H), 3.23 (t, J = 6.8 Hz, 2H), 1.35 (t, J = 7.1 Hz, 3H). ^{13}C NMR (101 MHz, $CDCl_3$) δ 169.0, 166.0, 150.9, 143.9, 141.1, 140.6, 136.8, 136.0, 135.4, 133.3, 133.3, 131.2, 131.1, 130.1, 128.7, 128.1, 125.1, 124.5, 121.8, 117.3, 113.0, 61.1, 48.2, 34.8, 14.4. HRMS (m/z, ESI-TOF): Calcd for $C_{27}H_{23}ClN_3O_7S^+$ [M+H$^+$] 568.0940, found 568.0944.

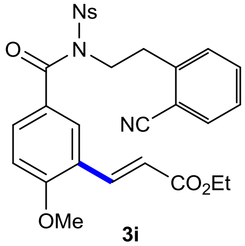

3i **(E)-ethyl 3-(5-((2-cyanophenethyl)(4-nitrophenylsulfonyl)carbamoyl)-2-methoxyphenyl)acrylate**: Conditions variations: Ac-Gly-OH (100 mol%), $Cu(OAc)_2$ (1.0 equiv), 90 °C, 48 h; 45 mg, 80%. ^{1}H NMR (400 MHz, $CDCl_3$) δ 8.36 (d, J = 8.9 Hz, 2H), 8.11 (d, J = 8.9 Hz, 2H), 7.85 (d, J = 16.2 Hz, 1H), 7.57-7.47 (m, 3H), 7.42 (d, J = 1.9 Hz, 1H), 7.35 (t, J = 7.2 Hz, 1H), 7.27 (t, J = 3.7 Hz, 2H), 6.93 (d, J = 8.7 Hz, 1H), 6.37 (d, J = 16.2 Hz, 1H), 4.27 (q, J = 7.1 Hz, 2H), 4.20 (t, J = 7.0 Hz, 2H), 3.96 (s, 3H), 3.18 (t, J = 7.0 Hz, 2H), 1.35 (t, J = 7.1 Hz, 3H). ^{13}C NMR (101 MHz, $CDCl_3$) δ 170.3, 170.0, 161.6, 150.7, 144.2, 140.9, 138.1, 133.2, 133.2, 132.2, 130.9, 130.0, 129.7, 127.9, 126.1, 124.4, 123.7, 120.6, 117.4, 112.9, 111.1, 60.8, 56.1, 48.3, 34.6, 14.4. HRMS (m/z, ESI-TOF): Calcd for $C_{28}H_{26}N_3O_8S^+$ [M+H$^+$] 564.1435, found 564.1445.

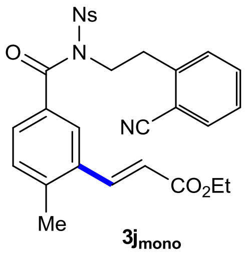

$3j_{mono}$ **(E)-ethyl 3-(5-((2-cyanophenethyl)(4-nitrophenylsulfonyl)carbamoyl)-2-methylphenyl)acrylate**: Conditions variations A: Formyl-Gly-OH (60 mol%), $Cu(OAc)_2$ (0.2 equiv), K_2HPO_4 (0.5 equiv), 80 °C, 24 h; 33 mg, 60%. Conditions variations B: Formyl-Gly-OH

(60 mol%), $Cu(OAc)_2$ (0.2 equiv), KH_2PO_4 (0.5 equiv), 80 °C, 24 h; 32 mg, 58%. ^1H NMR (400 MHz, $CDCl_3$) δ 8.35 (d, J = 9.0 Hz, 2H), 8.12 (d, J = 8.9 Hz, 2H), 7.85 (d, J = 15.9 Hz, 1H), 7.57-7.49 (m, 2H), 7.38-7.31 (m, 2H), 7.31-7.21 (m, 4H), 6.19 (d, J = 15.9 Hz, 1H), 4.33-4.20 (m, 4H), 3.19 (t, J = 7.0 Hz, 2H), 2.47 (s, 3H), 1.35 (t, J = 7.1 Hz, 3H). ^{13}C NMR (101 MHz, $CDCl_3$) δ 170.6, 166.5, 150.8, 144.3, 142.5, 140.8, 140.5, 133.9, 133.2, 132.0, 131.1, 131.0, 130.1, 129.0, 127.9, 126.4, 124.4, 121.3, 117.4, 112.9, 60.9, 48.3, 34.8, 20.1, 14.4. HRMS (m/z, ESI-TOF): Calcd for $C_{28}H_{26}N_3O_7S^+$ $[M+H^+]$ 548.1486, found 548.1490.

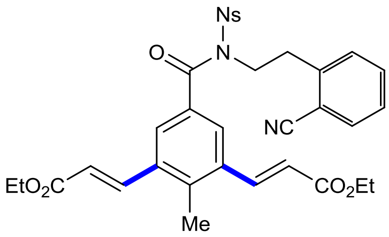

3j$_{(m,m')di}$ **(2*E*,2'*E*)-diethyl 3,3'-(5-((2-cyanophenethyl)(4-nitrophenylsulfonyl)carbamoyl)-2-methyl-1,3-phenylene)diacrylate**: Conditions variations A: Formyl-Gly-OH (60 mol%), $Cu(OAc)_2$ (0.2 equiv), K_2HPO_4 (0.5 equiv), 80 °C, 24 h; 6 mg, 9%. Conditions variations B: Formyl-Gly-OH (60 mol%), $Cu(OAc)_2$ (0.2 equiv), KH_2PO_4 (0.5 equiv), 80 °C, 24 h; 15 mg, 23%. ^1H NMR (400 MHz, $CDCl_3$) δ 8.36 (d, J = 8.9 Hz, 2H), 8.08 (d, J = 8.9 Hz, 2H), 7.94 (d, J = 15.8 Hz, 2H), 7.53 (d, J = 7.5 Hz, 2H), 7.37 (s, 2H), 7.36-7.28 (m, 2H), 6.20 (d, J = 15.8 Hz, 2H), 4.34-4.22 (m, 6H), 3.21 (t, J = 6.9 Hz, 2H), 2.49 (s, 3H), 1.36 (t, J = 7.1 Hz, 6H). ^{13}C NMR (101 MHz, $CDCl_3$) δ 170.1, 166.3, 150.8, 144.1, 141.0, 140.9, 140.8, 135.5, 133.3, 132.3, 131.1, 130.0, 128.0, 127.5, 124.5, 122.7, 117.4, 113.0, 61.1, 48.1, 34.6, 16.3, 14.4. HRMS (m/z, ESI-TOF): Calcd for $C_{33}H_{32}N_3O_9S^+$ $[M+H^+]$ 646.1854, found 646.1857.

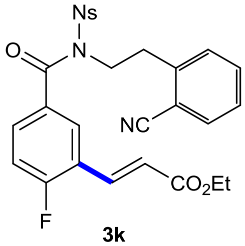

3k **(*E*)-ethyl 3-(5-((2-cyanophenethyl)(4-nitrophenylsulfonyl)carbamoyl)-2-fluorophenyl)acrylate**: Conditions variations A: Formyl-Gly-OH (60 mol%), $Cu(OAc)_2$ (0.2 equiv), K_2HPO_4 (0.5 equiv), 80 °C, 24 h; 32 mg, 58%. Conditions variations B: Formyl-Gly-OH (60 mol%), $Cu(OAc)_2$ (0.2 equiv), KH_2PO_4 (0.5 equiv), 80 °C, 24 h; 33 mg, 60%. ^1H NMR (400 MHz, $CDCl_3$) δ 8.37 (d, J = 8.8 Hz, 2H), 8.12 (d, J = 8.8 Hz, 2H), 7.69 (d, J = 16.2 Hz, 1H), 7.59-7.50 (m, 2H), 7.43-7.33 (m, 3H), 7.31 (d, J = 7.6 Hz, 1H), 7.14 (t, J = 9.5 Hz, 1H), 6.41 (d, J = 16.2 Hz, 1H), 4.28 (q, J = 7.1 Hz, 2H), 4.21 (t, J = 6.9 Hz, 2H), 3.20 (t, J = 6.9 Hz, 2H), 1.35 (t, J = 7.1 Hz, 3H). ^{13}C NMR (101 MHz, $CDCl_3$) δ 169.6, 166.3, 163.3 (d, $J_{C\text{-}F}$ = 263 Hz), 150.9, 143.9, 140.7, 135.3 (d, $J_{C\text{-}F}$ = 2.4 Hz), 133.3 (d, $J_{C\text{-}F}$ = 4.2 Hz), 131.5 (d, $J_{C\text{-}F}$ = 9.9 Hz), 131.0, 130.5 (d, $J_{C\text{-}F}$ = 3.6 Hz), 130.1, 129.6 (d, $J_{C\text{-}F}$ = 4.1 Hz), 128.0, 124.5, 123.2 (d, $J_{C\text{-}F}$ = 12.9 Hz), 122.8 (d, $J_{C\text{-}F}$ = 6.0 Hz), 117.4, 117.0, 116.8, 112.9, 61.1, 48.2, 34.7, 14.4. HRMS (m/z, ESI-TOF): Calcd for $C_{27}H_{23}FN_3O_7S^+$ $[M+H^+]$ 552.1235, found 552.1243.

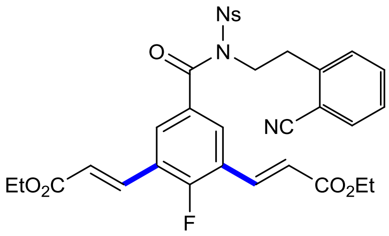

3k$_{(m,m')di}$ **(2*E*,2'*E*)-diethyl 3,3'-(5-((2-cyanophenethyl)(4-nitrophenylsulfonyl)carbamoyl)-4,6-difluoro-1,3-phenylene)diacrylate**: Conditions variations B: Formyl-Gly-OH (60 mol%), $Cu(OAc)_2$ (0.2 equiv), KH_2PO_4 (0.5 equiv), 80 °C, 24 h; 22 mg, 34%. ^1H NMR (400 MHz, $CDCl_3$) δ 8.38 (d, *J* = 8.2 Hz, 2H), 8.08 (d, *J* = 8.3 Hz, 2H), 7.74 (d, *J* = 16.1 Hz, 2H), 7.56 (t, *J* = 7.9 Hz, 2H), 7.44 (d, *J* = 5.7 Hz, 2H), 7.41-7.31 (m, 2H), 6.44 (d, *J* = 16.2 Hz, 2H), 4.30 (q, *J* = 6.6 Hz, 4H), 4.22 (t, *J* = 6.4 Hz, 2H), 3.21 (t, *J* = 6.3 Hz, 2H), 1.36 (t, *J* = 6.9 Hz, 6H). ^{13}C NMR (101 MHz, $CDCl_3$) δ 169.3, 166.1, 161.1 (J_{C-F} = 269 Hz), 150.9, 143.8, 140.7, 134.8 (J_{C-F} = 3.8 Hz), 133.4, 133.3, 131.1, 130.9 (J_{C-F} = 3.9 Hz), 129.9, 129.8 (J_{C-F} = 4.3 Hz), 128.1, 124.6, 124.0 (J_{C-F} = 13 Hz), 123.5 (J_{C-F} = 5.8 Hz), 117.4, 113.0, 61.2, 48.1, 34.5, 14.4. HRMS (m/z, ESI-TOF): Calcd for $C_{32}H_{29}FN_3O_9S^+$ [$M+H^+$] 650.1603, found 650.1612.

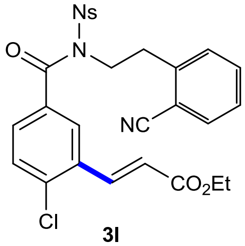

3l **(*E*)-ethyl 3-(2-chloro-5-((2-cyanophenethyl)(4-nitrophenylsulfonyl)carbamoyl)phenyl)acrylate**: Conditions variations A: Formyl-Gly-OH (60 mol%), $Cu(OAc)_2$ (0.2 equiv), K_2HPO_4 (0.5 equiv), 80 °C, 24 h; 35 mg, 62%. Conditions variations B: Formyl-Gly-OH (60 mol%), $Cu(OAc)_2$ (0.2 equiv), KH_2PO_4 (0.5 equiv), 80 °C, 24 h; 34 mg, 60%. ^1H NMR (400 MHz, $CDCl_3$) δ 8.36 (d, *J* = 8.7 Hz, 2H), 8.09 (d, *J* = 8.7 Hz, 2H), 7.97 (d, *J* = 16.0 Hz, 1H), 7.58-7.51 (m, 2H), 7.45 (d, *J* = 8.3 Hz, 1H), 7.41 (d, *J* = 1.7 Hz, 1H), 7.36 (t, *J* = 7.6 Hz, 1H), 7.30 (d, *J* = 8.0 Hz, 2H), 6.26 (d, *J* = 16.0 Hz, 1H), 4.28 (q, *J* = 7.1 Hz, 2H), 4.21 (t, *J* = 7.0 Hz, 2H), 3.21 (t, *J* = 7.0 Hz, 2H), 1.35 (t, *J* = 7.1 Hz, 3H). ^{13}C NMR (101 MHz, $CDCl_3$) δ 169.6, 166.0, 150.8, 143.9, 140.7, 139.0, 138.7, 133.3, 133.2, 132.9, 130.9, 130.6, 130.0, 129.9, 128.0, 127.5, 124.5, 122.7, 117.4, 112.9, 61.1, 48.1, 34.6, 14.4. HRMS (m/z, ESI-TOF): Calcd for $C_{27}H_{23}ClN_3O_7S^+$ [$M+H^+$] 568.0940, found 568.0947.

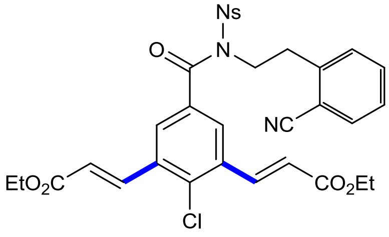

3l$_{(m,m')di}$ **(2*E*,2'*E*)-diethyl 3,3'-(2-chloro-5-((2-cyanophenethyl)(4-nitrophenylsulfonyl)carbamoyl)-1,3-phenylene)diacrylate**: Conditions variations B: Formyl-Gly-OH (60 mol%), $Cu(OAc)_2$ (0.2 equiv), KH_2PO_4 (0.5 equiv), 80 °C, 24 h; 19 mg, 29%. ^1H NMR (400 MHz, $CDCl_3$) δ 8.38 (d, *J* = 8.5 Hz, 2H), 8.12-8.00 (m, 4H), 7.59-7.52 (m, 2H), 7.47 (s, 2H), 7.41-7.30 (m, 2H), 6.28 (d, *J* = 15.9 Hz, 2H), 4.30 (q, *J* = 6.8 Hz, 4H), 4.24 (t, *J* = 6.5 Hz, 2H), 3.22 (t, *J* = 6.4 Hz, 2H), 1.37 (t, *J* = 7.0 Hz, 6H). ^{13}C NMR (101 MHz, $CDCl_3$) δ 169.3,

165.9, 150.9, 143.9, 140.7, 139.0, 138.7, 134.7, 133.3, 133.1, 131.0, 129.9, 128.1, 128.0, 124.7, 123.6, 117.5, 113.0, 61.3, 48.0, 34.4, 14.4. HRMS (m/z, ESI-TOF): Calcd for $C_{32}H_{29}ClN_3O_9S^+$ [M+H$^+$] 666.1308, found 666.1316.

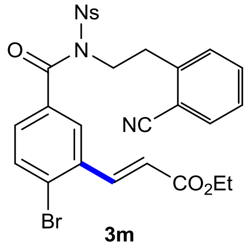

3m **(*E*)-ethyl 3-(2-bromo-5-((2-cyanophenethyl)(4-nitrophenylsulfonyl)carbamoyl)phenyl)acrylate**: Conditions variations: Formyl-Gly-OH (60 mol%), $Cu(OAc)_2$ (0.2 equiv), KH_2PO_4 (0.5 equiv), 80 °C, 24 h; 44 mg, 72%. ^{1}H NMR (400 MHz, $CDCl_3$) δ 8.37 (d, *J* = 8.7 Hz, 2H), 8.09 (d, *J* = 8.7 Hz, 2H), 7.95 (d, *J* = 16.0 Hz, 1H), 7.66 (d, *J* = 8.3 Hz, 1H), 7.55 (t, *J* = 7.1 Hz, 2H), 7.41-7.34 (m, 2H), 7.31 (d, *J* = 7.8 Hz, 1H), 7.21 (dd, *J* = 8.2, 1.7 Hz, 1H), 6.22 (d, *J* = 16.0 Hz, 1H), 4.29 (q, *J* = 7.1 Hz, 2H), 4.21 (t, *J* = 7.0 Hz, 2H), 3.22 (t, *J* = 7.0 Hz, 2H), 1.36 (t, *J* = 7.1 Hz, 3H). ^{13}C NMR (101 MHz, $CDCl_3$) δ 169.7, 165.9, 150.9, 144.0, 141.3, 140.7, 135.2, 133.9, 133.6, 133.3, 131.0, 130.0, 129.9, 129.6, 128.1, 127.5, 124.5, 122.9, 117.4, 113.0, 61.2, 48.1, 34.6, 14.4. HRMS (m/z, ESI-TOF): Calcd for $C_{27}H_{23}BrN_3O_7S^+$ [M+H$^+$] 612.0435, found 612.0444.

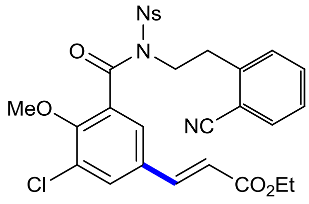

3n **(*E*)-ethyl 3-(3-chloro-5-((2-cyanophenethyl)(4-nitrophenylsulfonyl)carbamoyl)-4-methoxyphenyl)acrylate**: Conditions variations: 0.2 mmol scale, Ac-Gly-OH (60 mol%), $Cu(OAc)_2$ (0.5 equiv), 90 °C, 48 h; 105 mg, 88%. ^{1}H NMR (400 MHz, $CDCl_3$) δ 8.36 (d, *J* = 8.8 Hz, 2H), 8.09 (d, *J* = 8.7 Hz, 2H), 7.64-7.57 (m, 2H), 7.54 (d, *J* = 7.5 Hz, 1H), 7.46-7.36 (m, 3H), 6.78 (d, *J* = 1.5 Hz, 1H), 6.27 (d, *J* = 16.0 Hz, 1H), 4.27 (q, *J* = 7.1 Hz, 2H), 4.22-4.10 (m, 2H), 3.58 (s, 3H), 3.25 (t, *J* = 6.9 Hz, 2H), 1.34 (t, *J* = 7.1 Hz, 3H). ^{13}C NMR (101 MHz, $CDCl_3$) δ 167.1, 166.3, 153.1, 150.8, 144.0, 141.0, 140.8, 133.3, 133.3, 132.1, 132.0, 131.1, 130.9, 130.2, 128.7, 127.9, 125.8, 124.1, 120.6, 117.4, 113.1, 62.4, 61.0, 48.1, 35.2, 14.4. HRMS (m/z, ESI-TOF): Calcd for $C_{28}H_{25}ClN_3O_8S^+$ [M+H$^+$] 598.1045, found 598.1053.

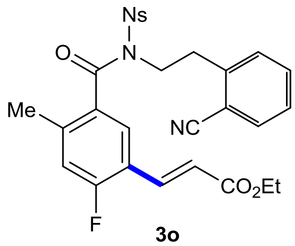

3o **(*E*)-ethyl 3-(5-((2-cyanophenethyl)(4-nitrophenylsulfonyl)carbamoyl)-2-fluoro-4-methylphenyl)acrylate**: Conditions variations: Formyl-Gly-OH (60 mol%), $Cu(OAc)_2$ (0.2 equiv), KH_2PO_4 (0.5 equiv), 80 °C, 24 h; 44 mg, 78%. ^{1}H NMR (400 MHz, $CDCl_3$) δ 8.34 (d, *J* = 8.9 Hz, 2H), 8.01 (d, *J* = 8.9 Hz, 2H), 7.63-7.52 (m, 3H), 7.45-7.37 (m, 2H), 6.95 (d, *J* = 11.0 Hz, 1H), 6.75 (d, *J* = 7.1 Hz, 1H), 6.19 (d, *J* = 16.2

Hz, 1H), 4.32-4.15 (m, 4H), 3.28 (t, J = 7.1 Hz, 2H), 2.15 (s, 3H), 1.34 (t, J = 7.1 Hz, 3H). ^{13}C NMR (101 MHz, $CDCl_3$) δ 169.4, 166.4, 161.9 (d, $J_{C\text{-}F}$ = 260 Hz), 150.8, 144.4, 140.8, 140.6 (d, $J_{C\text{-}F}$ = 9.5 Hz), 135.4 (d, $J_{C\text{-}F}$ = 1.8 Hz), 133.4, 133.3, 131.0, 130.7 (d, $J_{C\text{-}F}$ = 3.5 Hz), 130.0, 128.1, 127.6 (d, $J_{C\text{-}F}$ = 4.2 Hz), 124.4, 121.6 (d, $J_{C\text{-}F}$ = 6.3 Hz), 120.2 (d, $J_{C\text{-}F}$ = 12.9 Hz), 118.8 (d, $J_{C\text{-}F}$ = 22.7 Hz), 117.5, 113.1, 61.0, 48.0, 35.0, 19.4, 14.4. HRMS (m/z, ESI-TOF): Calcd for $C_{28}H_{24}FN_3O_7S^+$ [M+H$^+$] 566.1392, found 566.1393.

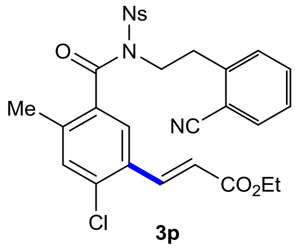

3p **(*E*)-ethyl 3-(2-chloro-5-((2-cyanophenethyl)(4-nitrophenylsulfonyl)carbamoyl)-4-methylphenyl)acrylate**: Conditions variations: Formyl-Gly-OH (60 mol%), $Cu(OAc)_2$ (0.2 equiv), K_2HPO_4 (0.5 equiv), 80 °C, 24 h; 43 mg, 74%. ^{1}H NMR (400 MHz, $CDCl_3$) δ 8.31 (d, J = 8.9 Hz, 2H), 8.02-7.85 (m, 3H), 7.64-7.55 (m, 2H), 7.43-7.36 (m, 2H), 7.26 (s, 1H), 6.88 (s, 1H), 5.96 (d, J = 16.0 Hz, 1H), 4.25 (dd, J = 14.3, 7.1 Hz, 4H), 3.27 (t, J = 7.2 Hz, 2H), 2.15 (s, 3H), 1.33 (t, J = 7.1 Hz, 3H). ^{13}C NMR (101 MHz, $CDCl_3$) δ 169.2, 166.0, 150.8, 144.4, 140.9, 139.2, 138.6, 137.2, 133.4, 133.3, 132.5, 130.9, 130.1, 129.8, 128.1, 125.7, 124.4, 121.5, 117.5, 113.1, 61.1, 47.9, 34.8, 19.1, 14.4. HRMS (m/z, ESI-TOF): Calcd for $C_{28}H_{25}ClN_3O_7S^+$ [M+H$^+$] 582.1096, found 582.1101.

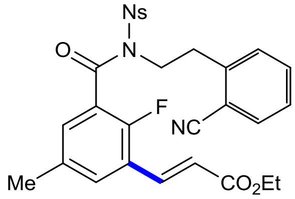

3q **(*E*)-ethyl 3-(3-((2-cyanophenethyl)(4-nitrophenylsulfonyl)carbamoyl)-2-fluoro-5-methylphenyl)acrylate**: Conditions variations: Ac-Gly-OH (100 mol%), $Cu(OAc)_2$ (1.0 equiv), 90 °C, 48 h; 52 mg, 92%. ^{1}H NMR (400 MHz, $CDCl_3$) δ 8.35 (d, J = 8.8 Hz, 2H), 8.10 (d, J = 8.8 Hz, 2H), 7.63-7.56 (m, 2H), 7.53 (d, J = 7.5 Hz, 1H), 7.47 (d, J = 7.7 Hz, 1H), 7.43-7.36 (m, 2H), 6.57 (d, J = 4.5 Hz, 1H), 6.43 (d, J = 16.2 Hz, 1H), 4.31-4.15 (m, 4H), 3.26 (t, J = 6.7 Hz, 2H), 2.26 (s, 3H), 1.33 (t, J = 7.1 Hz, 3H). ^{13}C NMR (101 MHz, $CDCl_3$) δ 166.3, 166.0, 154.6 (d, $J_{C\text{-}F}$ = 255 Hz), 150.9, 143.9, 140.7, 135.5 (d, $J_{C\text{-}F}$ = 2.7 Hz), 134.9 (d, $J_{C\text{-}F}$ = 3.9 Hz), 133.4, 133.0, 132.3 (d, $J_{C\text{-}F}$ = 2.9 Hz), 131.5, 130.5 (d, $J_{C\text{-}F}$ = 2.6 Hz), 130.1, 127.8, 124.2, 122.8 (d, $J_{C\text{-}F}$ = 1.9 Hz), 122.7 (d, $J_{C\text{-}F}$ = 7.1 Hz), 122.6 (d, $J_{C\text{-}F}$ = 6.3 Hz), 117.2, 113.0, 61.0, 48.1, 35.3, 20.6, 14.4. HRMS (m/z, ESI-TOF): Calcd for $C_{28}H_{25}FN_3O_7S^+$ [M+H$^+$] 566.1392, found 566.1394.

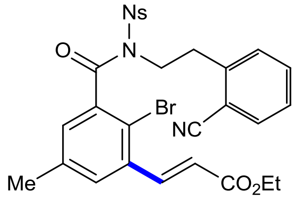

3r **(*E*)-ethyl 3-(2-bromo-3-((2-cyanophenethyl)(4-nitrophenylsulfonyl)carbamoyl)-5-methylphenyl)acrylate**: Conditions variations: Ac-Gly-OH (100 mol%), $Cu(OAc)_2$ (1.0 equiv), 90 °C, 48 h; 58 mg, 93%. ^{1}H NMR (400 MHz, $CDCl_3$) δ

8.36 (d, J = 8.8 Hz, 2H), 8.19 (d, J = 8.8 Hz, 2H), 7.81 (d, J = 15.9 Hz, 1H), 7.65-7.54 (m, 2H), 7.48 (d, J = 7.6 Hz, 1H), 7.44 (s, 1H), 7.40 (t, J = 7.6 Hz, 1H), 6.61 (s, 1H), 6.35 (d, J = 15.9 Hz, 1H), 4.37-3.84 (m, 4H), 3.29 (t, J = 6.4 Hz, 2H), 2.28 (s, 3H), 1.32 (t, J = 7.1 Hz, 3H). ^{13}C NMR (101 MHz, $CDCl_3$) δ 168.1, 166.0, 151.0, 143.5, 141.5, 140.7, 138.8, 136.6, 135.5, 133.4, 133.1, 131.2, 130.7, 130.3, 129.9, 127.9, 124.0, 123.0, 117.7, 117.3, 113.1, 61.0, 48.6, 35.4, 21.1, 14.4. HRMS (m/z, ESI-TOF): Calcd for $C_{28}H_{25}BrN_3O_7S^+$ [M+H$^+$] 626.0591, found 626.0592.

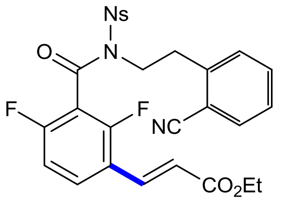

$\mathbf{3s_{mono}}$ **(*E*)-ethyl 3-(3-((2-cyanophenethyl)(4-nitrophenylsulfonyl)carbamoyl)-2,4-difluorophenyl)acrylate**: Conditions variations: Formyl-Gly-OH (60 mol%), $Cu(OAc)_2$ (0.2 equiv), K_2HPO_4 (0.5 equiv), 80 °C, 24 h; 33 mg, 58%. ^{1}H NMR (400 MHz, $CDCl_3$) δ 8.35 (d, J = 8.9 Hz, 2H), 8.01 (d, J = 8.6 Hz, 2H), 7.67-7.55 (m, 4H), 7.45 (d, J = 7.5 Hz, 1H), 7.39 (t, J = 7.6 Hz, 1H), 6.97 (t, J = 8.4 Hz, 1H), 6.41 (d, J = 16.2 Hz, 1H), 4.28 (q, J = 7.1 Hz, 2H), 4.21 (t, J = 7.2 Hz, 2H), 3.30 (t, J = 7.2 Hz, 2H), 1.34 (t, J = 7.1 Hz, 3H). ^{13}C NMR (101 MHz, $CDCl_3$) δ 166.2, 160.8, 159.3 (dd, $J_{C\text{-}F}$ = 257, 6.9 Hz), 157.1 (dd, $J_{C\text{-}F}$ = 260, 7.1 Hz), 151.0, 143.6, 140.5, 134.7, 133.5, 133.2, 132.0 (dd, $J_{C\text{-}F}$ = 9.9, 4.6 Hz), 131.2, 129.8, 128.0, 124.5, 122.3 (dd, $J_{C\text{-}F}$ = 6.6, 2.2 Hz), 119.7 (dd, $J_{C\text{-}F}$ = 12.0, 4.1 Hz), 117.6, 112.9-112.6 (m), 61.1, 47.8, 34.8, 14.4. HRMS (m/z, ESI-TOF): Calcd for $C_{27}H_{22}F_2N_3O_7S^+$ [M+H$^+$] 570.1141, found 570.1146.

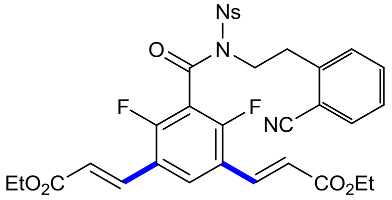

$\mathbf{3s_{(\mathit{m,m'})di}}$ **(2*E*,2'*E*)-diethyl 3,3'-(5-((2-cyanophenethyl)(4-nitrophenylsulfonyl)carbamoyl)-4,6-difluoro-1,3-phenylene)diacrylate**: Conditions variations: Formyl-Gly-OH (60 mol%), $Cu(OAc)_2$ (0.2 equiv), K_2HPO_4 (0.5 equiv), 80 °C, 24 h; 5 mg, 7%. ^{1}H NMR (400 MHz, $CDCl_3$) δ 8.36 (d, J = 8.5 Hz, 2H), 8.00 (d, J = 7.7 Hz, 2H), 7.78 (t, J = 7.8 Hz, 1H), 7.68-7.55 (m, 4H), 7.47 (d, J = 7.8 Hz, 1H), 7.40 (t, J = 7.6 Hz, 1H), 6.46 (d, J = 16.2 Hz, 2H), 4.29 (q, J = 7.0 Hz, 4H), 4.22 (t, J = 7.0 Hz, 2H), 3.31 (t, J = 6.9 Hz, 2H), 1.35 (t, J = 7.1 Hz, 6H). ^{13}C NMR (101 MHz, $CDCl_3$) δ 166.0, 160.3, 157.3 (dd, $J_{C\text{-}F}$ = 263, 7.4 Hz), 151.1, 143.5, 140.5, 134.2, 133.5, 133.2, 131.2, 130.8 (t, $J_{C\text{-}F}$ = 5.2 Hz), 129.7, 128.1, 124.6, 123.2 (t, $J_{C\text{-}F}$ = 4.1 Hz), 120.3 (dd, $J_{C\text{-}F}$ = 11.6, 5.6 Hz), 117.6, 112.8, 61.2, 47.8, 34.7, 14.4. HRMS (m/z, ESI-TOF): Calcd for $C_{32}H_{28}F_2N_3O_9S^+$ [M+H$^+$] 668.1509, found 668.1511.

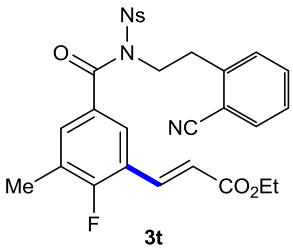

3t **(*E*)-ethyl 3-(5-((2-cyanophenethyl)(4-nitrophenylsulfonyl)carbamoyl)-2-fluoro-3-methylphenyl)acrylate**: Conditions variations: Ac-Gly-OH (100 mol%), $Cu(OAc)_2$ (1.0 equiv), 90 °C, 48 h; 42 mg, 74%. ^{1}H NMR (400 MHz,

$CDCl_3$) δ 8.37 (d, J = 8.8 Hz, 2H), 8.13 (d, J = 8.8 Hz, 2H), 7.70 (d, J = 16.2 Hz, 1H), 7.58-7.48 (m, 2H), 7.36 (t, J = 7.6 Hz, 1H), 7.30 (d, J = 7.7 Hz, 1H), 7.18 (t, J = 5.3 Hz, 2H), 6.37 (d, J = 16.2 Hz, 1H), 4.28 (q, J = 7.2 Hz, 2H), 4.22 (t, J = 7.0 Hz, 2H), 3.19 (t, J = 6.9 Hz, 2H), 2.29 (s, 3H), 1.35 (t, J = 7.1 Hz, 3H). ^{13}C NMR (101 MHz, $CDCl_3$) δ 169.9, 166.3, 161.8 (d, $J_{C\text{-}F}$ = 261 Hz), 150.8, 144.1, 140.7, 135.7 (d, $J_{C\text{-}F}$ = 3.4 Hz), 133.3, 133.2, 132.6 (d, $J_{C\text{-}F}$ = 6.8 Hz), 131.0, 130.1, 129.9 (d, $J_{C\text{-}F}$ = 4.0 Hz), 127.9, 126.9 (d, $J_{C\text{-}F}$ = 19.0 Hz), 126.7 (d, $J_{C\text{-}F}$ = 4.0 Hz), 124.4, 122.6 (d, $J_{C\text{-}F}$ = 13.8 Hz), 122.4 (d, $J_{C\text{-}F}$ = 6.0 Hz), 117.4, 113.0, 61.0, 48.2, 34.7, 14.7 (d, $J_{C\text{-}F}$ = 4.3 Hz), 14.4. HRMS (m/z, ESI-TOF): Calcd for $C_{28}H_{25}FN_3O_7S^+$ [M+H$^+$] 566.1392, found 566.1400.

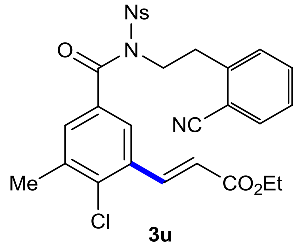

3u

(*E*)-ethyl 3-(2-chloro-5-((2-cyanophenethyl)(4-nitrophenylsulfonyl)carbamoyl)-3-methylphenyl)acrylate: Conditions variations: Formyl-Gly-OH (60 mol%), $Cu(OAc)_2$ (0.5 equiv), 90 °C, 48 h; 48 mg, 82%. ^{1}H NMR (400 MHz, $CDCl_3$) δ 8.36 (d, J = 8.7 Hz, 2H), 8.10 (d, J = 8.7 Hz, 2H), 8.03 (d, J = 16.0 Hz, 1H), 7.58-7.49 (m, 2H), 7.35 (t, J = 7.6 Hz, 1H), 7.30 (d, J = 7.6 Hz, 1H), 7.23 (s, 1H), 7.18 (s, 1H), 6.20 (d, J = 16.0 Hz, 1H), 4.34-4.18 (m, 4H), 3.20 (t, J = 6.9 Hz, 2H), 2.39 (s, 3H), 1.35 (t, J = 7.1 Hz, 3H). ^{13}C NMR (101 MHz, $CDCl_3$) δ 169.9, 166.1, 150.8, 144.0, 140.7, 139.6, 139.1, 138.3, 133.5, 133.3, 133.2, 132.2, 131.0, 130.9, 130.0, 127.9, 124.8, 124.4, 122.5, 117.4, 112.9, 61.1, 48.1, 34.7, 20.9, 14.4. HRMS (m/z, ESI-TOF): Calcd for $C_{28}H_{25}ClN_3O_7S^+$ [M+H$^+$] 582.1096, found 582.1103.

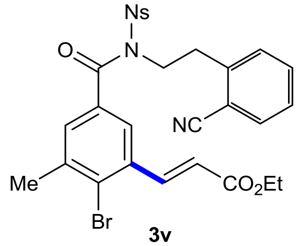

3v

(*E*)-ethyl 3-(2-bromo-5-((2-cyanophenethyl)(4-nitrophenylsulfonyl)carbamoyl)-3-methylphenyl)acrylate: Conditions variations: Ac-Gly-OH (100 mol%), $Cu(OAc)_2$ (1.0 equiv), 90 °C, 48 h; 48 mg, 77%. ^{1}H NMR (400 MHz, $CDCl_3$) δ 8.37 (d, J = 8.9 Hz, 2H), 8.10 (d, J = 8.9 Hz, 2H), 8.03 (d, J = 15.9 Hz, 1H), 7.58-7.50 (m, 2H), 7.36 (t, J = 7.4 Hz, 1H), 7.30 (d, J = 7.7 Hz, 1H), 7.17 (d, J = 9.4 Hz, 2H), 6.16 (d, J = 15.9 Hz, 1H), 4.29 (q, J = 7.2 Hz, 2H), 4.23 (t, J = 7.0 Hz, 2H), 3.20 (t, J = 6.9 Hz, 2H), 2.43 (s, 3H), 1.36 (t, J = 7.1 Hz, 3H). ^{13}C NMR (101 MHz, $CDCl_3$) δ 169.9, 166.0, 150.8, 144.0, 142.5, 140.7, 140.4, 135.7, 133.3, 133.2, 132.9, 132.0, 131.0, 130.5, 130.1, 128.0, 124.8, 124.5, 122.7, 117.4, 113.0, 61.1, 48.1, 34.7, 24.1, 14.4. HRMS (m/z, ESI-TOF): Calcd for $C_{28}H_{25}BrN_3O_7S^+$ [M+H$^+$] 626.0591, found 626.0600.

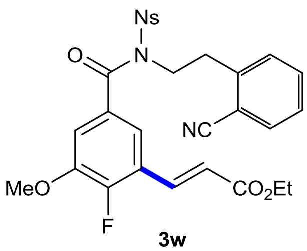

(*E*)-ethyl 3-(5-((2-cyanophenethyl)(4-nitrophenylsulfonyl)carbamoyl)-2-fluoro-3-methoxyphenyl)acrylate: Conditions variations: Formyl-Gly-OH (60 mol%), $Cu(OAc)_2$ (0.5 equiv), 90 °C, 48 h; 46 mg, 79%. ^{1}H NMR (400 MHz, $CDCl_3$) δ 8.37 (d, J = 8.8 Hz, 2H), 8.11 (d, J = 8.9 Hz, 2H), 7.71 (d, J = 16.2 Hz, 1H), 7.59-7.49 (m, 2H), 7.36 (t, J = 7.2 Hz, 1H), 7.29 (d, J = 7.6 Hz, 1H), 6.98 (t, J = 6.9 Hz, 2H), 6.33 (d, J = 16.2 Hz, 1H), 4.34-4.15 (m, 4H), 3.89 (s, 3H), 3.19 (t, J = 6.9 Hz, 2H), 1.35 (t, J = 7.1 Hz, 3H). ^{13}C NMR (101 MHz, $CDCl_3$) δ 169.8, 166.2, 153.3 (d, J_{C-F} = 264 Hz), 150.8, 148.6 (d, J_{C-F} = 11 Hz), 144.0, 140.8, 135.2 (d, J_{C-F} = 3.7 Hz), 133.3 (d, J_{C-F} = 3.3 Hz), 130.9, 130.0, 128.0, 124.5, 123.2 (d, J_{C-F} = 10.4 Hz), 122.8 (d, J_{C-F} = 5.8 Hz), 120.0 (d, J_{C-F} = 2.3 Hz), 117.4, 114.1 (d, J_{C-F} = 2.9 Hz), 113.0, 61.1, 56.7, 48.3, 34.6, 14.4. HRMS (m/z, ESI-TOF): Calcd for $C_{28}H_{25}FN_3O_8S^+$ [M+H$^+$] 582.1341, found 582.1349.

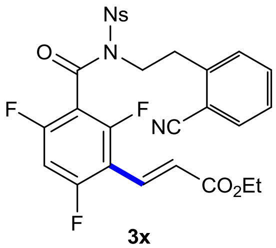

(*E*)-ethyl 3-(3-((2-cyanophenethyl)(4-nitrophenylsulfonyl)carbamoyl)-2,4,6-trifluorophenyl)acrylate: Conditions variations: Formyl-Gly-OH (60 mol%), $Cu(OAc)_2$ (0.2 equiv), 90 °C, 48 h; 35 mg, 60%. ^{1}H NMR (400 MHz, $CDCl_3$) δ 8.37 (d, J = 8.9 Hz, 2H), 8.03 (d, J = 8.8 Hz, 2H), 7.67-7.50 (m, 3H), 7.45 (d, J = 7.6 Hz, 1H), 7.39 (t, J = 7.3 Hz, 1H), 6.80 (t, J = 9.4 Hz, 1H), 6.58 (d, J = 16.4 Hz, 1H), 4.28 (q, J = 7.1 Hz, 2H), 4.18 (t, J = 7.3 Hz, 2H), 3.29 (t, J = 7.3 Hz, 2H), 1.34 (t, J = 7.1 Hz, 3H). ^{13}C NMR (101 MHz, $CDCl_3$) δ 166.3, 160.1, 151.1, 143.6, 140.5, 133.5, 133.2, 131.1, 129.7, 128.4, 128.1, 125.6 (td, J_{C-F} = 7.8, 1.1 Hz), 124.6, 117.6, 112.8, 102.1-101.5 (m), 61.2, 47.7, 34.7, 14.4 (The intensity of some peaks is low due to the couplings of carbon and the fluorine atoms). HRMS (m/z, ESI-TOF): Calcd for $C_{27}H_{21}F_3N_3O_7S^+$ [M+H$^+$] 588.1047, found 588.1054.

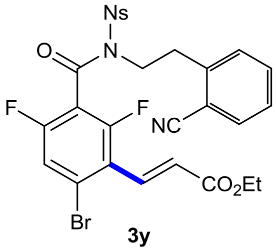

(*E*)-ethyl 3-(6-bromo-3-((2-cyanophenethyl)(4-nitrophenylsulfonyl)carbamoyl)-2,4-difluorophenyl)acrylate: Conditions variations: Ac-Gly-OH (60 mol%), $Cu(OAc)_2$ (0.5 equiv), 90 °C, 48 h; 33 mg, 51%. ^{1}H NMR (400 MHz, $CDCl_3$) δ 8.37 (d, J = 8.9 Hz, 2H), 8.02 (d, J = 8.7 Hz, 2H), 7.68 (d, J = 16.3 Hz, 1H), 7.64-7.55 (m, 2H), 7.45 (d, J = 7.6 Hz, 1H), 7.39 (t, J = 7.6 Hz, 1H), 7.31 (d, J = 8.0 Hz, 1H), 6.42 (d, J = 16.3 Hz, 1H), 4.29 (q, J = 7.1 Hz, 2H), 4.18 (t, J = 7.4 Hz, 2H), 3.29 (t, J = 7.4 Hz, 2H), 1.35 (t, J = 7.1 Hz, 3H). ^{13}C NMR (101 MHz, $CDCl_3$) δ 166.2, 151.1, 143.6, 140.5, 135.1, 133.5, 133.2, 131.1, 129.7, 128.1, 126.6 (dd, J_{C-F} = 13.5, 1.2 Hz), 124.7, 117.8-117.5 (m), 112.8, 61.3, 47.7, 34.6,

14.4 (The intensity of some peaks is low due to the couplings of carbon and the fluorine atoms). HRMS (m/z, ESI-TOF): Calcd for $C_{27}H_{21}BrF_2N_3O_7S^+$ [M+H$^+$] 648.0246, found 648.0236.

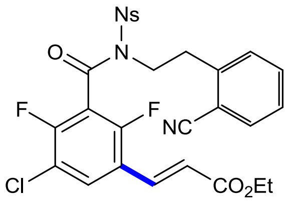

3z **(*E*)-ethyl 3-(5-chloro-3-((2-cyanophenethyl)(4-nitrophenylsulfonyl)carbamoyl)-2,4-difluorophenyl)acrylate**: Conditions variations: Formyl-Gly-OH (60 mol%), $Cu(OAc)_2$ (0.2 equiv), 80 °C, 48 h; 49 mg, 81%. ^{1}H NMR (400 MHz, $CDCl_3$) δ 8.36 (d, *J* = 8.7 Hz, 2H), 7.99 (d, *J* = 8.2 Hz, 2H), 7.68 (t, *J* = 7.6 Hz, 1H), 7.64-7.51 (m, 3H), 7.45 (d, *J* = 7.6 Hz, 1H), 7.39 (t, *J* = 7.6 Hz, 1H), 6.43 (d, *J* = 16.2 Hz, 1H), 4.27 (q, *J* = 7.1 Hz, 2H), 4.21 (t, *J* = 7.1 Hz, 2H), 3.30 (t, *J* = 7.2 Hz, 2H), 1.33 (t, *J* = 7.1 Hz, 3H). ^{13}C NMR (101 MHz, $CDCl_3$) δ 165.8, 159.8, 155.2 (dd, $J_{C\text{-}F}$ = 259, 5.5 Hz), 154.4 (dd, $J_{C\text{-}F}$ = 258, 7.3 Hz), 151.1, 143.3, 140.4, 133.5, 133.5, 133.2, 131.4 (d, $J_{C\text{-}F}$ = 4.1 Hz), 131.2, 129.6, 128.1, 124.6, 123.4 (dd, $J_{C\text{-}F}$ = 5.8, 1.8 Hz), 120.7 (dd, $J_{C\text{-}F}$ = 13.1, 4.3 Hz), 118.1 (dd, $J_{C\text{-}F}$ = 18.0, 3.8 Hz), 117.6, 112.7, 61.2, 47.7, 34.6, 14.3. HRMS (m/z, ESI-TOF): Calcd for $C_{27}H_{21}ClF_2N_3O_7S^+$ [M+H$^+$] 604.0751, found 604.0757.

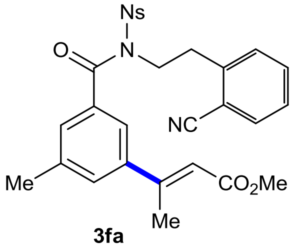

3fa **(*E*)-methyl 3-(3-((2-cyanophenethyl)(4-nitrophenylsulfonyl)carbamoyl)-5-methylphenyl)but-2-enoate**: Conditions variations: Ac-Gly-OH (60 mol%), $Cu(OAc)_2$ (0.5 equiv), 90 °C, 48 h; 33 mg, 60%. ^{1}H NMR (400 MHz, $CDCl_3$) δ 8.36 (d, *J* = 8.8 Hz, 2H), 8.15 (d, *J* = 8.8 Hz, 2H), 7.54 (td, *J* = 7.6, 0.9 Hz, 1H), 7.49 (d, *J* = 7.6 Hz, 1H), 7.38 (s, 1H), 7.37-7.28 (m, 2H), 6.99 (s, 2H), 6.00 (d, *J* = 0.9 Hz, 1H), 4.28 (t, *J* = 6.9 Hz, 2H), 3.76 (s, 3H), 3.20 (t, *J* = 6.9 Hz, 2H), 2.49 (d, *J* = 0.8 Hz, 3H), 2.35 (s, 3H). ^{13}C NMR (101 MHz, $CDCl_3$) δ 170.8, 166.9, 154.1, 150.8, 144.3, 142.7, 140.8, 139.1, 134.0, 133.3, 133.2, 131.1, 130.8, 130.2, 128.6, 127.9, 124.3, 123.0, 118.0, 117.3, 113.0, 51.4, 48.3, 35.0, 21.4, 18.0. HRMS (m/z, ESI-TOF): Calcd for $C_{28}H_{26}N_3O_7S^+$ [M+H$^+$] 548.1486, found 548.1496.

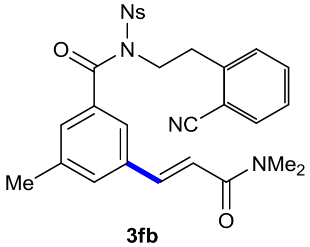

3fb **(*E*)-*N*-(2-cyanophenethyl)-3-(3-(dimethylaminooxy)-3-oxoprop-1-enyl)-5-methyl-*N*-(4-nitrophenylsulfonyl)benzamide**: Conditions variations: Ac-Gly-OH (60 mol%), $Cu(OAc)_2$ (0.5 equiv), 90 °C, 48 h; 36 mg, 66%. ^{1}H NMR (400 MHz, $CDCl_3$) δ 8.36 (d, *J* = 8.8 Hz, 2H), 8.17 (d, *J* = 8.8 Hz, 2H), 7.58-7.46 (m, 3H), 7.43 (s, 1H), 7.35 (t, *J* = 7.5 Hz, 1H), 7.28 (s, 1H), 7.08 (s, 1H), 6.98 (s, 1H), 6.83 (d, *J* = 15.4 Hz, 1H), 4.27 (t, *J* = 6.9 Hz, 2H),

3.26-3.12 (m, 5H), 3.07 (s, 3H), 2.35 (s, 3H). ^{13}C NMR (101 MHz, $CDCl_3$) δ 170.8, 166.2, 150.8, 144.3, 140.7, 140.7, 139.3, 136.1, 134.2, 133.2, 133.2, 132.1, 131.0, 130.3, 128.8, 127.9, 124.3, 124.2, 119.4, 117.4, 113.0, 48.4, 37.6, 36.1, 35.1, 21.3. HRMS (m/z, ESI-TOF): Calcd for $C_{28}H_{27}N_4O_6S^+$ [M+H$^+$] 547.1646, found 547.1658.

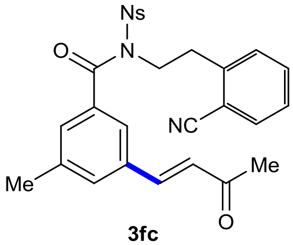

3fc **(*E*)-*N*-(2-cyanophenethyl)-3-methyl-*N*-(4-nitrophenylsulfonyl)-5-(3-oxobut-1-enyl)benzamide**: Conditions variations: Ac-Gly-OH (20 mol%), AgOAc (3.0 equiv), air, 80 °C, 24 h; 37 mg, 72%. ^{1}H NMR (400 MHz, $CDCl_3$) δ 8.36 (d, *J* = 8.8 Hz, 2H), 8.16 (d, *J* = 8.9 Hz, 2H), 7.54 (t, *J* = 7.6 Hz, 1H), 7.49 (d, *J* = 7.2 Hz, 1H), 7.46 (s, 1H), 7.41-7.33 (m, 2H), 7.31 (d, *J* = 7.7 Hz, 1H), 7.11 (s, 1H), 7.02 (s, 1H), 6.62 (d, *J* = 16.3 Hz, 1H), 4.25 (t, *J* = 7.0 Hz, 2H), 3.21 (t, *J* = 7.0 Hz, 2H), 2.37 (s, 3H), 2.35 (s, 3H). ^{13}C NMR (101 MHz, $CDCl_3$) δ 197.9, 170.6, 150.8, 144.2, 141.4, 140.7, 139.6, 135.1, 134.5, 133.3, 133.2, 132.4, 131.1, 130.3, 129.9, 128.5, 127.9, 124.6, 124.3, 117.3, 113.0, 48.4, 35.0, 27.9, 21.3. HRMS (m/z, ESI-TOF): Calcd for $C_{27}H_{24}N_3O_6S^+$ [M+H$^+$] 518.1380, found 518.1386.

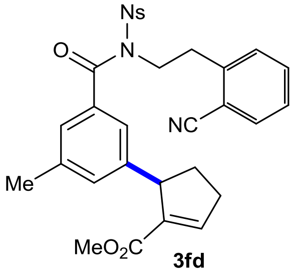

3fd **methyl 2-(3-((2-cyanophenethyl)(4-nitrophenylsulfonyl)carbamoyl)-5-methylphenyl)cyclopent-2-enecarboxylate**: Conditions variations: Ac-Gly-OH (20 mol%), AgOAc (3.0 equiv), 80 °C, 24 h; 50 mg, 87%. ^{1}H NMR (400 MHz, $CDCl_3$) δ 8.33 (d, *J* = 9.0 Hz, 2H), 8.15 (d, *J* = 9.0 Hz, 2H), 7.58-7.47 (m, 2H), 7.33 (td, *J* = 7.6, 0.8 Hz, 1H), 7.21 (d, *J* = 7.7 Hz, 1H), 7.10 (s, 1H), 7.01 (d, *J* = 1.4 Hz, 1H), 6.93 (s, 1H), 6.77 (s, 1H), 4.25 (t, *J* = 7.2 Hz, 2H), 4.13-4.02 (m, 1H), 3.56 (s, 3H), 3.13 (t, *J* = 7.2 Hz, 2H), 2.77-2.39 (m, 3H), 2.24 (s, 3H), 1.92-1.78 (m, 1H). ^{13}C NMR (101 MHz, $CDCl_3$) δ 171.3, 164.9, 150.7, 145.9, 145.8, 144.5, 140.9, 138.9, 138.5, 133.6, 133.2, 133.1, 131.7, 130.9, 130.3, 127.7, 126.4, 124.2, 123.9, 117.4, 113.0, 51.5, 49.9, 48.3, 34.9, 33.8, 32.4, 21.3. HRMS (m/z, ESI-TOF): Calcd for $C_{30}H_{28}N_3O_7S^+$ [M+H$^+$] 574.1642, found 574.1652.

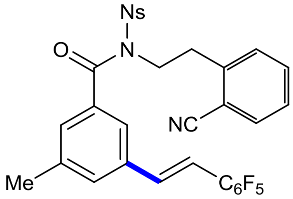

3fe **(*E*)-*N*-(2-cyanophenethyl)-3-(1,2-difluoro-2-(perfluorophenyl)vinyl)-5-methyl-*N*-(4-nitrophenylsulfonyl)benzamide**: Conditions variations: Ac-Gly-OH (60 mol%), $Cu(OAc)_2$ (0.5 equiv), 90 °C, 48 h; 40 mg, 62%. ^{1}H NMR (400 MHz, $CDCl_3$) δ 8.36 (d, *J* = 8.8 Hz, 2H), 8.16 (d, *J* = 8.8 Hz, 2H), 7.55 (t, *J* = 7.6 Hz, 1H), 7.50 (d, *J* = 7.5 Hz, 1H), 7.44 (s, 1H), 7.38-7.30 (m, 2H), 7.27 (d, *J* = 16.2 Hz, 1H), 6.98 (s, 2H), 6.87 (d, *J* = 16.8 Hz, 1H), 4.31 (t, *J* =

6.8 Hz, 2H), 3.23 (t, J = 6.7 Hz, 2H), 2.37 (s, 3H). ^{13}C NMR (101 MHz, $CDCl_3$) δ 170.8, 150.8, 144.4, 140.8, 139.4, 137.0, 135.6-135.4 (m), 134.4, 133.3, 133.2, 131.2, 131.2, 130.3, 128.3, 127.8, 124.3, 123.4, 117.4, 114.5, 113.1, 48.4, 35.1, 21.4 (The intensity of carbons of C_6F_5 is very low due to the couplings of carbon and the fluorine atoms). HRMS (m/z, ESI-TOF): Calcd for $C_{31}H_{21}F_5N_3O_5S^+$ [M+H$^+$] 642.1117, found 642.1125.

General *meta*-C–H acetoxylation procedure and characterization of acetoxylated compounds.

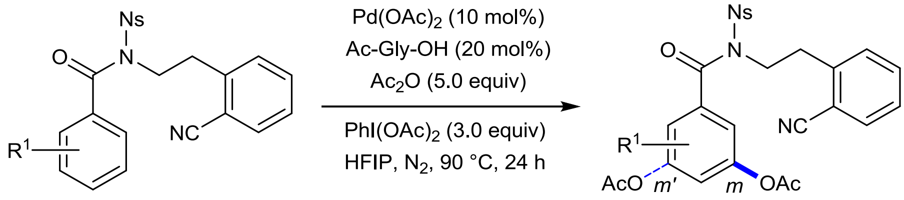

1a-za **6a-za**

To a 50 mL Schlenk sealed tube (with a Teflon cap) equipped with a magnetic stir bar was charged with amide **1** (0.10 mmol, 1.0 equiv), $Pd(OAc)_2$ (2.3 mg, 0.010 mmol, 10 mol%), Ac-Gly-OH (2.4 mg, 0.020 mmol, 20 mol%) and $PhI(OAc)_2$ (96.6 mg, 0.30 mmol, 3 equiv). HFIP (1.0 mL) was added to the mixture along the inside wall of the tube, followed by Ac_2O (47 µL, 5 equiv). The reaction tube was capped, then evacuated briefly under vacuum and charged with N_2 (1 atm, balloon, × 3). The tube was then submerged into a preheated 90 °C oil bath. The reaction was stirred for 24 h and cooled to room temperature. The crude reaction mixture was diluted with EtOAc (5 mL) and filtered through a short pad of Celite. The sealed tube and Celite pad were washed with an additional 20 mL of EtOAc. The filtrate was concentrated in vacuo, and the resulting residue was purified by flash silica gel chromatography using petroleum ether/EtOAc as the eluent.

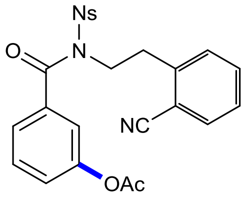

6a$_{mono}$ **3-((2-cyanophenethyl)(4-nitrophenylsulfonyl)carbamoyl)phenyl acetate**: (0.1 mmol scale, 29 mg, 59%; 1.3 mmol scale, 317 mg, 49.4%.) ^{1}H NMR (400 MHz, $CDCl_3$) δ 8.35 (d, J = 8.9 Hz, 2H), 8.16 (d, J = 8.9 Hz, 2H), 7.59-7.47 (m, 2H), 7.43-7.30 (m, 2H), 7.29-7.22 (m, 2H), 7.17 (d, J = 7.7 Hz, 1H), 7.02 (s, 1H), 4.23 (t, J = 7.2 Hz, 2H), 3.19 (t, J = 7.2 Hz, 2H), 2.30 (s, 3H). ^{13}C NMR (101 MHz, $CDCl_3$) δ 169.9, 169.0, 150.8, 150.5, 144.1, 140.7, 134.8, 133.3, 133.3, 130.9, 130.3, 129.9, 127.9, 125.6, 125.2, 124.4, 121.3, 117.5, 112.9, 48.39, 34.9, 21.2. HRMS (m/z, ESI-TOF): Calcd for $C_{24}H_{20}N_3O_7S^+$ [M+H$^+$] 494.1016, found 494.1020.

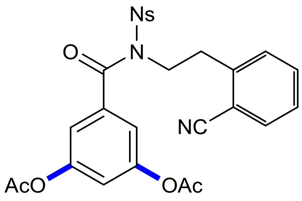

6a$_{(m,m')di}$ **5-((2-cyanophenethyl)(4-nitrophenylsulfonyl)carbamoyl)-1,3-phenylene diacetate**: (0.1 mmol scale, 12 mg, 22%; 1.3 mmol scale, 75 mg, 10.4%.) ^{1}H NMR (400 MHz, $CDCl_3$) δ 8.33 (d, *J* = 9.0 Hz, 2H), 8.11 (d, *J* = 9.0 Hz, 2H), 7.58 (d, *J* = 7.0 Hz, 1H), 7.52 (td, *J* = 7.6, 1.1 Hz, 1H), 7.37-7.31 (m, 1H), 7.29-7.26 (m, 1H), 7.07 (t, *J* = 2.0 Hz, 1H), 7.02-6.89 (m, 2H), 4.23 (t, *J* = 7.3 Hz, 2H), 3.20 (t, *J* = 7.3 Hz, 2H), 2.29 (s, 6H). ^{13}C NMR (101 MHz, $CDCl_3$) δ 169.0, 168.6, 151.1, 150.9, 144.0, 140.8, 135.4, 133.3, 133.3, 130.8, 130.2, 127.9, 124.4, 119.2, 118.7, 117.6, 113.0, 48.2, 34.7, 21.1. HRMS (m/z, ESI-TOF): Calcd for $C_{26}H_{22}N_3O_9S^+$ [M+H$^+$] 552.1071, found 552.1073.

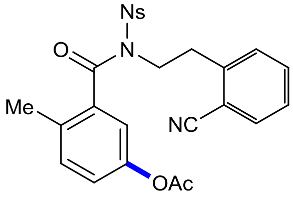

6b$_{mono}$ **3-((2-cyanophenethyl)(4-nitrophenylsulfonyl)carbamoyl)-4-methylphenyl acetate**: (0.1 mmol scale, 6 h; 26 mg, 51%.) ^{1}H NMR (400 MHz, $CDCl_3$) δ 8.34 (d, *J* = 8.8 Hz, 2H), 8.12 (d, *J* = 8.8 Hz, 2H), 7.61 (d, *J* = 7.7 Hz, 1H), 7.54 (t, *J* = 7.7 Hz, 1H), 7.36 (t, *J* = 7.6 Hz, 1H), 7.30 (d, *J* = 7.7 Hz, 1H), 7.16 (d, *J* = 8.4 Hz, 1H), 7.08 (dd, *J* = 8.3, 2.1 Hz, 1H), 6.67 (d, *J* = 2.0 Hz, 1H), 4.22-4.05 (m, 2H), 3.24 (t, *J* = 7.4 Hz, 2H), 2.28 (s, 3H), 2.07 (s, 3H). ^{13}C NMR (101 MHz, $CDCl_3$) δ 169.6, 169.2, 150.8, 148.4, 144.3, 140.9, 134.7, 133.3, 133.2, 132.7, 131.9, 130.7, 130.3, 127.9, 124.3, 124.0, 119.8, 117.6, 113.0, 47.9, 35.0, 21.2, 18.6. HRMS (m/z, ESI-TOF): Calcd for $C_{25}H_{22}N_3O_7S^+$ [M+H$^+$] 508.1173, found 508.1175.

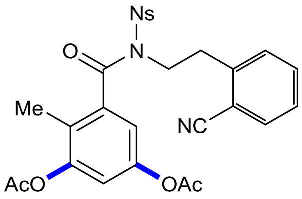

6b$_{(m,m')di}$ **5-((2-cyanophenethyl)(4-nitrophenylsulfonyl)carbamoyl)-4-methyl-1,3-phenylene diacetate**: (0.1 mmol scale, 6 h; 11 mg, 19%.) ^{1}H NMR (400 MHz, $CDCl_3$) δ 8.32 (d, *J* = 8.9 Hz, 2H), 8.02 (d, *J* = 8.9 Hz, 2H), 7.63 (d, *J* = 7.7 Hz, 1H), 7.54 (t, *J* = 7.1 Hz, 1H), 7.36 (t, *J* = 7.4 Hz, 1H), 7.29 (d, *J* = 7.9 Hz, 1H), 6.96 (s, 1H), 6.84 (s, 1H), 4.20 (t, *J* = 7.5 Hz, 2H), 3.26 (t, *J* = 7.6 Hz, 2H), 2.31 (s, 3H), 2.26 (s, 3H), 2.05 (s, 3H). ^{13}C NMR (101 MHz, $CDCl_3$) δ 168.9, 168.0, 167.9, 150.9, 144.3, 143.6, 140.9, 139.9, 134.6, 133.4, 133.2, 131.9, 130.7, 130.2, 127.9, 125.8, 124.4, 122.2, 117.7, 113.0, 47.8, 34.9, 20.7, 20.7, 18.8. HRMS (m/z, ESI-TOF): Calcd for $C_{27}H_{24}N_3O_9S^+$ [M+H$^+$] 566.1228, found 566.1237.

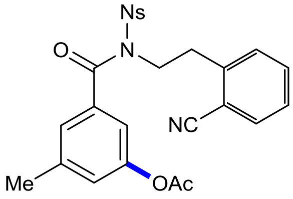

6f

3-((2-cyanophenethyl)(4-nitrophenylsulfonyl)carbamoyl)-5-methylphenyl acetate: (0.1 mmol scale, 39.6 mg, 78%.) ^{1}H NMR (400 MHz, $CDCl_3$) δ 8.35 (d, *J* = 8.9 Hz, 2H), 8.17 (d, *J* = 8.9 Hz, 2H), 7.59-7.48 (m, 2H), 7.34 (t, *J* = 7.3 Hz, 1H), 7.27 (d, *J* = 5.7 Hz, 1H), 7.05 (s, 1H), 6.89 (s, 1H), 6.82 (s, 1H), 4.24 (t, *J* = 7.2 Hz, 2H), 3.18 (t, *J* = 7.2 Hz, 2H), 2.32 (s, 3H), 2.29 (s, 3H). ^{13}C NMR (101 MHz, $CDCl_3$) δ 170.1, 169.1, 150.8, 150.4, 144.2, 140.8, 140.6, 134.5, 133.3, 133.2, 130.9, 130.3, 127.8, 126.2, 125.8, 124.3, 118.3, 117.5, 112.9, 48.4, 34.9, 21.3, 21.2. HRMS (m/z, ESI-TOF): Calcd for $C_{25}H_{22}N_3O_7S^+$ [M+H$^+$] 508.1173, found 508.1172.

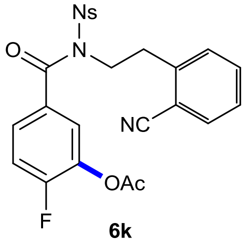

6k

5-((2-cyanophenethyl)(4-nitrophenylsulfonyl)carbamoyl)-2-fluorophenyl acetate: (0.2 mmol scale, 62 mg, 61%.) ^{1}H NMR (400 MHz, $CDCl_3$) δ 8.35 (d, *J* = 8.8 Hz, 2H), 8.11 (d, *J* = 8.8 Hz, 2H), 7.57 (d, *J* = 7.7 Hz, 1H), 7.52 (t, *J* = 7.7 Hz, 1H), 7.34 (t, *J* = 7.4 Hz, 1H), 7.30-7.24 (m, 2H), 7.22-7.12 (m, 2H), 4.19 (t, *J* = 7.2 Hz, 2H), 3.19 (t, *J* = 7.2 Hz, 2H), 2.34 (s, 3H). ^{13}C NMR (101 MHz, $CDCl_3$) δ 169.2, 167.9, 156.7 (d, $J_{C\text{-}F}$ = 259 Hz), 150.9, 144.0, 140.7, 138.3 (d, $J_{C\text{-}F}$ = 13.5 Hz), 133.3, 130.8, 130.3 (d, $J_{C\text{-}F}$ = 4.0 Hz), 130.1, 128.0, 127.7 (d, $J_{C\text{-}F}$ = 8.3 Hz), 124.6 (d, $J_{C\text{-}F}$ = 2.0 Hz), 124.5, 117.5, 117.2 (d, $J_{C\text{-}F}$ = 20.0 Hz), 112.9, 48.3, 34.7, 20.5. HRMS (m/z, ESI-TOF): Calcd for $C_{24}H_{19}FN_3O_7S^+$ [M+H$^+$] 512.0922, found 512.0926.

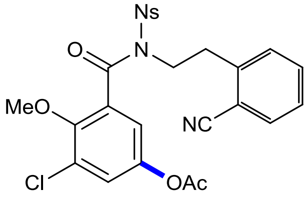

6n

3-chloro-5-((2-cyanophenethyl)(4-nitrophenylsulfonyl)carbamoyl)-4-methoxyphenyl acetate: (0.1 mmol scale, 43 mg, 77%.) ^{1}H NMR (400 MHz, $CDCl_3$) δ 8.32 (d, *J* = 8.9 Hz, 2H), 8.06 (d, *J* = 8.9 Hz, 2H), 7.61 (d, *J* = 7.4 Hz, 1H), 7.59-7.53 (m, 1H), 7.40-7.32 (m, 2H), 7.22 (d, *J* = 2.7 Hz, 1H), 6.71 (d, *J* = 2.7 Hz, 1H), 4.15 (t, *J* = 7.1 Hz, 2H), 3.57 (s, 3H), 3.24 (t, *J* = 7.3 Hz, 2H), 2.30 (s, 3H). ^{13}C NMR (101 MHz, $CDCl_3$) δ 168.9, 166.5, 150.8, 149.8, 146.5, 143.9, 140.9, 133.2, 133.2, 130.8, 130.7, 130.1, 128.6, 127.8, 126.4, 124.1, 119.8, 117.6, 113.1, 62.4, 47.8, 35.0, 21.1. HRMS (m/z, ESI-TOF): Calcd for $C_{25}H_{21}ClN_3O_8S^+$ [M+H$^+$] 558.0732, found 558.0721.

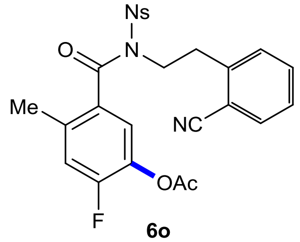

6o **5-((2-cyanophenethyl)(4-nitrophenylsulfonyl)carbamoyl)-2-fluoro-4-methylphenyl acetate**: (0.1 mmol scale, 33 mg, 63%.) ^{1}H NMR (400 MHz, $CDCl_3$) δ 8.34 (d, *J* = 8.9 Hz, 2H), 8.09 (d, *J* = 8.9 Hz, 2H), 7.62 (d, *J* = 7.7 Hz, 1H), 7.55 (td, *J* = 7.6, 1.0 Hz, 1H), 7.41-7.29 (m, 2H), 6.96 (d, *J* = 10.5 Hz, 1H), 6.71 (d, *J* = 7.3 Hz, 1H), 4.15 (t, *J* = 7.2 Hz, 2H), 3.23 (t, *J* = 7.5 Hz, 2H), 2.32 (s, 3H), 2.08 (s, 3H). ^{13}C NMR (101 MHz, $CDCl_3$) δ 168.8, 168.2, 154.9 (d, J_{C-F} = 256 Hz), 150.8, 144.2, 140.7, 135.9-135.6 (m), 133.3, 133.2, 130.7, 130.2, 130.1 (d, J_{C-F} = 3.8 Hz), 127.9, 124.4, 122.5 (d, J_{C-F} = 1.6 Hz), 119.1 (d, J_{C-F} = 19.3 Hz), 117.6, 113.0, 47.9, 34.9, 20.5, 18.9. HRMS (m/z, ESI-TOF): Calcd for $C_{25}H_{21}FN_3O_7S^+$ [M+H$^+$] 526.1079, found 526.1083.

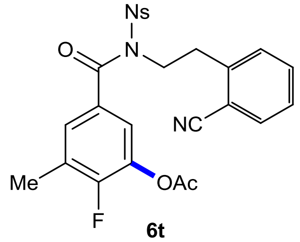

6t **5-((2-cyanophenethyl)(4-nitrophenylsulfonyl)carbamoyl)-2-fluoro-3-methylphenyl acetate**: (0.1 mmol scale, 33 mg, 63%.) ^{1}H NMR (400 MHz, $CDCl_3$) δ 8.36 (d, *J* = 8.9 Hz, 2H), 8.14 (d, *J* = 8.9 Hz, 2H), 7.61-7.47 (m, 2H), 7.34 (t, *J* = 7.3 Hz, 1H), 7.29-7.22 (m, 1H), 7.05 (d, *J* = 4.7 Hz, 1H), 6.97 (dd, *J* = 6.4, 1.7 Hz, 1H), 4.20 (t, *J* = 7.2 Hz, 2H), 3.17 (t, *J* = 7.2 Hz, 2H), 2.34 (s, 3H), 2.27 (d, *J* = 1.2 Hz, 3H). ^{13}C NMR (101 MHz, $CDCl_3$) δ 169.5, 168.1, 155.3 (d, J_{C-F} = 257 Hz), 150.8, 144.0, 140.7, 138.0 (d, J_{C-F} = 14.3 Hz), 133.3, 133.2, 130.9, 130.2, 129.3 (d, J_{C-F} = 4.5 Hz), 128.9 (d, J_{C-F} = 5.5 Hz), 127.9, 127.6 (d, J_{C-F} = 16.2 Hz), 124.4, 121.7, 117.5, 112.9, 48.3, 34.7, 20.6, 14.6 (d, J_{C-F} = 3.3 Hz). HRMS (m/z, ESI-TOF): Calcd for $C_{25}H_{21}FN_3O_7S^+$ [M+H$^+$] 526.1079, found 526.1086.

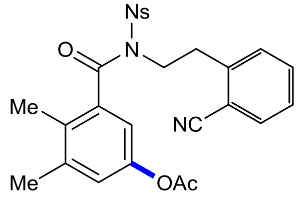

6za **3-((2-cyanophenethyl)(4-nitrophenylsulfonyl)carbamoyl)-4,5-dimethylphenyl acetate**: (0.1 mmol scale, 28 mg, 54%.) ^{1}H NMR (400 MHz, $CDCl_3$) δ 8.33 (d, *J* = 8.8 Hz, 2H), 8.11 (d, *J* = 8.8 Hz, 2H), 7.61 (d, *J* = 7.7 Hz, 1H), 7.54 (t, *J* = 7.7 Hz, 1H), 7.36 (t, *J* = 7.6 Hz, 1H), 7.31 (d, *J* = 7.7 Hz, 1H), 6.98 (s, 1H), 6.52 (d, *J* = 1.7 Hz, 1H), 4.13 (s, 2H), 3.25 (t, *J* = 7.6 Hz, 2H), 2.26 (s, 3H), 2.21 (s, 3H), 1.94 (s, 3H). ^{13}C NMR (101 MHz, $CDCl_3$) δ 170.0, 169.3, 150.8, 148.1, 144.3, 140.9, 139.9, 134.9, 133.3, 133.2, 131.0, 130.7, 130.3, 127.8, 125.1, 124.2, 117.6, 117.2, 113.0, 47.9, 35.0, 21.2, 20.3, 16.0. HRMS (m/z, ESI-TOF): Calcd for $C_{26}H_{24}N_3O_7S^+$ [M+H$^+$] 522.1329, found 522.1334.

Elaborations of the acetoxylated product.

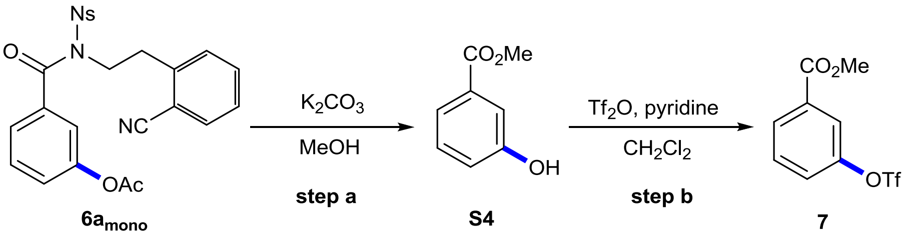

Step a: To a solution of $\mathbf{6a_{mono}}$ (49.3 mg, 0.1 mmol) in MeOH (1 mL) was added K_2CO_3 (41 mg, 0.3 mmol) at room temperature. After stirring for 0.5 h, solvent was removed under reduced pressure and 1N HCl (5 mL) was added. The aqueous phase was extracted with EtOAc (5 mL × 3). The combined organic layer was dried over Na_2SO_4. After removal of the solvent, the residue was purified by flash silica gel chromatography (hexane/EtOAc = 4/1) to give **S4** (15 mg, 99%) as a colorless oil.

Step b: To a stirred solution of **S4** (76 mg, 0.5 mmol) and pyridine (40 μL, 1.0 mmol, 2 equiv) in CH_2Cl_2 (5 mL) at 0 °C was added Tf_2O (100 μL, 0.6 mmol, 1.2 equiv). The reaction was allowed to warm to ambient temperature and its progress was monitored by thin layer chromatography (TLC). When the starting material disappeared, the reaction was quenched with 2N HCl (5 mL) and the resulting mixture was extracted with CH_2Cl_2 (3 × 10 mL). The combined organic phase was dried over Na_2SO_4, filtered, and concentrated under vacuum. The residue was purified by flash chromatography with petroleum ether/EtOAc (10:1) to give compound **7** (135 mg, 95%).

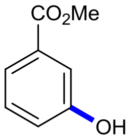

S4 **methyl 3-hydroxybenzoate** (known compound):[1] ^{1}H NMR (400 MHz, $CDCl_3$) δ 7.65-7.55 (m, 2H), 7.30 (t, *J* = 7.9 Hz, 1H), 7.09 (dd, *J* = 8.1, 2.3 Hz, 1H), 6.50 (s, 1H), 3.92 (s, 3H).

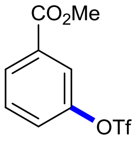

7 **methyl 3-(trifluoromethylsulfonyloxy)benzoate** (known compound):[2] ^{1}H NMR (400 MHz, $CDCl_3$) δ 8.08 (d, *J* = 7.7 Hz, 1H), 7.94 (s, 1H), 7.55 (t, *J* = 8.0 Hz, 1H), 7.48 (dd, *J* = 8.3, 1.4 Hz, 1H), 3.95 (s, 3H).

Amination:[3]

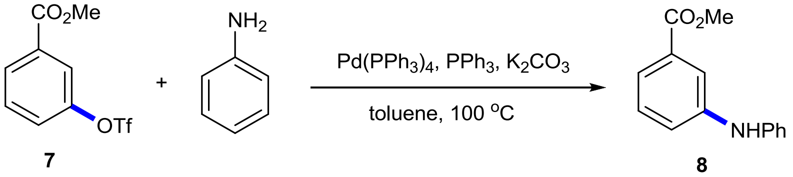

To a reaction tube equipped with a stirring bar charged with toluene (1 mL), **7** (56.8 mg, 0.2 mmol, 1.0 equiv), aniline (25.4 μL, 0.28 mmol, 1.4 equiv), $Pd(PPh_3)_4$ (11.6 mg, 0.01 mmol, 5 mol%), Ph_3P (5.2 mg, 0.02 mmol, 0.1 equiv) and K_2CO_3 (63.6 mg, 0.46 mmol, 2.3 equiv) were

added and the reaction mixture was stirred at 100 ^{o}C for 6 h. Then the reaction was cooled to room temperature and filtered through Celite. The filtrate was diluted in CH_2Cl_2 and washed with H_2O and saturated NaCl aqueous solution. The combined organic layers was dried over anhydrous Na_2SO_4, concentrated under vacuum and purified by flash silica gel chromatography to yield **8** (35.5 mg, 78%).

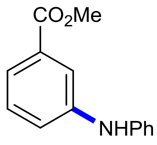

8 **methyl 3-(phenylamino)benzoate** (known compound):[3] ^{1}H NMR (400 MHz, $CDCl_3$) δ 7.72 (s, 1H), 7.57 (d, *J* = 7.5 Hz, 1H), 7.35-7.26 (m, 3H), 7.25-7.22 (m, 1H), 7.09 (d, *J* = 8.1 Hz, 2H), 6.98 (t, *J* = 7.3 Hz, 1H), 5.80 (s, 1H), 3.90 (s, 3H). ^{13}C NMR (101 MHz, $CDCl_3$) δ 167.2, 143.9, 142.5, 131.5, 129.6, 129.5, 122.0, 122.0, 121.6, 118.6, 118.3, 52.3.

Arylation[4]:

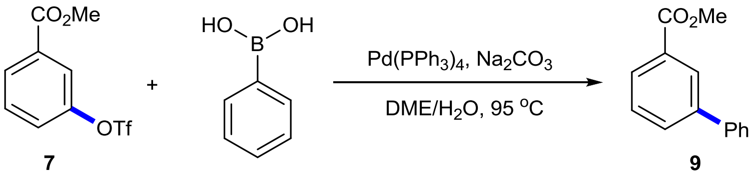

To a mixture of **7** (56.8 mg, 0.2 mmol, 1.0 equiv) and phenylboronic acid (29.2 mg, 0.24 mmol, 1.2 equiv) in 1,2-dimethoxyethane (0.8 mL) was added Na_2CO_3 aqueous solution (2M, 0.1 mL). After the reaction vessel was purged with N_2 for 15 min at room temperature, $Pd(PPh_3)_4$ (11.6 mg, 0.01 mmol, 5 mol%) was added and the reaction was heated at 95 ^{o}C. After starting material disappeared, the reaction mixture was diluted with EtOAc and washed with saturated $NaHCO_3$ aqueous solution. The combined organic layers was dried over anhydrous Na_2SO_4, concentrated under vacuum and purified by flash silica gel chromatography to yield **9** (40.8 mg, 96%).

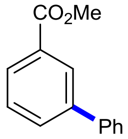

9 **methyl biphenyl-3-carboxylate** (known compound):[4] ^{1}H NMR (400 MHz, $CDCl_3$) δ 8.30 (s, 1H), 8.03 (d, *J* = 7.6 Hz, 1H), 7.79 (d, *J* = 7.5 Hz, 1H), 7.63 (d, *J* = 7.3 Hz, 2H), 7.56-7.42 (m, 3H), 7.39 (t, *J* = 7.1 Hz, 1H), 3.96 (s, 3H). ^{13}C NMR (101 MHz, $CDCl_3$) δ 167.2, 141.6, 140.2, 131.7, 130.8, 129.0, 129.0, 128.5, 128.4, 127.9, 127.3, 52.3.

Alkynylation[5]:

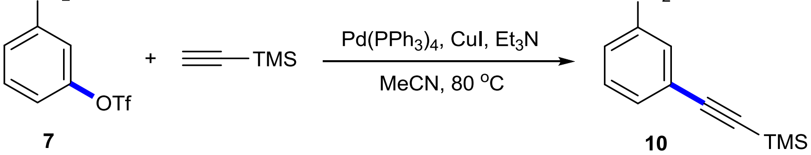

To a solution of **7** (56.8 mg, 0.2 mmol, 1.0 equiv) in MeCN (1 mL) at room temperature was added ethynyltrimethylsilane (84.8 μL, 0.6 mmol, 3.0 equiv), Et_3N (83.5 μL, 0.6 mmol, 3.0 equiv), CuI (2 mg, 0.01 mmol, 5 mol%) and $Pd(PPh_3)_4$ (11.6 mg, 0.01 mmol, 5 mol%). After the

reaction was stirred at 80 °C overnight, MeCN was removed by rotary evaporator. Then the residue was dissolved in EtOAc, washed with H_2O, 1N HCl (aq.) and saturated NaCl aqueous solution. The combined organic layer was dried over anhydrous Na_2SO_4 and concentrated under vacuum to afford a crude product, which was further purified by flash silica gel chromatography to yield **10** (42.8 mg, 92%).

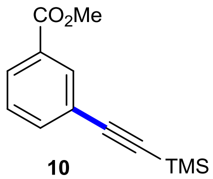

10 **methyl 3-((trimethylsilyl)ethynyl)benzoate** (known compound):[5] ^{1}H NMR (400 MHz, $CDCl_3$) δ 8.13 (s, 1H), 7.97 (d, *J* = 7.8 Hz, 1H), 7.62 (d, *J* = 7.7 Hz, 1H), 7.37 (t, *J* = 7.8 Hz, 1H), 3.91 (s, 3H), 0.25 (s, 9H). ^{13}C NMR (101 MHz, $CDCl_3$) δ 166.5, 136.1, 133.3, 130.5, 129.6, 128.5, 123.8, 104.0, 95.5, 52.4, 0.0.

Cyanation[6]:

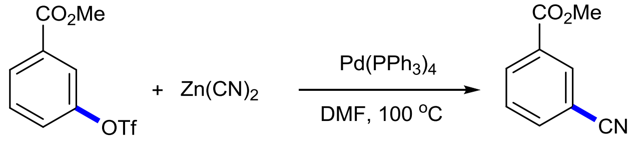

To a mixture of **7** (56.8 mg, 0.2 mmol, 1.0 equiv), $Zn(CN)_2$ (31 mg, 0.26 mmol, 1.3 equiv) and $Pd(PPh_3)_4$ (23.2 mg, 0.02mmol, 10 mol%) in DMF (1 mL) was stirred at 100 °C for 1 h. After The reaction was cooled to room temperature when completed, and it was diluted with EtOAc, washed with H_2O and saturated NaCl aqueous solution. The combined organic layers was dried over anhydrous Na_2SO_4, concentrated under vacuum and purified by flash silica gel chromatography to yield **11** (28.4 mg, 88%).

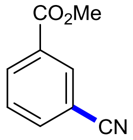

11 **methyl 3-cyanobenzoate** (known compound):[6] ^{1}H NMR (400 MHz, $CDCl_3$) δ 8.32 (s, 1H), 8.26 (d, *J* = 7.9 Hz, 1H), 7.83 (d, *J* = 7.7 Hz, 1H), 7.58 (t, *J* = 7.8 Hz, 1H), 3.95 (s, 3H). ^{13}C NMR (101 MHz, $CDCl_3$) δ 165.2, 136.1, 133.8, 133.4, 131.6, 129.6, 118.0, 113.1, 52.8.

Carbonylation[7]:

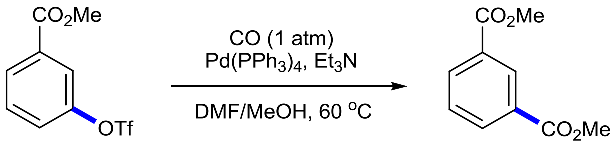

To a solution of **7** (56.8 mg, 0.2 mmol, 1.0 equiv) in MeOH (0.3 mL) and DMF (2 mL) at room temperature was added Et_3N (56 μL, 0.4 mmol, 2.0 equiv) and $Pd(PPh_3)_4$ (23.2 mg, 0.02 mmol, 10 mol%). After stirring at 60 °C for 24 h under CO atmosphere, the reaction mixture was diluted with H_2O and extract with EtOAc (10 mL × 3). The combined organic phases was washed with H_2O, 1N HCl (aq.) and saturated NaCl aqueous solution, dried over anhydrous

Na_2SO_4 and concentrated to afford a crude product, which was further purified by flash silica gel chromatography to yield **12** (39 mg, 100%).

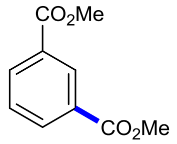

12 **dimethyl isophthalate** (known compound):[7] ^{1}H NMR (400 MHz, $CDCl_3$) δ 8.68 (s, 1H), 8.22 (dd, J = 7.8, 1.5 Hz, 2H), 7.53 (t, J = 7.8 Hz, 1H), 3.95 (s, 6H). ^{13}C NMR (101 MHz, $CDCl_3$) δ 166.4, 133.9, 130.9, 130.8, 128.8, 52.5.

Removal of the directing group and characterization of selected hydrolyzed products.

Method a:

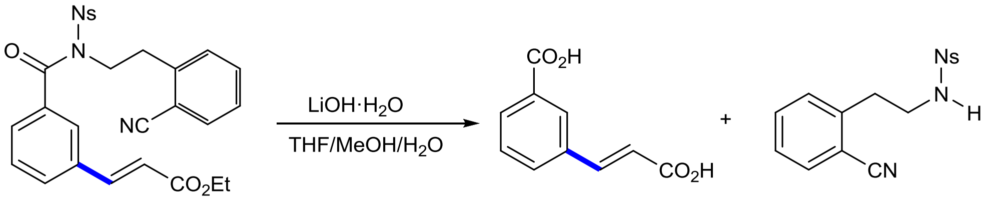

$3a_{mono}$ **$4a_1$** **5**

Compound **$3a_{mono}$** (33 mg, 0.062 mmol) was dissolved in THF (1.5 mL), MeOH (1 mL) and H_2O (0.5 mL). The solution was cooled to 0 ^{o}C and $LiOH·H_2O$ (10 mg, 0.25 mmol) was added. The mixture was warmed to room temperature gradually in 1 h and stirred for another 1 h. H_2O (5 mL) was added and most of the organic solvent was removed under reduced pressure. The aqueous phase was extracted with Et_2O (10 mL × 3). The combined organic phase was dried over anhydrous Na_2SO_4 and concentrated under reduced pressure to recover the pure auxiliary **5** (18 mg) in 88% yield. The aqueous phase was acidized with 2N HCl (5 mL) and extracted with CH_2Cl_2 (10 mL × 3). The combined organic phase was dried over anhydrous Na_2SO_4 and concentrated under reduced pressure to give pure **$4a_1$** (11 mg) in 92% yield.

Method b:

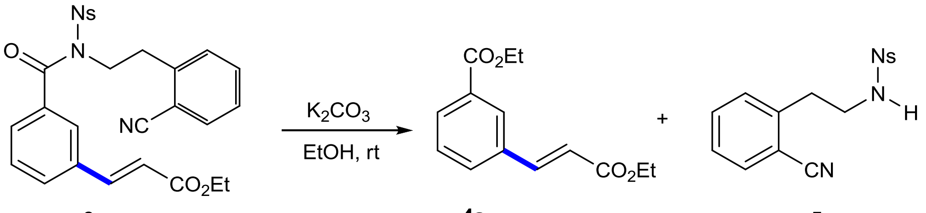

$3a_{mono}$ **$4a_2$** **5**

To a solution of **$3a_{mono}$** (50 mg, 0.094 mmol) in ethanol (1.0 mL) was added K_2CO_3 (35 mg, 0.25 mmol). The mixture was stirred at room temperature for 2 h. The insolubles were filtered off and the filtrate was concentrated under reduced pressure. The residue was purified by silica gel chromatography with petroleum ether/EtOAc (10:1) to afford compound **$4a_2$** (23 mg) in 99% yield. Then with petroleum ether/EtOAc (3:1), the auxiliary **5** can be recovered in 93% yield (29 mg).

Method c:

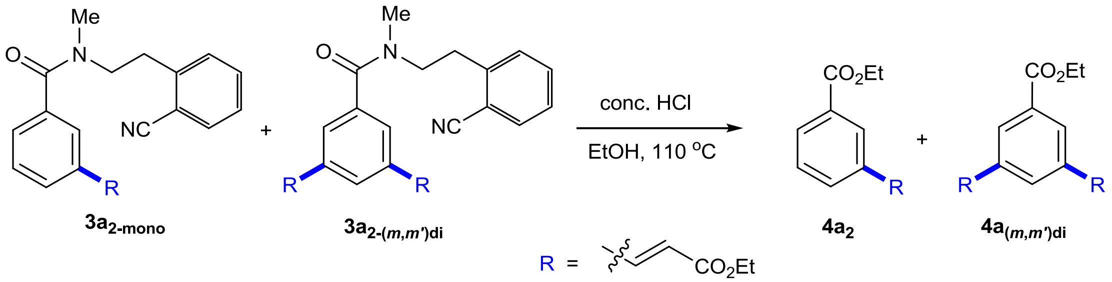

A mixture of compound **3a$_{2\text{-mono}}$** (0.102 mmol) and compound **3a$_{2\text{-}(m,m')\text{di}}$** (0.062 mmol) was dissolved in EtOH (1 mL). To the solution was added HCl (aq., 1 mL, 36 wt%) dropwise at 0 °C. After stirring for 10 min at 0 °C, the reaction was allowed to reflux at 110 °C for 24 h. Then the reaction mixture was diluted with 5 mL of H_2O. After basified with ammonium hydroxide solution at 0 °C until pH value reached about 10, the solution was extracted with EtOAc (10 mL × 2). The combined organic phase was washed with saturated NaCl aqueous solution, dried over Na_2SO_4 and concentrated in *vacuo*. The crude mixture was purified by preparative thin layer chromatography using petroleum ether/EtOAc (10/1) as the eluent to yield **4a$_2$** (20 mg, 79%) and **4a$_{(m,m')\text{di}}$** (16 mg, 74%).

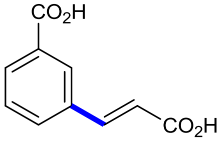

4a$_1$ **(*E*)-3-(2-carboxyvinyl)benzoic acid** (known compound):[8] ^{1}H NMR (400 MHz, CD_3OD) δ 8.20 (s, 1H), 8.03 (d, *J* = 7.6 Hz, 1H), 7.82 (d, *J* = 7.6 Hz, 1H), 7.70 (d, *J* = 16.0 Hz, 1H), 7.51 (t, *J* = 7.7 Hz, 1H), 6.54 (d, *J* = 16.0 Hz, 1H). ^{13}C NMR (101 MHz, CD_3OD) δ 170.0, 169.1, 145.1, 136.3, 133.2, 132.8, 132.3, 130.2, 130.2, 120.7.

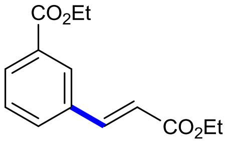

4a$_2$ **(*E*)-ethyl 3-(3-ethoxy-3-oxoprop-1-enyl)benzoate** (known compound):[9] ^{1}H NMR (400 MHz, $CDCl_3$) δ 8.20 (s, 1H), 8.05 (d, *J* = 7.8 Hz, 1H), 7.77-7.65 (m, 2H), 7.46 (t, *J* = 7.7 Hz, 1H), 6.51 (d, *J* = 16.0 Hz, 1H), 4.40 (q, *J* = 7.1 Hz, 2H), 4.28 (q, *J* = 7.1 Hz, 2H), 1.41 (t, *J* = 7.1 Hz, 3H), 1.34 (t, *J* = 7.1 Hz, 3H). ^{13}C NMR (101 MHz, $CDCl_3$) δ 166.8, 166.1, 143.6, 134.9, 132.2, 131.4, 131.1, 129.1, 129.1, 119.7, 61.4, 60.8, 14.5, 14.4.

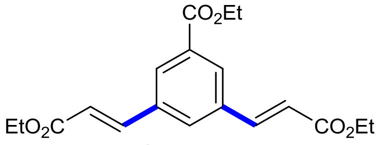

4a$_{(m,m')\text{di}}$ **(2*E*,2'*E*)-diethyl 3,3'-(5-(ethoxycarbonyl)-1,3-phenylene)diacrylate**: ^{1}H NMR (400 MHz, $CDCl_3$) δ 8.20 (d, *J* = 1.3 Hz, 2H), 7.79 (s, 1H), 7.70 (d, *J* = 16.0 Hz, 2H), 6.54 (d, *J* = 16.0 Hz, 2H), 4.42 (q, *J* = 7.1 Hz, 2H), 4.28 (q, *J* = 7.1 Hz, 4H), 1.42 (t, *J* = 7.1 Hz, 3H), 1.35 (t, *J* = 7.1 Hz, 6H).

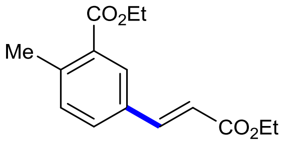

4b **(*E*)-ethyl 5-(3-ethoxy-3-oxoprop-1-enyl)-2-methylbenzoate** (method b): ^{1}H NMR (400 MHz, $CDCl_3$) δ 8.06 (s, 1H), 7.67 (d, *J* = 16.0 Hz, 1H), 7.54 (d, *J* = 7.9 Hz, 1H), 7.31-7.23 (m, 1H), 6.45 (d, *J* = 16.0 Hz, 1H), 4.38 (q, *J* = 7.1 Hz, 2H), 4.27 (q, *J* = 7.1 Hz, 2H), 2.62 (s, 3H), 1.41 (t, *J* = 7.1 Hz, 3H), 1.34 (t, *J* = 7.1 Hz, 3H). ^{13}C NMR (101 MHz, $CDCl_3$) δ 167.2, 167.0, 143.6, 142.5, 132.5, 132.3, 130.9, 130.7, 130.4, 118.6, 61.2, 60.7, 21.8, 14.5.

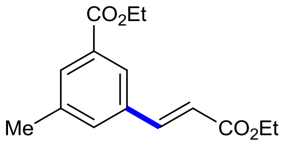

4f **(*E*)-ethyl 3-(3-ethoxy-3-oxoprop-1-enyl)-5-methylbenzoate** (method b): ^{1}H NMR (400 MHz, $CDCl_3$) δ 8.01 (s, 1H), 7.86 (s, 1H), 7.68 (d, *J* = 16.0 Hz, 1H), 7.50 (s, 1H), 6.49 (d, *J* = 16.0 Hz, 1H), 4.39 (q, *J* = 7.1 Hz, 2H), 4.27 (q, *J* = 7.1 Hz, 2H), 2.42 (s, 3H), 1.41 (t, *J* = 7.1 Hz, 3H), 1.34 (t, *J* = 7.1 Hz, 3H). ^{13}C NMR (101 MHz, $CDCl_3$) δ 166.9, 166.4, 143.8, 139.0, 134.8, 133.0, 131.9, 131.3, 126.4, 119.4, 61.3, 60.8, 21.3, 14.5, 14.5.

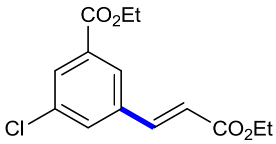

4h **(*E*)-ethyl 3-chloro-5-(3-ethoxy-3-oxoprop-1-enyl)benzoate** (method b): ^{1}H NMR (400 MHz, $CDCl_3$) δ 8.07 (s, 1H), 8.00 (s, 1H), 7.70-7.58 (m, 2H), 6.51 (d, *J* = 16.0 Hz, 1H), 4.40 (q, *J* = 7.1 Hz, 2H), 4.28 (q, *J* = 7.1 Hz, 2H), 1.41 (t, *J* = 7.1 Hz, 3H), 1.34 (t, *J* = 7.1 Hz, 3H). ^{13}C NMR (101 MHz, $CDCl_3$) δ 166.4, 165.0, 142.1, 136.6, 135.3, 132.9, 131.8, 130.9, 127.2, 121.1, 61.8, 61.0, 14.4.

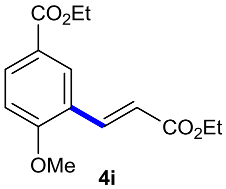

4i **(*E*)-ethyl 3-(3-ethoxy-3-oxoprop-1-enyl)-4-methoxybenzoate** (method b): ^{1}H NMR (400 MHz, $CDCl_3$) δ 8.20 (s, 1H), 8.03 (d, *J* = 8.7 Hz, 1H), 7.96 (d, *J* = 16.2 Hz, 1H), 6.93 (d, *J* = 8.7 Hz, 1H), 6.58 (d, *J* = 16.2 Hz, 1H), 4.36 (q, *J* = 7.1 Hz, 2H), 4.27 (q, *J* = 7.1 Hz, 2H), 3.94 (s, 3H), 1.39 (t, *J* = 7.1 Hz, 3H), 1.34 (t, *J* = 7.1 Hz, 3H). ^{13}C NMR (101 MHz, $CDCl_3$) δ 167.3, 166.1, 161.6, 139.1, 133.1, 130.5, 123.5, 123.2, 120.0, 110.8, 61.1, 60.6, 56.0, 14.5, 14.5.

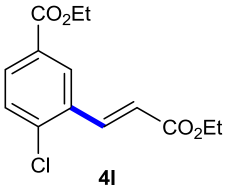

4l **(*E*)-ethyl 4-chloro-3-(3-ethoxy-3-oxoprop-1-enyl)benzoate** (method b): ^{1}H NMR (400 MHz, $CDCl_3$) δ 8.28 (d, *J* = 1.7 Hz, 1H), 8.06 (d, *J* = 16.0 Hz, 1H), 7.95 (dd, *J* = 8.4, 1.9 Hz, 1H), 7.48 (d, *J* = 8.4 Hz, 1H), 6.53 (d, *J* = 16.0 Hz, 1H), 4.39 (q, *J* = 7.1 Hz, 2H), 4.29 (q, *J* = 7.1 Hz, 2H), 1.40 (t, *J* = 7.1 Hz, 3H), 1.35 (t, *J* = 7.1 Hz, 3H). ^{13}C NMR (101 MHz, $CDCl_3$) δ 166.4, 165.4, 139.5, 139.5, 133.1, 131.6, 130.4, 129.7, 128.9, 122.3, 61.6, 61.0, 14.4, 14.4.

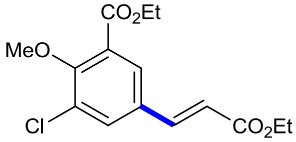

4n **(*E*)-ethyl 3-chloro-5-(3-ethoxy-3-oxoprop-1-enyl)-2-methoxybenzoate** (method b): ^{1}H NMR (400 MHz, $CDCl_3$) δ 7.83 (d, *J* = 2.1 Hz, 1H), 7.69 (d, *J* = 2.1 Hz, 1H), 7.57 (d, *J* = 16.0 Hz, 1H), 6.40 (d, *J* = 16.0 Hz, 1H), 4.40 (q, *J* = 7.1 Hz, 2H), 4.27 (q, *J* = 7.1 Hz, 2H), 3.96 (s, 3H), 1.41 (t, *J* = 7.1 Hz, 3H), 1.33 (t, *J* = 7.1 Hz, 3H). ^{13}C NMR (101 MHz, $CDCl_3$) δ 166.5, 165.0, 157.0, 141.7, 132.8, 131.2, 130.4, 129.5, 127.8, 120.0, 62.3, 61.9, 60.9, 14.4, 14.4.

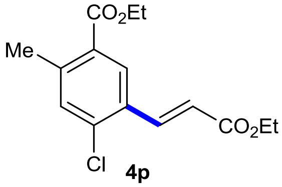

4p **(*E*)-ethyl 4-chloro-5-(3-ethoxy-3-oxoprop-1-enyl)-2-methylbenzoate** (method b): ^{1}H NMR (400 MHz, $CDCl_3$) δ 8.18 (s, 1H), 8.02 (d, *J* = 16.0 Hz, 1H), 7.31 (s, 1H), 6.49 (d, *J* = 16.0 Hz, 1H), 4.37 (q, *J* = 6.8 Hz, 2H), 4.29 (q, *J* = 6.8 Hz, 2H), 2.58 (s, 3H), 1.41 (t, *J* = 6.9 Hz, 3H), 1.35 (t, *J* = 7.2 Hz, 3H). ^{13}C NMR (101 MHz, $CDCl_3$) δ 166.6, 166.4, 143.5, 139.5, 138.1, 133.2, 130.4, 130.0, 129.1, 121.2, 61.4, 60.9, 21.7, 14.5, 14.5.

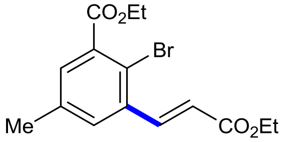

4r **(*E*)-ethyl 2-bromo-3-(3-ethoxy-3-oxoprop-1-enyl)-5-methylbenzoate** (method b): ^{1}H NMR (400 MHz, $CDCl_3$) δ 8.09 (d, *J* = 15.9 Hz, 1H), 7.46 (s, 1H), 7.43 (s, 1H), 6.34 (d, *J* = 15.9 Hz, 1H), 4.40 (q, *J* = 7.1 Hz, 2H), 4.28 (q, *J* = 7.1 Hz, 2H), 2.35 (s, 3H), 1.41 (t, *J* = 7.1 Hz, 3H), 1.34 (t, *J* = 7.1 Hz, 3H). ^{13}C NMR (101 MHz, $CDCl_3$) δ 167.1, 166.4, 143.3, 137.6, 136.3, 135.3, 132.1, 130.8, 122.2, 119.9, 62.0, 60.9, 20.9, 14.4, 14.3.

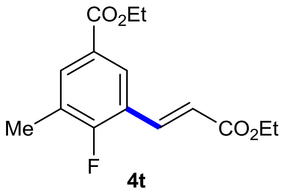

4t **(*E*)-ethyl 3-(3-ethoxy-3-oxoprop-1-enyl)-4-fluoro-5-methylbenzoate** (method b): ^{1}H NMR (400 MHz, $CDCl_3$) δ 8.08 (dd, *J* = 6.5, 1.9 Hz, 1H), 7.90 (d, *J* = 6.9 Hz, 1H), 7.82 (d, *J* = 16.2 Hz, 1H), 6.59 (d, *J* = 16.2 Hz, 1H), 4.38 (q, *J* = 7.1 Hz, 2H), 4.28 (q, *J* = 7.1 Hz, 2H), 2.34 (d, *J* = 2.0 Hz, 3H), 1.40 (t, *J* = 7.1 Hz, 3H), 1.35 (t, *J* = 7.1 Hz, 3H). ^{13}C NMR (101 MHz, $CDCl_3$) δ 166.8, 165.6, 162.5 (d, $J_{C\text{-}F}$ = 260 Hz), 136.6 (d, $J_{C\text{-}F}$ = 3.6 Hz), 134.4 (d, $J_{C\text{-}F}$ = 7.0 Hz), 128.2 (d, $J_{C\text{-}F}$ = 4.0 Hz), 126.6 (d, $J_{C\text{-}F}$ = 3.8 Hz), 126.2 (d, $J_{C\text{-}F}$ = 18.7 Hz), 122.5 (d, $J_{C\text{-}F}$ = 13.6 Hz), 121.8 (d, $J_{C\text{-}F}$ = 6.1 Hz), 61.5, 60.9, 14.70 (d, $J_{C\text{-}F}$ = 4.4 Hz), 14.5, 14.4.

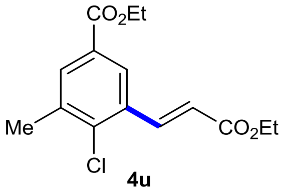

4u **(*E*)-ethyl 4-chloro-3-(3-ethoxy-3-oxoprop-1-enyl)-5-methylbenzoate** (method b): ^{1}H NMR (400 MHz, $CDCl_3$) δ 8.19-8.06 (m, 2H), 7.91 (s, 1H), 6.49 (d, *J* = 16.0 Hz, 1H), 4.38 (q, *J* = 7.1 Hz, 2H), 4.29 (q, *J* = 7.1 Hz, 2H), 2.45 (s, 3H), 1.40 (t, *J* = 7.1 Hz, 3H), 1.35 (t, *J* = 7.1 Hz, 3H). ^{13}C NMR (101 MHz, $CDCl_3$) δ 166.5, 165.7, 140.5, 139.6, 137.8, 133.5, 132.7, 128.9, 126.4, 122.1, 61.5, 60.9, 20.9, 14.5, 14.4.

Supplementary References

1 Anderson, K. W., Ikawa, T., Tundel, R. E. & Buchwald, S. L. The selective reaction of aryl halides with KOH: Synthesis of phenols, aromatic ethers, and benzofurans. *J. Am. Chem. Soc.* **128**, 10694-10695 (2006).

2 Merck Patent GMBH, Patent: WO2003/76406 A1, **2003**.

3 Smithkline Beecham Corporation, Patent: WO2007/143456 A2, **2007**, p127.

4 Yung, H. C., Patent: US2009/105275 A1, **2009**, p30.

5 Chugai Seiyaku Kabushiki Kaisha, Patent: EP1894911 A1, **2008**, p25-26.

6 Willardsen, J. A. *et al*. Design, synthesis, and biological activity of potent and selective inhibitors of blood coagulation factor Xa. *J. Med. Chem.* **47**, 4089-4099 (2004).

7 Torisu, K. *et al*. Development of prostaglandin D_2 receptor antagonist: discovery of highly potent antagonists. *Bioorg. Med. Chem.* **12**, 4685-4700 (2004).

8 Hubrich, F., Mordhorst, S. & Andexer, J. N. Cinnamic acid derivatives as inhibitors for chorismatases and isochorismatases. *Bioorg. Med. Chem. Lett.* **23**, 1477-1481 (2013).

9 Saito, S. *et al*. Enhanced reactivity of electron-deficient enynes in the palladium-catalyzed *homo*-benzannulation of conjugated enynes. *J. Org. Chem.* **65**, 5350-5354 (2000).
